# Sequence Relationships among *C. elegans*, *D. melanogaster* and Human microRNAs Highlight the Extensive Conservation of microRNAs in Biology

**DOI:** 10.1371/journal.pone.0002818

**Published:** 2008-07-30

**Authors:** Carolina Ibáñez-Ventoso, Mehul Vora, Monica Driscoll

**Affiliations:** Department of Molecular Biology and Biochemistry, Rutgers, The State University of New Jersey, Piscataway, New Jersey, United States of America; University of Maryland, United States of America

## Abstract

microRNAs act in a prevalent and conserved post-transcriptional gene regulatory mechanism that impacts development, homeostasis and disease, yet biological functions for the vast majority of miRNAs remain unknown. Given the power of invertebrate genetics to promote rapid evaluation of miRNA function, recently expanded miRNA identifications (miRBase 10.1), and the importance of assessing potential functional redundancies within and between species, we evaluated miRNA sequence relationships by 5′ end match and overall homology criteria to compile a snapshot overview of miRNA families within the *C. elegans* and *D. melanogaster* genomes that includes their identified human counterparts. This compilation expands literature documentation of both the number of families and the number of family members, within and between nematode and fly models, and highlights sequences conserved between species pairs or among nematodes, flies and humans. Themes that emerge include the substantial potential for functional redundancy of miRNA sequences within species (84/139 *C. elegans* miRNAs and 70/152 *D. melanogaster* miRNAs share significant homology with other miRNAs encoded by their respective genomes), and the striking extent to which miRNAs are conserved across species—over half (73/139) *C. elegans* miRNAs share sequence homology with miRNAs encoded also in both fly and human genomes. This summary analysis of mature miRNA sequence relationships provides a quickly accessible resource that should facilitate functional and evolutionary analyses of miRNAs and miRNA families.

## Introduction

microRNAs (miRNAs) are small (16–29 nucleotide (nt)), non-coding RNAs that regulate gene expression at the post-transcriptional level [1]–[5]. Intensive research over the last several years has led to the appreciation that these tiny RNAs act via a highly prevalent, and generally conserved, gene expression regulatory mechanism that impacts development, homeostasis and disease. A major research challenge for the decade will be the elaboration of miRNA function in biology and the investigation of how microRNAs can be exploited for therapeutic application.

To date, little is actually known about the biological functions of most miRNAs—the roles of only a small number have been experimentally elucidated [6], [7]. Numerous studies have reported on miRNA expression profiles in cells, tissues, organisms, and disease states [8]–[31]. In addition, multiple bioinformatic efforts have predicted target mRNA transcripts to suggest candidate genes regulated by miRNA interactions e.g. [13], [32], [33]–[38]. The potential for complex cross-regulation that emerges from these general surveys is staggering, and appreciation for the complexity is further extended by observations that: 1) many mRNA transcripts include potential binding sites for multiple, distinct miRNAs, and 2) different miRNAs that share sequence similarity (especially in the 5′ end seed region) can recognize the same binding sites on individual mRNA targets [39]–[42]. Against this backdrop, the need for understanding when and where miRNAs are expressed, what the relevant mRNA targets are, and what the complete miRNA family sequence relationships encoded by the genome are, is dramatically underscored. This work addresses the latter goal, with an emphasis on invertebrate genetic models that are likely to have a major impact on advancing understanding of miRNA function.

Over the last few years, intensive discovery efforts have contributed to extensive additions and sequence changes to annotated miRBase miRNA compilations for *C. elegans*, *D. melanogaster* and humans as total numbers of mature miRNA sequences increased from 107 *C. elegans*, 79 *D. melanogaster* and 152 human (miRBase release 3.0, Jan. 2004) to 139 *C. elegans*, 152 *D. melanogaster* and 733 human (miRBase release 10.1, December 2007) [43]–[46]. Although it is anticipated that miRNAs will continue to be identified, (numbers of human miRNAs may be in the thousands (see Bentwich et al. [47]), it is likely that most of the abundant miRNAs have been identified in nematodes, flies and humans. Moreover, the majority of identified *C. elegans* miRNAs have been genetically deleted [48]–[50], an accomplishment that sets the stage for detailed evaluation of functions in this model. Initial studies support that evaluation of functional redundancies will be an important factor in this analysis [39], [40], [42], [48] and that conserved regulatory functions may shed light on disease mechanisms [6], [42]. Thus, we considered it a timely moment to pause and compile an overview of sequence miRNA relationships in invertebrate genetic models.

Given the expanded *C. elegans*, *D. melanogaster* and human miRNA identifications and the importance of rapidly identifying potential functional redundancies within and between species, we probed miRNA sequence relationships to compile a current list of mature miRNA sequence families within the *C. elegans* and *D. melanogaster* genomes, and we identified their human counterparts. Our analysis presents an overview that significantly expands the memberships of described sequence-related groups within, and between, species. We highlight new sequences conserved between species pairs or among nematodes, flies and humans. This compilation of sequence relationships should facilitate studies on miRNA evolution and conserved function that will contribute to enhanced understanding of complex miRNA regulatory networks and their biological activities.

## Results

Recent reports have markedly expanded the numbers of identified miRNAs expressed in *C. elegans*, *D. melanogaster*, and humans [10], [11], [25], [47], [51]–[69]. Given the tremendous potential of invertebrate genetics to address *in vivo* function of conserved miRNAs, the availability of genetic knockouts of most of the 139 reported *C. elegans* miRNA genes, and our interest in evaluating miRNA contributions to cellular robustness and mechanisms of aging, we sought to generate a current overview of miRNA sequence families identifiable by comparisons among these species. We compared all reported *C. elegans* 139, *D. melanogaster* 152 and human 733 mature miRNA sequences available in the miRNA database miRBase 10.1 [43]–[46] using the ClustalW algorithm [70] to identify intra-species and inter-species sequence similarities. We classified miRNAs as sequence-related based on current understanding of functional miRNA-target interactions, which may occur via either of two sequence relationships: 1) perfect complementarity of miRNA 5′ end sequences, especially at nucleotide positions 1,2–8 referred to as the “seed” region, and 2) high level complementarity across the length of the miRNA (>70% identity overall) that can have less precise pairing in the seed region.

### 5′ end seed region search criteria

5′ end sequences are critical for miRNA function [2], [41], [71]–[73] and the seed region is thought to contribute to target recognition by perfect (or near perfect) complementary binding to the mRNA target site. The requirement for uninterrupted homology may render the miRNA-mRNA seed region under considerable selective pressure. Indeed, seed regions are highly conserved in mRNAs across species [32], [41], [74]. We therefore searched for 5′ end seed matches that featured at least 7 continuous nucleotides of homology within the first 10 nt of the miRNA, a modestly relaxed criteria chosen to provide confidence that most potential seed region sequence relationships would be identified by this search. We did not allow interruptions (mismatches or gaps) within the first 10 nt except base changes that would permit G..U pairing, because G..U base pairing in the seed region has been documented to be possible *in vivo* under conditions of efficient miRNA target regulation [73].

### Homology over miRNA length criteria

Because some miRNAs have less stringent seed region pairing and instead exhibit homology to target transcripts over their lengths [41], [71], we also compiled a list of related miRNAs using the criteria of full-span homology. To establish a reasonable homology cut-off value, we examined previously identified miRNA families and determined that a score of ≥70% sequence similarity over length should identify most, if not all, miRNA homologs known from published target site models and miRNA groups. Because the current mechanism of action of miRNAs has been inferred from only very few validated miRNA-gene targets, we also elected to list miRNAs that exhibit 60–69.9% identity in supporting information. According to current understanding, the potential functional significance of the 60–69.9% similar miRNAs is of higher probability if the homology in the seed region is high.

### Overview: Sequence relationships that expand miRNA family lists in nematodes, flies and humans

We performed alignments by both 5′ end seed matching (nt 1–10) and by analysis of homology across complete mature miRNA sequences, comparing individual *C. elegans* miRNA sequences against all known *C. elegans*, *D. melanogaster* and human miRNAs, and individual *D. melanogaster* miRNAs against *D. melanogaster* and human miRNAs (Tables 1–6). Our analysis greatly expands the documentation of miRNA sequence family members. For example, our combined list of *C. elegans* miRNA sequence relationships identified 211 sequence relationships, placing 84 sequence-related miRNAs into 20 family groups (Table 1), whereas previous reports on *C. elegans* miRNAs [43], [51], [63], [64] identified 110 sequence relationships between 70 miRNAs. Similarly, our combined searches for *D. melanogaster* miRNAs detected 126 sequence relationships including 70 sequence-related miRNAs in 24 family groups (Table 2), a considerable expansion of the previously reported 53 sequence relationships between 31 miRNAs [25], [43], [52], [57], [60], [65], [75]. Our work increases the number of *C. elegans* miRNAs with identified human counterparts to 76 (Table 4) and *D. melanogaster* miRNAs with identified human counterparts to 83 (Table 5) [25], [43], [51], [52], [57], [60], [63]–[65], [75], [76]. Significantly, we associated as many as 133 human miRNA sequences with sequence-related worm and/or fly miRNAs (Tables 4–6 and Figure S1). Below we highlight some details of *C. elegans* and *D. melanogaster* miRNA family searches and then discuss the invertebrate miRNAs that have clear human counterparts.

**Table 1:** Summary of *C. elegans* miRNA relationships.

miRNA Group ID	Sequence-Related miRNAs	
	5′ Sequence	Full Sequence
let-7	cel-let-7	cel-miR-84
	cel-miR-48	
	cel-miR-84	
	cel-miR-241	
	cel-miR-793	
	cel-miR-794	
	cel-miR-795	
lin-4	cel-lin-4	
	cel-miR-237	
miR-2	cel-miR-2	cel-miR-43
	cel-miR-43	
	cel-miR-250	
	cel-miR-797	
miR-35	cel-miR-35	cel-miR-36
		cel-miR-37
		cel-miR-38
		cel-miR-39
		cel-miR-271
	cel-miR-36	cel-miR-37
		cel-miR-39
		cel-miR-41
	cel-miR-37	cel-miR-38
		cel-miR-42
	cel-miR-38	
	cel-miR-39	cel-miR-40
		cel-miR-41
	cel-miR-40	cel-miR-41
		cel-miR-42
	cel-miR-41	
	cel-miR-42	
	cel-miR-271	
miR-44	cel-miR-44	cel-miR-45
	cel-miR-45	
	cel-miR-61	cel-miR-247
	cel-miR-247	
miR-46	cel-miR-46	cel-miR-47
	cel-miR-47	
miR-49	cel-miR-49	
	cel-miR-83	
miR-50	cel-miR-50	
	cel-miR-62	
	cel-miR-90	
miR-51	cel-miR-51	
	cel-miR-52	cel-miR-53
		cel-miR-56
	cel-miR-53	
	cel-miR-54	cel-miR-56
	cel-miR-55	cel-miR-56
	cel-miR-56	cel-miR-273
	cel-miR-267	
	cel-miR-273	
	cel-miR-360	
miR-63	cel-miR-63	cel-miR-64
		cel-miR-65
	cel-miR-64	cel-miR-65
	cel-miR-65	
	cel-miR-66	
	cel-miR-228	
	cel-miR-229	
	cel-miR-790	
	cel-miR-791	
miR-75	cel-miR-75	
	cel-miR-79	
miR-78		cel-miR-78
		cel-miR-272
miR-80	cel-miR-58	
	cel-miR-80	cel-miR-82
	cel-miR-81	cel-miR-82
	cel-miR-82	
	cel-miR-1018	
	cel-miR-1022	
miR-86	cel-miR-86	
	cel-miR-785	
miR-231	cel-miR-231	
	cel-miR-787	
miR-233	cel-miR-87	
	cel-miR-233	
	cel-miR-356	
miR-239a	cel-miR-238	
	cel-miR-239a	cel-miR-239b
	cel-miR-239b	
miR-251	cel-miR-251	cel-miR-252
	cel-miR-252	
miR-256	cel-miR-1	cel-miR-256
	cel-miR-232	
	cel-miR-256	
	cel-miR-357	
	cel-miR-796	
miR-266	cel-miR-72	cel-miR-266
	cel-miR-73	cel-miR-268
		cel-miR-270
	cel-miR-74	
	cel-miR-266	cel-miR-269
	cel-miR-268	
	cel-miR-269	

84/139 *C. elegans* miRNAs can be classified into 20 groups that share either identity at the 5′ end (81 miRNAs, listed separately in Dataset S1) and/or homology over sequence length (45 miRNAs with ≥70%, listed separately in Dataset S2). See text and Methods for explanation of match criteria. The miRNA group ID chosen is the miRNA closest to the consensus sequence of the grouped related miRNAs. Less closely related *C. elegans* miRNAs with 60–69.9% sequence similarity over full length are listed in Dataset S3.

**Table 2 pone-0002818-t002:** Summary of *D. melanogaster* miRNA relationships.

miRNA Group ID	Sequence-Related miRNAs	
	5′ Sequence	Full Sequence
bantam	dme-bantam	
	dme-miR-306*	
let-7	dme-let-7	
	dme-miR-963	
	dme-miR-977	
	dme-miR-984	
miR-2a	dme-miR-2a	dme-miR-2b
		dme-miR-2c
		dme-miR-13a
		dme-miR-13b
	dme-miR-2b	dme-miR-2c
		dme-miR-13a
		dme-miR-13b
	dme-miR-2c	dme-miR-13a
		dme-miR-13b
	dme-miR-6	
	dme-miR-11	
	dme-miR-13a	dme-miR-13b
	dme-miR-13b	
	dme-miR-308	
miR-3	dme-miR-3	dme-miR-309
		dme-miR-318
	dme-miR-309	
	dme-miR-318	
miR-9a	dme-miR-9a	dme-miR-9b
		dme-miR-9c
	dme-miR-9b	dme-miR-9c
	dme-miR-9c	
miR-10		dme-miR-10
		dme-miR-100
miR-12	dme-miR-12	
	dme-miR-280	
	dme-miR-283	
	dme-miR-289	
	dme-miR-960	
miR-14	dme-miR-14	
	dme-miR-316	
miR-31a	dme-miR-31a	dme-miR-31b
	dme-miR-31b	
miR-219	dme-miR-219	
	dme-miR-315	
miR-263a		dme-miR-263a
		dme-miR-263b
miR-275	dme-miR-275	
	dme-miR-306	
	dme-miR-967	
miR-276a	dme-miR-276a	dme-miR-276b
	dme-miR-276b	
miR-279	dme-miR-279	
	dme-miR-286	
	dme-miR-996	
miR-281-2*	dme-miR-4	
	dme-miR-7	
	dme-miR-79	
	dme-miR-281-1*	dme-miR-281-2*
	dme-miR-281-2*	
miR-285	dme-miR-285	dme-miR-998
	dme-miR-995	dme-miR-998
	dme-miR-998	
miR-312	dme-miR-92a	dme-miR-92b
		dme-miR-310
		dme-miR-312
	dme-miR-92b	dme-miR-310
		dme-miR-312
	dme-miR-310	dme-miR-311
	dme-miR-311	dme-miR-312
		dme-miR-313
	dme-miR-312	dme-miR-313
	dme-miR-313	
miR-954		dme-miR-954
		dme-miR-966
miR-1003	dme-miR-1003	
	dme-miR-1004	
miR-1006	dme-miR-1006	
	dme-miR-1014	
miR-1009		dme-miR-1009
		dme-miR-1010
miR-1010	dme-miR-1010	
	dme-miR-1016	
miR-iab4as-3p		dme-miR-iab4as-3p
		dme-miR-iab-4-3p
miR-iab4as-5p	dme-miR-iab4as-5p	dme-miR-iab-4-5p
	dme-miR-iab-4-5p	

70/152 *Drosophila* miRNAs can be arranged into 24 groups that have sequence homology at the 5′ end (61 miRNAs, listed separately in Dataset S4) and/or over their entire length (38 miRNAs, listed separately in Dataset S5). See Methods and text for explanation of match criteria. The miRNA group ID was chosen as the miRNA closest to the consensus sequence of the grouped related miRNAs. *D. melanogaster* miRNAs with 60–69.9% similarity over their full sequence are listed in Dataset S6.

**Table 3 pone-0002818-t003:** Combined searches for 5′ and ≥70% full sequence similarities detect 87 miRNA families containing 87 *C. elegans* miRNAs and 65 *D. melanogaster* miRNAs.

miRNA Group ID	*C. elegans* miRNA	Sequence-related *Drosophila* miRNAs	
		5′ Sequence	Full Sequence
let-7	cel-let-7	dme-let-7	dme-let-7
		dme-miR-963	dme-miR-984
		dme-miR-977	
		dme-miR-984	
lin-4	cel-lin-4	dme-miR-125	dme-miR-125
miR-1	cel-miR-1	dme-miR-1	dme-miR-1
miR-2	cel-miR-2	dme-miR-2a	dme-miR-2a
		dme-miR-2b	dme-miR-2b
		dme-miR-2c	dme-miR-2c
		dme-miR-6	dme-miR-13a
		dme-miR-11	dme-miR-13b
		dme-miR-13a	
		dme-miR-13b	
		dme-miR-308	
miR-34	cel-miR-34	dme-miR-34	dme-miR-34
miR-43	cel-miR-43	dme-miR-2a	
		dme-miR-2b	
		dme-miR-2c	
		dme-miR-6	
		dme-miR-11	
		dme-miR-13a	
		dme-miR-13b	
		dme-miR-308	
miR-44	cel-miR-44	dme-miR-279	
		dme-miR-286	
		dme-miR-996	
miR-45	cel-miR-45	dme-miR-279	
		dme-miR-286	
		dme-miR-996	
miR-46	cel-miR-46	dme-miR-281	dme-miR-281
miR-47	cel-miR-47	dme-miR-281	dme-miR-281
miR-48	cel-miR-48	dme-let-7	
		dme-miR-963	
		dme-miR-977	
		dme-miR-984	
miR-49	cel-miR-49	dme-miR-285	
		dme-miR-995	
		dme-miR-998	
miR-50	cel-miR-50	dme-miR-190	dme-miR-190
miR-51	cel-miR-51	dme-miR-100	
miR-52	cel-miR-52	dme-miR-100	
miR-53	cel-miR-53	dme-miR-100	
miR-54	cel-miR-54	dme-miR-100	
miR-55	cel-miR-55	dme-miR-100	
miR-56	cel-miR-56	dme-miR-100	
miR-57	cel-miR-57	dme-miR-10	
miR-58	cel-miR-58	dme-bantam	
		dme-miR-306*	
miR-61	cel-miR-61	dme-miR-279	
		dme-miR-286	
		dme-miR-996	
miR-62	cel-miR-62	dme-miR-190	
miR-63	cel-miR-63	dme-miR-263b	
miR-64	cel-miR-64	dme-miR-263b	
miR-65	cel-miR-65	dme-miR-263b	
miR-66	cel-miR-66	dme-miR-263b	
miR-67	cel-miR-67	dme-miR-307	dme-miR-307
miR-72	cel-miR-72	dme-miR-31a	dme-miR-31a
		dme-miR-31b	dme-miR-31b
miR-73	cel-miR-73	dme-miR-31a	dme-miR-31a
		dme-miR-31b	
miR-74	cel-miR-74	dme-miR-31a	
		dme-miR-31b	
miR-75	cel-miR-75	dme-miR-4	
		dme-miR-79	
		dme-miR-281-1*	
		dme-miR-281-2*	
miR-76	cel-miR-76	dme-miR-981	dme-miR-981
miR-79	cel-miR-79	dme-miR-4	dme-miR-79
		dme-miR-7	
		dme-miR-79	
		dme-miR-281-1*	
		dme-miR-281-2*	
miR-80	cel-miR-80	dme-bantam	dme-bantam
		dme-miR-306*	
miR-81	cel-miR-81	dme-bantam	dme-bantam
		dme-miR-306*	
miR-82	cel-miR-82	dme-bantam	dme-bantam
		dme-miR-306*	
miR-83	cel-miR-83	dme-miR-285	dme-miR-285
		dme-miR-995	dme-miR-998
		dme-miR-998	
miR-84	cel-miR-84	dme-let-7	dme-let-7
		dme-miR-963	
		dme-miR-977	
		dme-miR-984	
miR-86	cel-miR-86	dme-miR-987	
miR-87	cel-miR-87	dme-miR-87	dme-miR-87
miR-90	cel-miR-90	dme-miR-190	
miR-124	cel-miR-124	dme-miR-124	dme-miR-124
miR-228	cel-miR-228	dme-miR-263a	dme-miR-263a
		dme-miR-263b	
miR-229	cel-miR-229	dme-miR-263a	
		dme-miR-263b	
miR-231	cel-miR-231	dme-miR-993	
miR-232	cel-miR-232	dme-miR-277	
miR-233	cel-miR-233	dme-miR-87	
miR-234	cel-miR-234	dme-miR-137	dme-miR-137
miR-235	cel-miR-235	dme-miR-92a	dme-miR-92a
		dme-miR-92b	dme-miR-92b
		dme-miR-310	dme-miR-310
		dme-miR-311	dme-miR-311
		dme-miR-312	dme-miR-312
		dme-miR-313	dme-miR-313
miR-236	cel-miR-236	dme-miR-8	dme-miR-8
miR-237	cel-miR-237	dme-miR-125	
miR-238	cel-miR-238	dme-miR-305	
miR-239a	cel-miR-239a	dme-miR-305	dme-miR-12
miR-239b	cel-miR-239b	dme-miR-305	
miR-240	cel-miR-240	dme-miR-193	
miR-241	cel-miR-241	dme-let-7	
		dme-miR-963	
		dme-miR-977	
		dme-miR-984	
miR-244	cel-miR-244	dme-miR-9a	
		dme-miR-9b	
		dme-miR-9c	
miR-245	cel-miR-245	dme-miR-133	dme-miR-133
miR-247	cel-miR-247	dme-miR-279	dme-miR-996
		dme-miR-286	
		dme-miR-996	
miR-249	cel-miR-249	dme-miR-308	
miR-250	cel-miR-250	dme-miR-2a	dme-miR-1007
		dme-miR-2b	
		dme-miR-2c	
		dme-miR-6	
		dme-miR-11	
		dme-miR-13a	
		dme-miR-13b	
		dme-miR-308	
miR-251	cel-miR-251	dme-miR-1002	
miR-252	cel-miR-252	dme-miR-1002	dme-miR-252
miR-256	cel-miR-256	dme-miR-1	dme-miR-1
		dme-miR-277	
miR-259	cel-miR-259	dme-miR-304	
miR-260	cel-miR-260	dme-miR-989	
miR-266	cel-miR-266	dme-miR-31a	
		dme-miR-31b	
miR-267	cel-miR-267	dme-miR-100	
miR-268	cel-miR-268	dme-miR-31a	
		dme-miR-31b	
miR-269	cel-miR-269	dme-miR-31a	
		dme-miR-31b	
miR-273	cel-miR-273	dme-miR-100	
miR-356	cel-miR-356	dme-miR-87	
miR-357	cel-miR-357	dme-miR-277	
miR-358	cel-miR-358	dme-miR-9c	
miR-359	cel-miR-359	dme-miR-3	
		dme-miR-318	
miR-785	cel-miR-785	dme-miR-987	
miR-787	cel-miR-787	dme-miR-993	
miR-790	cel-miR-790	dme-miR-263b	
miR-791	cel-miR-791	dme-miR-263b	
miR-793	cel-miR-793	dme-let-7	
		dme-miR-977	
		dme-miR-984	
miR-794	cel-miR-794	dme-let-7	dme-miR-977
		dme-miR-963	
		dme-miR-977	
		dme-miR-984	
miR-795	cel-miR-795	dme-let-7	
		dme-miR-963	
		dme-miR-977	
		dme-miR-984	
miR-796	cel-miR-796	dme-miR-1	
miR-797	cel-miR-797	dme-miR-2a	
		dme-miR-2b	
		dme-miR-2c	
		dme-miR-6	
		dme-miR-11	
		dme-miR-13a	
		dme-miR-13b	
		dme-miR-308	
miR-1018	cel-miR-1018	dme-bantam	
miR-1022	cel-miR-1022	dme-bantam	
		dme-miR-306*	

87 *C. elegans* miRNAs are 5′ related to 62 *Drosophila* miRNAs (sequence alignments shown in Dataset S7), whereas 31 *C. elegans* miRNAs have ≥70% overall similarity to 37 *Drosophila* miRNAs (Dataset S8). Group IDs correspond to *C. elegans* miRNAs with sequence-related miRNAs in *Drosophila*. Nematode-fly miRNAs with weaker identity (60–69.9%) over full sequence are listed in Dataset S9.

**Table 4 pone-0002818-t004:** Analysis of 5′ and ≥70% full sequences identifies 76 *C. elegans*-human miRNA families including 76 worm miRNAs and 102 human miRNAs.

miRNA Group ID	*C. elegans* miRNA	Human related miRNAs	
		5′ Sequence	Full Sequence
let-7	cel-let-7	hsa-let-7a	hsa-let-7a
		hsa-let-7b	hsa-let-7b
		hsa-let-7c	hsa-let-7c
		hsa-let-7d	hsa-let-7d
		hsa-let-7e	hsa-let-7e
		hsa-let-7f	hsa-let-7f
		hsa-let-7g	hsa-let-7g
		hsa-let-7i	hsa-let-7i
		hsa-miR-98	hsa-miR-98
		hsa-miR-196a	
		hsa-miR-196b	
lin-4	cel-lin-4	hsa-miR-125a-5p	hsa-miR-125a-5p
		hsa-miR-125b	hsa-miR-125b
		hsa-miR-331-3p	
miR-1	cel-miR-1	hsa-miR-1	hsa-miR-1
		hsa-miR-122	hsa-miR-206
		hsa-miR-206	
miR-2	cel-miR-2	hsa-miR-499-3p	
miR-34	cel-miR-34	hsa-miR-34a	hsa-miR-34a
		hsa-miR-34b*	hsa-miR-34b*
		hsa-miR-34c-5p	hsa-miR-34c-5p
		hsa-miR-449a	hsa-miR-449a
		hsa-miR-449b	hsa-miR-449b
miR-43	cel-miR-43	hsa-miR-27a	
		hsa-miR-27b	
		hsa-miR-128	
		hsa-miR-499-3p	
		hsa-miR-768-3p	
miR-44	cel-miR-44	hsa-miR-134	
		hsa-miR-708*	
miR-45	cel-miR-45	hsa-miR-134	
		hsa-miR-708*	
miR-48	cel-miR-48	hsa-let-7a	
		hsa-let-7b	
		hsa-let-7c	
		hsa-let-7d	
		hsa-let-7e	
		hsa-let-7f	
		hsa-let-7g	
		hsa-let-7i	
		hsa-miR-98	
miR-49	cel-miR-49	hsa-miR-21*	
		hsa-miR-29a	
		hsa-miR-29b	
		hsa-miR-29c	
		hsa-miR-593*	
miR-50	cel-miR-50	hsa-miR-190	hsa-miR-190
		hsa-miR-190b	hsa-miR-190b
miR-51	cel-miR-51	hsa-miR-99a	hsa-miR-99a
		hsa-miR-99b	
		hsa-miR-100	
miR-52	cel-miR-52	hsa-miR-99a	
		hsa-miR-99b	
		hsa-miR-100	
miR-53	cel-miR-53	hsa-miR-99a	
		hsa-miR-99b	
		hsa-miR-100	
miR-54	cel-miR-54	hsa-miR-99a	
		hsa-miR-99b	
		hsa-miR-100	
miR-55	cel-miR-55	hsa-miR-99a	
		hsa-miR-99b	
		hsa-miR-100	
miR-56	cel-miR-56	hsa-miR-99a	
		hsa-miR-99b	
		hsa-miR-100	
miR-57	cel-miR-57	hsa-miR-10a	hsa-miR-10a
		hsa-miR-10b	hsa-miR-10b
		hsa-miR-146b-3p	hsa-miR-99a
			hsa-miR-100
miR-58	cel-miR-58	hsa-miR-450b-3p	
miR-61	cel-miR-61	hsa-miR-134	
		hsa-miR-708*	
miR-62	cel-miR-62	hsa-miR-190	
		hsa-miR-190b	
miR-63	cel-miR-63	hsa-miR-96	
		hsa-miR-183	
		hsa-miR-200a	
		hsa-miR-514	
miR-64	cel-miR-64	hsa-miR-96	
		hsa-miR-183	
		hsa-miR-200a	
		hsa-miR-514	
miR-65	cel-miR-65	hsa-miR-96	
		hsa-miR-183	
		hsa-miR-200a	
		hsa-miR-514	
miR-66	cel-miR-66	hsa-miR-96	
		hsa-miR-183	
		hsa-miR-200a	
		hsa-miR-514	
miR-72	cel-miR-72	hsa-miR-31	hsa-miR-31
miR-73	cel-miR-73	hsa-miR-31	
miR-74	cel-miR-74	hsa-miR-31	
		hsa-miR-513b	
		hsa-miR-873	
miR-75	cel-miR-75	hsa-miR-9*	
		hsa-miR-320	
		hsa-miR-548a-3p	
miR-79	cel-miR-79	hsa-miR-7	hsa-miR-9*
		hsa-miR-9*	
		hsa-miR-320	
		hsa-miR-340	
		hsa-miR-548a-3p	
miR-80	cel-miR-80	hsa-miR-450b-3p	
miR-81	cel-miR-81	hsa-miR-450b-3p	
miR-82	cel-miR-82	hsa-miR-450b-3p	
miR-83	cel-miR-83	hsa-miR-21*	hsa-miR-29a
		hsa-miR-29a	hsa-miR-29b
		hsa-miR-29b	hsa-miR-29c
		hsa-miR-29c	
		hsa-miR-593*	
miR-84	cel-miR-84	hsa-let-7a	hsa-let-7a
		hsa-let-7b	hsa-let-7b
		hsa-let-7c	hsa-let-7c
		hsa-let-7d	hsa-let-7e
		hsa-let-7e	hsa-let-7f
		hsa-let-7f	hsa-miR-98
		hsa-let-7g	
		hsa-let-7i	
		hsa-miR-98	
		hsa-miR-196a	
		hsa-miR-196b	
miR-86	cel-miR-86	hsa-miR-545*	
		hsa-miR-559	
miR-90	cel-miR-90	hsa-miR-190	
		hsa-miR-190b	
miR-124	cel-miR-124	hsa-miR-124	hsa-miR-124
		hsa-miR-506	
miR-228	cel-miR-228	hsa-miR-96	hsa-miR-183
		hsa-miR-183	
		hsa-miR-200a	
		hsa-miR-514	
miR-229	cel-miR-229	hsa-miR-96	
		hsa-miR-183	
		hsa-miR-200a	
		hsa-miR-514	
miR-231	cel-miR-231	hsa-miR-99a*	
		hsa-miR-99b*	
		hsa-miR-556-5p	
miR-232	cel-miR-232	hsa-miR-302a	
		hsa-miR-302b	
		hsa-miR-302c	
		hsa-miR-302d	
		hsa-miR-519a	
		hsa-miR-519b-3p	
		hsa-miR-519c-3p	
miR-234	cel-miR-234	hsa-miR-126*	hsa-miR-137
		hsa-miR-137	
miR-235	cel-miR-235	hsa-miR-25	hsa-miR-25
		hsa-miR-32	hsa-miR-92a
		hsa-miR-92a	hsa-miR-92b
		hsa-miR-92b	
		hsa-miR-363	
		hsa-miR-367	
		hsa-miR-885-5p	
miR-236	cel-miR-236	hsa-miR-200b	hsa-miR-141
		hsa-miR-200c	hsa-miR-200a
		hsa-miR-429	hsa-miR-200b
			hsa-miR-200c
			hsa-miR-429
miR-237	cel-miR-237	hsa-miR-125a-5p	
		hsa-miR-125b	
		hsa-miR-331-3p	
miR-240	cel-miR-240	hsa-miR-193a-3p	hsa-miR-193b
		hsa-miR-193b	
miR-241	cel-miR-241	hsa-let-7a	
		hsa-let-7b	
		hsa-let-7c	
		hsa-let-7d	
		hsa-let-7e	
		hsa-let-7f	
		hsa-let-7g	
		hsa-let-7i	
		hsa-miR-98	
miR-244	cel-miR-244	hsa-miR-9	
miR-245	cel-miR-245	hsa-miR-133a	hsa-miR-133a
		hsa-miR-133b	hsa-miR-133b
miR-247	cel-miR-247	hsa-miR-134	
		hsa-miR-708*	
miR-250	cel-miR-250	hsa-miR-27a	
		hsa-miR-27b	
		hsa-miR-128	
		hsa-miR-499-3p	
		hsa-miR-768-3p	
miR-251	cel-miR-251	hsa-miR-26a	
		hsa-miR-26b	
miR-252	cel-miR-252	hsa-miR-26a	
		hsa-miR-26b	
miR-254	cel-miR-254	hsa-miR-19a	
		hsa-miR-19b	
miR-256	cel-miR-256	hsa-miR-1	hsa-miR-1
		hsa-miR-122	
		hsa-miR-206	
		hsa-miR-519a	
		hsa-miR-519b-3p	
		hsa-miR-519c-3p	
miR-259	cel-miR-259	hsa-miR-216a	
		hsa-miR-216b	
miR-266	cel-miR-266	hsa-miR-31	hsa-miR-25*
			hsa-miR-31
			hsa-miR-301a
			hsa-miR-301b
miR-267	cel-miR-267	hsa-miR-99a	
		hsa-miR-99b	
		hsa-miR-100	
miR-268	cel-miR-268	hsa-miR-31	
		hsa-miR-873	
miR-269	cel-miR-269	hsa-miR-31	hsa-miR-31
miR-273	cel-miR-273	hsa-miR-99a	
		hsa-miR-99b	
		hsa-miR-100	
miR-357	cel-miR-357	hsa-miR-302a	
		hsa-miR-302b	
		hsa-miR-302c	
		hsa-miR-302d	
miR-785	cel-miR-785	hsa-miR-545*	
		hsa-miR-559	
miR-786	cel-miR-786	hsa-miR-18a*	
		hsa-miR-18b*	
		hsa-miR-365	
miR-787	cel-miR-787	hsa-miR-99a*	
		hsa-miR-99b*	
		hsa-miR-556-5p	
miR-790	cel-miR-790	hsa-miR-96	
		hsa-miR-183	
		hsa-miR-200a	
		hsa-miR-514	
miR-791	cel-miR-791	hsa-miR-96	
		hsa-miR-182	
		hsa-miR-183	
		hsa-miR-200a	
		hsa-miR-514	
miR-793	cel-miR-793	hsa-let-7a	hsa-let-7g
		hsa-let-7b	
		hsa-let-7c	
		hsa-let-7e	
		hsa-let-7f	
		hsa-let-7g	
		hsa-let-7i	
		hsa-miR-98	
		hsa-miR-202	
miR-794	cel-miR-794	hsa-let-7a	
		hsa-let-7b	
		hsa-let-7c	
		hsa-let-7d	
		hsa-let-7e	
		hsa-let-7f	
		hsa-let-7g	
		hsa-let-7i	
		hsa-miR-98	
		hsa-miR-196a	
miR-795	cel-miR-795	hsa-let-7a	
		hsa-let-7b	
		hsa-let-7c	
		hsa-let-7d	
		hsa-let-7e	
		hsa-let-7f	
		hsa-let-7g	
		hsa-let-7i	
		hsa-miR-98	
miR-796	cel-miR-796	hsa-miR-1	
		hsa-miR-122	
		hsa-miR-206	
miR-797	cel-miR-797	hsa-miR-499-3p	
miR-1018	cel-miR-1018	hsa-miR-450b-3p	
miR-1020	cel-miR-1020	hsa-miR-148b*	
miR-1022	cel-miR-1022	hsa-miR-450b-3p	

76 *C. elegans* miRNAs are 5′ related to 98 human miRNAs (Dataset S10), and 22 nematode miRNAs are ≥70% identical over the full length of 46 human miRNAs (Dataset S11). Worm-human miRNAs with weaker identity (60–69.9%) over full sequence are detailed in Dataset S12. Group IDs correspond to *C. elegans* miRNAs with human related-sequences.

**Table 5 pone-0002818-t005:** Analysis of 5′ and ≥70% similarity groups identifies 83 *D. melanogaster*-human miRNA families including 83 fly miRNAs and 121 human miRNAs.

miRNA Group ID	*D.melanogaster* miRNA	Human Sequence-Related miRNAs	
		5′ Sequence	Full Sequence
bantam	dme-bantam	hsa-miR-450b-3p	
let-7	dme-let-7	hsa-let-7a	hsa-let-7a
		hsa-let-7b	hsa-let-7b
		hsa-let-7c	hsa-let-7c
		hsa-let-7d	hsa-let-7d
		hsa-let-7e	hsa-let-7e
		hsa-let-7f	hsa-let-7f
		hsa-let-7g	hsa-let-7g
		hsa-let-7i	hsa-let-7i
		hsa-miR-98	hsa-miR-98
		hsa-miR-196a	
		hsa-miR-196b	
miR-1	dme-miR-1	hsa-miR-1	hsa-miR-1
		hsa-miR-122	hsa-miR-206
		hsa-miR-206	
miR-2a	dme-miR-2a	hsa-miR-499-3p	
miR-2b	dme-miR-2b	hsa-miR-499-3p	
miR-2c	dme-miR-2c	hsa-miR-499-3p	
miR-3	dme-miR-3	hsa-miR-612	
miR-4	dme-miR-4	hsa-miR-9*	hsa-miR-9*
		hsa-miR-320	
		hsa-miR-548a-3p	
		hsa-miR-7	
		hsa-miR-340	
miR-6	dme-miR-6	hsa-miR-27a	
		hsa-miR-27b	
		hsa-miR-128	
		hsa-miR-499-3p	
		hsa-miR-768-3p	
miR-7	dme-miR-7	hsa-miR-7	hsa-miR-7
		hsa-miR-9*	
		hsa-miR-548a-3p	
		hsa-miR-146a	
		hsa-miR-146b-5p	
miR-8	dme-miR-8	hsa-miR-200b	hsa-miR-141
		hsa-miR-200c	hsa-miR-200a
		hsa-miR-429	hsa-miR-200b
			hsa-miR-200c
			hsa-miR-429
miR-9a	dme-miR-9a	hsa-miR-9	hsa-miR-9
miR-9b	dme-miR-9b	hsa-miR-9	hsa-miR-9
miR-9c	dme-miR-9c	hsa-miR-9	hsa-miR-9
miR-10	dme-miR-10	hsa-miR-10a	hsa-miR-10a
		hsa-miR-10b	hsa-miR-10b
		hsa-miR-146b-3p	hsa-miR-99a
			hsa-miR-100
miR-11	dme-miR-11	hsa-miR-27a	hsa-miR-27b
		hsa-miR-27b	
		hsa-miR-128	
		hsa-miR-499-3p	
		hsa-miR-768-3p	
miR-12	dme-miR-12	hsa-miR-496	
miR-13a	dme-miR-13a	hsa-miR-499-3p	
miR-13b	dme-miR-13b	hsa-miR-499-3p	
miR-14	dme-miR-14	hsa-miR-511	
miR-31a	dme-miR-31a	hsa-miR-31	hsa-miR-31
miR-31b	dme-miR-31b	hsa-miR-31	hsa-miR-31
miR-33	dme-miR-33	hsa-miR-18a	hsa-miR-33a
		hsa-miR-18b	hsa-miR-33b
		hsa-miR-33a	
		hsa-miR-33b	
		hsa-miR-221	
miR-34	dme-miR-34	hsa-miR-34a	hsa-miR-34a
		hsa-miR-34b*	hsa-miR-34b*
		hsa-miR-34c-5p	hsa-miR-34c-5p
		hsa-miR-449a	hsa-miR-449a
		hsa-miR-449b	
miR-79	dme-miR-79	hsa-miR-9*	hsa-miR-9*
		hsa-miR-320	
		hsa-miR-548a-3p	
		hsa-miR-7	
miR-92a	dme-miR-92a	hsa-miR-25	hsa-miR-25
		hsa-miR-32	hsa-miR-92a
		hsa-miR-92a	hsa-miR-92b
		hsa-miR-92b	
		hsa-miR-363	
		hsa-miR-367	
		hsa-miR-885-5p	
miR-92b	dme-miR-92b	hsa-miR-25	hsa-miR-25
		hsa-miR-32	hsa-miR-92a
		hsa-miR-92a	hsa-miR-92b
		hsa-miR-92b	
		hsa-miR-363	
		hsa-miR-367	
		hsa-miR-885-5p	
miR-100	dme-miR-100	hsa-miR-99a	hsa-miR-10a
		hsa-miR-99b	hsa-miR-10b
		hsa-miR-100	hsa-miR-99a
			hsa-miR-99b
			hsa-miR-100
miR-124	dme-miR-124	hsa-miR-124	hsa-miR-124
		hsa-miR-506	
miR-125	dme-miR-125	hsa-miR-125a-5p	hsa-miR-10a
		hsa-miR-125b	hsa-miR-10b
		hsa-miR-331-3p	hsa-miR-125a-5p
			hsa-miR-125b
miR-133	dme-miR-133	hsa-miR-133a	hsa-miR-133a
		hsa-miR-133b	hsa-miR-133b
miR-137	dme-miR-137	hsa-miR-137	hsa-miR-137
miR-184	dme-miR-184	hsa-miR-184	hsa-miR-184
miR-190	dme-miR-190	hsa-miR-190	hsa-miR-190
		hsa-miR-190b	hsa-miR-190b
miR-193	dme-miR-193	hsa-miR-193a-3p	hsa-miR-193a-3p
		hsa-miR-193b	
miR-210	dme-miR-210	hsa-miR-210	hsa-miR-210
miR-219	dme-miR-219	hsa-miR-219-5p	hsa-miR-219-5p
miR-263a	dme-miR-263a	hsa-miR-569	hsa-miR-183
miR-263b	dme-miR-263b	hsa-miR-96	hsa-miR-182
		hsa-miR-183	hsa-miR-183
		hsa-miR-514	
		hsa-miR-200a	
miR-274	dme-miR-274	hsa-miR-758	
miR-276a	dme-miR-276a	hsa-miR-28-5p	
miR-276b	dme-miR-276b	hsa-miR-28-5p	
miR-277	dme-miR-277	hsa-miR-148a	
		hsa-miR-302a	
		hsa-miR-302b	
		hsa-miR-302c	
		hsa-miR-302d	
		hsa-miR-519a	
		hsa-miR-519b-3p	
		hsa-miR-519c-3p	
miR-279	dme-miR-279	hsa-miR-28-3p	
		hsa-miR-134	
miR-281-1*	dme-miR-281-1*	hsa-miR-9*	
		hsa-miR-320	
		hsa-miR-548a-3p	
		hsa-miR-146a	
		hsa-miR-146b-5p	
miR-281-2*	dme-miR-281-2*	hsa-miR-146a	
		hsa-miR-146b-5p	
		hsa-miR-9*	
		hsa-miR-320	
miR-283	dme-miR-283	hsa-miR-496	
miR-285	dme-miR-285	hsa-miR-21*	hsa-miR-29a
		hsa-miR-29a	hsa-miR-29b
		hsa-miR-29b	hsa-miR-29c
		hsa-miR-29c	
		hsa-miR-593*	
miR-286	dme-miR-286	hsa-miR-134	
		hsa-miR-708*	
miR-304	dme-miR-304	hsa-miR-216a	hsa-miR-216a
miR-306	dme-miR-306		hsa-miR-873
miR-306*	dme-miR-306*	hsa-miR-450b-3p	
miR-308	dme-miR-308	hsa-miR-499-3p	
miR-310	dme-miR-310	hsa-miR-25	hsa-miR-92a
		hsa-miR-32	hsa-miR-92b
		hsa-miR-92a	
		hsa-miR-92b	
		hsa-miR-363	
		hsa-miR-367	
		hsa-miR-885-5p	
miR-311	dme-miR-311	hsa-miR-25	hsa-miR-92a
		hsa-miR-32	
		hsa-miR-92a	
		hsa-miR-92b	
		hsa-miR-363	
		hsa-miR-367	
		hsa-miR-885-5p	
miR-312	dme-miR-312	hsa-miR-25	hsa-miR-25
		hsa-miR-32	hsa-miR-92a
		hsa-miR-92a	hsa-miR-92b
		hsa-miR-92b	
		hsa-miR-363	
		hsa-miR-367	
		hsa-miR-885-5p	
miR-313	dme-miR-313	hsa-miR-92a	hsa-miR-25
		hsa-miR-92b	hsa-miR-92a
		hsa-miR-25	
		hsa-miR-32	
		hsa-miR-363	
		hsa-miR-367	
		hsa-miR-885-5p	
miR-314	dme-miR-314	hsa-miR-498	
miR-316	dme-miR-316	hsa-miR-511	
miR-318	dme-miR-318	hsa-miR-612	
miR-375	dme-miR-375	hsa-miR-375	hsa-miR-375
miR-957	dme-miR-957	hsa-miR-451	
miR-960	dme-miR-960	hsa-miR-496	
miR-961	dme-miR-961	hsa-miR-133a	
		hsa-miR-133b	
miR-963	dme-miR-963	hsa-let-7a	
		hsa-let-7b	
		hsa-let-7c	
		hsa-let-7d	
		hsa-let-7e	
		hsa-let-7f	
		hsa-let-7g	
		hsa-let-7i	
		hsa-miR-98	
miR-964	dme-miR-964	hsa-miR-651	
miR-967	dme-miR-967	hsa-miR-620	
miR-977	dme-miR-977	hsa-let-7a	
		hsa-let-7b	
		hsa-let-7c	
		hsa-let-7e	
		hsa-let-7f	
		hsa-let-7g	
		hsa-let-7i	
		hsa-miR-98	
		hsa-miR-202	
miR-980	dme-miR-980	hsa-miR-22	
miR-983	dme-miR-983	hsa-miR-655	
miR-984	dme-miR-984	hsa-let-7a	hsa-let-7a
		hsa-let-7b	hsa-let-7d
		hsa-let-7c	hsa-let-7f
		hsa-let-7d	hsa-let-7g
		hsa-let-7e	
		hsa-let-7f	
		hsa-let-7g	
		hsa-let-7i	
		hsa-miR-98	
miR-986	dme-miR-986	hsa-miR-513c	
miR-987	dme-miR-987	hsa-miR-545*	
		hsa-miR-559	
miR-990	dme-miR-990	hsa-miR-197	
miR-993	dme-miR-993	hsa-miR-99a*	hsa-miR-100*
		hsa-miR-99b*	
		hsa-miR-556-5p	
miR-995	dme-miR-995	hsa-miR-21*	hsa-miR-29a
		hsa-miR-29a	hsa-miR-29c
		hsa-miR-29b	
		hsa-miR-29c	
		hsa-miR-593*	
miR-996	dme-miR-996	hsa-miR-28-3p	
		hsa-miR-134	
		hsa-miR-708*	
miR-998	dme-miR-998	hsa-miR-21*	
		hsa-miR-29a	
		hsa-miR-29b	
		hsa-miR-29c	
		hsa-miR-593*	
miR-1001	dme-miR-1001	hsa-miR-555	
miR-1002	dme-miR-1002	hsa-miR-26a	
		hsa-miR-26b	
miR-1003	dme-miR-1003	hsa-miR-342-3p	
miR-1010	dme-miR-1010	hsa-miR-412	
miR-1016	dme-miR-1016	hsa-miR-412	

82 *D. melanogaster* miRNAs have homology at the 5′ end with 117 human miRNAs (Dataset S13), and 40 *D. melanogaster* miRNAs are ≥70% identical with 56 human miRNAs (Dataset S14). Please refer to Dataset S15 for fly-human miRNAs with 60–69.9% overall similarity. Group IDs correspond to *D. melanogaster* miRNAs with human related sequences.

**Table 6:** *C. elegans* miRNAs conserved in *D. melanogaster* and *H. sapiens*.

miRNA probe	Conserved miRNA Sequences		
	*C. elegans*	*D. melanogaster*	*H. sapiens*
cel-let-7	cel-miR-48 ˆ	dme-let-7 ˆ ^70^	hsa-let-7a ˆ ^70^
	cel-miR-84 ˆ ^70^	dme-miR-963 ˆ	hsa-let-7b ˆ ^70^
	cel-miR-241 ˆ	dme-miR-977 ˆ	hsa-let-7c ˆ ^70^
	cel-miR-793 ˆ	dme-miR-984 ˆ ^70^	hsa-let-7d ˆ ^70^
	cel-miR-794 ˆ		hsa-let-7e ˆ ^70^
	cel-miR-795 ˆ		hsa-let-7f ˆ ^70^
			hsa-let-7g ˆ ^70^
			hsa-let-7i ˆ ^70^
			hsa-miR-98 ˆ ^70^
			hsa-miR-196a ˆ
			hsa-miR-196b ˆ
cel-lin-4	cel-miR-237 ˆ	dme-miR-125 ˆ ^70^	hsa-miR-125a-5p ˆ ^70^
			hsa-miR-125b ˆ ^70^
			hsa-miR-331-3p ˆ
cel-miR-1	cel-miR-256 ˆ ^70^	dme-miR-1 ˆ ^70^	hsa-miR-1 ˆ ^70^
	cel-miR-796 ˆ		hsa-miR-122 ˆ
			hsa-miR-206 ˆ ^70^
cel-miR-2	cel-miR-43 ˆ ^70^	dme-miR-2a ˆ ^70^	hsa-miR-499-3p ˆ
	cel-miR-250 ˆ	dme-miR-2b ˆ ^70^	
	cel-miR-797 ˆ	dme-miR-2c ˆ ^70^	
		dme-miR-6 ˆ	
		dme-miR-11 ˆ	
		dme-miR-13a ˆ ^70^	
		dme-miR-13b ˆ ^70^	
		dme-miR-308 ˆ	
cel-miR-34		dme-miR-34 ˆ ^70^	hsa-miR-34a ˆ ^70^
			hsa-miR-34b*( ˆ ^70^)
			hsa-miR-34c-5p ˆ ^70^
			hsa-miR-449a ˆ ^70^
			hsa-miR-449b ˆ ^70^
cel-miR-43	cel-miR-2 ˆ ^70^	dme-miR-2a ˆ	hsa-miR-27a ˆ
	cel-miR-250 ˆ	dme-miR-2b ˆ	hsa-miR-27b ˆ
	cel-miR-797 ˆ	dme-miR-2c ˆ	hsa-miR-128 ˆ
		dme-miR-6 ˆ	hsa-miR-499-3p ˆ
		dme-miR-11 ˆ	hsa-miR-768-3p ˆ
		dme-miR-13a ˆ	
		dme-miR-13b ˆ	
		dme-miR-308 ˆ	
cel-miR-44	cel-miR-45 ˆ ^70^	dme-miR-279 ˆ	hsa-miR-134 ˆ
	cel-miR-61 ˆ	dme-miR-286 ˆ	hsa-miR-708*( ˆ)
	cel-miR-247 ˆ	dme-miR-996 ˆ	
cel-miR-45	cel-miR-44 ˆ ^70^	dme-miR-279 ˆ	hsa-miR-134 ˆ
	cel-miR-61 ˆ	dme-miR-286 ˆ	hsa-miR-708*( ˆ)
	cel-miR-247 ˆ	dme-miR-996 ˆ	
cel-miR-48	cel-let-7 ˆ	dme-let-7 ˆ	hsa-let-7a ˆ
	cel-miR-84 ˆ	dme-miR-963 ˆ	hsa-let-7b ˆ
	cel-miR-241 ˆ	dme-miR-977 ˆ	hsa-let-7c ˆ
	cel-miR-793 ˆ	dme-miR-984 ˆ	hsa-let-7d ˆ
	cel-miR-794 ˆ		hsa-let-7e ˆ
	cel-miR-795 ˆ		hsa-let-7f ˆ
			hsa-let-7g ˆ
			hsa-let-7i ˆ
			hsa-miR-98 ˆ
cel-miR-49	cel-miR-83 ˆ	dme-miR-285 ˆ	hsa-miR-21*( ˆ)
		dme-miR-995 ˆ	hsa-miR-29a ˆ
		dme-miR-998 ˆ	hsa-miR-29b ˆ
			hsa-miR-29c ˆ
			hsa-miR-593*( ˆ)
cel-miR-50	cel-miR-62 ˆ	dme-miR-190 ˆ ^70^	hsa-miR-190 ˆ ^70^
	cel-miR-90 ˆ		hsa-miR-190b ˆ ^70^
cel-miR-51	cel-miR-52 ˆ	dme-miR-100 ˆ	hsa-miR-99a ˆ ^70^
	cel-miR-53 ˆ		hsa-miR-99b ˆ
	cel-miR-54 ˆ		hsa-miR-100 ˆ
	cel-miR-55 ˆ		
	cel-miR-56 ˆ		
	cel-miR-267 ˆ		
	cel-miR-273 ˆ		
cel-miR-52	cel-miR-51 ˆ	dme-miR-100 ˆ	hsa-miR-99a ˆ
	cel-miR-53 ˆ ^70^		hsa-miR-99b ˆ
	cel-miR-54 ˆ		hsa-miR-100 ˆ
	cel-miR-55 ˆ		
	cel-miR-56 ˆ ^70^		
	cel-miR-273 ˆ		
cel-miR-53	cel-miR-51 ˆ	dme-miR-100 ˆ	hsa-miR-99a ˆ
	cel-miR-52 ˆ ^70^		hsa-miR-99b ˆ
	cel-miR-54 ˆ		hsa-miR-100 ˆ
	cel-miR-55 ˆ		
	cel-miR-56 ˆ		
	cel-miR-273 ˆ		
cel-miR-54	cel-miR-51 ˆ	dme-miR-100 ˆ	hsa-miR-99a ˆ
	cel-miR-52 ˆ		hsa-miR-99b ˆ
	cel-miR-53 ˆ		hsa-miR-100 ˆ
	cel-miR-55 ˆ		
	cel-miR-56 ˆ ^70^		
	cel-miR-267 ˆ		
	cel-miR-273 ˆ		
	cel-miR-360 ˆ		
cel-miR-55	cel-miR-51 ˆ	dme-miR-100 ˆ	hsa-miR-99a ˆ
	cel-miR-52 ˆ		hsa-miR-99b ˆ
	cel-miR-53 ˆ		hsa-miR-100 ˆ
	cel-miR-54 ˆ		
	cel-miR-56 ˆ ^70^		
	cel-miR-273 ˆ		
cel-miR-56	cel-miR-51 ˆ	dme-miR-100 ˆ	hsa-miR-99a ˆ
	cel-miR-52 ˆ ^70^		hsa-miR-99b ˆ
	cel-miR-53 ˆ		hsa-miR-100 ˆ
	cel-miR-54 ˆ ^70^		
	cel-miR-55 ˆ ^70^		
	cel-miR-267 ˆ		
	cel-miR-273 ˆ ^70^		
	cel-miR-360 ˆ		
cel-miR-57		dme-miR-10 ˆ	hsa-miR-10a ˆ ^70^
			hsa-miR-10b ˆ ^70^
			hsa-miR-99a^70^
			hsa-miR-100^70^
			hsa-miR-146b-3p ˆ
cel-miR-58	cel-miR-80 ˆ	dme-bantam ˆ	hsa-miR-450b-3p ˆ
	cel-miR-81 ˆ	dme-miR-306*( ˆ)	
	cel-miR-82 ˆ		
	cel-miR-1018 ˆ		
	cel-miR-1022 ˆ		
cel-miR-61	cel-miR-44 ˆ	dme-miR-279 ˆ	hsa-miR-134 ˆ
	cel-miR-45 ˆ	dme-miR-286 ˆ	hsa-miR-708*( ˆ)
	cel-miR-247 ˆ ^70^	dme-miR-996 ˆ	
cel-miR-62	cel-miR-50 ˆ	dme-miR-190 ˆ	hsa-miR-190 ˆ
	cel-miR-90 ˆ		hsa-miR-190b ˆ
cel-miR-63	cel-miR-64 ˆ ^70^	dme-miR-263b ˆ	hsa-miR-96 ˆ
	cel-miR-65 ˆ ^70^		hsa-miR-183 ˆ
	cel-miR-66 ˆ		hsa-miR-200a ˆ
	cel-miR-228 ˆ		hsa-miR-514 ˆ
	cel-miR-229 ˆ		
	cel-miR-790 ˆ		
	cel-miR-791 ˆ		
cel-miR-64	cel-miR-63 ˆ ^70^	dme-miR-263b ˆ	hsa-miR-96 ˆ
	cel-miR-65 ˆ ^70^		hsa-miR-183 ˆ
	cel-miR-66 ˆ		hsa-miR-200a ˆ
	cel-miR-228 ˆ		hsa-miR-514 ˆ
	cel-miR-229 ˆ		
	cel-miR-790 ˆ		
	cel-miR-791 ˆ		
cel-miR-65	cel-miR-63 ˆ ^70^	dme-miR-263b ˆ	hsa-miR-96 ˆ
	cel-miR-64 ˆ ^70^		hsa-miR-183 ˆ
	cel-miR-66 ˆ		hsa-miR-200a ˆ
	cel-miR-228 ˆ		hsa-miR-514 ˆ
	cel-miR-229 ˆ		
	cel-miR-790 ˆ		
	cel-miR-791 ˆ		
cel-miR-66	cel-miR-63 ˆ	dme-miR-263b ˆ	hsa-miR-96 ˆ
	cel-miR-64 ˆ		hsa-miR-183 ˆ
	cel-miR-65 ˆ		hsa-miR-200a ˆ
	cel-miR-228 ˆ		hsa-miR-514 ˆ
	cel-miR-229 ˆ		
	cel-miR-790 ˆ		
	cel-miR-791 ˆ		
cel-miR-72	cel-miR-73 ˆ	dme-miR-31a ˆ ^70^	hsa-miR-31 ˆ ^70^
	cel-miR-74 ˆ	dme-miR-31b ˆ ^70^	
	cel-miR-266 ˆ ^70^		
	cel-miR-268 ˆ		
	cel-miR-269 ˆ		
cel-miR-73	cel-miR-72 ˆ	dme-miR-31a ˆ ^70^	hsa-miR-31 ˆ
	cel-miR-74 ˆ	dme-miR-31b ˆ	
	cel-miR-266 ˆ		
	cel-miR-268 ˆ ^70^		
	cel-miR-269 ˆ		
	cel-miR-270^70^		
cel-miR-74	cel-miR-72 ˆ	dme-miR-31a ˆ	hsa-miR-31 ˆ
	cel-miR-73 ˆ	dme-miR-31b ˆ	hsa-miR-513b ˆ
	cel-miR-266 ˆ		hsa-miR-873 ˆ
	cel-miR-268 ˆ		
	cel-miR-269 ˆ		
cel-miR-75	cel-miR-79 ˆ	dme-miR-4 ˆ	hsa-miR-9*( ˆ)
		dme-miR-79 ˆ	hsa-miR-320 ˆ
		dme-miR-281-1*( ˆ)	hsa-miR-548a-3p ˆ
		dme-miR-281-2*( ˆ)	
cel-miR-79	cel-miR-75 ˆ	dme-miR-4 ˆ	hsa-miR-7 ˆ
		dme-miR-7 ˆ	hsa-miR-9*( ˆ ^70^)
		dme-miR-79 ˆ ^70^	hsa-miR-320 ˆ
		dme-miR-281-1*( ˆ)	hsa-miR-340 ˆ
		dme-miR-281-2*( ˆ)	hsa-miR-548a-3p ˆ
cel-miR-80	cel-miR-58 ˆ	dme-bantam ˆ ^70^	hsa-miR-450b-3p ˆ
	cel-miR-81 ˆ	dme-miR-306*( ˆ)	
	cel-miR-82 ˆ ^70^		
	cel-miR-1018 ˆ		
	cel-miR-1022 ˆ		
cel-miR-81	cel-miR-58 ˆ	dme-bantam ˆ ^70^	hsa-miR-450b-3p ˆ
	cel-miR-80 ˆ	dme-miR-306*( ˆ)	
	cel-miR-82 ˆ ^70^		
	cel-miR-1018 ˆ		
	cel-miR-1022 ˆ		
cel-miR-82	cel-miR-58 ˆ	dme-bantam ˆ ^70^	hsa-miR-450b-3p ˆ
	cel-miR-80 ˆ ^70^	dme-miR-306*( ˆ)	
	cel-miR-81 ˆ ^70^		
	cel-miR-1018 ˆ		
	cel-miR-1022 ˆ		
cel-miR-83	cel-miR-49 ˆ	dme-miR-285 ˆ ^70^	hsa-miR-21*( ˆ)
		dme-miR-995 ˆ	hsa-miR-29a ˆ ^70^
		dme-miR-998 ˆ ^70^	hsa-miR-29b ˆ ^70^
			hsa-miR-29c ˆ ^70^
			hsa-miR-593*( ˆ)
cel-miR-84	cel-let-7 ˆ ^70^	dme-let-7 ˆ ^70^	hsa-let-7a ˆ ^70^
	cel-miR-48 ˆ	dme-miR-963 ˆ	hsa-let-7b ˆ ^70^
	cel-miR-241 ˆ	dme-miR-977 ˆ	hsa-let-7c ˆ ^70^
	cel-miR-793 ˆ	dme-miR-984 ˆ	hsa-let-7d ˆ
	cel-miR-794 ˆ		hsa-let-7e ˆ ^70^
	cel-miR-795 ˆ		hsa-let-7f ˆ ^70^
			hsa-let-7g ˆ
			hsa-let-7i ˆ
			hsa-miR-98 ˆ ^70^
			hsa-miR-196a ˆ
			hsa-miR-196b ˆ
cel-miR-86	cel-miR-785 ˆ	dme-miR-987 ˆ	hsa-miR-545*( ˆ)
			hsa-miR-559 ˆ
cel-miR-90	cel-miR-50 ˆ	dme-miR-190 ˆ	hsa-miR-190 ˆ
	cel-miR-62 ˆ		hsa-miR-190b ˆ
cel-miR-124		dme-miR-124 ˆ ^70^	hsa-miR-124 ˆ ^70^
			hsa-miR-506 ˆ
cel-miR-228	cel-miR-63 ˆ	dme-miR-263a ˆ ^70^	hsa-miR-96 ˆ
	cel-miR-64 ˆ	dme-miR-263b ˆ	hsa-miR-183 ˆ ^70^
	cel-miR-65 ˆ		hsa-miR-200a ˆ
	cel-miR-66 ˆ		hsa-miR-514 ˆ
	cel-miR-229 ˆ		
	cel-miR-790 ˆ		
	cel-miR-791 ˆ		
cel-miR-229	cel-miR-63 ˆ	dme-miR-263a ˆ	hsa-miR-96 ˆ
	cel-miR-64 ˆ	dme-miR-263b ˆ	hsa-miR-183 ˆ
	cel-miR-65 ˆ		hsa-miR-200a ˆ
	cel-miR-66 ˆ		hsa-miR-514 ˆ
	cel-miR-228 ˆ		
	cel-miR-790 ˆ		
	cel-miR-791 ˆ		
cel-miR-231	cel-miR-787 ˆ	dme-miR-993 ˆ	hsa-miR-99a*( ˆ)
			hsa-miR-99b*( ˆ)
			hsa-miR-556-5p ˆ
cel-miR-232	cel-miR-256 ˆ	dme-miR-277 ˆ	hsa-miR-302a ˆ
	cel-miR-357 ˆ		hsa-miR-302b ˆ
			hsa-miR-302c ˆ
			hsa-miR-302d ˆ
			hsa-miR-519a ˆ
			hsa-miR-519b-3p ˆ
			hsa-miR-519c-3p ˆ
cel-miR-234		dme-miR-137 ˆ ^70^	hsa-miR-126*( ˆ)
			hsa-miR-137 ˆ ^70^
cel-miR-235		dme-miR-92a ˆ ^70^	hsa-miR-25 ˆ ^70^
		dme-miR-92b ˆ ^70^	hsa-miR-32 ˆ
		dme-miR-310 ˆ ^70^	hsa-miR-92a ˆ ^70^
		dme-miR-311 ˆ ^70^	hsa-miR-92b ˆ ^70^
		dme-miR-312 ˆ ^70^	hsa-miR-363 ˆ
		dme-miR-313 ˆ ^70^	hsa-miR-367 ˆ
			hsa-miR-885-5p ˆ
cel-miR-236		dme-miR-8 ˆ ^70^	hsa-miR-141^70^
			hsa-miR-200a^70^
			hsa-miR-200b ˆ ^70^
			hsa-miR-200c ˆ ^70^
			hsa-miR-429 ˆ ^70^
cel-miR-237	cel-lin-4 ˆ	dme-miR-125 ˆ	hsa-miR-125a-5p ˆ
			hsa-miR-125b ˆ
			hsa-miR-331-3p ˆ
cel-miR-240		dme-miR-193 ˆ	hsa-miR-193a-3p ˆ
			hsa-miR-193b ˆ ^70^
cel-miR-241	cel-let-7 ˆ	dme-let-7 ˆ	hsa-let-7a ˆ
	cel-miR-48 ˆ	dme-miR-963 ˆ	hsa-let-7b ˆ
	cel-miR-84 ˆ	dme-miR-977 ˆ	hsa-let-7c ˆ
	cel-miR-793 ˆ	dme-miR-984 ˆ	hsa-let-7d ˆ
	cel-miR-794 ˆ		hsa-let-7e ˆ
	cel-miR-795 ˆ		hsa-let-7f ˆ
			hsa-let-7g ˆ
			hsa-let-7i ˆ
			hsa-miR-98 ˆ
cel-miR-244		dme-miR-9a ˆ	hsa-miR-9 ˆ
		dme-miR-9b ˆ	
		dme-miR-9c ˆ	
cel-miR-245		dme-miR-133 ˆ ^70^	hsa-miR-133a ˆ ^70^
			hsa-miR-133b ˆ ^70^
cel-miR-247	cel-miR-44 ˆ	dme-miR-279 ˆ	hsa-miR-134 ˆ
	cel-miR-45 ˆ	dme-miR-286 ˆ	hsa-miR-708*( ˆ)
	cel-miR-61 ˆ ^70^	dme-miR-996 ˆ ^70^	
cel-miR-250	cel-miR-2 ˆ	dme-miR-2a ˆ	hsa-miR-27a ˆ
	cel-miR-43 ˆ	dme-miR-2b ˆ	hsa-miR-27b ˆ
	cel-miR-797 ˆ	dme-miR-2c ˆ	hsa-miR-128 ˆ
		dme-miR-6 ˆ	hsa-miR-499-3p ˆ
		dme-miR-11 ˆ	hsa-miR-768-3p ˆ
		dme-miR-13a ˆ	
		dme-miR-13b ˆ	
		dme-miR-308 ˆ	
		dme-miR-1007^70^	
cel-miR-251	cel-miR-252 ˆ ^70^	dme-miR-1002 ˆ	hsa-miR-26a ˆ
			hsa-miR-26b ˆ
cel-miR-252	cel-miR-251 ˆ ^70^	dme-miR-1002 ˆ	hsa-miR-26a ˆ
		dme-miR-252^70^	hsa-miR-26b ˆ
cel-miR-256	cel-miR-1 ˆ ^70^	dme-miR-1 ˆ ^70^	hsa-miR-1 ˆ ^70^
	cel-miR-232 ˆ	dme-miR-277 ˆ	hsa-miR-122 ˆ
	cel-miR-796 ˆ		hsa-miR-206 ˆ
			hsa-miR-519a ˆ
			hsa-miR-519b-3p ˆ
			hsa-miR-519c-3p ˆ
cel-miR-259		dme-miR-304 ˆ	hsa-miR-216a ˆ
			hsa-miR-216b ˆ
cel-miR-266	cel-miR-72 ˆ ^70^	dme-miR-31a ˆ	hsa-miR-25*(^70^)
	cel-miR-73 ˆ	dme-miR-31b ˆ	hsa-miR-31 ˆ ^70^
	cel-miR-74 ˆ		hsa-miR-301a^70^
	cel-miR-268 ˆ		hsa-miR-301b^70^
	cel-miR-269 ˆ ^70^		
cel-miR-267	cel-miR-51 ˆ	dme-miR-100 ˆ	hsa-miR-99a ˆ
	cel-miR-54 ˆ		hsa-miR-99b ˆ
	cel-miR-56 ˆ		hsa-miR-100 ˆ
cel-miR-268	cel-miR-72 ˆ	dme-miR-31a ˆ	hsa-miR-31 ˆ
	cel-miR-73 ˆ ^70^	dme-miR-31b ˆ	hsa-miR-873 ˆ
	cel-miR-74 ˆ		
	cel-miR-266 ˆ		
	cel-miR-269 ˆ		
cel-miR-269	cel-miR-72 ˆ	dme-miR-31a ˆ	hsa-miR-31 ˆ ^70^
	cel-miR-73 ˆ	dme-miR-31b ˆ	
	cel-miR-74 ˆ		
	cel-miR-266 ˆ ^70^		
	cel-miR-268 ˆ		
cel-miR-273	cel-miR-51 ˆ	dme-miR-100 ˆ	hsa-miR-99a ˆ
	cel-miR-52 ˆ		hsa-miR-99b ˆ
	cel-miR-53 ˆ		hsa-miR-100 ˆ
	cel-miR-54 ˆ		
	cel-miR-55 ˆ		
	cel-miR-56 ˆ ^70^		
cel-miR-357	cel-miR-232 ˆ	dme-miR-277 ˆ	hsa-miR-302a ˆ
			hsa-miR-302b ˆ
			hsa-miR-302c ˆ
			hsa-miR-302d ˆ
cel-miR-785	cel-miR-86 ˆ	dme-miR-987 ˆ	hsa-miR-545*( ˆ)
			hsa-miR-559 ˆ
cel-miR-787	cel-miR-231 ˆ	dme-miR-993 ˆ	hsa-miR-99a*( ˆ)
			hsa-miR-99b*( ˆ)
			hsa-miR-556-5p ˆ
cel-miR-790	cel-miR-63 ˆ	dme-miR-263b ˆ	hsa-miR-96 ˆ
	cel-miR-64 ˆ		hsa-miR-183 ˆ
	cel-miR-65 ˆ		hsa-miR-200a ˆ
	cel-miR-66 ˆ		hsa-miR-514 ˆ
	cel-miR-228 ˆ		
	cel-miR-229 ˆ		
	cel-miR-791 ˆ		
cel-miR-791	cel-miR-63 ˆ	dme-miR-263b ˆ	hsa-miR-96 ˆ
	cel-miR-64 ˆ		hsa-miR-182 ˆ
	cel-miR-65 ˆ		hsa-miR-183 ˆ
	cel-miR-66 ˆ		hsa-miR-200a ˆ
	cel-miR-228 ˆ		hsa-miR-514 ˆ
	cel-miR-229 ˆ		
	cel-miR-790 ˆ		
cel-miR-793	cel-let-7 ˆ	dme-let-7 ˆ	hsa-let-7a ˆ
	cel-miR-48 ˆ	dme-miR-977 ˆ	hsa-let-7b ˆ
	cel-miR-84 ˆ	dme-miR-984 ˆ	hsa-let-7c ˆ
	cel-miR-241 ˆ		hsa-let-7e ˆ
	cel-miR-794 ˆ		hsa-let-7f ˆ
	cel-miR-795 ˆ		hsa-let-7g ˆ ^70^
			hsa-let-7i ˆ
			hsa-miR-98 ˆ
			hsa-miR-202 ˆ
cel-miR-794	cel-let-7 ˆ	dme-let-7 ˆ	hsa-let-7a ˆ
	cel-miR-48 ˆ	dme-miR-963 ˆ	hsa-let-7b ˆ
	cel-miR-84 ˆ	dme-miR-977 ˆ ^70^	hsa-let-7c ˆ
	cel-miR-241 ˆ	dme-miR-984 ˆ	hsa-let-7d ˆ
	cel-miR-793 ˆ		hsa-let-7e ˆ
	cel-miR-795 ˆ		hsa-let-7f ˆ
			hsa-let-7g ˆ
			hsa-let-7i ˆ
			hsa-miR-98 ˆ
			hsa-miR-196a ˆ
cel-miR-795	cel-let-7 ˆ	dme-let-7 ˆ	hsa-let-7a ˆ
	cel-miR-48 ˆ	dme-miR-963 ˆ	hsa-let-7b ˆ
	cel-miR-84 ˆ	dme-miR-977 ˆ	hsa-let-7c ˆ
	cel-miR-241 ˆ	dme-miR-984 ˆ	hsa-let-7d ˆ
	cel-miR-793 ˆ		hsa-let-7e ˆ
	cel-miR-794 ˆ		hsa-let-7f ˆ
			hsa-let-7g ˆ
			hsa-let-7i ˆ
			hsa-miR-98 ˆ
cel-miR-796	cel-miR-1 ˆ	dme-miR-1 ˆ	hsa-miR-1 ˆ
	cel-miR-256 ˆ		hsa-miR-122 ˆ
			hsa-miR-206 ˆ
cel-miR-797	cel-miR-2 ˆ	dme-miR-2a ˆ	hsa-miR-499-3p ˆ
	cel-miR-43 ˆ	dme-miR-2b ˆ	
	cel-miR-250 ˆ	dme-miR-2c ˆ	
		dme-miR-6 ˆ	
		dme-miR-11 ˆ	
		dme-miR-13a ˆ	
		dme-miR-13b ˆ	
		dme-miR-308 ˆ	
cel-miR-1018	cel-miR-58 ˆ	dme-bantam ˆ	hsa-miR-450b-3p ˆ
	cel-miR-80 ˆ		
	cel-miR-81 ˆ		
	cel-miR-82 ˆ		
	cel-miR-1022 ˆ		
cel-miR-1022	cel-miR-58 ˆ	dme-bantam ˆ	hsa-miR-450b-3p ˆ
	cel-miR-80 ˆ	dme-miR-306*( ˆ)	
	cel-miR-81 ˆ		
	cel-miR-82 ˆ		
	cel-miR-1018 ˆ		

73 *C. elegans* miRNAs have significant identity at their 5′ ends and/or ≥70% similarity over their entire sequences to both fly and human miRNAs. All the 73 *C. elegans* miRNAs have 5′ related sequences in both flies and humans, whereas 16/73 *C. elegans* miRNAs are also classified as ≥70% homologous over length to miRNAs in flies and humans. For detail in sequence relationships refer to Figure S1.

ˆ indicates miRNAs with 5′ end sequence homology present in worms, flies and humans. Superscript **70** denotes miRNAs with ≥70% similarity over full sequence across the three analyzed species.

It should be noted that the miRNA registry was extensively modified in the year 2007 (releases 10.0 and 10.1), introducing changes to previous mature miRNA sequences as well as adding new mature miRNA sequences to *C. elegans* (5), *D. melanogaster* (75) and human (494) miRNA databases. We performed our analysis using the latest miRBase release (10.1). We elected to use *C. elegans* sequences as reference anchors because of the general availability of deletions for *mir* genes.

### *C. elegans* miRNA families

#### *C. elegans* miRNA families defined by searches for homology in 5′ end sequences

We searched for 5′ end sequence alignments that included at least 7 nucleotides of continuous similarity within nt 1–10 of the mature miRNA, with no allowed gaps and only G..U mismatches permitted. By these criteria, we identified 81 *C. elegans* miRNAs that can be placed into 19 different families (Table 1, Dataset S1). We observed that 5′ homologies were mainly located from nucleotides 2 to 8, consistent with conserved sequence present in the seed region (Figure S2). Moreover, related miRNAs sharing longer nucleotide homologies at the 5′ end tend to be more similar at the 3′ end (and therefore over their full lengths) as compared to miRNAs with 5′ homologous regions of only 7 or 8 nucleotides.

#### *C. elegans* miRNA families defined by searches for homology over their lengths

We also compiled a list of miRNA families by requiring homology over the entire miRNA length. We grouped 45 of the 139 *C. elegans* mature miRNAs into 15 different families based on ≥70% identity over mature sequence length (Table 1, Dataset S2). Consistent with current reports in the field, the highest similarity occurs predominantly at the 5′ end in full-length sequence alignments.

#### Two homology search criteria generate a *C. elegans* miRNA family list with substantial, but not complete, overlap

Combining the two strategies for identification of homologies among miRNAs that we described above, we identify 84 *C. elegans* sequence-related miRNAs grouped in 20 families (Table 1). This analysis expands the previously reported number of members in *C. elegans* miRNA families [43], [51], [63], [64] and establishes 1 new sequence-related group containing miRNAs cel-miR-78 and cel-miR-272. About half (101/211) of the sequence relationships described in this work have not been posted in previous works and in the miRBase page listing of sequence relationships among miRNA precursors.

The two homology search approaches we used identify a substantially overlapping list, although clearly not all miRNAs fit both 5′ end and overall similarity criteria. Of the 139 *C. elegans* miRNAs analyzed, 77 miRNAs exhibit high identity at the 5′ end but <70% overall similarity with at least one of their 5′ sequence-related miRNAs (indicated in Dataset S1). 40 miRNAs have significant homology to sequence-related worm miRNAs only at the 5′ end and thus were not included in the ≥70% homology lists compiled after full length sequence comparison (Table 1, Dataset S2). Conversely, not all miRNAs with similarity over the sequence length include 7 or more continuous identical nucleotides within the first 10 nt of the 5′ end. 3 of the 45 miRNAs with ≥70% identity (cel-miR-78, cel-miR-270 and cel-miR-272) fail to comply with our criteria for 5′ end family grouping and therefore are not included in the list of 5′ end-related miRNAs in Dataset S1.

### 3′ end sequences

miRNA target sites with perfect complementarity to miRNA 3′ ends and negligible pairing at the 5′ end have not been described—extensive 3′ pairing has been suggested to act as a determinant of target specificity or regulatory sensitivity within miRNA families [41], but it is the 5′ end sequences that are thought to drive target selection and major regulation. Nonetheless, we were curious as to whether miRNAs could share significant sequence similarity at the 3′ end but negligible or weak 5′ similarity. We therefore probed relationships among 3′ end sequences of mature *C. elegans* miRNAs by multiple alignments of the 3′ sequence of each miRNA against 3′ sequences of all the remaining miRNAs. About half of *C. elegans* miRNAs are ≥60% similar to another at their 3′ end (67/139); one quarter of these are >70% identical. In general, however, the more nucleotide similarity at the 3′ end, the more identical the miRNAs are at the 5′ end.

It may be noteworthy that within the group of miRNAs with 50–70% 3′ similarity, we could identify some with extensive sequence identity at the 3′ end and low 5′ similarity (Figure S3). These groups are: 1) cel-lin-4, cel-miR-87; 2) cel-miR-90, cel-miR-124 (3′ region of identity also conserved to some extent in cel-miR-80, cel-miR-81, cel-miR-82 and cel-miR-234); 3) cel-miR-81, cel-miR-799 (3′ region of identity also conserved to some extent in cel-miR-80 and cel-miR-82); and 4) cel-miR-52, cel-mir-53, cel-miR-70, cel-miR-229 and cel-miR-272. Although no data are yet available to address the potential functions of these 3′-related miRNAs, their conservation suggests these 3′ motifs could be important for miRNA function. For example, a hexanucleotide 3′ terminal motif has recently been shown to direct hsa-miR-29b to the nucleus [77].

Searches of the *C. elegans* 3′ miRNA motifs in *Drosophila* and humans identified 3′ relationships of cel-miR-80 and cel-miR-799 with hsa-miR-208a, and interestingly revealed 3′ relationships of hsa-miR-208a with hsa-miR-129-3p and hsa-miR-129* and of hsa-miR-124 with hsa-miR-377* (Figure S3). Thus, 3′ homologous sequences might reveal functional similarities among miRNAs in nematodes, flies and humans.

Overall, although some 3′ end similarities can be distinguished among miRNAs (even for miRNAs placed into different families), our overview of 3′ end homologies among miRNAs strongly supports the current idea that 5′ end miRNA sequences are much more highly conserved than 3′ ends.

### Clustering of *mir* genes in *C. elegans* and *D. melanogaster* genomes

miRNAs can derive from their own transcription units or from exons or introns of other genes [78]. Consecutive *mir* genes with the same transcriptional orientation within relatively short distances can be considered as clustered. 42% of human *mir* genes appear in clusters of 2 or more within 3 Kb intervals [79].

**Figure 1:**
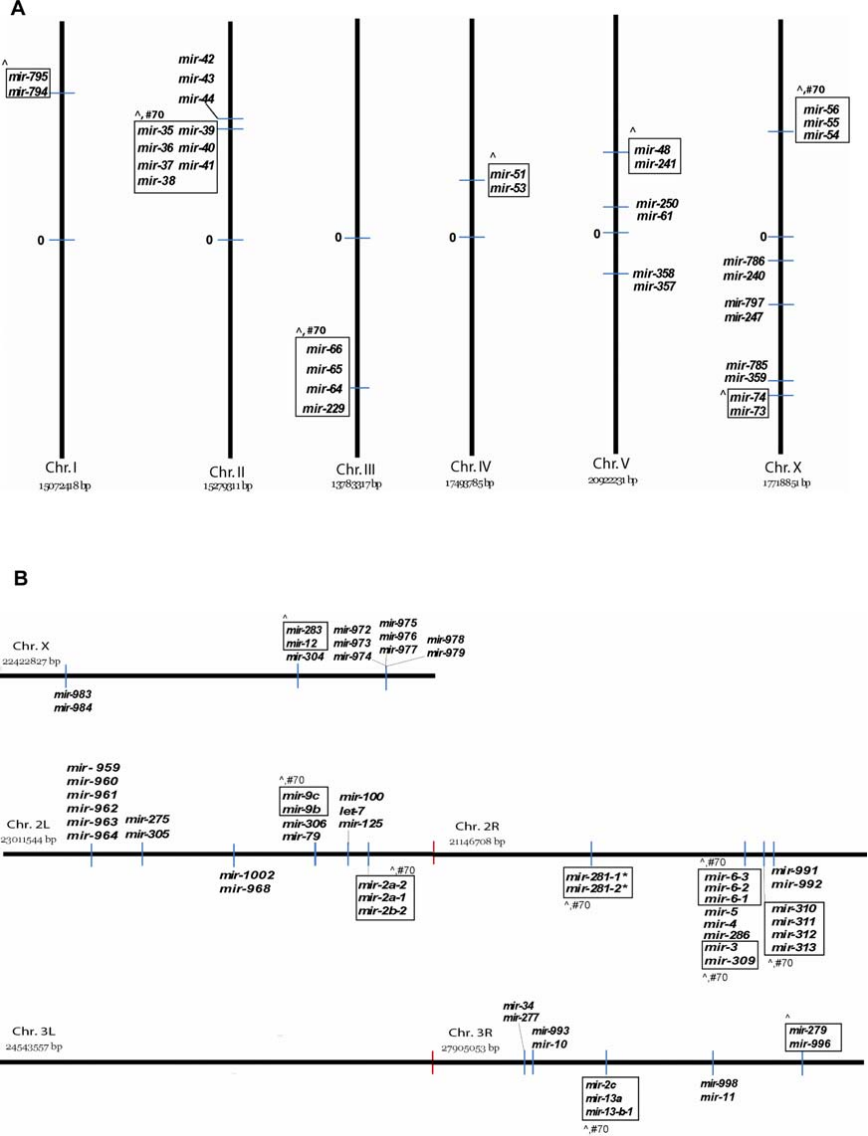
Clusters of *mir* genes in the *C. elegans* and *D. melanogaster* genomes. 35 of the 137 *C. elegans mir* genes (the 137 genes produce 139 miRNA forms) (A) and 60 of the 152 *D. melanogaster mir* genes (B) are situated within 2 Kb of each other on one of their chromosomes (6 chromosomes in *C. elegans*: Chr. I–V, Chr. X; 4 pairs of chromosomes in *D. melanogaster*: Chr. 2–4, X/Y). ∼63% clustered *mir* genes in the *C. elegans* genome and ∼38% in the *D. melanogaster* genome are related in sequence. Bounding boxes highlight clustered *mir* genes of conserved sequences at the 5′ end (ˆ) and/or over full length (#70 indicates ≥70% similarity) (see Tables 1, 2 and Datasets S1, S2, S4 and S5 for details). *mir* genes on the left or above chromosomes are found in the Watson strand whereas those on the right or below are located in the Crick strand. The physical centers of *C. elegans* chromosomes are indicated by “0”.

Some *C. elegans mir* gene clusters have been previously described: *mir-35-mir-41* (within a 796 bp region), *mir-42-mir-44* (307 bp), *mir-54-mir-56* (403 bp), *mir-229_mir-64-mir-66* (754 bp), *mir-73-mir-74* (374 bp), and *mir-241_mir-48* (∼1.7 Kb) [61], [63], [80]. Genes within these groups exhibit similar expression patterns, indicating that they might be co-transcribed into polycistronic units. Based on these observations, we chose a potential clustering range of 2 Kb to evaluate relative *mir* gene distribution in the *C. elegans* genome (Figure 1A). Interestingly, 35/137 *C. elegans mir* genes cluster into a total of 13 groups by this criterion. Most of the clusters contain 2 *mir* genes, with clustered *mir* genes more abundant on chromosomes II and X (the latter of which harbors a higher than average number of *mirs* overall (Figure S4A)). We checked whether clustered *mirs* are related in sequence and found that about half of the *mir* clusters contain *mir* genes that are homologous at the 5′ end and/or over full length (≥70%). If co-expressed, such genes might regulate common mRNAs by recognizing the same target sites. *mirs* in the remaining half of the clusters do not exhibit significant homology between them. If these *mirs* are co-expressed, they may target different mRNAs or might interact with the same target transcripts via multiple, distinct miRNA binding sites.

We also looked at the distribution of *mir* genes in the *D. melanogaster* genome (Figure 1B). Consistent with previous reports [52], [60], we determined that 60/152 *Drosophila mir* genes are clustered into 20 different regions 2 kb long. Clusters contain on average 3 *mir* genes with the longest cluster including 8 *mir* genes. Clustered *mir* genes are more abundant on chromosomes 2L and 2R, which also have a higher than average number of *mirs* overall (Figure S4B). ∼38% of the clustered *mir* genes in the *Drosophila* genome have 5′ and/or ≥70% full homologous sequences.

### *D. melanogaster* miRNA families

Similar to our strategy for *C. elegans* miRNA analysis, we screened *D. melanogaster* miRNAs for 7 consecutive identical nucleotides at the 5′ ends and classified 61 miRNAs into 19 families (Table 2, Dataset S4). Using the criteria of ≥70% overall identity, we highlight a total of 38 miRNAs that can be classified into 14 families (Table 2, Dataset S5). Overall, we identified 70 of the 152 *Drosophila* miRNAs as part of 24 sequence-related groups (Table 2).

As is the case for *C. elegans* miRNAs, lists of related *Drosophila* miRNAs compiled by the 5′ and ≥70% search criteria overlapped. However, 48 fly miRNAs are significantly similar at their 5′ end but have <70% overall identity with at least one of their sequence-related miRNAs (indicated in Dataset S4). Of these, 33 miRNAs have significant homology to other fly miRNAs only at their 5′ end and thus are not listed in the ≥70% homology groups (Table 2, Dataset S5). Most of the fly ≥70% full length homologs exhibit blocks of ≥7 nt identity at the 5′ end except the following 10: dme-miR-10, dme-miR-100, dme-miR-263a, dme-miR-263b, dme-miR-954, dme-miR-966, dme-miR-1009, dme-miR-1010, dme-miR-iab-4-3p and dme-miR-iab4as-3p.

### miRNAs conserved between *C. elegans* and *D. melanogaster*

We next compiled an expanded list of sequence-related miRNAs common to nematodes and flies. We searched for both 5′ end matches and for ≥70% homology over extended length between the 139 *C. elegans* and 152 *D. melanogaster* miRNAs using the criteria we described above for intra-species comparison. Overall, our sequence comparisons establish 64 novel worm/fly miRNA relationships [25], [43], [51], [52], [57], [60], [63]–[65], [75] and identify 87 miRNA families that now include 87 *C. elegans* and 65 *D. melanogaster* members (Table 3).

5′ end homology searches detected 87 worm miRNAs related to 62 fly miRNAs (Table 3, Dataset S7), whereas ≥70% overall identity searches highlighted 31 worm miRNAs and 37 fly miRNAs in family relationships (Table 3, Dataset S8). Of the 87 5′ related *C. elegans* miRNAs, 68 have a ≥7 nt block homology at the 5′ end but weak full length identity (<70%) with at least one of their 5′ fly miRNA relatives (indicated in Dataset S7). 59 of these have <70% full length sequence similarity with all their 5′ *Drosophila* relatives and thus these relationships are not present in our ≥70% homology lists in Dataset S8. 15 of the 87 *C. elegans* miRNAs with 5′ identities in flies have significant extended homology over their full length (≥70%) with all their *Drosophila* counterparts. Most of the *C. elegans_Drosophila* ≥70% miRNA homologs have ≥7 nt identity at the 5′ end except cel-miR-239a_dme-miR-12, cel-miR-252_dme-miR-252 and cel-miR-250_dme-miR-1007.

### miRNAs conserved between *C. elegans* and *H. sapiens*

We also searched for both 5′ end identities and for homologous (≥70%) extended sequence between *C. elegans* (139) and human (733) miRNAs using the criteria we described above. Overall, our sequence comparisons establish 141 novel nematode_human relationships [43], [51], [63], [64], [76] and identify 76 miRNA families that now include 76 *C. elegans* and 102 human members (Table 4). 76 worm miRNAs exhibit significant homologies to the 5′ ends of 98 human miRNAs (Table 4, Dataset S10), whereas 22 nematode miRNAs are ≥70% homologous over their full length to 46 human miRNAs (Table 4, Dataset S11). 69 of the 76 5′ related *C. elegans* miRNAs have <70% extended homology with at least one of their 5′ human counterparts (shown in Dataset S10), and 54 are weakly similar (<70%) with human miRNAs outside their 5′ end sequences. 7 of the above 76 *C. elegans* miRNAs have significant 5′ and overall (≥70%) homology with all their 5′ related sequences in humans. In our set of *C. elegans*_human ≥70% homologs, the following do not have ≥7 nucleotides of continuous similarity at the 5′ end: cel-miR-57 with hsa-miR-99a and hsa-miR-100; cel-miR-236 with hsa-miR-141 and hsa-miR-200a; and cel-miR-266 with hsa-miR-25*, hsa-miR-301a and hsa-miR-301b.

### miRNAs conserved between *D. melanogaster* and *H. sapiens*

Looking for 5′ end and ≥70% overall sequence similarities between *D. melanogaster* (152) and human (733) miRNAs, we detected 149 novel sequence relationships previous reported in [25], [43], [52], [57], [60], [65], [75], [76] expanding family groups to 83 defined by 83 *Drosophila* miRNAs and 121 human miRNAs (Table 5). Specifically, 82 *Drosophila* miRNAs show significant 5′ sequence identity to 117 human miRNAs (Table 5, Dataset S13), and 40 fly miRNAs are ≥70% homologous over full length to 56 human miRNAs (Table 5, Dataset S14). 67 of the above 82 *Drosophila* miRNAs are <70% identical to the full sequences of some of their 5′-related human miRNAs (identified in Dataset S13)—45 are weakly similar (<70%) to all their 5′ related human sequences outside the 5′ region. The remaining 15 of the 82 *Drosophila* miRNAs have ≥70% overall homology in addition to 5′ relation to all their 5′ human counterparts. 8 of the 40 *Drosophila* miRNAs with ≥70% homologous sequences in humans show extensive overall similarity with 5′ mismatches: dme-miR-8 with hsa-miR-141 and hsa-miR-200a, dme-miR-10 with hsa-miR-100 and hsa-miR-99a, dme-miR-100 with hsa-miR-10a and hsa-miR-10b, dme-miR-125 with hsa-miR-10a and hsa-miR-10b, dme-miR263a with hsa-miR-183, dme-miR-263b with hsa-miR-183, dme-miR-306 with hsa-miR-873, and dme-miR-993 with hsa-miR-100*.

### miRNAs conserved among nematodes, flies and humans

**Figure 2:**
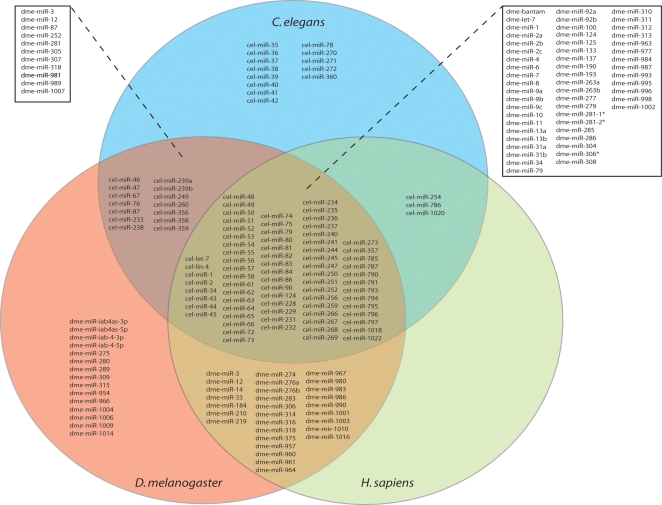
miRNAs of nematode and fly model organisms conserved across species (5′ and/or overall ≥70%). 73/139 *C. elegans* miRNAs share 5′ end identities and/or ≥70% homology over sequence with miRNAs both in fly and humans. 13 *C. elegans* miRNAs currently appear to have sequence-related miRNAs limited to *C. elegans*, 14 miRNAs are shared by nematodes and flies, and 3 miRNAs are shared by nematodes and humans. For *Drosophila*, 54/152 miRNAs have 5′ and/or ≥70% overall homology to nematode and human miRNAs. 15 *D. melanogaster* miRNAs have sequence-related sequences restricted to fly, 11 miRNAs appear present both in fly and nematodes and 29 in fly and humans. Names of family members cross species can be found in Tables 1–6 and sequence alignments in supporting datasets and Figure S1. Human miRNAs that have family members only in human are not included. It should be noted that the Venn diagram is inclusive showing miRNAs that have 5′ and ≥70% overall conserved sequences as well as miRNAs with either 5′ or ≥70% overall conserved sequences. Thus, miRNA totals in the diagram sections do not necessarily match those stated in the main text referring only to 5′ sequence identity or only to ≥70% overall homology. Moreover, dme-miR-3, dme-miR-12 and dme-miR-318 are listed in both fly_nematode and fly_human groups but not in the fly_nematode_human group because their corresponding *C. elegans* and *H. sapiens* homologs are not cross related in sequence under our criteria. Similarly, dme-miR-3, dme-miR-12, dme-miR-263a and dme-miR-318 are included in both fly_nematode and fly_human groups but not in the fly_nematode_human group when only 5′ homology is considered (main text).

miRNAs that are conserved among nematodes, flies and humans are likely to regulate biological functions common between invertebrates and vertebrates. Thus, we had considerable interest in identifying miRNAs that are conserved in these three organisms. We found a total of 73 *C. elegans* miRNAs with identifiable sequence related counterparts shared by nematodes, flies and humans, summarized in Table 6, Venn diagram of Figure 2 and Figure S1.

Limiting our relationship criteria to 5′ end sequence identities, we identified 73 *C. elegans* miRNAs with 5′ homologs in both flies and humans (Table 6, Figure S1). Some *C. elegans* miRNAs have conserved 5′ ends either in flies or humans: 14 nematode miRNAs have 5′ homologs in flies and 3 have 5′ homologs in humans. 10 nematode miRNAs have similar 5′ ends with other *C. elegans* miRNAs that have not yet been found among fly or human miRNAs.

Using extended homology search criteria, we identified 16 *C. elegans* miRNAs that exhibit ≥70% sequence identity with both fly and human counterparts (Table 6, Figure S1). We found that 15 *C. elegans* miRNAs have ≥70% homologous counterparts in flies that are not found in the human genome, possibly lost during evolution of complex higher organisms, or possibly remaining to be discovered in human genomes. 6 nematode miRNAs have ≥70% homologous counterparts in humans but currently lack identifiable family members in *Drosophila*. 28 *C. elegans* miRNAs have ≥70% sequence similarity with other *C. elegans* miRNAs but were not found in either fly or human genomes.

In a similar manner, we inspected the conservation of *D. melanogaster* miRNAs in nematodes, flies and humans. 54 *D. melanogaster* miRNAs have homologous sequences both in nematodes and humans (Figure 2). Searches with 5′ end sequences identified 54 *D. melanogaster* miRNAs with 5′-related sequences in both nematodes and humans, 9 in nematodes and 29 in humans. 11 *D. melanogaster* miRNAs have 5′ related sequences only in flies and are not present or remain unidentified in nematodes and humans. Considering ≥70% identity over the entire length, 21 *D. melanogaster* miRNAs have ≥70% homology counterparts in both nematodes and humans, 16 in nematodes and 19 in humans. 15 *D. melanogaster* miRNAs have ≥70% similarity to only other fly miRNAs.

Overall, analysis of most recent miRBase release data highlights significant conservation of many miRNAs, supporting that analysis of their biological activities in invertebrate models will shed insight into functions relevant to human biology.

## Discussion

### An overview of inter- and intra-species relationships among miRNAs

miRBase release 10.1 (December 2007) identifies 733 human, 139 *C. elegans* and 152 *D. melanogaster* mature miRNAs [43]–[46]. This list of annotated miRNAs, compiled predominantly from large-scale sequencing studies, has grown at an impressive rate in the recent past–for example, the list of human miRNAs has increased by over 500 sequences during the last 3 years. Although miRNA identification efforts are unlikely to yet be complete, current documented miRNAs most likely represent abundant species processed from typical hairpin structures. The field now faces the challenge of determining the biological activities of these miRNAs. Recently, extensive collections of *C. elegans mir* mutants have been generated [48], defining an opportune moment at which to evaluate sequence-related families and conserved functions.

In this paper, we present a comprehensive classification of all the miRBase 10.1 miRNA sequences annotated in *C. elegans*, *D. melanogaster* and humans into sequence-related groups to identify miRNAs with possible redundant functions in the same species and those with potentially conserved functions across species. This compilation, which takes into account the two ways in which functionally related mature miRNAs can be similar (either 5′ end seed homology or homology over length), is based on mature miRNA sequences rather than precursor gene sequence and adds to the considerable numbers of documented sequence-related family members [25], [43], [51], [52], [57], [60], [63]–[65], [75], [76], providing details of sequence relationships.

### Intraspecies analysis: many invertebrate miRNAs have potential for functional redundancy

Looking within individual species, we find that ∼60% (84/139) *C. elegans* miRNAs and ∼46% (70/152) *D. melanogaster* miRNAs share significant homology with other miRNAs encoded by their respective genomes. The potential for functional redundancy of miRNAs is clearly considerable within these species.

The importance of evaluating sequence-related miRNAs during functional analysis has been elegantly exemplified by work on the *C. elegans let-7* miRNA family. Sequence-related miR-48, miR-84 and miR-241 work together to regulate developmental timing by redundant complementarity to binding sites in the 3′ UTR of *hbl-1* [40]. *mir-48*, *mir-84* and *mir-241* single mutants are seemingly wild type at 20°C. However, double and triple combinations of *mir-48*, *mir-84* and *mir-241* mutations cause developmental defects, revealing biological roles for these family members and stressing the importance of the analysis of multiple homologous miRNAs during functional studies. Of course, sequence-related miRNAs might be expressed in different tissues or at different times in development, and therefore might be excluded from performing similar functions with common targets. Still, the extensive sequence relationships that we document underscore that potential co-expression of sequence-related miRNAs will be a significant factor in evaluation of genetic disruptions as well as in commonly executed over-expression studies. Information on the expression patterns of sequence-related miRNAs will be important to careful interpretation of experimental outcomes.

### The extent of conservation of miRNA sequences from invertebrates to humans is striking

Another theme that our analysis underscores is the substantial conservation of miRNA sequences across species. ∼62% *C. elegans* miRNAs are related to *Drosophila* miRNAs (87/139), ∼55% *C. elegans* miRNAs are related to human miRNAs (76/139), and ∼55% *Drosophila* miRNAs are related to human miRNAs (83/152). Over half of the *C. elegans* miRNAs share sequence homology with miRNAs expressed in both flies and humans (73/139), and this number should increase with an increase in reported miRNAs.

The extensive conservation across species suggests that this group of miRNAs contributes important functions in biology and that experiments in one species may well inform on the biology of another. Indeed, cross-species analyses of *let-7* miRNA function has already provided useful leads for addressing human disease regulation. *let-7* represses *C. elegans* RAS ortholog *let-60* [81], as well as the human RAS oncogene transcript [42]. Recently these findings have been extended to demonstrate that *let-7* expression reduces tumor growth in mouse lung tumor models [82].

### Taking stock in a dynamic field

miRNA discovery is an highly active research area. Here we report 133 human miRNAs with related sequences encoded by the *C. elegans* and/or *D. melanogaster* genomes. The majority of cataloged human miRNAs have unknown functions. Gene knock-outs, chemically modified antisense oligonucleotides, decoy miRNA targets (miRNA sponges) and over-expression studies are currently being used to evaluate loss-of-function of miRNAs [48], [83]–[86]. Functional investigation of sequence-related miRNAs from *C. elegans* and *D. melanogaster* in a whole-organism context will most certainly provide insight into miRNA roles in specific mechanisms relevant to normal development as well as disease. The numerous sequence relationships identified to date will help focus research on abundantly expressed, conserved miRNAs while additional miRNA discovery continues to expand known miRNA families.

## Methods

### miRNA sequences and criteria for family grouping

Mature miRNA sequences in *C. elegans*, *D. melanogaster* and *H. sapiens* were retrieved from the miRNA registry release 10.1 (December 2007) in miRBase [43]–[46]. miRNAs in *C. elegans*, *D. melanogaster*, *C. elegans-D. melanogaster*, *C. elegans-H. sapiens* and *D. melanogaster-H. sapiens* were classified into homology groups based on their sequence similarity at the 5′ end (nucleotides 1–10) and/or over full length. 5′ end sequences (10 nt) were considered homologous when they exhibited identity over 7 continuous nucleotides. Only interruptions implying G..U pairing were allowed within the 7 nt identity block. ≥70% overall similarity was the threshold used for grouping full miRNA sequences into families. miRNAs were thus classified as members of a specific family group if they met the criteria of 5′ 7 nt identity or ≥70% overall similarity with a at least one other miRNA member of the group. The sub-groups noted in supporting information contain miRNAs with more closely similar sequences (≥80% overall identity or highly similar 5′ ends). Expanded groupings of miRNAs exhibiting 60–69.9% sequence similarity are also included in supporting information to provide access to potentially related sequences that might be relevant to a given study. 3′ similarity searches were performed with 3′ end sequences (nucleotides 11-3′ end) of *C. elegans* miRNAs.

### miRNA sequence analysis

Analysis of mature miRNA sequences was performed using Clustal X 1.83 [87] and AlignX (a component of Vector NTi Advance 10.3.0, Invitrogen), which are both based on the Clustal W algorithm [70]. Intraspecies sequence-related miRNAs in *C. elegans* and *D. melanogaster* were evaluated by manual examination of multiple sequence alignments and 1000 bootstrapped NJ-trees. Interspecies sequence-related miRNAs were identified by manual inspection of profile alignments, in which all *D. melanogaster* or *H. sapiens* miRNA sequences were aligned against each of the 139 *C. elegans* miRNAs (used as reference sequence) in *C. elegans-D. melanogaster* and *C. elegans-H. sapiens* analyses, and all *H. sapiens* miRNA sequences were aligned against each of the 152 *D. melanogaster* miRNAs (reference) in the *D. melanogaster-H. sapiens* analysis.

### *mir* gene clusters

Coordinates of *mir* genes in the *C. elegans* and *D. melanogaster* genomes were obtained from miRBase release 10.1, December 2007 [43]–[46]. *mir* genes were considered to form part of a cluster if they were positioned on the same DNA strand within a 2 Kb region. Diagrams were designed using Vector NTi Advance 10.3.0 (Invitrogen).

## Supporting Information

Figure S1 Alignments of miRNA sequences conserved across species. See Table 6 and Figure 2. Grey shading identifies potential G..U pairing.
(0.06 MB PDF)

Figure S2 Frequency and distribution of 5′ homologous nucleotides and their correlation with overall sequence conservation. A: Analysis of all 5′ sequence-related miRNAs in *C. elegans* indicates that homologous nucleotides are mainly positioned from nucleotides 2 to 8 (Dataset S1). B: Sequence-related miRNAs with 7 or 8 homologous nucleotides at the 5′ end tend to have poorer sequence similarity at the 3′ end and thus weaker overall similarity than related miRNAs with 9 or 10 5′ nucleotide homologies. miRNAs with 10 5′ homologous nucleotides tend to have significant nucleotide similarities at the 3′ end with an overall sequence identity of 70–100% (Table 1, Dataset S2) or less frequently of 60–69.9% (Dataset S3).
(0.32 MB PDF)

Figure S3 Sequence alignments of *C. elegans* miRNAs with extensive similarity at the 3′ end but poor homology at the 5′ end. *C. elegans* mature miRNAs vary in length from 18 nt to 26 nt, and thus the 3′ end sequences used differed in length to some extent. Since the majority of *C. elegans* miRNAs are 21–23 nt long and on average 22 nt, most of the 3′ end sequences varied 1–2 nt in size. Grey shading denotes potential G..U pairing.
(0.02 MB PDF)

Figure S4 Average distribution of *mir* genes in the *C. elegans* and *D. melanogaster* genomes. Despite additional *mir* genes might still be discovered further concentrating genetic maps, it is worthy of note that a higher proportion of *mir genes* in miRBase 10.1 are located in *C. elegans* chromosome X (A) and in *Drosophila* chromosome pair 2L, 2R (B) than expected by random distribution. The expected number of *mir* genes was determined by dividing total number of *mirs* in the genome by chromosome length.
(0.39 MB PDF)

Dataset S1 Homology table and sequence alignments of *C. elegans* miRNAs with significant identity at the 5′ 10 nt.
(0.39 MB DOC)

Dataset S2 Homology table and sequence alignments of *C. elegans* miRNAs with ≥70% overall sequence identity.
(0.25 MB DOC)

Dataset S3 Table and alignments of related *C. elegans* miRNAs with 60–69.9% overall sequence similarity.
(0.15 MB DOC)

Dataset S4 Homology table and sequence alignments of *D. melanogaster* miRNAs with similar 5′ ends.
(0.12 MB DOC)

Dataset S5 Homology table and alignments of *D. melanogaster* miRNAs showing ≥70% overall sequence identity.
(0.08 MB DOC)

Dataset S6 Table and alignments of *D. melanogaster* miRNA sequences with 60–69.9% similarity.
(0.15 MB DOC)

Dataset S7 Homology table and alignments of *C. elegans* miRNAs related at the 5′ end to *Drosophila* miRNAs.
(0.32 MB DOC)

Dataset S8 Identity table and alignments of *C. elegans* miRNAs with ≥70% full sequence homology to *Drosophila* miRNAs.
(0.09 MB DOC)

Dataset S9 Table and alignments of *C. elegans* and *Drosophila* miRNAs with 60–69.9% similarity over whole sequence.
(0.21 MB DOC)

Dataset S10 Tables and alignments of *C. elegans* and *H. sapiens* miRNAs with homologous 5′ ends.
(0.47 MB DOC)

Dataset S11 Sequence identity table and alignments of *C. elegans*-*H. sapiens* miRNAs with ≥70% overall homology.
(0.12 MB DOC)

Dataset S12 Similarity table and sequence alignments of *C. elegans*-*H. sapiens* miRNAs with 60–69.9% overall identity.
(0.24 MB DOC)

Dataset S13 Table and alignments of *D. melanogaster* and human miRNAs with 5′ homology.
(0.44 MB DOC)

Dataset S14 Table and alignments of *D. melanogaster* and human miRNAs with ≥70% overall sequence homology.
(0.15 MB DOC)

Dataset S15 Similarity table and sequence alignments of *D. melanogaster*-*H. sapiens* miRNAs with 60–69.9% overall identity.
(0.31 MB DOC)

## References

[1] Vasudevan S, Tong Y, Steitz JA (2007). Switching from repression to activation: microRNAs can up-regulate translation.. Science.

[2] Bartel DP (2004). MicroRNAs: Genomics, biogenesis, mechanism, and function.. Cell.

[3] Yang MC, Li Y, Padgett RW (2005). MicroRNAs: Small regulators with a big impact.. Cytokine Growth Factor Rev.

[4] Carthew RW (2006). Gene regulation by microRNAs.. Current Opinion in Genetics & Development.

[5] He L, Hannon GJ (2004). MicroRNAs: Small RNAs with a big role in gene regulation.. Nature Rev Genet.

[6] Esquela-Kerscher A, Slack FJ (2006). Oncomirs–microRNAs with a role in cancer.. Nature Reviews Cancer.

[7] Krutzfeldt J, Poy MN, Stoffel M (2006). Strategies to determine the biological function of microRNAs.. Nature Genetics.

[8] Sempere LF, Freemantle S, Pitha-Rowe I, Moss E, Dmitrovsky E (2004). Expression profiling of mammalian microRNAs uncovers a subset of brain-expressed microRNAs with possible roles in murine and human neuronal differentiation.. Genome Biology.

[9] Miska EA, Alvarez-Saavedra E, Townsend M, Yoshii A, Sestan N (2004). Microarray analysis of microRNA expression in the developing mammalian brain.. Genome Biology.

[10] Landgraf P, Rusu M, Sheridan R, Sewer A, Iovino N (2007). A mammalian microRNA expression atlas based on small RNA library sequencing.. Cell.

[11] Suh MR, Lee Y, Kim JY, Kim SK, Moon SH (2004). Human embryonic stem cells express a unique set of microRNAs.. Developmental Biology.

[12] Wienholds E, Kloosterman WP, Miska E, Alvarez-Saavedra E, Berezikov E (2005). MicroRNA expression in zebrafish embryonic development.. Science.

[13] Ibanez-Ventoso C, Yang M, Guo S, Robins H, Padgett RW (2006). Modulated microRNA expression during adult lifespan in *C. elegans*.. Aging Cell.

[14] Lu J, Getz G, Miska EA, Alvarez-Saavedra E, Lamb J (2005). MicroRNA expression profiles classify human cancers.. Nature.

[15] Jiang J, Lee EJ, Gusev Y, Schmittgen TD (2005). Real-time expression profiling of microRNA precursors in human cancer cell lines.. Nucleic Acids Res.

[16] Volinia S, Calin GA, Liu CG, Ambs S, Cimmino A (2006). A microRNA expression signature of human solid tumors defines cancer gene targets.. Proc Natl Acad Sci U S A.

[17] Aboobaker AA, Tomancak P, Patel N, Rubin GM, Lai EC (2005). Drosophila microRNAs exhibit diverse spatial expression patterns during embryonic development.. Proc Natl Acad Sci U S A.

[18] Kulshreshtha R, Ferracin M, Wojcik SE, Garzon R, Alder H (2007). A microRNA signature of hypoxia.. Mol Cell Biol.

[19] Sonkoly E, Wei T, Janson PC, Saaf A, Lundeberg L (2007). MicroRNAs: novel regulators involved in the pathogenesis of Psoriasis?. PLoS ONE.

[20] Yan N, Lu Y, Sun H, Tao D, Zhang S (2007). A microarray for microRNA profiling in mouse testis tissues.. Reproduction.

[21] Ikeda S, Kong SW, Lu J, Bisping E, Zhang H (2007). Altered microRNA expression in human heart disease.. Physiol Genomics.

[22] Hansen T, Olsen L, Lindow M, Jakobsen KD, Ullum H (2007). Brain expressed microRNAs implicated in schizophrenia etiology.. PLoS ONE.

[23] Wu H, Neilson JR, Kumar P, Manocha M, Shankar P (2007). miRNA Profiling of Naive, Effector and Memory CD8 T Cells.. PLoS ONE.

[24] Ro S, Park C, Sanders KM, McCarrey JR, Yan W (2007). Cloning and expression profiling of testis-expressed microRNAs.. Dev Biol.

[25] Ruby JG, Stark A, Johnston WK, Kellis M, Bartel DP (2007). Evolution, biogenesis, expression, and target predictions of a substantially expanded set of Drosophila microRNAs.. Genome Res.

[26] Lakshmipathy U, Love B, Goff LA, Jornsten R, Graichen R (2007). MicroRNA expression pattern of undifferentiated and differentiated human embryonic stem cells.. Stem Cells Dev.

[27] Arora A, McKay GJ, Simpson DA (2007). Prediction and verification of miRNA expression in human and rat retinas.. Invest Ophthalmol Vis Sci.

[28] Grey F, Hook L, Nelson J (2007). The functions of herpesvirus-encoded microRNAs.. Med Microbiol Immunol.

[29] Karali M, Peluso I, Marigo V, Banfi S (2007). Identification and characterization of microRNAs expressed in the mouse eye.. Invest Ophthalmol Vis Sci.

[30] Tagami Y, Inaba N, Kutsuna N, Kurihara Y, Watanabe Y (2007). Specific Enrichment of miRNAs in Arabidopsis thaliana Infected with Tobacco mosaic virus.. DNA Res.

[31] Zhan M, Miller CP, Papayannopoulou T, Stamatoyannopoulos G, Song CZ (2007). MicroRNA expression dynamics during murine and human erythroid differentiation.. Exp Hematol.

[32] Lewis BP, Burge CB, Bartel DP (2005). Conserved seed pairing, often flanked by adenosines, indicates that thousands of human genes are microRNA targets.. Cell.

[33] Stark A, Brennecke J, Russell RB, Cohen SM (2003). Identification of Drosophila MicroRNA targets.. PLoS Biol.

[34] Enright AJ, John B, Gaul U, Tuschl T, Sander C (2003). MicroRNA targets in Drosophila.. Genome Biology.

[35] Rajewsky N (2006). microRNA target predictions in animals.. Nature Genetics.

[36] Grun D, Wang YL, Langenberger D, Gunsalus KC, Rajewsky N (2005). microRNA target predictions across seven *Drosophila* species and comparison to mammalian targets.. PLoS Computational Biology.

[37] John B, Enright AJ, Aravin A, Tuschl T, Sander C (2004). Human MicroRNA targets.. PLoS Biol.

[38] Robins H, Li Y, Padgett RW (2005). Incorporating structure to predict microRNA targets.. Proc Natl Acad Sci USA.

[39] Hayes GD, Frand AR, Ruvkun G (2006). The mir-84 and let-7 paralogous microRNA genes of *Caenorhabditis elegans* direct the cessation of molting via the conserved nuclear hormone receptors NHR-23 and NHR-25.. Development.

[40] Abbott AL, Alvarez-Saavedra E, Miska EA, Lau NC, Bartel DP (2005). The *let-7* microRNA family members *mir-48*, *mir-84*, and *mir-241* function together to regulate developmental timing in *Caenorhabditis elegans*.. Develop Cell.

[41] Brennecke J, Stark A, Russell RB, Cohen SM (2005). Principles of microRNA-target recognition.. PLoS Biology.

[42] Johnson SM, Grosshans H, Shingara J, Byrom M, Jarvis R (2005). RAS is regulated by the *let-7* microRNA family.. Cell.

[43] miRBase (http://www.microrna.sanger.ac.uk)

[44] Griffiths-Jones S (2004). The microRNA Registry.. Nucleic Acids Research.

[45] Griffiths-Jones S, Grocock RJ, van Dongen S, Bateman A, Enright AJ (2006). miRBase: microRNA sequences, targets and gene nomenclature.. Nucleic Acids Res.

[46] Griffiths-Jones S, Saini HK, van Dongen S, Enright AJ (2008). miRBase: tools for microRNA genomics.. Nucleic Acids Research.

[47] Bentwich I, Avniel A, Karov Y, Aharonov R, Gilad S (2005). Identification of hundreds of conserved and nonconserved human microRNAs.. Nature Genetics.

[48] Miska EA, Alvarez-Saavedra E, Abbott AL, Lau NC, Hellman AB (2007). Most *Caenorhabditis elegans* microRNAs are individually not essential for development or viability.. PLoS Genet.

[49] National Bioresource Project for the Experimental Animal “Nematode *C. elegans*”: http://shigen.lab.nig.ac.jp/c.elegans/ChangeLocale.do?url=home&lang=en 10.1538/expanim.58.351 19654432

[50] The *C. elegans* Gene Knockout Consortium: http://celeganskoconsortium.omrf.org/

[51] Ambros V, Lee RC, Lavanway A, Williams PT, Jewell D (2003). MicroRNAs and other tiny endogenous RNAs in *C. elegans*.. Current Biology.

[52] Aravin AA, Lagos-Quintana M, Yalcin A, Zavolan M, Marks D (2003). The small RNA profile during *Drosophila melanogaster* development.. Developmental Cell.

[53] Berezikov E, van Tetering G, Verheul M, van de Belt J, van Laake L (2006). Many novel mammalian microRNA candidates identified by extensive cloning and RAKE analysis.. Genome Research.

[54] Cummins JM, He Y, Leary RJ, Pagliarini R, Diaz LA (2006). The colorectal microRNAome.. Proc Natl Acad Sci USA.

[55] Dostie J, Mourelatos Z, Yang M, Sharma A, Dreyfuss G (2003). Numerous microRNPs in neuronal cells containing novel microRNAs.. RNA.

[56] Fu H, Tie Y, Xu C, Zhang Z, Zhu J (2005). Identification of human fetal liver miRNAs by a novel method.. FEBS Letters.

[57] Lagos-Quintana M, Rauhut R, Lendeckel W, Tuschl T (2001). Identification of novel genes coding for small expressed RNAs.. Science.

[58] Lagos-Quintana M, Rauhut R, Meyer J, Borkhardt A, Tuschl T (2003). New microRNAs from mouse and human.. RNA.

[59] Lagos-Quintana M, Rauhut R, Yalcin A, Meyer J, Lendeckel W (2002). Identification of tissue-specific microRNAs from mouse.. Current Biology.

[60] Lai EC, Tomancak P, Williams RW, Rubin GM (2003). Computational identification of *Drosophila* microRNA genes.. Genome Biology.

[61] Lau NC, Lim LP, Weinstein EG, Bartel DP (2001). An abundant class of tiny RNAs with probable regulatory roles in *Caenorhabditis elegans*.. Science.

[62] Lim LP, Glasner ME, Yekta S, Burge CB, Bartel DP (2003). Vertebrate MicroRNA genes.. Science.

[63] Lim LP, Lau NC, Weinstein EG, Abdelhakim A, Yekta S (2003). The microRNAs of *Caenorhabditis elegans*.. Genes & Development.

[64] Ruby JG, Jan C, Player C, Axtell MJ, Lee W (2006). Large-Scale sequencing reveals 21U-RNAs and additional microRNAs and endogenous siRNAs in *C. elegans*.. Cell.

[65] Sandmann T, Cohen SM (2007). Identification of novel *Drosophila melanogaster* MicroRNAs.. PLoS ONE.

[66] Stark A, Lin MF, Kheradpour P, Pedersen JS, Parts L (2007). Discovery of functional elements in 12 Drosophila genomes using evolutionary signatures.. Nature.

[67] Weber MJ (2005). New human and mouse microRNA genes found by homology search.. FEBS Letters.

[68] Lui WO, Pourmand N, Patterson BK, Fire A (2007). Patterns of known and novel small RNAs in human cervical cancer.. Cancer Research.

[69] Novotny GW, Nielsen JE, Sonne SB, Skakkebaek NE, Rajpert-De Meyts E (2007). Analysis of gene expression in normal and neoplastic human testis: new roles of RNA.. International Journal of Andrology.

[70] Thompson JD, Higgins DG, Gibson TJ (1994). CLUSTAL W: improving the sensitivity of progressive multiple sequence alignment through sequence weighting, position-specific gap penalties and weight matrix choice.. Nucleic Acids Research.

[71] Doench JG, Sharp PA (2004). Specificity of microRNA target selection in translational repression.. Genes & Development.

[72] Lai EC, Tam B, Rubin GM (2005). Pervasive regulation of *Drosophila* Notch target genes by GY-box, Brd-Box, and K-box-class microRNAs.. Genes & Development.

[73] Didiano D, Hobert O (2006). Perfect seed pairing is not a generally reliable predictor for miRNA-target interactions.. Nature Structural & Molecular Biology.

[74] Lewis BP, Shih IH, Jones-Rhoades MW, Bartel DP, Burge CB (2003). Prediction of mammalian microRNA targets.. Cell.

[75] Sempere LF, Sokol NS, Dubrovsky EB, Berger EM, Ambros V (2003). Temporal regulation of microRNA expression in *Drosophila melanogaster* mediated by hormonal signals and Broad-Complex gene activity.. Developmental Biology.

[76] Huang Y, Gu X (2007). A bootstrap based analysis pipeline for efficient classification of phylogenetically related animal miRNAs.. BMC Genomics.

[77] Hwang H, Wentzel EA, Mendell JT (2007). A hexanucleotide element directs microRNA nuclear import.. Science.

[78] Rodriguez A, Griffiths-Jones S, Ashurst JL, Bradley A (2004). Identification of mammalian microRNA host genes and transcription units.. Genome Research.

[79] Altuvia Y, Landgraf P, Lithwick G, Elefant N, Pfeffer S (2005). Clustering and conservation patterns of human microRNAs.. Nucleic Acids Research.

[80] Esquela-Kerscher A, Johnson SM, Bai L, Saito K, Partridge J (2005). Post-embryonic expression of *C. elegans* microRNAs belonging to the *lin-4* and *let-7* families in the hypodermis and the reproductive system.. Developmental Dynamics.

[81] Han M, Sternberg PW (1990). let-60, a gene that specifies cell fates during *C. elegans* vulval induction, encodes a ras protein.. Cell.

[82] Esquela-Kerscher A, Trang P, Wiggins JF, Patrawala L, Cheng A (2008). The *let-7* microRNA reduces tumor growth in mouse models of lung cancer.. Cell Cycle.

[83] Ebert MS, Neilson JR, Sharp PA (2007). MicroRNA sponges: competitive inhibitors of small RNAs in mammalian cells.. Nat Methods.

[84] Esau CC (2008). Inhibition of microRNA with antisense oligonucleotides.. Methods.

[85] Hutvagner G, Simard MJ, Mello CC, Zamore PD (2004). Sequence-specific inhibition of small RNA function.. PLoS Biology.

[86] Orom UA, Kauppinen S, Lund AH (2006). LNA-modified oligonucleotides mediate specific inhibition of microRNA function.. Gene.

[87] Thompson JD, Gibson TJ, Plewniak F, Jeanmougin F, Higgins DG (1997). The CLUSTAL_X windows interface: flexible strategies for multiple sequence alignment aided by quality analysis tools.. Nucleic Acids Research.

