# Supplementary material for: Sequence Relationships among C. elegans, D. melanogaster and Human microRNAs Highlight the Extensive Conservation of microRNAs in Biology
Source: PLoS One. 2008 Jul 30;3(7):e2818. doi: 10.1371/journal.pone.0002818 (PMC2486268; doi:10.1371/journal.pone.0002818)
Supplement: Figure S1 — Alignments of miRNA sequences conserved across species. See Table 6 and Figure 2. Grey shading identifies potential G..U pairing. (0.06 MB PDF) [file pone.0002818.s001.pdf]

cel-let-7:

```
            1                      24
  cel-let-7 UGAGGUAG-UA-GGUUGUAUAGUU
  dme-let-7 UGAGGUAG-UA-GGUUGUAUAGU-
 hsa-let-7a UGAGGUAG-UA-GGUUGUAUAGUU
 hsa-let-7b UGAGGUAG-UA-GGUUGUGUGGUU
 hsa-let-7c UGAGGUAG-UA-GGUUGUAUGGUU
 hsa-let-7d AGAGGUAG-UA-GGUUGCAUAGUU
 hsa-let-7e UGAGGUAG-GA-GGUUGUAUAGUU
 hsa-let-7g UGAGGUAG-UA-GUUUGUACAGUU
 hsa-let-7f UGAGGUAG-UA-GAUUGUAUAGUU
dme-miR-984 UGAGGUAAAUACGGUUGGAAUUU-
 cel-miR-84 UGAGGUAG-UA-UGUAAUAUUGUA
 hsa-miR-98 UGAGGUAG-UA-AGUUGUAUUGUU
 hsa-let-7i UGAGGUAG-UA-GUUUGUGCUGUU
```

```
             1
   cel-let-7 -UGAGGUAGUA-
  cel-miR-84 -UGAGGUAGUA-
   dme-let-7 -UGAGGUAGUA-
  hsa-let-7a -UGAGGUAGUA-
  hsa-let-7b -UGAGGUAGUA-
  hsa-let-7c -UGAGGUAGUA-
  hsa-let-7d -AGAGGUAGUA-
  hsa-let-7e -UGAGGUAGGA-
  hsa-let-7f -UGAGGUAGUA-
  hsa-let-7g -UGAGGUAGUA-
  hsa-let-7i -UGAGGUAGUA-
  hsa-miR-98 -UGAGGUAGUA-
 cel-miR-241 -UGAGGUAGGU-
  cel-miR-48 -UGAGGUAGGC-
 cel-miR-793 -UGAGGUAUCU-
 cel-miR-794 -UGAGGUAAUC-
 cel-miR-795 -UGAGGUAGAU-
 dme-miR-984 -UGAGGUAAAU-
hsa-miR-196a --UAGGUAGUUU
hsa-miR-196b --UAGGUAGUUU
 dme-miR-963 ACAAGGUAAA--
 dme-miR-977 -UGAGAUAUUC-
```

cel-lin-4:

```
                 1                      24
      cel-lin-4  UCCCUGAGACCUC---AAGUGUGA
hsa-miR-125a-5p  UCCCUGAGACCCUUUAACCUGUGA
    dme-miR-125  UCCCUGAGACCCU--AACUUGUGA
   hsa-miR-125b  UCCCUGAGACCCU--AACUUGUGA
```

```
                 1
      cel-lin-4 -UCCCUGAGAC
    dme-miR-125 -UCCCUGAGAC
hsa-miR-125a-5p -UCCCUGAGAC
   hsa-miR-125b -UCCCUGAGAC
    cel-miR-237 -UCCCUGAGAA
 hsa-miR-331-3p GCCCCUGGGC-
```

cel-miR-1:

```
            1                    22
  cel-miR-1 UGGAAUGUAAAGAAGUAUGUA-
  hsa-miR-1 UGGAAUGUAAAGAAGUAUGUAU
  dme-miR-1 UGGAAUGUAAAGAAGUAUGGAG
cel-miR-256 UGGAAUGCAUAGAAGACUGUA-
hsa-miR-206 UGGAAUGUAAGGAAGUGUGUGG
```

```
            1        10
  cel-miR-1 UGGAAUGUAA
  dme-miR-1 UGGAAUGUAA
  hsa-miR-1 UGGAAUGUAA
hsa-miR-206 UGGAAUGUAA
cel-miR-796 UGGAAUGUAG
cel-miR-256 UGGAAUGCAU
hsa-miR-122 UGGAGUGUGA
```

cel-miR-2:

```
                 1
      cel-miR-2  --UAUCACAGCC
    cel-miR-250  --AAUCACAGUC
    dme-miR-308  --AAUCACAGGA
     dme-miR-11  --CAUCACAGUC
 hsa-miR-499-3p  AACAUCACAG--
     cel-miR-43  --UAUCACAGUU
      dme-miR-6  --UAUCACAGUG
    cel-miR-797  --UAUCACAGCA
    dme-miR-13a  --UAUCACAGCC
    dme-miR-13b  --UAUCACAGCC
     dme-miR-2a  --UAUCACAGCC
     dme-miR-2b  --UAUCACAGCC
     dme-miR-2c  --UAUCACAGCC
```

cel-miR-34:

```
                 1                         27
     cel-miR-34  -AGGCAGUGUGG--UUAGCUGGUUG--
     dme-miR-34  -UGGCAGUGUGG--UUAGCUGGUUGUG
    hsa-miR-34a  -UGGCAGUGUC---UUAGCUGGUUGU-
   hsa-miR-34b*  UAGGCAGUGUCA--UUAGCUGAUUG--
 hsa-miR-34c-5p  -AGGCAGUGUAG--UUAGCUGAUUGC-
   hsa-miR-449a  -UGGCAGUGUAUUGUUAGCUGGU----
   hsa-miR-449b  -AGGCAGUGUAUUGUUAGCUGGC----
```

```
                 1
     cel-miR-34  -AGGCAGUGUG
     dme-miR-34  -UGGCAGUGUG
    hsa-miR-34a  -UGGCAGUGUC
   hsa-miR-34b*  UAGGCAGUGU-
 hsa-miR-34c-5p  -AGGCAGUGUA
   hsa-miR-449a  -UGGCAGUGUA
   hsa-miR-449b  -AGGCAGUGUA
```

cel-miR-43:

```
                1
     cel-miR-43 --UAUCACAGUU--
      cel-miR-2 --UAUCACAGCC--
      dme-miR-6 --UAUCACAGUG--
```

```
    cel-miR-797   --UAUCACAGCA--
    dme-miR-13a   --UAUCACAGCC--
     dme-miR-2a   --UAUCACAGCC--
     dme-miR-2b   --UAUCACAGCC--
     dme-miR-2c   --UAUCACAGCC--
    dme-miR-13b   --UAUCACAGCC--
    cel-miR-250   --AAUCACAGUC--
    dme-miR-308   --AAUCACAGGA--
     dme-miR-11   --CAUCACAGUC--
 hsa-miR-499-3p   AACAUCACAG----
    hsa-miR-27a   ---UUCACAGUGG-
    hsa-miR-27b   ---UUCACAGUGG-
    hsa-miR-128   ----UCACAGUGAA
 hsa-miR-768-3p   ----UCACAAUGCU
```

cel-miR-44:

```
               1
   cel-miR-44  --UGACUAGAGA
   cel-miR-45  --UGACUAGAGA
  cel-miR-247  --UGACUAGAGC
  hsa-miR-134  UGUGACUGGU--
  dme-miR-996  --UGACUAGAUU
 hsa-miR-708*  --CAACUAGACU
  dme-miR-279  --UGACUAGAUC
   cel-miR-61  --UGACUAGAAC
  dme-miR-286  --UGACUAGACC
```

cel-miR-45:

```
               1
   cel-miR-45  --UGACUAGAGA
   cel-miR-44  --UGACUAGAGA
  cel-miR-247  --UGACUAGAGC
  hsa-miR-134  UGUGACUGGU--
  dme-miR-996  --UGACUAGAUU
 hsa-miR-708*  --CAACUAGACU
  dme-miR-279  --UGACUAGAUC
   cel-miR-61  --UGACUAGAAC
  dme-miR-286  --UGACUAGACC
```

cel-miR-48:

```
              1
 cel-miR-48   -UGAGGUAGGC
  cel-let-7   -UGAGGUAGUA
  dme-let-7   -UGAGGUAGUA
 cel-miR-84   -UGAGGUAGUA
 hsa-let-7a   -UGAGGUAGUA
 hsa-let-7b   -UGAGGUAGUA
 hsa-let-7c   -UGAGGUAGUA
 hsa-let-7d   -AGAGGUAGUA
 hsa-let-7e   -UGAGGUAGGA
 hsa-let-7f   -UGAGGUAGUA
 hsa-let-7g   -UGAGGUAGUA
 hsa-let-7i   -UGAGGUAGUA
 hsa-miR-98   -UGAGGUAGUA
cel-miR-795   -UGAGGUAGAU
cel-miR-241   -UGAGGUAGGU
cel-miR-793   -UGAGGUAUCU
cel-miR-794   -UGAGGUAAUC
dme-miR-984   -UGAGGUAAAU
dme-miR-963   ACAAGGUAAA-
dme-miR-977   -UGAGAUAUUC
```

cel-miR-49:

```
                1        10
  cel-miR-49    AAGCACCACG
hsa-miR-593*    AGGCACCAGC
 hsa-miR-21*    CAACACCAGU
  cel-miR-83    UAGCACCAUA
 dme-miR-995    UAGCACCACA
 dme-miR-285    UAGCACCAUU
 hsa-miR-29b    UAGCACCAUU
 hsa-miR-29c    UAGCACCAUU
 dme-miR-998    UAGCACCAUG
 hsa-miR-29a    UAGCACCAUC
```

cel-miR-50:

```
               1                      24
  cel-miR-50    UGAUAUGUCUGGUAUUCUUGGG--
 dme-miR-190    AGAUAUGUUUGAUAUUCUUGGUUG
 hsa-miR-190    UGAUAUGUUUGAUAUAUUAGGU--
hsa-miR-190b    UGAUAUGUUUGAUAU--UGGGUU-
```

```
               1        10
  cel-miR-50   UGAUAUGUCU
  cel-miR-62   UGAUAUGUAA
  cel-miR-90   UGAUAUGUUG
 dme-miR-190   AGAUAUGUUU
 hsa-miR-190   UGAUAUGUUU
hsa-miR-190b   UGAUAUGUUU
```

cel-miR-51:

```
              1
 cel-miR-51   UACCCGUAGC--
 cel-miR-52   CACCCGUACA--
 cel-miR-53   CACCCGUACA--
 cel-miR-54   UACCCGUAAU--
 cel-miR-55   UACCCGUAUA--
 cel-miR-56   UACCCGUAAU--
dme-miR-100   AACCCGUAAA--
hsa-miR-100   AACCCGUAGA--
hsa-miR-99a   AACCCGUAGA--
hsa-miR-99b   CACCCGUAGA--
cel-miR-273   UGCCCGUACU--
cel-miR-267   --CCCGUGAAGU
```

cel-miR-52:

```
               1        10
cel-miR-273    UGCCCGUACU
 cel-miR-54    UACCCGUAAU
 cel-miR-56    UACCCGUAAU
 cel-miR-51    UACCCGUAGC
 cel-miR-55    UACCCGUAUA
 cel-miR-52    CACCCGUACA
 cel-miR-53    CACCCGUACA
hsa-miR-99b    CACCCGUAGA
dme-miR-100    AACCCGUAAA
hsa-miR-100    AACCCGUAGA
hsa-miR-99a    AACCCGUAGA
```

cel-miR-53:

```
               1       10
cel-miR-273    UGCCCGUACU
 cel-miR-54    UACCCGUAAU
 cel-miR-56    UACCCGUAAU
 cel-miR-51    UACCCGUAGC
 cel-miR-55    UACCCGUAUA
 cel-miR-52    CACCCGUACA
 cel-miR-53    CACCCGUACA
hsa-miR-99b    CACCCGUAGA
dme-miR-100    AACCCGUAAA
hsa-miR-100    AACCCGUAGA
hsa-miR-99a    AACCCGUAGA
```

cel-miR-54:

```
               1
cel-miR-267    --CCCGUGAAGU
dme-miR-100    AACCCGUAAA--
cel-miR-273    UGCCCGUACU--
cel-miR-360    UGACCGUAAU--
 cel-miR-54    UACCCGUAAU--
 cel-miR-56    UACCCGUAAU--
 cel-miR-51    UACCCGUAGC--
 cel-miR-55    UACCCGUAUA--
 cel-miR-52    CACCCGUACA--
 cel-miR-53    CACCCGUACA--
hsa-miR-99b    CACCCGUAGA--
hsa-miR-100    AACCCGUAGA--
hsa-miR-99a    AACCCGUAGA--
```

cel-miR-55:

```
               1       10
cel-miR-273    UGCCCGUACU
 cel-miR-54    UACCCGUAAU
 cel-miR-56    UACCCGUAAU
 cel-miR-51    UACCCGUAGC
 cel-miR-55    UACCCGUAUA
 cel-miR-52    CACCCGUACA
 cel-miR-53    CACCCGUACA
hsa-miR-99b    CACCCGUAGA
dme-miR-100    AACCCGUAAA
hsa-miR-100    AACCCGUAGA
hsa-miR-99a    AACCCGUAGA
```

cel-miR-56:

```
               1
cel-miR-267    --CCCGUGAAGU
dme-miR-100    AACCCGUAAA--
cel-miR-273    UGCCCGUACU--
cel-miR-360    UGACCGUAAU--
 cel-miR-54    UACCCGUAAU--
 cel-miR-56    UACCCGUAAU--
 cel-miR-51    UACCCGUAGC--
 cel-miR-55    UACCCGUAUA--
 cel-miR-52    CACCCGUACA--
 cel-miR-53    CACCCGUACA--
hsa-miR-99b    CACCCGUAGA--
hsa-miR-100    AACCCGUAGA--
hsa-miR-99a    AACCCGUAGA--
```

cel-miR-57:

```
                   1
     cel-miR-57    UACCCUGUAG-
    hsa-miR-10a    UACCCUGUAG-
    hsa-miR-10b    UACCCUGUAG-
     dme-miR-10    -ACCCUGUAGA
hsa-miR-146b-3p    UGCCCUGUGG-
```

cel-miR-58:

```
                   1
     cel-miR-58    --UGAGAUCGUU-
     cel-miR-80    --UGAGAUCAUU-
     cel-miR-81    --UGAGAUCAUC-
     cel-miR-82    --UGAGAUCAUC-
     dme-bantam    --UGAGAUCAUU-
hsa-miR-450b-3p    -UUGGGAUCAU--
   cel-miR-1018    AGAGAGAUCA---
   cel-miR-1022    ---AAGAUCAUUG
   dme-miR-306*    --GGGGUCACU-
```

cel-miR-61:

```
             1
 cel-miR-61   --UGACUAGAAC
 cel-miR-247  --UGACUAGAGC
 cel-miR-44   --UGACUAGAGA
 cel-miR-45   --UGACUAGAGA
 dme-miR-279  --UGACUAGAUC
 dme-miR-286  --UGACUAGACC
 dme-miR-996  --UGACUAGAUU
 hsa-miR-134  UGUGACUGGU--
 hsa-miR-708* --CAACUAGACU
```

cel-miR-62

```
             1       10
 cel-miR-62   UGAUAUGUAA
 cel-miR-50   UGAUAUGUCU
 cel-miR-90   UGAUAUGUUG
 hsa-miR-190  UGAUAUGUUU
 dme-miR-190  AGAUAUGUUU
 hsa-miR-190b UGAUAUGUUU
```

cel-miR-63:

```
             1
 cel-miR-63   UAUGACACUG--
 cel-miR-64   UAUGACACUG--
 cel-miR-65   UAUGACACUG--
 cel-miR-66   CAUGACACUG--
 cel-miR-229  AAUGACACUG--
 cel-miR-228  AAUGGCACUG--
 cel-miR-790  CUUGGCACUC--
 cel-miR-791  UUUGGCACUC--
 dme-miR-263b CUUGGCACUG--
 hsa-miR-183  UAUGGCACUG--
 hsa-miR-200a --UAACACUGUC
 hsa-miR-514  AUUGACACUU--
 hsa-miR-96   UUUGGCACUA--
```

cel-miR-64:

```
             1
 cel-miR-64   UAUGACACUG--
 cel-miR-63   UAUGACACUG--
 cel-miR-65   UAUGACACUG--
 cel-miR-66   CAUGACACUG--
 cel-miR-229  AAUGACACUG--
 cel-miR-228  AAUGGCACUG--
 cel-miR-790  CUUGGCACUC--
 cel-miR-791  UUUGGCACUC--
 dme-miR-263b CUUGGCACUG--
 hsa-miR-183  UAUGGCACUG--
 hsa-miR-200a --UAACACUGUC
 hsa-miR-514  AUUGACACUU--
 hsa-miR-96   UUUGGCACUA--
```

cel-miR-65:

```
             1
 cel-miR-65   UAUGACACUG--
 cel-miR-64   UAUGACACUG--
 cel-miR-63   UAUGACACUG--
 cel-miR-66   CAUGACACUG--
 cel-miR-229  AAUGACACUG--
 cel-miR-228  AAUGGCACUG--
 cel-miR-790  CUUGGCACUC--
 cel-miR-791  UUUGGCACUC--
 dme-miR-263b CUUGGCACUG--
 hsa-miR-183  UAUGGCACUG--
 hsa-miR-200a --UAACACUGUC
 hsa-miR-514  AUUGACACUU--
 hsa-miR-96   UUUGGCACUA--
```

cel-miR-66:

```
               1
  cel-miR-66   CAUGACACUG--
  cel-miR-65   UAUGACACUG--
  cel-miR-64   UAUGACACUG--
  cel-miR-63   UAUGACACUG--
 cel-miR-229   AAUGACACUG--
 cel-miR-228   AAUGGCACUG--
 cel-miR-790   CUUGGCACUC--
 cel-miR-791   UUUGGCACUC--
dme-miR-263b   CUUGGCACUG--
 hsa-miR-183   UAUGGCACUG--
hsa-miR-200a   --UAACACUGUC
 hsa-miR-514   AUUGACACUU--
  hsa-miR-96   UUUGGCACUA--
```

cel-miR-72:

```
              1                    23
 cel-miR-72   AGGCAAGAUGUUGGCAUAGCUGA
 hsa-miR-31   AGGCAAGAUGCUGGCAUAGCU--
cel-miR-266   AGGCAAGACUUUGGCAAAGC---
dme-miR-31a   UGGCAAGAUGUCGGCAUAGCUGA
dme-miR-31b   UGGCAAGAUGUCGGAAUAGCUG-
```

```
              1
 cel-miR-72   AGGCAAGAUG-
 cel-miR-73   UGGCAAGAUG-
dme-miR-31a   UGGCAAGAUG-
dme-miR-31b   UGGCAAGAUG-
 hsa-miR-31   AGGCAAGAUG-
 cel-miR-74   UGGCAAGAAA-
cel-miR-266   AGGCAAGACU-
cel-miR-268   -GGCAAGAAUU
cel-miR-269   -GGCAAGACUC
```

cel-miR-73:

```
              1
 cel-miR-73   UGGCAAGAUG-
 cel-miR-74   UGGCAAGAAA-
 cel-miR-72   AGGCAAGAUG-
dme-miR-31a   UGGCAAGAUG-
dme-miR-31b   UGGCAAGAUG-
 hsa-miR-31   AGGCAAGAUG-
cel-miR-266   AGGCAAGACU-
cel-miR-269   -GGCAAGACUC
cel-miR-268   -GGCAAGAAUU
```

cel-miR-74:

```
                1
  cel-miR-74    -UGGCAAGAAA--
  cel-miR-73    -UGGCAAGAUG--
  cel-miR-72    -AGGCAAGAUG--
 cel-miR-266    -AGGCAAGACU--
 cel-miR-268    --GGCAAGAAUU-
 cel-miR-269    --GGCAAGACUC-
 dme-miR-31a    -UGGCAAGAUG--
 dme-miR-31b    -UGGCAAGAUG--
  hsa-miR-31    -AGGCAAGAUG--
hsa-miR-513b    UUCACAAGGA---
 hsa-miR-873    ---GCAGGAACUU
```

cel-miR-75:

```
                   1
     cel-miR-75   -UUAAAGCUAC-
     dme-miR-79   --UAAAGCUAGA
     cel-miR-79   -AUAAAGCUAG-
      dme-miR-4   -AUAAAGCUAG-
     hsa-miR-9*   -AUAAAGCUAG-
    hsa-miR-320   --AAAAGCUGGG
hsa-miR-548a-3p   --CAAAACUGGC
 dme-miR-281-1*   AAGAGAGCUG--
 dme-miR-281-2*   AAGAGAGCUA--
```

cel-miR-79:

```
               1                      23
cel-miR-79     AUAAAGCUAGGUUACCAAAGCU-
dme-miR-79     -UAAAGCUAGAUUACCAAAGCAU
hsa-miR-9*     AUAAAGCUAGAUAACCGAAAGU-
```

```
                  1
       cel-miR-79    --AUAAAGCUAG-
       cel-miR-75    --UUAAAGCUAC-
       hsa-miR-9*    --AUAAAGCUAG-
         dme-miR-4   --AUAAAGCUAG-
       dme-miR-79    ---UAAAGCUAGA
      hsa-miR-340    UUAUAAAGCA---
   dme-miR-281-1*    -AAGAGAGCUG--
   dme-miR-281-2*    -AAGAGAGCUA--
      hsa-miR-320    ---AAAAGCUGGG
         dme-miR-7   -UGGAAGACUA--
hsa-miR-548a-3p      ---CAAAACUGGC
         hsa-miR-7   -UGGAAGACUA--
```

cel-miR-80:

```
                  1
       cel-miR-80    --UGAGAUCAUU-
       dme-bantam    --UGAGAUCAUU-
       cel-miR-81    --UGAGAUCAUC-
       cel-miR-82    --UGAGAUCAUC-
       cel-miR-58    --UGAGAUCGUU-
     cel-miR-1022    ---AAGAUCAUUG
     cel-miR-1018    AGAGAGAUCA---
     dme-miR-306*    --GGGGGUCACU-
hsa-miR-450b-3p      -UUGGGAUCAU--
```

cel-miR-81:

```
                  1
       cel-miR-81    --UGAGAUCAUC-
       cel-miR-82    --UGAGAUCAUC-
       cel-miR-80    --UGAGAUCAUU-
       dme-bantam    --UGAGAUCAUU-
       cel-miR-58    --UGAGAUCGUU-
```

```
     cel-miR-1022    ---AAGAUCAUUG
     cel-miR-1018    AGAGAGAUCA---
     dme-miR-306*    --GGGGGUCACU-
hsa-miR-450b-3p      -UUGGGAUCAU--
```

cel-miR-82:

```
                  1
       cel-miR-82    --UGAGAUCAUC-
       cel-miR-81    --UGAGAUCAUC-
       cel-miR-80    --UGAGAUCAUU-
       dme-bantam    --UGAGAUCAUU-
       cel-miR-58    --UGAGAUCGUU-
     cel-miR-1022    ---AAGAUCAUUG
     cel-miR-1018    AGAGAGAUCA---
     dme-miR-306*    --GGGGGUCACU-
hsa-miR-450b-3p      -UUGGGAUCAU--
```

cel-miR-83:

```
               1                      23
  cel-miR-83   UAGCACCAUAUAAAUUCAGUAA-
dme-miR-285    UAGCACCAUUCGAAAUCAGUGC-
dme-miR-998    UAGCACCAUGAGA-UUCAGCUC-
hsa-miR-29a    UAGCACCAUCUGAAAUCGGUUA-
hsa-miR-29b    UAGCACCAUUUGAAAUCAGUGUU
hsa-miR-29c    UAGCACCAUUUGAAAUCGGUUA-
```

```
                 1        10
   cel-miR-83    UAGCACCAUA
  dme-miR-285    UAGCACCAUU
  hsa-miR-29b    UAGCACCAUU
  hsa-miR-29c    UAGCACCAUU
  dme-miR-998    UAGCACCAUG
  hsa-miR-29a    UAGCACCAUC
  dme-miR-995    UAGCACCACA
   cel-miR-49    AAGCACCACG
 hsa-miR-593*    AGGCACCAGC
  hsa-miR-21*    CAACACCAGU
```

cel-miR-84:

```
              1                    22
cel-miR-84    UGAGGUAGUAUGUAAUAUUGUA
 cel-let-7    UGAGGUAGUAGGUUGUAUAGUU
 dme-let-7    UGAGGUAGUAGGUUGUAUAGU-
hsa-let-7a    UGAGGUAGUAGGUUGUAUAGUU
hsa-let-7b    UGAGGUAGUAGGUUGUGUGGUU
hsa-let-7c    UGAGGUAGUAGGUUGUAUGGUU
hsa-let-7e    UGAGGUAGGAGGUUGUAUAGUU
hsa-let-7f    UGAGGUAGUAGAUUGUAUAGUU
hsa-miR-98    UGAGGUAGUAAGUUGUAUUGUU
```

```
                 1
  cel-miR-84    -UGAGGUAGUA-
   cel-let-7    -UGAGGUAGUA-
 cel-miR-241    -UGAGGUAGGU-
  cel-miR-48    -UGAGGUAGGC-
 cel-miR-793    -UGAGGUAUCU-
 cel-miR-794    -UGAGGUAAUC-
 cel-miR-795    -UGAGGUAGAU-
   dme-let-7    -UGAGGUAGUA-
 dme-miR-963    ACAAGGUAAA--
 dme-miR-977    -UGAGAUAUUC-
 dme-miR-984    -UGAGGUAAAU-
  hsa-let-7a    -UGAGGUAGUA-
  hsa-let-7b    -UGAGGUAGUA-
  hsa-let-7c    -UGAGGUAGUA-
  hsa-let-7d    -AGAGGUAGUA-
  hsa-let-7e    -UGAGGUAGGA-
  hsa-let-7f    -UGAGGUAGUA-
  hsa-let-7g    -UGAGGUAGUA-
  hsa-let-7i    -UGAGGUAGUA-
hsa-miR-196a    --UAGGUAGUUU
hsa-miR-196b    --UAGGUAGUUU
  hsa-miR-98    -UGAGGUAGUA-
```

cel-miR-86:

```
                1
  cel-miR-86    -UAAGUGAAUG
 cel-miR-785    -UAAGUGAAUU
 dme-miR-987    UAAAGUAAAU-
 hsa-miR-559    UAAAGUAAAU-
hsa-miR-545*    -UCAGUAAAUG
```

cel-miR-90:

```
                1       10
  cel-miR-90    UGAUAUGUUG
 dme-miR-190    AGAUAUGUUU
 hsa-miR-190    UGAUAUGUUU
hsa-miR-190b    UGAUAUGUUU
  cel-miR-50    UGAUAUGUCU
  cel-miR-62    UGAUAUGUAA
```

cel-miR-124:

```
               1                    23
cel-miR-124    UAAGGCACGCGGUGAAUGCCA--
dme-miR-124    UAAGGCACGCGGUGAAUGCCAAG
hsa-miR-124    UAAGGCACGCGGUGAAUGCC---
```

```
               1       10
cel-miR-124    UAAGGCACGC
dme-miR-124    UAAGGCACGC
hsa-miR-124    UAAGGCACGC
hsa-miR-506    UAAGGCACCC
```

cel-miR-228:

```
                 1                        26
 cel-miR-228    ---AAUGGCACUGCAUGAAUUCACGG
dme-miR-263a    GUUAAUGGCACUGGAAGAAUUCAC--
 hsa-miR-183    ---UAUGGCACUGGUAGAAUUCACU-
```

```
                1
 cel-miR-228   AAUGGCACUG--
 hsa-miR-183   UAUGGCACUG--
 cel-miR-790   CUUGGCACUC--
 cel-miR-791   UUUGGCACUC--
  hsa-miR-96   UUUGGCACUA--
dme-miR-263b   CUUGGCACUG--
 hsa-miR-514   AUUGACACUU--
 cel-miR-229   AAUGACACUG--
  cel-miR-63   UAUGACACUG--
  cel-miR-64   UAUGACACUG--
  cel-miR-65   UAUGACACUG--
  cel-miR-66   CAUGACACUG--
hsa-miR-200a   --UAACACUGUC
```

```
                 1
 cel-miR-228    ---AAUGGCACUG
dme-miR-263a    GUUAAUGGCA---
```

cel-miR-229:

```
                1
 cel-miR-229   AAUGACACUG--
  cel-miR-63   UAUGACACUG--
  cel-miR-64   UAUGACACUG--
  cel-miR-65   UAUGACACUG--
  cel-miR-66   CAUGACACUG--
hsa-miR-200a   --UAACACUGUC
 hsa-miR-183   UAUGGCACUG--
 cel-miR-790   CUUGGCACUC--
 cel-miR-791   UUUGGCACUC--
 cel-miR-228   AAUGGCACUG--
  hsa-miR-96   UUUGGCACUA--
dme-miR-263b   CUUGGCACUG--
 hsa-miR-514   AUUGACACUU--
```

```
                 1
 cel-miR-229    ---AAUGACACUG
dme-miR-263a    GUUAAUGGCA---
```

cel-miR-231:

```
                1
  cel-miR-231  --UAAGCUCGUG
  cel-miR-787  --UAAGCUCGUU
  dme-miR-993  --GAAGCUCGUC
 hsa-miR-99a*  --CAAGCUCGCU
 hsa-miR-99b*  --CAAGCUCGUG
hsa-miR-556-5p GAUGAGCUCA--
```

cel-miR-232:

```
                  1
     cel-miR-232  -UAAAUGCAUC
     cel-miR-256  UGGAAUGCAU-
     cel-miR-357  -UAAAUGCCAG
     dme-miR-277  -UAAAUGCACU
    hsa-miR-302a  -UAAGUGCUUC
    hsa-miR-302b  -UAAGUGCUUC
    hsa-miR-302c  -UAAGUGCUUC
    hsa-miR-302d  -UAAGUGCUUC
    hsa-miR-519a  -AAAGUGCAUC
hsa-miR-519b-3p   -AAAGUGCAUC
hsa-miR-519c-3p   -AAAGUGCAUC
```

cel-miR-234:

```
               1                     23
cel-miR-234    UUAUUGCUCGAGAAUACCCUU--
dme-miR-137    -UAUUGCUUGAGAAUACACGUAG
hsa-miR-137    UUAUUGCUUAAGAAUACGCGUAG
```

```
                1
 cel-miR-234   --UUAUUGCUCG-
 dme-miR-137   ---UAUUGCUUGA
hsa-miR-126*   CAUUAUUACU---
 hsa-miR-137   --UUAUUGCUUA-
```

cel-miR-235:

```
                 1                    22
cel-miR-235      UAUUGCACUCUCCCCGGCCUGA
dme-miR-311      UAUUGCACAUUCACCGGCCUGA
dme-miR-310      UAUUGCACACUUCCCGGCCUUU
dme-miR-312      UAUUGCACUUGAGACGGCCUGA
dme-miR-313      UAUUGCACUUUUCACAGCCCGA
 hsa-miR-25      CAUUGCACUUGUCUCGGUCUGA
dme-miR-92a      CAUUGCACUUGUCCCGGCCUAU
hsa-miR-92a      UAUUGCACUUGUCCCGGCCUGU
dme-miR-92b      AAUUGCACUAGUCCCGGCCUGC
hsa-miR-92b      UAUUGCACUCGUCCCGGCCUCC
```

```
                  1
   cel-miR-235    --UAUUGCACUC
   dme-miR-310    --UAUUGCACAC
   dme-miR-311    --UAUUGCACAU
   dme-miR-312    --UAUUGCACUU
   dme-miR-313    --UAUUGCACUU
   dme-miR-92a    --CAUUGCACUU
   dme-miR-92b    --AAUUGCACUA
    hsa-miR-25    --CAUUGCACUU
    hsa-miR-32    --UAUUGCACAU
   hsa-miR-363    --AAUUGCACGG
   hsa-miR-367    --AAUUGCACUU
hsa-miR-885-5p    UCCAUUACAC--
   hsa-miR-92a    --UAUUGCACUU
   hsa-miR-92b    --UAUUGCACUC
```

cel-miR-236:

```
                 1                     23
 cel-miR-236     UAAUACUGUCAGGUAAUGACGCU
hsa-miR-200b     UAAUACUGCCUGGUAAUGAUGA-
hsa-miR-200c     UAAUACUGCCGGGUAAUGAUGGA
 hsa-miR-141     UAACACUGUCUGGUAAAGAUGG-
hsa-miR-200a     UAACACUGUCUGGUAACGAUGU-
   dme-miR-8     UAAUACUGUCAGGUAAAGAUGUC
 hsa-miR-429     UAAUACUGUCUGGUAAAACCGU-
```

```
                 1        10
 cel-miR-236     UAAUACUGUC
   dme-miR-8     UAAUACUGUC
hsa-miR-200b     UAAUACUGCC
hsa-miR-200c     UAAUACUGCC
 hsa-miR-429     UAAUACUGUC
```

cel-miR-237:

```
                  1
    cel-miR-237   -UCCCUGAGAA
      cel-lin-4   -UCCCUGAGAC
    dme-miR-125   -UCCCUGAGAC
hsa-miR-125a-5p   -UCCCUGAGAC
   hsa-miR-125b   -UCCCUGAGAC
 hsa-miR-331-3p   GCCCCUGGGC-
```

cel-miR-240:

```
                  1        10
    cel-miR-240   UACUGGCCCC
    dme-miR-193   UACUGGCCUA
hsa-miR-193a-3p   AACUGGCCUA
   hsa-miR-193b   AACUGGCCCU
```

cel-miR-241:

```
                 1
cel-miR-241     -UGAGGUAGGU
  cel-let-7     -UGAGGUAGUA
 cel-miR-48     -UGAGGUAGGC
cel-miR-793     -UGAGGUAUCU
cel-miR-794     -UGAGGUAAUC
cel-miR-795     -UGAGGUAGAU
 cel-miR-84     -UGAGGUAGUA
  dme-let-7     -UGAGGUAGUA
dme-miR-963     ACAAGGUAAA-
dme-miR-977     -UGAGAUAUUC
dme-miR-984     -UGAGGUAAAU
 hsa-let-7a     -UGAGGUAGUA
 hsa-let-7b     -UGAGGUAGUA
 hsa-let-7c     -UGAGGUAGUA
 hsa-let-7d     -AGAGGUAGUA
 hsa-let-7e     -UGAGGUAGGA
 hsa-let-7f     -UGAGGUAGUA
 hsa-let-7g     -UGAGGUAGUA
 hsa-let-7i     -UGAGGUAGUA
 hsa-miR-98     -UGAGGUAGUA
```

cel-miR-244:

```
                1       10
cel-miR-244     UCUUUGGUUG
 dme-miR-9c     UCUUUGGUAU
 dme-miR-9b     UCUUUGGUGA
 dme-miR-9a     UCUUUGGUUA
  hsa-miR-9     UCUUUGGUUA
```

cel-miR-245:

```
                 1                    23
 cel-miR-245     AUUGGUCCCCUCCAAGUAGCUC-
 dme-miR-133     -UUGGUCCCCUUCAACCAGCUGU
hsa-miR-133a     UUUGGUCCCCUUCAACCAGCUG-
hsa-miR-133b     UUUGGUCCCCUUCAACCAGCUA-
```

```
                1
 cel-miR-245    AUUGGUCCCC-
 dme-miR-133    -UUGGUCCCCU
hsa-miR-133a    UUUGGUCCCC-
hsa-miR-133b    UUUGGUCCCC-
```

cel-miR-247:

```
                1
 cel-miR-247    --UGACUAGAGC
  cel-miR-61    --UGACUAGAAC
  cel-miR-44    --UGACUAGAGA
  cel-miR-45    --UGACUAGAGA
 dme-miR-279    --UGACUAGAUC
 dme-miR-286    --UGACUAGACC
 dme-miR-996    --UGACUAGAUU
 hsa-miR-134    UGUGACUGGU--
hsa-miR-708*    --CAACUAGACU
```

cel-miR-250:

```
                  1
   cel-miR-250    --AAUCACAGUC--
     cel-miR-2    --UAUCACAGCC--
    cel-miR-43    --UAUCACAGUU--
   cel-miR-797    --UAUCACAGCA--
    dme-miR-11    --CAUCACAGUC--
   dme-miR-13a    --UAUCACAGCC--
   dme-miR-13b    --UAUCACAGCC--
    dme-miR-2a    --UAUCACAGCC--
    dme-miR-2b    --UAUCACAGCC--
    dme-miR-2c    --UAUCACAGCC--
   dme-miR-308    --AAUCACAGGA--
     dme-miR-6    --UAUCACAGUG--
   hsa-miR-128    ----UCACAGUGAA
   hsa-miR-27a    ---UUCACAGUGG-
   hsa-miR-27b    ---UUCACAGUGG-
hsa-miR-499-3p    AACAUCACAG----
hsa-miR-768-3p    ----UCACAAUGCU
```

cel-miR-251:

```
                1
 cel-miR-251    -UUAAGUAGUG
 cel-miR-252    -AUAAGUAGUA
dme-miR-1002    -UUAAGUAGUG
 hsa-miR-26a    UUCAAGUAAU-
 hsa-miR-26b    UUCAAGUAAU-
```

cel-miR-252:

```
                1
 cel-miR-252    -AUAAGUAGUA
 cel-miR-251    -UUAAGUAGUG
dme-miR-1002    -UUAAGUAGUG
 hsa-miR-26a    UUCAAGUAAU-
 hsa-miR-26b    UUCAAGUAAU-
```

cel-miR-256:

```
               1                     22
 cel-miR-256   UGGAAUGCAUAGAAGACUGUA-
   cel-miR-1   UGGAAUGUAAAGAAGUAUGUA-
   dme-miR-1   UGGAAUGUAAAGAAGUAUGGAG
   hsa-miR-1   UGGAAUGUAAAGAAGUAUGUAU
```

```
                  1
    cel-miR-256   UGGAAUGCAU-
      cel-miR-1   UGGAAUGUAA-
    cel-miR-796   UGGAAUGUAG-
      dme-miR-1   UGGAAUGUAA-
      hsa-miR-1   UGGAAUGUAA-
    hsa-miR-206   UGGAAUGUAA-
    hsa-miR-122   UGGAGUGUGA-
    cel-miR-232   -UAAAUGCAUC
    dme-miR-277   -UAAAUGCACU
   hsa-miR-519a   -AAAGUGCAUC
hsa-miR-519b-3p   -AAAGUGCAUC
hsa-miR-519c-3p   -AAAGUGCAUC
```

cel-miR-259:

```
                1       10
 cel-miR-259    AAAUCUCAUC
 dme-miR-304    UAAUCUCAAU
hsa-miR-216a    UAAUCUCAGC
hsa-miR-216b    AAAUCUCUGC
```

cel-miR-266:

```
               1
cel-miR-266    AGGCAAGACU-
cel-miR-269    -GGCAAGACUC
cel-miR-268    -GGCAAGAAUU
 cel-miR-74    UGGCAAGAAA-
 cel-miR-72    AGGCAAGAUG-
 hsa-miR-31    AGGCAAGAUG-
 cel-miR-73    UGGCAAGAUG-
dme-miR-31a    UGGCAAGAUG-
dme-miR-31b    UGGCAAGAUG-
```

cel-miR-267:

```
              1
cel-miR-267   --CCCGUGAAGU
 cel-miR-51   UACCCGUAGC--
 cel-miR-54   UACCCGUAAU--
 cel-miR-56   UACCCGUAAU--
dme-miR-100   AACCCGUAAA--
hsa-miR-100   AACCCGUAGA--
hsa-miR-99a   AACCCGUAGA--
hsa-miR-99b   CACCCGUAGA--
```

cel-miR-268:

```
               1
cel-miR-266    AGGCAAGACU--
cel-miR-269    -GGCAAGACUC-
cel-miR-268    -GGCAAGAAUU-
 cel-miR-74    UGGCAAGAAA--
hsa-miR-873    --GCAGGAACUU
 cel-miR-72    AGGCAAGAUG--
 hsa-miR-31    AGGCAAGAUG--
 cel-miR-73    UGGCAAGAUG--
dme-miR-31a    UGGCAAGAUG--
dme-miR-31b    UGGCAAGAUG--
```

cel-miR-269:

```
               1
cel-miR-266    AGGCAAGACU-
cel-miR-269    -GGCAAGACUC
cel-miR-268    -GGCAAGAAUU
 cel-miR-74    UGGCAAGAAA-
 cel-miR-72    AGGCAAGAUG-
 hsa-miR-31    AGGCAAGAUG-
 cel-miR-73    UGGCAAGAUG-
dme-miR-31a    UGGCAAGAUG-
dme-miR-31b    UGGCAAGAUG-
```

cel-miR-273:

```
               1       10
cel-miR-273    UGCCCGUACU
 cel-miR-54    UACCCGUAAU
 cel-miR-56    UACCCGUAAU
 cel-miR-51    UACCCGUAGC
 cel-miR-55    UACCCGUAUA
 cel-miR-52    CACCCGUACA
 cel-miR-53    CACCCGUACA
hsa-miR-99b    CACCCGUAGA
dme-miR-100    AACCCGUAAA
hsa-miR-100    AACCCGUAGA
hsa-miR-99a    AACCCGUAGA
```

cel-miR-357:

```
                1       10
 cel-miR-357    UAAAUGCCAG
 dme-miR-277    UAAAUGCACU
 cel-miR-232    UAAAUGCAUC
hsa-miR-302a    UAAGUGCUUC
hsa-miR-302b    UAAGUGCUUC
hsa-miR-302c    UAAGUGCUUC
hsa-miR-302d    UAAGUGCUUC
```

cel-miR-785:

```
                1
 cel-miR-785    -UAAGUGAAUU
  cel-miR-86    -UAAGUGAAUG
 dme-miR-987    UAAAGUAAAU-
hsa-miR-545*    -UCAGUAAAUG
 hsa-miR-559    UAAAGUAAAU-
```

cel-miR-787:

```
                  1
   cel-miR-787    --UAAGCUCGUU
   cel-miR-231    --UAAGCUCGUG
   dme-miR-993    --GAAGCUCGUC
hsa-miR-556-5p    GAUGAGCUCA--
  hsa-miR-99a*    --CAAGCUCGCU
  hsa-miR-99b*    --CAAGCUCGUG
```

cel-miR-790:

```
                 1
  cel-miR-790    CUUGGCACUC--
  cel-miR-228    AAUGGCACUG--
  cel-miR-229    AAUGACACUG--
   cel-miR-63    UAUGACACUG--
   cel-miR-64    UAUGACACUG--
   cel-miR-65    UAUGACACUG--
   cel-miR-66    CAUGACACUG--
  cel-miR-791    UUUGGCACUC--
dme-miR-263b     CUUGGCACUG--
  hsa-miR-183    UAUGGCACUG--
hsa-miR-200a     --UAACACUGUC
  hsa-miR-514    AUUGACACUU--
   hsa-miR-96    UUUGGCACUA--
```

cel-miR-791:

```
                 1
  cel-miR-791    UUUGGCACUC--
  cel-miR-228    AAUGGCACUG--
  cel-miR-229    AAUGACACUG--
   cel-miR-63    UAUGACACUG--
   cel-miR-64    UAUGACACUG--
   cel-miR-65    UAUGACACUG--
   cel-miR-66    CAUGACACUG--
  hsa-miR-514    AUUGACACUU--
hsa-miR-200a     --UAACACUGUC
  hsa-miR-183    UAUGGCACUG--
dme-miR-263b     CUUGGCACUG--
   hsa-miR-96    UUUGGCACUA--
  hsa-miR-182    UUUGGCAAUG--
  cel-miR-790    CUUGGCACUC--
```

cel-miR-793:

```
                1        10
cel-miR-793     UGAGGUAUCU
  cel-let-7     UGAGGUAGUA
cel-miR-241     UGAGGUAGGU
 cel-miR-48     UGAGGUAGGC
cel-miR-794     UGAGGUAAUC
cel-miR-795     UGAGGUAGAU
 cel-miR-84     UGAGGUAGUA
  dme-let-7     UGAGGUAGUA
dme-miR-977     UGAGAUAUUC
dme-miR-984     UGAGGUAAAU
 hsa-let-7a     UGAGGUAGUA
 hsa-let-7b     UGAGGUAGUA
 hsa-let-7c     UGAGGUAGUA
 hsa-let-7e     UGAGGUAGGA
 hsa-let-7f     UGAGGUAGUA
 hsa-let-7g     UGAGGUAGUA
 hsa-let-7i     UGAGGUAGUA
hsa-miR-202     AGAGGUAUAG
 hsa-miR-98     UGAGGUAGUA
```

cel-miR-794:

```
                 1
cel-miR-794     -UGAGGUA-AUC
  cel-let-7     -UGAGGUAGUA-
cel-miR-241     -UGAGGUAGGU-
 cel-miR-48     -UGAGGUAGGC-
cel-miR-793     -UGAGGUAUCU-
cel-miR-795     -UGAGGUAGAU-
 cel-miR-84     -UGAGGUAGUA-
  dme-let-7     -UGAGGUAGUA-
dme-miR-963     ACAAGGUAAA--
dme-miR-977     -UGAGAUAUUC-
dme-miR-984     -UGAGGUAAAU-
 hsa-let-7a     -UGAGGUAGUA-
 hsa-let-7b     -UGAGGUAGUA-
 hsa-let-7c     -UGAGGUAGUA-
 hsa-let-7d     -AGAGGUAGUA-
 hsa-let-7e     -UGAGGUAGGA-
 hsa-let-7f     -UGAGGUAGUA-
 hsa-let-7g     -UGAGGUAGUA-
 hsa-let-7i     -UGAGGUAGUA-
hsa-miR-196a    --UAGGUAGUUU
 hsa-miR-98     -UGAGGUAGUA-
```

cel-miR-795:

```
                 1
cel-miR-795     -UGAGGUAGAU-
cel-miR-241     -UGAGGUAGGU-
 cel-miR-48     -UGAGGUAGGC-
  cel-let-7     -UGAGGUAGUA-
 cel-miR-84     -UGAGGUAGUA-
  dme-let-7     -UGAGGUAGUA-
 hsa-let-7a     -UGAGGUAGUA-
 hsa-let-7b     -UGAGGUAGUA-
 hsa-let-7c     -UGAGGUAGUA-
 hsa-let-7d     -AGAGGUAGUA-
 hsa-let-7e     -UGAGGUAGGA-
 hsa-let-7f     -UGAGGUAGUA-
cel-miR-793     -UGAGGUAUCU-
 hsa-let-7g     -UGAGGUAGUA-
 hsa-let-7i     -UGAGGUAGUA-
cel-miR-794     -UGAGGUA-AUC
 hsa-miR-98     -UGAGGUAGUA-
dme-miR-984     -UGAGGUAAAU-
dme-miR-963     ACAAGGUAAA--
dme-miR-977     -UGAGAUAUUC-
```

cel-miR-796:

```
                1        10
  cel-miR-1     UGGAAUGUAA
cel-miR-256     UGGAAUGCAU
cel-miR-796     UGGAAUGUAG
  dme-miR-1     UGGAAUGUAA
  hsa-miR-1     UGGAAUGUAA
hsa-miR-122     UGGAGUGUGA
hsa-miR-206     UGGAAUGUAA
```

cel-miR-797:

```
                1
   cel-miR-797  --UAUCACAGCA
   cel-miR-250  --AAUCACAGUC
   dme-miR-308  --AAUCACAGGA
    dme-miR-11  --CAUCACAGUC
hsa-miR-499-3p  AACAUCACAG--
    cel-miR-43  --UAUCACAGUU
     dme-miR-6  --UAUCACAGUG
     cel-miR-2  --UAUCACAGCC
   dme-miR-13a  --UAUCACAGCC
   dme-miR-13b  --UAUCACAGCC
    dme-miR-2a  --UAUCACAGCC
    dme-miR-2b  --UAUCACAGCC
    dme-miR-2c  --UAUCACAGCC
```

cel-miR-1018:

```
                  1
   cel-miR-1018   AGAGAGAUCA---
     cel-miR-81   --UGAGAUCAUC-
     cel-miR-82   --UGAGAUCAUC-
     cel-miR-80   --UGAGAUCAUU-
     cel-miR-58   --UGAGAUCGUU-
     dme-bantam   --UGAGAUCAUU-
   cel-miR-1022   ---AAGAUCAUUG
hsa-miR-450b-3p   -UUGGGAUCAU--
```

cel-miR-1022:

```
                  1
   cel-miR-1022   ---AAGAUCAUUG
   cel-miR-1018   AGAGAGAUCA---
     cel-miR-81   --UGAGAUCAUC-
     cel-miR-82   --UGAGAUCAUC-
   dme-miR-306*   --GGGGGUCACU-
hsa-miR-450b-3p   -UUGGGAUCAU--
     cel-miR-80   --UGAGAUCAUU-
     cel-miR-58   --UGAGAUCGUU-
     dme-bantam   --UGAGAUCAUU-
```
