# Supplementary figures and images for: Sequence Relationships among C. elegans, D. melanogaster and Human microRNAs Highlight the Extensive Conservation of microRNAs in Biology
Source: PLoS One. 2008 Jul 30;3(7):e2818. doi: 10.1371/journal.pone.0002818 (PMC2486268; doi:10.1371/journal.pone.0002818)

**A**

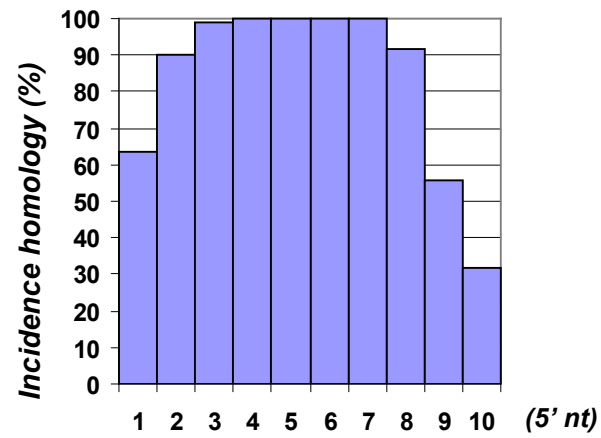

**B**

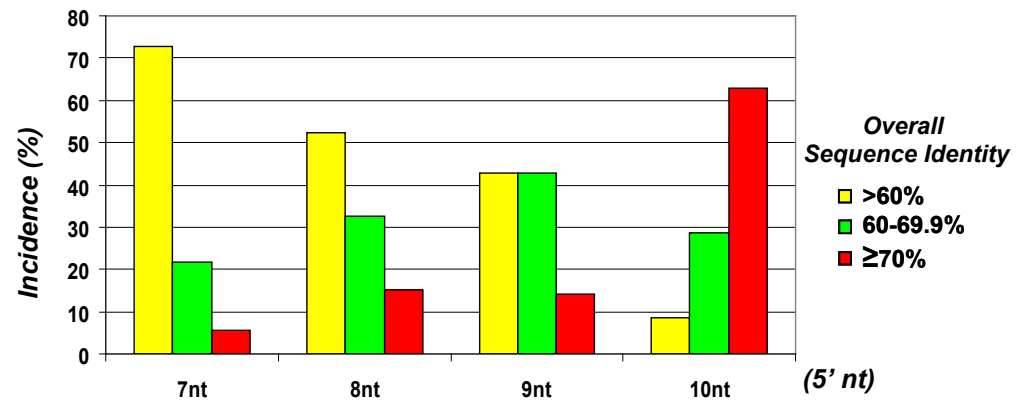

Supplement: Figure S2 — Frequency and distribution of 5′ homologous nucleotides and their correlation with overall sequence conservation. A: Analysis of all 5′ sequence-related miRNAs in C. elegans indicates that homologous nucleotides are mainly positioned from nucleotides 2 to 8 (Dataset S1). B: Sequence-related miRNAs with 7 or 8 homologous nucleotides at the 5′ end tend to have poorer sequence similarity at the 3′ end and thus weaker overall similarity than related miRNAs with 9 or 10 5′ nucleotide homologies. miRNAs with 10 5′ homologous nucleotides tend to have significant nucleotide similarities at the 3′ end with an overall sequence identity of 70–100% (Table 1, Dataset S2) or less frequently of 60–69.9% (Dataset S3). (0.32 MB PDF) [file pone.0002818.s002.pdf]

**A**

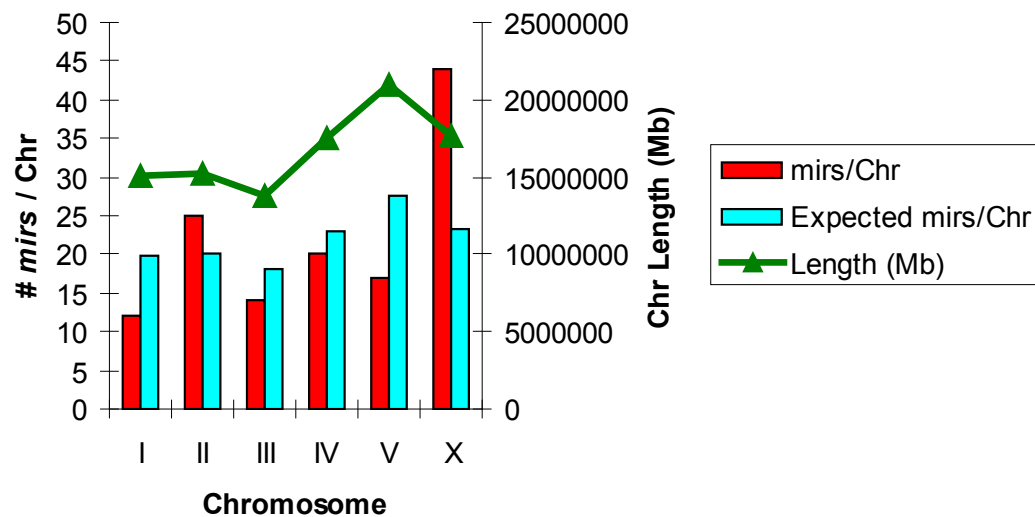

**B**

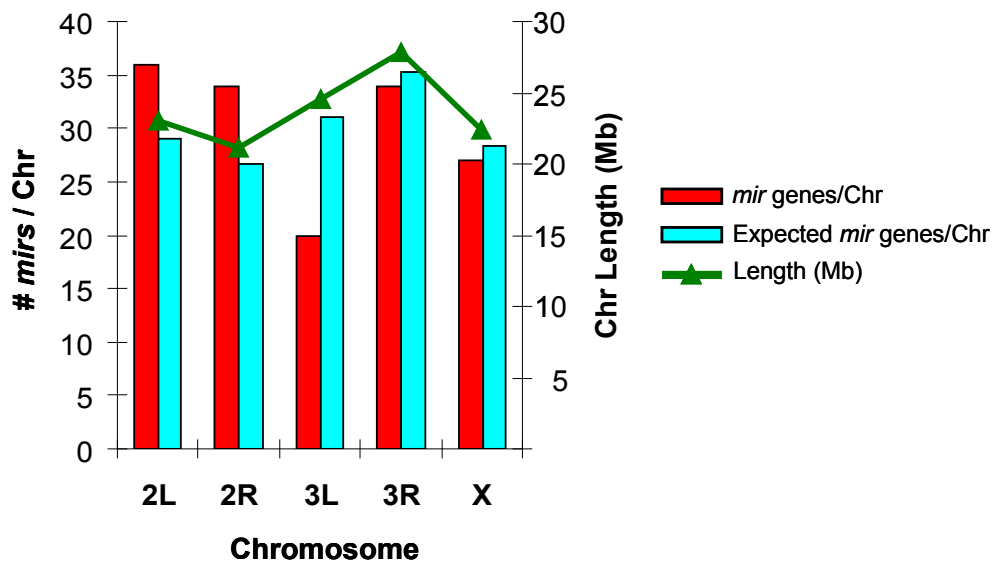

Supplement: Figure S4 — Average distribution of mir genes in the C. elegans and D. melanogaster genomes. Despite additional mir genes might still be discovered further concentrating genetic maps, it is worthy of note that a higher proportion of mir genes in miRBase 10.1 are located in C. elegans chromosome X (A) and in Drosophila chromosome pair 2L, 2R (B) than expected by random distribution. The expected number of mir genes was determined by dividing total number of mirs in the genome by chromosome length. (0.39 MB PDF) [file pone.0002818.s004.pdf]
