# Supplementary material for: Sequence Relationships among C. elegans, D. melanogaster and Human microRNAs Highlight the Extensive Conservation of microRNAs in Biology
Source: PLoS One. 2008 Jul 30;3(7):e2818. doi: 10.1371/journal.pone.0002818 (PMC2486268; doi:10.1371/journal.pone.0002818)
Supplement: Figure S3 — Sequence alignments of C. elegans miRNAs with extensive similarity at the 3′ end but poor homology at the 5′ end. C. elegans mature miRNAs vary in length from 18 nt to 26 nt, and thus the 3′ end sequences used differed in length to some extent. Since the majority of C. elegans miRNAs are 21–23 nt long and on average 22 nt, most of the 3′ end sequences varied 1–2 nt in size. Grey shading denotes potential G..U pairing. (0.02 MB PDF) [file pone.0002818.s003.pdf]

cel-lin-4, cel-miR-87:

```
              1       10          22
  cel-lin-4   -UCCCUGAGACCUCAAGUGUGA
cel-miR-87    GUGAGCAAAGUUUCAGGUGUGC   45.5%

                18.2%       66.7%
```

cel-miR-90, cel-miR-124:

```
              1       10           23
cel-miR-124   UAAGGCACGCGGUGAAUGCCA--
 cel-miR-90   UGAUAUGUUGUUUGAAUGCCCCU  43.5%

                 20%        61.5%
```

It should be noted that the cel-miR-90_cel-miR-124 3’ block of identity is conserved to some extent in miRNAs cel-miR-80, cel-miR-81, cel-miR-82 and cel-miR-234.
Alignment of 3’ end sequences:

```
              1           14
cel-miR-124   -GGUGAAUGCCA--
cel-miR-234   ---AGAAUACCCUU
 cel-miR-90   -UUUGAAUGCCCCU
 cel-miR-80   AGUUGAAAGCCGA-
 cel-miR-81   --GUGAAAGCUAGU
 cel-miR-82   --GUGAAAGCCAGU
```

Interestingly the human miRNA hsa-miR-124, counterpart of *C. elegans* cel-miR-124, has extensive 3’ sequence similarity to hsa-miR-377*.

```
               1                   23
 hsa-miR-124   UAAGGCACGC---GGUGAAUGCC
hsa-miR-377*   AGAGGUUGCCCUUGGUGAAUUC-   52.2%

                  40%        61.5%
```

cel-miR-81, cel-miR-799:

```
               1        10            24
cel-miR-799    --UGAACCCUGAUAAAGCUAGUGG
 cel-miR-81    UGAGAUCAUCGUGAAAGCUAGU--  54.2%

                 36.3%         64.3%
```

It should be noted that the cel-miR-799_cel-miR-81 3’ block of identity is conserved to some extent in *C. elegans* miRNAs cel-miR-80 and cel-miR-82, and in human miRNA hsa-miR-208a.
Alignment of 3’ end sequences:

```
              1             16
cel-miR-799   ----UAAAGCUAGUGG
 cel-miR-81   --GUGAAAGCUAGU--
 cel-miR-80   AGUUGAAAGCCGA---
 cel-miR-82   --GUGAAAGCCAGU--

             1           14
cel-miR-799   --UAAAGCUAGUGG
 cel-miR-81   GUGAAAGCUAGU--
hsa-miR-208a  CAAAAAGCUUGU—
```

In turn, human miRNA hsa-miR-208a is 3’-related to human miRNAs hsa-miR-129-3p and hsa-miR-129*.

```
                  1        10                26
hsa-miR-129-3p    --AAGCCCUUACCCCAAAAAGCAU--
   hsa-miR-208a   AUAAGACGAG----CAAAAAGCUUGU  50%

                  [illegible]    [illegible]

                 1        10                26
 hsa-miR-208a    AUAAGACGAG----CAAAAAGCUUGU
hsa-miR-129*     --AAGCCCUUACCCCAAAAAGUAU--  46.2%

                    33.3%        57.1%
```

cel-miR-70, cel-miR-52, cel-miR-53, cel-miR-229 and cel-miR-272:

```
              1        10             26
cel-miR-70    UAAUACGUCGUUGGUGUUUCCAU---
cel-miR-52    -CACCCGUACAUA-UGUUUCCGUGCU  50.0%
               36.4%          50%
```

```
              1        10             26
cel-miR-70    UAAUACGUCGUUGGUGUUUCCAU---
cel-miR-53    -CACCCGUACAUU-UGUUUCCGUGCU  50.0%
               36.4%          50%
```

```
               1         10            27
cel-miR-70     --UAAUACGUCGUUGGUGUUUCCAU--
cel-miR-229    AAUGACAC-UGGUUAUCUUUUCCAUCG  55.6%
                 54.5%          56.3%
```

```
               1        10         23
cel-miR-70     UAAUACGUCGUUGGUGUUUCCAU
cel-miR-272    -UGUAGG-CAUGGGUGUUUG---   52.2%
                 27.3%      53.8%
```
