# Supplementary material for: Sequence Relationships among C. elegans, D. melanogaster and Human microRNAs Highlight the Extensive Conservation of microRNAs in Biology
Source: PLoS One. 2008 Jul 30;3(7):e2818. doi: 10.1371/journal.pone.0002818 (PMC2486268; doi:10.1371/journal.pone.0002818)
Supplement: Dataset S1 — Homology table and sequence alignments of C. elegans miRNAs with significant identity at the 5′ 10 nt. (0.39 MB DOC) [file pone.0002818.s005.doc]

**Supplementary Table S1: Analysis of 5' end sequence conservation identifies 81 *C. elegans* miRNAs that define 19 different families.** Superscript “less than” symbol (**<**) before miRNA names indicates that A-G base changes (G..U pairing) interrupt the 5’ 7nt homology blocks grouping specific miRNAs into families (see alignments below). Sequence similarities grouping miRNAs into families are summarized in the table and detailed in alignments. **(5’)** identifies 77 miRNAs with high identity at the 5’ end but <70% overall similarity to some of their sequence-related miRNAs. Of these, 21 miRNAs (cel-lin-4, cel-miR-49, cel-miR-50, cel-miR-62, cel-miR-75, cel-miR-79, cel-miR-83, cel-miR-90, cel-miR-228, cel-miR-229, cel-miR-231, cel-miR-232, cel-miR-237, cel-miR-238, cel-miR-356, cel-miR-357, cel-miR-360, cel-miR-787, cel-miR-795, cel-miR-797 and cel-miR-1018) are significantly related in sequence only at the 5’ end—they do not have ≥60% similarity over their full sequence with their 5’ related miRNAs and therefore are not included in Datasets S2 and S3.

**?** = Unknown function.

| **miRNA**  **Group ID** | **Family Members** | **Sub-groups** | **Functional Information** |
| --- | --- | --- | --- |
| **let-7**  *UGAGGUA* | cel-let-7 **(5’)** | cel-let-7  cel-miR-84 | Cell fate in L4-Adult transition [1]; L2-Adult expression [2,3]; Regulated during adult aging [4] |
|  | cel-miR-48 **(5’)** | cel-miR-48 cel-miR-241 cel-miR-795 | Cell fate in larval transitions [3]; Embryonic and post-embryonic expression enhanced at L3 stage [2,3] |
|  | cel-miR-84 **(5’)** |  | Cell fate in larval transitions and vulva morphogenesis [3,5]; Post-embryonic expression enhanced at L3 stage [2,3] |
|  | cel-miR-241 **(5’)** |  | Cell fate in larval transitions [3]; L2-Adult expression enhanced at L3 stage [2,3]; Regulated during adult aging [4] |
|  | cel-miR-793 **(5’)** |  | **?** |
|  | cel-miR-794 **(5’)** |  | **?** |
|  | cel-miR-795 **(5’)** |  | **?** |
| **lin-4**  *UCCCUGA* | cel-lin-4 **(5’)** |  | L1-L2 developmental transition [6]; Aging [7]; L2-Adult expression [6,8]; Regulated during adult aging [4] |
|  | cel-miR-237 **(5’)** |  | L2-Adult expression enhanced at L3 and L4 stages [2] |
| **miR-2**  *UAUCACA* | cel-miR-2 **(5’)** | cel-miR-2  cel-miR-43 cel-miR-797 | Expressed throughout development [9]; Regulated during adult aging [4] |
|  | cel-miR-43 **(5’)** |  | Larval and predominantly embryonic expression [8,9]; Regulated during adult aging [4] |
|  | cel-miR-250 **(5’)** |  | Expressed throughout development [9] |
|  | cel-miR-797 **(5’)** |  | ? |
| **miR-35**  *UCA/GCCGG* | cel-miR-35 **(5’)** | cel-miR-35 cel-miR-36 cel-miR-37 cel-miR-39 cel-miR-40 cel-miR-41 | Embryonic and adult expression [8,9]; Regulated during adult aging [4] |
|  | cel-miR-36 **(5’)** |  | Embryonic and adult expression [8,9]; Regulated during adult aging [4] |
|  | cel-miR-37 **(5’)** |  | Embryonic and adult expression [8,9]; Regulated during adult aging [4] |
|  | cel-miR-38 **(5’)** |  | Embryonic and adult expression [8,9]; Regulated during adult aging [4] |
|  | cel-miR-39 **(5’)** |  | Embryonic and adult expression [8,9]; Regulated during adult aging [4] |
|  | cel-miR-40 **(5’)** |  | Embryonic and adult expression [8,9] |
|  | cel-miR-41 **(5’)** |  | Regulated during adult aging [4] |
|  | cel-miR-42 **(5’)** |  | Larval and predominant embryonic expression [8,9]; Regulated during adult aging [4] |
|  | cel-miR-271 **(5’)** |  | **?** |
| **miR-44**  *UGACUAG* | cel-miR-44 **(5’)** | cel-miR-44 cel-miR-45 | Expressed throughout development [8,9] |
|  | cel-miR-45 **(5’)** |  | Expressed throughout development [8,9]; Regulated during adult aging [4] |
|  | cel-miR-61 **(5’)** |  | Vulval morphogenesis [10]; Expressed throughout development [9] |
|  | cel-miR-247 **(5’)** |  | Expression at L3 stage and Enhanced at dauer stage [9] |
| **miR-46**  *UGUCAUG* | cel-miR-46 |  | Expressed throughout development [8,9]; Regulated during adult aging [4] |
|  | cel-miR-47 |  | Expressed throughout development [8,9]; Regulated during adult aging [4] |
| **miR-49**  *AGCACCA* | cel-miR-49 **(5’)** |  | Expressed throughout development with predominant expression at embryonic and L1 stages [9] |
|  | cel-miR-83 **(5’)** |  | Expressed throughout development [9] |
| **miR-50**  *UGAUAUG* | cel-miR-50 **(5’)** |  | Expressed throughout development [9]; Regulated during adult aging [4] |
|  | cel-miR-62 **(5’)** |  | Expressed throughout development [9] |
|  | cel-miR-90 **(5’)** |  | Expressed throughout development [9] |
| **miR-51**  *A/GCCCGUA* **/**  *(C)CCGUA/GA(U)* | cel-miR-51 **(5’)** |  | Expressed throughout development [9] |
|  | cel-miR-52 **(5’)** | cel-miR-52  cel-miR-53 | Expressed throughout development [8,9] |
|  | cel-miR-53 **(5’)** |  | Expressed throughout development [9] |
|  | cel-miR-54 **(5’)** | cel-miR-54 cel-miR-56 | Expressed throughout development [9]; Regulated during adult aging [4] |
|  | cel-miR-55 **(5’)** |  | Expressed throughout development [9] |
|  | cel-miR-56 **(5’)** |  | Expressed throughout development [8,9]; Regulated during adult aging [4] |
|  | **<**cel-miR-267 **(5’)** |  | **?** |
|  | cel-miR-273 **(5’)** |  | Left/right neuronal asymmetry [11]; Regulated during adult aging [4] |
|  | cel-miR-360 **(5’)** |  | **?** |
| **miR-63**  *UGA/GCACU* | cel-miR-63 **(5’)** | cel-miR-63 cel-miR-64 cel-miR-65 | Expressed throughout development [9]; Regulated during adult aging [4] |
|  | cel-miR-64 **(5’)** |  | Expressed throughout development [8,9]; Regulated during adult aging [4] |
|  | cel-miR-65 **(5’)** |  | Expressed throughout development [8,9]; Regulated during adult aging [4] |
|  | cel-miR-66 **(5’)** |  | Expressed throughout development [8,9] |
|  | cel-miR-228 **(5’)** |  | Expressed throughout development [9]; Regulated during adult aging [4] |
|  | cel-miR-229 **(5’)** |  | Expressed throughout development [9]; Regulated during adult aging [4] |
|  | **<**cel-miR-790 **(5’)** | cel-miR-790 cel-miR-791 | **?** |
|  | cel-miR-791 **(5’)** |  | **?** |
| **miR-75**  *UAAAGCU* | cel-miR-75 **(5’)** |  | Embryonic and strong post-embryonic expression [9] |
|  | cel-miR-79 **(5’)** |  | Expressed throughout development [9] |
| **miR-80**  *G/AAGAUCA/G* | cel-miR-58 **(5’)** | cel-miR-58 cel-miR-80 | Expressed throughout development [9]; Regulated during adult aging [4] |
|  | cel-miR-80 **(5’)** |  | Expressed throughout development [8,9] |
|  | cel-miR-81 **(5’)** | cel-miR-81 cel-miR-82 | Expressed throughout development [9]; Regulated during adult aging [4] |
|  | cel-miR-82 **(5’)** |  | Expressed throughout development [9]; Regulated during adult aging [4] |
|  | cel-miR-1018 **(5’)** |  | **?** |
|  | cel-miR-1022 **(5’)** |  | **?** |
| **miR-86**  *UAAGUGA* | cel-miR-86 **(5’)** |  | Expressed throughout development [9] |
|  | cel-miR-785 **(5’)** |  | **?** |
| **miR-231**  *UAAGCUC* | cel-miR-231 **(5’)** |  | Expression throughout development [9]; Regulated during adult aging [4] |
|  | cel-miR-787 **(5’)** |  | **?** |
| **miR-233**  *UGAGCAA* | cel-miR-87 **(5’)** |  | Expressed throughout development [9] |
|  | cel-miR-233 **(5’)** |  | Expressed throughout development [9]; Regulated during adult aging [4] |
|  | cel-miR-356 **(5’)** |  | **?** |
| **miR-239a**  *UUUGUAC* | cel-miR-238 **(5’)** |  | Embryonic and strong post-embryonic expression [9]; Regulated during adult aging [4] |
|  | cel-miR-239a **(5’)** | cel-miR-239a cel-miR-239b | Embryonic and strong post-embryonic expression [9]; Regulated during adult aging [4] |
|  | cel-miR-239b **(5’)** |  | Embryonic and strong post-embryonic expression [9] |
| **miR-251**  *UAAGUAG* | cel-miR-251 |  | Expressed throughout development [9]; Regulated during adult aging [4] |
|  | cel-miR-252 |  | Expressed throughout development [9] |
| **miR-256**  *UGGAAUG* ***/***  *(UA)AAUGC(AU)* | cel-miR-1 **(5’)** | cel-miR-1  cel-miR-256 cel-miR-796 | Expressed throughout development [8,9]; Regulated during adult aging [4] |
|  | cel-miR-232 **(5’)** | cel-miR-232  cel-miR-357 | Expressed throughout development [9] |
|  | cel-miR-256 **(5’)** |  | **?** |
|  | cel-miR-357 **(5’)** |  | **?** |
|  | cel-miR-796 **(5’)** |  | **?** |
| **miR-266**  *GGCAAGA* | cel-miR-72 **(5’)** | cel-miR-72 cel-miR-73 | Expressed throughout development [9] |
|  | cel-miR-73 **(5’)** |  | Expressed throughout development [9]; Regulated during adult aging [4] |
|  | cel-miR-74 **(5’)** |  | Expressed throughout development [9]; Regulated during adult aging [4] |
|  | cel-miR-266 **(5’)** | cel-miR-266 cel-miR-269 | **?** |
|  | cel-miR-268 **(5’)** |  | Regulated during adult aging [4] |
|  | cel-miR-269 **(5’)** |  | **?** |

**Supplementary Alignments S1:**

**5’ end sequence alignments of *C. elegans* miRNAs with significant homology at the 5’ 10nt.** Members of a group have ≥7 continuous nt of homology with at least one other group member. Nucleotides at the end of sequences indicate number of residues identical to the reference miRNA (top of group alignment), which has the closest sequence to the group consensus sequence. Sub-groups contain miRNAs with most closely similar 5’ end sequences. Grey shading denotes potential G..U pairing. Superscript “less than” symbol (**<**) before miRNA names indicates allowed A-G base changes (G..U pairing) interrupting the 7nt homology block at the 5' end (10nt) which groups miRNAs into families. Superscript (5’) indicates miRNAs with 5’ end identity but weak overall similarity (<70%) to some of their miRNA homologs. Highlighted in blue are 21 miRNAs that show high homology at the 5’ end but low whole sequence similarity (<60%) with all their sequence-related miRNAs.

**let-7: cel-let-7 ^(5’)^, cel-miR-48 ^(5’)^, cel-miR-84 ^(5’)^, cel-miR-241^(5’)^,**

**cel-miR-793 ^(5’)^, cel-miR-794 ^(5’)^, cel-miR-795 ^(5’)^**

1 10

cel-let-7 UGAGGUAGUA

cel-miR-84 UGAGGUAGUA 10nt

cel-miR-794 UGAGGUAAUC 7nt

cel-miR-48 UGAGGUAGGC 8nt

cel-miR-241 UGAGGUAGGU 8nt

cel-miR-793 UGAGGUAUCU 7nt

cel-miR-795 UGAGGUAGAU 8nt

**Sub-groups**

**i. cel-let-7, cel-miR-84:**

1 10

cel-let-7 UGAGGUAGUA

cel-miR-84 UGAGGUAGUA 10nt

**ii. cel-miR-48, cel-miR-241, cel-miR-795:**

1 10

cel-miR-241 UGAGGUAGGU

cel-miR-48 UGAGGUAGGC 9nt

cel-miR-795 UGAGGUAGAU 9nt

**lin-4: cel-lin-4 ^(5’)^, cel-miR-237 ^(5’)^**

1 10

cel-lin-4 UCCCUGAGAC

cel-miR-237 UCCCUGAGAA 9nt

**miR-2: cel-miR-2 ^(5’)^, cel-miR-43 ^(5’)^, cel-miR-250 ^(5’)^,**

**cel-miR-797 ^(5’)^**

1 10

cel-miR-2 UAUCACAGCC

cel-miR-797 UAUCACAGCA 9nt

cel-miR-43 UAUCACAGUU 8nt

cel-miR-250 AAUCACAGUC 8nt

**Sub-group cel-miR-2, cel-miR-43, cel-miR-797:**

1 10

cel-miR-2 UAUCACAGCC

cel-miR-797 UAUCACAGCA 9nt

cel-miR-43 UAUCACAGUU 8nt

**miR-35: cel-miR-35 ^(5’)^, cel-miR-36 ^(5’)^, cel-miR-37 ^(5’)^,**

**cel-miR-38 ^(5’)^, cel-miR-39 ^(5’)^, cel-miR-40 ^(5’)^,**

**cel-miR-41 ^(5’)^, cel-miR-42 ^(5’)^, cel-miR-271 ^(5’)^**

1 10

cel-miR-35 UCACCGGGUG

cel-miR-36 UCACCGGGUG 10nt

cel-miR-37 UCACCGGGUG 10nt

cel-miR-39 UCACCGGGUG 10nt

cel-miR-40 UCACCGGGUG 10nt

cel-miR-41 UCACCGGGUG 10nt

cel-miR-271 UCGCCGGGUG 9nt

cel-miR-38 UCACCGGGAG 9nt

cel-miR-42 UCACCGGGUU 9nt

**Sub-group cel-miR-35, cel-miR-36, cel-miR-37, cel-miR-39, cel-miR-40, cel-miR-41:**

1 10

cel-miR-35 UCACCGGGUG

cel-miR-36 UCACCGGGUG 10nt

cel-miR-37 UCACCGGGUG 10nt

cel-miR-39 UCACCGGGUG 10nt

cel-miR-40 UCACCGGGUG 10nt

cel-miR-41 UCACCGGGUG 10nt

**miR-44: cel-miR-44 ^(5’)^, cel-miR-45 ^(5’)^, cel-miR-61 ^(5’)^,**

**cel-miR-247 ^(5’)^**

1 10

cel-miR-44 UGACUAGAGA

cel-miR-45 UGACUAGAGA 10nt

cel-miR-247 UGACUAGAGC 9nt

cel-miR-61 UGACUAGAAC 8nt

**Sub-groups**

**i. cel-miR-44, cel-miR-45:**

1 10

cel-miR-44 UGACUAGAGA

cel-miR-45 UGACUAGAGA 10nt

**ii. cel-miR-61, cel-miR-247:**

1 10

cel-miR-247 UGACUAGAGC

cel-miR-61 UGACUAGAAC 10nt

**miR-46: cel-miR-46, cel-miR-47**

1 10

cel-miR-46 UGUCAUGGAG

cel-miR-47 UGUCAUGGAG 10nt

**miR-49: cel-miR-49 ^(5’)^, cel-miR-83 ^(5’)^**

1 10

cel-miR-49 AAGCACCACG

cel-miR-83 UAGCACCAUA 7nt

**miR-50: cel-miR-50 ^(5’)^, cel-miR-62 ^(5’)^, cel-miR-90 ^(5’)^**

1 10

cel-miR-50 UGAUAUGUCU

cel-miR-62 UGAUAUGUAA 8nt

cel-miR-90 UGAUAUGUUG 8nt

**miR-51: cel-miR-51 ^(5’)^, cel-miR-52 ^(5’)^, cel-miR-53 ^(5’)^,**

**cel-miR-54 ^(5’)^, cel-miR-55 ^(5’)^, cel-miR-56 ^(5’)^,**

**<cel-miR-267 ^(5’)^, cel-miR-273 ^(5’)^, cel-miR-360 ^(5’)^**

1

cel-miR-51 UACCCGUAGC--

cel-miR-55 UACCCGUAUA-- 8nt

cel-miR-56 UACCCGUAAU-- 8nt

cel-miR-54 UACCCGUAAU-- 8nt

cel-miR-360 UGACCGUAAU-- 6nt

cel-miR-273 UGCCCGUACU-- 7nt

cel-miR-53 CACCCGUACA-- 7nt

cel-miR-52 CACCCGUACA-- 7nt

cel-miR-267 --CCCGUGAAGU 5nt

**Sub-groups**

**i. cel-miR-52, cel-miR-53:**

1 10

cel-miR-52 CACCCGUACA

cel-miR-53 CACCCGUACA 10nt

**ii. cel-miR-54, cel-miR-56:**

1 10

cel-miR-54 UACCCGUAAU

cel-miR-56 UACCCGUAAU 10nt

**miR-63: cel-miR-63 ^(5’)^, cel-miR-64 ^(5’)^, cel-miR-65 ^(5’)^,**

**cel-miR-66 ^(5’)^, cel-miR-228 ^(5’)^, cel-miR-229 ^(5’)^,**

**<cel-miR-790 ^(5’)^, cel-miR-791 ^(5’)^**

1 10

cel-miR-63 UAUGACACUG

cel-miR-64 UAUGACACUG 10nt

cel-miR-65 UAUGACACUG 10nt

cel-miR-66 CAUGACACUG 9nt

cel-miR-229 AAUGACACUG 9nt

cel-miR-228 AAUGGCACUG 8nt

cel-miR-791 UUUGGCACUC 7nt

cel-miR-790 CUUGGCACUC 6nt

**Sub-group cel-miR-63, cel-miR-64, cel-miR-65:**

1 10

cel-miR-63 UAUGACACUG

cel-miR-64 UAUGACACUG 10nt

cel-miR-65 UAUGACACUG 10nt

**miR-75: cel-miR-75 ^(5’)^, cel-miR-79 ^(5’)^**

1 10

cel-miR-75 UUAAAGCUAC

cel-miR-79 AUAAAGCUAG 8nt

**miR-80: cel-miR-58 ^(5’)^, cel-miR-80 ^(5’)^, cel-miR-81 ^(5’)^,**

**cel-miR-82 ^(5’)^, cel-miR-1018 ^(5’)^, cel-miR-1022 ^(5’)^**

1

cel-miR-80 --UGAGAUCAUU-

cel-miR-58 --UGAGAUCGUU- 9nt

cel-miR-1022 ---AAGAUCAUUG 8nt

cel-miR-81 --UGAGAUCAUC- 9nt

cel-miR-82 --UGAGAUCAUC- 9nt

cel-miR-1018 AGAGAGAUCA--- 7nt

**Sub-group cel-miR-58, cel-miR-80:**

1 10

cel-miR-58 UGAGAUCGUU

cel-miR-80 UGAGAUCAUU 9nt

**Sub-group cel-miR-81, cel-miR-82:**

1 10

cel-miR-81 UGAGAUCAUC

cel-miR-82 UGAGAUCAUC 10nt

**miR-86: cel-miR-86 ^(5’)^, cel-miR-785 ^(5’)^**

1 10

cel-miR-86 UAAGUGAAUG

cel-miR-785 UAAGUGAAUU 9nt

**miR-231: cel-miR-231 ^(5’)^, cel-miR-787 ^(5’)^**

1 10

cel-miR-231 UAAGCUCGUG

cel-miR-787 UAAGCUCGUU 9nt

**miR-233: cel-miR-87 ^(5’)^, cel-miR-233 ^(5’)^, cel-miR-356 ^(5’)^**

1 10

cel-miR-233 UUGAGCAAUG

cel-miR-356 UUGAGCAACG 9nt

cel-miR-87 GUGAGCAAAG 8nt

**miR-239a: cel-miR-238 ^(5’)^, cel-miR-239a ^(5’)^, cel-miR-239b ^(5’)^**

1 10

cel-miR-239a UUUGUACUAC

cel-miR-239b UUUGUACUAC 10nt

cel-miR-238 UUUGUACUCC 9nt

**Sub-group cel-miR-239a, cel-miR-239b:**

1 10

cel-miR-239a UUUGUACUAC

cel-miR-239b UUUGUACUAC 10nt

**miR-251: cel-miR-251, cel-miR-252**

1 10

cel-miR-251 UUAAGUAGUG

cel-miR-252 AUAAGUAGUA 8nt

**miR-256: cel-miR-1 ^(5’)^, cel-miR-232 ^(5’)^, cel-miR-256 ^(5’)^,**

**cel-miR-357 ^(5’)^, cel-miR-796 ^(5’)^**

1

cel-miR-256 UGGAAUGCAU-

cel-miR-1 UGGAAUGUAA- 8nt

cel-miR-796 UGGAAUGUAG- 8nt

cel-miR-232 -UAAAUGCAUC 7nt

cel-miR-357 -UAAAUGCCAG 5nt

**Sub-groups**

**i. cel-miR-1, cel-miR-256, cel-miR-796:**

1 10

cel-miR-1 UGGAAUGUAA

cel-miR-796 UGGAAUGUAG 9nt

cel-miR-256 UGGAAUGCAU 8nt

**ii. cel-miR-232, cel-miR-357:**

1 10

cel-miR-232 UAAAUGCAUC

cel-miR-357 UAAAUGCCAG 7nt

**miR-266: cel-miR-72 ^(5’)^, cel-miR-73 ^(5’)^, cel-miR-74 ^(5’)^,**

**cel-miR-266 ^(5’)^, cel-miR-268 ^(5’)^, cel-miR-269 ^(5’)^**

1

cel-miR-266 AGGCAAGACU-

cel-miR-269 -GGCAAGACUC 9nt

cel-miR-72 AGGCAAGAUG- 7nt

cel-miR-73 UGGCAAGAUG- 7nt

cel-miR-268 -GGCAAGAAUU 8nt

cel-miR-74 UGGCAAGAAA- 7nt

**Sub-groups**

**i. cel-miR-72, cel-miR-73:**

1 10

cel-miR-72 AGGCAAGAUG

cel-miR-73 UGGCAAGAUG 9nt

**ii. cel-miR-266, cel-miR-269:**

1

cel-miR-266 AGGCAAGACU-

cel-miR-269 -GGCAAGACUC 9nt

REFERENCES

1. Reinhart BJ, Slack FJ, Basson M, Pasquinelli AE, Bettinger JC, et al. (2000) The 21-nucleotide *let-7* RNA regulates developmental timing in *Caenorhabditis elegans*. Nature 403: 901-906.

2. Esquela-Kerscher A, Johnson SM, Bai L, Saito K, Partridge J, et al. (2005) Post-embryonic expression of *C. elegans* microRNAs belonging to the *lin-4* and *let-7* families in the hypodermis and the reproductive system. Developmental Dynamics 234: 868-877.

3. Abbott AL, Alvarez-Saavedra E, Miska EA, Lau NC, Bartel DP, et al. (2005) The *let-7* microRNA family members *mir-48*, *mir-84*, and *mir-241* function together to regulate developmental timing in *Caenorhabditis elegans*. Develop Cell 9: 403-414.

4. Ibanez-Ventoso C, Yang M, Guo S, Robins H, Padgett RW, et al. (2006) Modulated microRNA expression during adult lifespan in *C. elegans*. Aging Cell 5: 235-246.

5. Johnson SM, Grosshans H, Shingara J, Byrom M, Jarvis R, et al. (2005) RAS is regulated by the *let-7* microRNA family. Cell 120: 635-647.

6. Lee RC, Feinbaum RL, Ambros V (1993) The *C. elegans* heterochronic gene *lin-4* encodes small RNAs with antisense complementarity to *lin-14*. Cell 75: 843-854.

7. Boehm M, Slack FJ (2005) A developmental timing microRNA and its target regulate life span in *C. elegans*. Science 310: 1954-1957.

8. Lau NC, Lim LP, Weinstein EG, Bartel DP (2001) An abundant class of tiny RNAs with probable regulatory roles in *Caenorhabditis elegans*. Science 294: 858-862.

9. Lim LP, Lau NC, Weinstein EG, Abdelhakim A, Yekta S, et al. (2003) The microRNAs of *Caenorhabditis elegans*. Genes & Development 17: 991-1008.

10. Yoo AS, Greenwald I (2005) LIN-12/Notch activation leads to microRNA-mediated down-regulation of vav in *C. elegans*. Science 310: 1330-1333.

11. Chang S, Johnston RJ, Frokjaer-Jensen C, Lockery S, Hobert O (2004) MicroRNAs act sequentially and asymmetrically to control chemosensory laterality in the nematode. Nature 430: 785-789.
