# Supplementary material for: Sequence Relationships among C. elegans, D. melanogaster and Human microRNAs Highlight the Extensive Conservation of microRNAs in Biology
Source: PLoS One. 2008 Jul 30;3(7):e2818. doi: 10.1371/journal.pone.0002818 (PMC2486268; doi:10.1371/journal.pone.0002818)
Supplement: Dataset S2 — Homology table and sequence alignments of C. elegans miRNAs with ≥70% overall sequence identity. (0.25 MB DOC) [file pone.0002818.s006.doc]

**Supplementary Table S2: Search criteria of ≥70% similarity over the length of *C. elegans* miRNA mature sequences identifies 15 families with 45 members*.***

**?** = Unknown function.

| **miRNA Group ID** | **Family Members** | **Sub-groups** | **Functional Information** |
| --- | --- | --- | --- |
| **let-7** | cel-let-7 |  | Cell fate in L4-Adult transition [1]; L2-Adult expression [2,3]; Regulated during adult aging [4] |
|  | cel-miR-84 |  | Cell fate in larval transitions and vulva morphogenesis [3,5]; Post-embryonic expression enhanced at L3 stage [2,3] |
| **miR-1** | cel-miR-1 |  | Expressed throughout development [6,7]; Regulated during adult aging [4] |
|  | cel-miR-256 |  | **?** |
| **miR-2** | cel-miR-2 |  | Expressed throughout development [6]; Regulated during adult aging [4] |
|  | cel-miR-43 |  | Larval and predominantly embryonic expression [6,7]; Regulated during adult aging [4] |
| **miR-39** | cel-miR-35 | cel-miR-35 cel-miR-37 | Embryonic and adult expression [6,7]; Regulated during adult aging [4] |
|  | cel-miR-36 | cel-miR-36 cel-miR-39 cel-miR-41 | Embryonic and adult expression [6,7]; Regulated during adult aging [4] |
|  | cel-miR-37 |  | Embryonic and adult expression [6,7]; Regulated during adult aging [4] |
|  | cel-miR-38 |  | Embryonic and adult expression [6,7]; Regulated during adult aging [4] |
|  | cel-miR-39 | cel-miR-39 cel-miR-40 | Embryonic and adult expression [6,7]; Regulated during adult aging [4] |
|  | cel-miR-40 |  | Embryonic and adult expression [6,7] |
|  | cel-miR-41 |  | Regulated during adult aging [4] |
|  | cel-miR-42 |  | Larval and predominantly embryonic expression [6,7]; Regulated during adult aging [4] |
|  | cel-miR-271 |  | **?** |
| **miR-44** | cel-miR-44 |  | Expressed throughout development [6,7] |
|  | cel-miR-45 |  | Expressed throughout development [6,7]; Regulated during adult aging [4] |
| **miR-46** | cel-miR-46 |  | Expressed throughout development [6,7]; Regulated during adult aging [4] |
|  | cel-miR-47 |  | Expressed throughout development [6,7]; Regulated during adult aging [4] |
| **miR-55** | cel-miR-52 | cel-miR-52 cel-miR-53 | Expressed throughout development [6,7] |
|  | cel-miR-53 |  | Expressed throughout development [6] |
|  | cel-miR-54 |  | Expressed throughout development [6]; Regulated during adult aging [4] |
|  | cel-miR-55 | cel-miR-55 cel-miR-56 | Expressed throughout development [6] |
|  | cel-miR-56 |  | Expressed throughout development [6]; Regulated during adult aging [4] |
|  | cel-miR-273 |  | Left/right neuronal asymmetry [8]; Regulated during adult aging [4] |
| **miR-61** | cel-miR-61 |  | Vulval morphogenesis [9]; Expressed throughout development [6] |
|  | cel-miR-247 |  | Expression at L3 stage and Enhanced at dauer stage [6] |
| **miR-64** | cel-miR-63 |  | Expressed throughout development [6]; Regulated during adult aging [4] |
|  | cel-miR-64 | cel-miR-64 cel-miR-65 | Expressed throughout development [6,7]; Regulated during adult aging [4] |
|  | cel-miR-65 |  | Expressed throughout development [6,7]; Regulated during adult aging [4] |
| **miR-73** | cel-miR-73 |  | Expressed throughout development [6]; Regulated during adult aging [4] |
|  | cel-miR-268 |  | Regulated during adult aging [4] |
|  | cel-miR-270 |  | **?** |
| **miR-78** | cel-miR-78 |  | **?** |
|  | cel-miR-272 |  | **?** |
| **miR-82** | cel-miR-80 |  | Expressed throughout development [6,7] |
|  | cel-miR-81 | cel-miR-81 cel-miR-82 | Expressed throughout development [6]; Regulated during adult aging [4] |
|  | cel-miR-82 |  | Expressed throughout development [6]; Regulated during adult aging [4] |
| **miR-239a** | cel-miR-239a |  | Predominantly post-embryonic expression [6]; Regulated during adult aging [4] |
|  | cel-miR-239b |  | Predominantly post-embryonic expression [6] |
| **miR-251** | cel-miR-251 |  | Expressed throughout development [6]; Regulated during adult aging [4] |
|  | cel-miR-252 |  | Expressed throughout development [6] |
| **miR-266** | cel-miR-72 |  | Expressed throughout development [6] |
|  | cel-miR-266 |  | **?** |
|  | cel-miR-269 |  | **?** |

**Supplementary Alignments S2:**

**Sequence alignments of *C. elegans* miRNAs grouped based on ≥70% overall sequence identity.** Members of families are ≥70% identical to at least one other miRNA member. Percentages at the end of miRNA sequences indicate identity with the reference miRNA of their corresponding family (top of group alignment)—the reference miRNA has the closest sequence to the consensus sequence of a miRNA family. Sub-groups contain miRNA family members with ≥80% sequence identity. Grey shading indicates potential G..U pairing.

**let-7: cel-let-7, cel-miR-84**

1 22

cel-let-7 UGAGGUAGUAGGUUGUAUAGUU

cel-miR-84 UGAGGUAGUAUGUAAUAUUGUA 77.3%

**miR-1: cel-miR-1, cel-miR-256**

1 21

cel-miR-1 UGGAAUGUAAAGAAGUAUGUA

cel-miR-256 UGGAAUGCAUAGAAGACUGUA 81.0%

**miR-2: cel-miR-2, cel-miR-43**

1 24

cel-miR-2 UAUCACAGCCAGCUUUGAUGU-GC

cel-miR-43 UAUCACAGUUUACUU-GCUGUCGC 70.8%

**miR-39: cel-miR-35, cel-miR-36, cel-miR-37, cel-miR-38,**

**cel-miR-39, cel-miR-40, cel-miR-41, cel-miR-42,**

**cel-miR-271**

1 23

cel-miR-39 UCACCGGGUGU-AAAUCAGCUUG

cel-miR-40 UCACCGGGUGU-ACAUCAGCUAA 86.4%

cel-miR-41 UCACCGGGUGAAAAAUCACCUA- 78.3%

cel-miR-36 UCACCGGGUGAAAAUUCGCAUG- 81.8%

cel-miR-42 UCACCGGGUUA-ACAUCUACAGA 63.6%

cel-miR-38 UCACCGGGAGA-AAAACUGGAGU 63.6%

cel-miR-37 UCACCGGGUGA-ACACUUGCAGU 63.6%

cel-miR-35 UCACCGGGUGG-AAACUAGCAGU 72.7%

cel-miR-271 UCGCCGGGUGGGAAAGCAUU--- 68.2%

**Sub-groups**

**i. cel-miR-35, cel-miR-37:**

1 22

cel-miR-35 UCACCGGGUGGAAACUAGCAGU

cel-miR-37 UCACCGGGUGAACACUUGCAGU 86.4%

**ii. cel-miR-36, cel-miR-39, cel-miR-41:**

1 22

cel-miR-36 UCACCGGGUGAAAAUUCGCAUG

cel-miR-39 UCACCGGGUGUAAAUCAGCUUG 81.8%

cel-miR-41 UCACCGGGUGAAAAAUCACCUA 81.8%

**iii. celmiR-39, cel-miR-40:**

1 22

cel-miR-39 UCACCGGGUGUAAAUCAGCUUG

cel-miR-40 UCACCGGGUGUACAUCAGCUAA 86.4%

**miR-44: cel-miR-44, cel-miR-45**

1 21

cel-miR-44 UGACUAGAGACACAUUCAGCU

cel-miR-45 UGACUAGAGACACAUUCAGCU 100%

**miR-46: cel-miR-46, cel-miR-47**

1 22

cel-miR-46 UGUCAUGGAGUCGCUCUCUUCA

cel-miR-47 UGUCAUGGAGGCGCUCUCUUCA 95.5%

**miR-55: cel-miR-52, cel-miR-53, cel-miR-54, cel-miR-55,**

**cel miR-56, cel-miR-273**

1 27

cel-miR-55 UACCCGUAUAA--GUUUC--UGCUGAG

cel-miR-53 CACCCGUACAUUUGUUUCCGUGCU--- 63.0%

cel-miR-52 CACCCGUACAUAUGUUUCCGUGCU--- 66.7%

cel-miR-56 UACCCGUAAU---GUUUC--CGCUGAG 87.0%

cel-miR-54 UACCCGUAAU---CUUCAUAAUCCGAG 56.0%

cel-miR-273 UGCCCGUACU---GUGUC--GGCUG-- 65.2%

**Sub-groups**

**i. cel-miR-52, cel-miR-53:**

1 24

cel-miR-52 CACCCGUACAUAUGUUUCCGUGCU

cel-miR-53 CACCCGUACAUUUGUUUCCGUGCU 95.8%

**ii. cel-miR-55, cel-miR-56:**

1 23

cel-miR-55 UACCCGUAUAAGUUUCUGCUGAG

cel-miR-56 UACCCGUA-AUGUUUCCGCUGAG 87.0%

**miR-61: cel-miR-61, cel-miR-247**

1 23

cel-miR-61 UGACUAGAACCGUUACUCAUC

cel-miR-247 UGACUAGAGCCUAUUCUCUUCU 72.7%

**miR-64: cel-miR-63, cel-miR-64, cel-miR-65**

1 24

cel-miR-64 UAUGACACUGAAGCGUUACCGAA-

cel-miR-65 UAUGACACUGAAGCGUAACCGAA- 95.7%

cel-miR-63 UAUGACACUGAAGCGAGUUGGAAA 75.0%

**Sub-group cel-miR-64, cel-miR-65:**

1 23

cel-miR-64 UAUGACACUGAAGCGUUACCGAA

cel-miR-65 UAUGACACUGAAGCGUAACCGAA 95.7%

**miR-73: cel-miR-73, cel-miR-268, cel-miR-270**

1 25

cel-miR-73 UGGCAAGAUGUAGG--CAGUUCAGU

cel-miR-270 -GGCAUGAUGUAG---CAGUGGAG- 73.9%

cel-miR-268 -GGCAAGAAUUAGAAGCAGUUUGGU 72.0%

**miR-78: cel-miR-78, cel-miR-272**

1 21

cel-miR-78 UGGAGGCCUGGUUGUUUGUGC

cel-miR-272 UGUAGGCAUGGGUGUUUG--- 71.4%

**miR-82: cel-miR-80, cel-miR-81, cel-miR-82**

1 24

cel-miR-82 UGAGAUCAUCG—-UGAAAGCCAGU

cel-miR-81 UGAGAUCAUCG--UGAAAGCUAGU 95.5%

cel-miR-80 UGAGAUCAUUAGUUGAAAGCCGA- 70.8%

**Sub-group cel-miR-81, cel-miR-82:**

1 22

cel-miR-81 UGAGAUCAUCGUGAAAGCUAGU

cel-miR-82 UGAGAUCAUCGUGAAAGCCAGU 95.5%

**miR-239a: cel-miR-239a, cel-miR-239b**

1 23

cel-miR-239a UUUGUACUACACAUAGGUACUGG

cel-miR-239b UUUGUACUACACAAAAGUACUG- 87.0%

**miR-251: cel-miR-251, cel-miR-252**

1 24

cel-miR-251 UUAAGUAGUGGUGCCGCUCUUAUU

cel-miR-252 AUAAGUAGUAGUGCCGCAGGUAA- 70.8%

**miR-266: cel-miR-72, cel-miR-266, cel-miR-269**

1 21

cel-miR-266 AGGCAAGACUUUGGCAAAGC-

cel-miR-269 -GGCAAGACUCUGGCAAAACU 81.0%

cel-miR-72 AGGCAAGAUGUUGGCAUAGCUGA 73.9%

REFERENCES

1. Reinhart BJ, Slack FJ, Basson M, Pasquinelli AE, Bettinger JC, et al. (2000) The 21-nucleotide *let-7* RNA regulates developmental timing in *Caenorhabditis elegans*. Nature 403: 901-906.

2. Esquela-Kerscher A, Johnson SM, Bai L, Saito K, Partridge J, et al. (2005) Post-embryonic expression of *C. elegans* microRNAs belonging to the *lin-4* and *let-7* families in the hypodermis and the reproductive system. Developmental Dynamics 234: 868-877.

3. Abbott AL, Alvarez-Saavedra E, Miska EA, Lau NC, Bartel DP, et al. (2005) The *let-7* microRNA family members *mir-48*, *mir-84*, and *mir-241* function together to regulate developmental timing in *Caenorhabditis elegans*. Develop Cell 9: 403-414.

4. Ibanez-Ventoso C, Yang M, Guo S, Robins H, Padgett RW, et al. (2006) Modulated microRNA expression during adult lifespan in *C. elegans*. Aging Cell 5: 235-246.

5. Johnson SM, Grosshans H, Shingara J, Byrom M, Jarvis R, et al. (2005) RAS is regulated by the *let-7* microRNA family. Cell 120: 635-647.

6. Lim LP, Lau NC, Weinstein EG, Abdelhakim A, Yekta S, et al. (2003) The microRNAs of *Caenorhabditis elegans*. Genes & Development 17: 991-1008.

7. Lau NC, Lim LP, Weinstein EG, Bartel DP (2001) An abundant class of tiny RNAs with probable regulatory roles in *Caenorhabditis elegans*. Science 294: 858-862.

8. Chang S, Johnston RJ, Frokjaer-Jensen C, Lockery S, Hobert O (2004) MicroRNAs act sequentially and asymmetrically to control chemosensory laterality in the nematode. Nature 430: 785-789.

9. Yoo AS, Greenwald I (2005) LIN-12/Notch activation leads to microRNA-mediated down-regulation of vav in *C. elegans*. Science 310: 1330-1333.
