# Supplementary material for: Sequence Relationships among C. elegans, D. melanogaster and Human microRNAs Highlight the Extensive Conservation of microRNAs in Biology
Source: PLoS One. 2008 Jul 30;3(7):e2818. doi: 10.1371/journal.pone.0002818 (PMC2486268; doi:10.1371/journal.pone.0002818)
Supplement: Dataset S3 — Table and alignments of related C. elegans miRNAs with 60–69.9% overall sequence similarity. (0.15 MB DOC) [file pone.0002818.s007.doc]

**Supplementary Table S3: 70 sequence relationships between 68 *C. elegans* miRNAs show 60-69.9% overall similarity.** 31 of these miRNAs are not related in sequence to other miRNAs above the ≥70% threshold (Dataset S2). As the functional significance of the 60-69.9% related sequences might be less certain, it is worthy of note that 53 of the 70 related sequences have ≥7nt-homology block at the 5’ end (Dataset S1, sequence alignments below).

| **miRNA**  **Group ID** | ***D*istantly Related *C. elegans* miRNAs** | | Overall Identity **(60-69.9%)** |
| --- | --- | --- | --- |
| let-7 | cel-let-7 | cel-miR-265 | 62.5 |
|  |  | cel-miR-793 | 60.9 |
| miR-1 | cel-miR-1 | cel-miR-796 | 69.6 |
| miR-34 | cel-miR-34 | cel-miR-264 | 69.6 |
| miR-35 | cel-miR-35 | cel-miR-40 | 68.2 |
|  |  | cel-miR-41 | 68.2 |
|  |  | cel-miR-42 | 63.6 |
| miR-36 | cel-miR-36 | cel-miR-38 | 68.2 |
|  |  | cel-miR-40 | 68.2 |
|  |  | cel-miR-42 | 68.2 |
|  |  | cel-miR-271 | 68.2 |
| miR-37 | cel-miR-37 | cel-miR-40 | 68.2 |
|  |  | cel-miR-41 | 68.2 |
|  |  | cel-miR-39 | 63.6 |
|  |  | cel-miR-271 | 63.6 |
| miR-38 | cel-miR-38 | cel-miR-41 | 69.6 |
|  |  | cel-miR-42 | 68.2 |
|  |  | cel-miR-39 | 63.6 |
|  |  | cel-miR-271 | 63.6 |
| miR-39 | cel-miR-39 | cel-miR-271 | 68.2 |
|  |  | cel-miR-42 | 63.6 |
| miR-41 | cel-miR-41 | cel-miR-42 | 68.2 |
|  |  | cel-miR-271 | 63.6 |
| miR-43 | cel-miR-43 | cel-miR-250 | 65.2 |
|  |  | cel-miR-392 | 60.9 |
| miR-44 | cel-miR-44 | cel-miR-247 | 68.2 |
|  |  | cel-miR-61 | 63.6 |
| miR-45 | cel-miR-45 | cel-miR-247 | 68.2 |
|  |  | cel-miR-61 | 63.6 |
| miR-46 | cel-miR-46 | cel-miR-74 | 63.6 |
| miR-48 | cel-miR-48 | cel-miR-241 | 60.9 |
|  |  | cel-miR-793 | 60.9 |
| miR-51 | cel-miR-51 | cel-miR-57 | 64.0 |
|  |  | cel-miR-52 | 62.5 |
|  |  | cel-miR-53 | 62.5 |
|  |  | cel-miR-54 | 61.5 |
| miR-52 | cel-miR-52 | cel-miR-55 | 66.7 |
|  |  | cel-miR-273 | 60.0 |
| miR-53 | cel-miR-53 | cel-miR-56 | 68.0 |
|  |  | cel-miR-55 | 63.0 |
|  |  | cel-miR-273 | 60.0 |
| miR-55 | cel-miR-55 | cel-miR-267 | 69.6 |
|  |  | cel-miR-273 | 65.2 |
|  |  | cel-miR-57 | 60.0 |
| miR-56 | cel-miR-56 | cel-miR-267 | 63.6 |
| miR-58 | cel-miR-58 | cel-miR-82 | 63.6 |
| miR-62 | cel-miR-62 | cel-miR-792 | 62.5 |
| miR-63 | cel-miR-63 | cel-miR-66 | 62.5 |
| miR-67 | cel-miR-67 | cel-miR-797 | 61.5 |
| miR-72 | cel-miR-72 | cel-miR-73 | 69.6 |
|  |  | cel-miR-269 | 65.2 |
|  |  | cel-miR-270 | 60.9 |
| miR-73 | cel-miR-73 | cel-miR-74 | 69.6 |
| miR-74 | cel-miR-74 | cel-miR-268 | 60.0 |
| miR-80 | cel-miR-80 | cel-miR-81 | 66.7 |
|  |  | cel-miR-793 | 60.0 |
| miR-82 | cel-miR-82 | cel-miR-793 | 65.2 |
| miR-86 | cel-miR-86 | cel-miR-785 | 60.9 |
| miR-87 | cel-miR-87 | cel-miR-233 | 63.6 |
| miR-239a | cel-miR-239a | cel-miR-355 | 60.9 |
| miR-250 | cel-miR-250 | cel-miR-259 | 62.5 |
| miR-256 | cel-miR-256 | cel-miR-796 | 65.2 |
| miR-261 | cel-miR-261 | cel-miR-787 | 65.2 |
| miR-267 | cel-miR-267 | cel-miR-273 | 63.6 |
| miR-353 | cel-miR-353 | cel-miR-357 | 60.9 |
| miR-790 | cel-miR-790 | cel-miR-791 | 65.2 |
| miR-793 | cel-miR-793 | cel-miR-794 | 69.6 |
| miR-1021 | cel-miR-1021 | cel-miR-81 | 69.2 |
|  |  | cel-miR-82 | 65.4 |
| miR-1022 | cel-miR-1022 | cel-miR-82 | 62.5 |

**Supplementary Alignments S3:**

**Aligned sequences of *C. elegans* miRNAs with 60-69.9% overall sequence similarity.** Identity between *C. elegans* miRNAs is given in percentage at the end of each alignment. Grey shading indicates potential G..U pairing.

**let-7: cel-let-7, cel-miR-265 and cel-miR-793**

1 24

cel-let-7 UGAGGUAGUA--GGUUGUAUAGUU

cel-miR-265 UGAGGGAGGAAGGGUGGUAU---- 62.5%

1 23

cel-let-7 UGAGGUAG-UAGGUUGUAUAGUU

cel-miR-793 UGAGGUAUCUUAGUUAGACAGA- 60.9%

**miR-1: cel-miR-1 and cel-miR-796**

1 23

cel-miR-1 UGGAAUGUAAA-GAAGUAUGUA-

cel-miR-796 UGGAAUGUAGUUGAGGUUAGUAA 69.6%

**miR-34: cel-miR-34 and cel-miR-264**

1 23

cel-miR-34 AGGCAGUGUGGUUAGCUGGU-UG

cel-miR-264 -GGCGG-GUGGUU-GUUGUUAUG 69.6%

**miR-35: cel-miR-35, cel-miR-40, cel-miR-41 and cel-miR-42**

1 22

cel-miR-35 UCACCGGGUGGAAACUAGCAGU

cel-miR-40 UCACCGGGUGUACAUCAGCUAA 68.2%

1 22

cel-miR-35 UCACCGGGUGGAAACUAGCAGU

cel-miR-41 UCACCGGGUGAAAAAUCACCUA 68.2%

1 22

cel-miR-35 UCACCGGGUGGAAACUAGCAGU

cel-miR-42 UCACCGGGUUAACAUCUACAGA 63.6%

**miR-36: cel-miR-36, cel-miR-38, cel-miR-40, cel-miR-42 and**

**cel-miR-271**

1 22

cel-miR-36 UCACCGGGUGAAAAUUCGCAUG

cel-miR-38 UCACCGGGAGAAAAACUGGAGU 68.2%

1 22

cel-miR-36 UCACCGGGUGAAAAUUCGCAUG

cel-miR-40 UCACCGGGUGUACAUCAGCUAA 68.2%

1 22

cel-miR-36 UCACCGGGUGAAAAUUCGCAUG

cel-miR-42 UCACCGGGUUAACAUCUACAGA 68.2%

1 22

cel-miR-36 UCACCGGGUGAAAAUUCGCAUG

cel-miR-271 UCGCCGGGUGGGAAA--GCAUU 68.2%

**miR-37: cel-miR-37, cel-miR-39, cel-miR-40, cel-miR-41 and**

**cel-miR-271**

1 22

cel-miR-37 UCACCGGGUGAACACUUGCAGU

cel-miR-40 UCACCGGGUGUACAUCAGCUAA 68.2%

1 22

cel-miR-37 UCACCGGGUGAACACUUGCAGU

cel-miR-41 UCACCGGGUGAAAAAUCACCUA 68.2%

1 22

cel-miR-37 UCACCGGGUGAACACUUGCAGU

cel-miR-39 UCACCGGGUGUAAAUCAGCUUG 63.6%

1 22

cel-miR-37 UCACCGGGUGAACACUUGCAGU

cel-miR-271 UCGCCGGGUGGGAAA--GCAUU 63.6%

**miR-38: cel-miR-38, cel-miR-39, cel-miR-41, cel-miR-42 and**

**cel-miR-271**

1 23

cel-miR-38 UCACCGGGAGAAAAACUGGAGU-

cel-miR-41 UCACCGGGUGAAAAA-UCACCUA 69.6%

1 22

cel-miR-38 UCACCGGGAGAAAAACUGGAGU

cel-miR-42 UCACCGGGUUAACAUCUACAGA 68.2%

1 22

cel-miR-38 UCACCGGGAGAAAAACUGGAGU

cel-miR-39 UCACCGGGUGUAAAUCAGCUUG 63.6%

1 22

cel-miR-38 UCACCGGGAGAAAAACUGGAGU

cel-miR-271 UCGCCGGGUGGGAAA--GCAUU 63.6%

**miR-39: cel-miR-39, cel-miR-42 and cel-miR-271**

1 22

cel-miR-39 UCACCGGGUGUAAAUCAGCUUG

cel-miR-271 UCGCCGGGUGGGAA--AGCAUU 68.2%

1 22

cel-miR-39 UCACCGGGUGUAAAUCAGCUUG

cel-miR-42 UCACCGGGUUAACAUCUACAGA 63.6%

**miR-41: cel-miR-41, cel-miR-42 and cel-miR-271**

1 22

cel-miR-41 UCACCGGGUGAAAAAUCACCUA

cel-miR-42 UCACCGGGUUAACAUCUACAGA 68.2%

1 22

cel-miR-41 UCACCGGGUGAAAAAUCACCUA

cel-miR-271 UCGCCGGGUGGGAAAGCAUU-- 63.6%

**miR-43: cel-miR-43, cel-miR-250 and cel-miR-392**

1 23

cel-miR-43 UAUCACAGUUUACUUGCUGUCGC

cel-miR-250 AAUCACAGUCAACU-GUUGGCA- 65.2%

1 23

cel-miR-43 UAUCACAGUUUACUUGCUGUCGC

cel-miR-392 UAUCAUCGAUCACGUG-UGAUGA 60.9%

**miR-44: cel-miR-44, cel-miR-61 and cel-miR-247**

1 23

cel-miR-44 UGACUAGAGAC-ACAUUCAGCU-

cel-miR-247 UGACUAGAGCCUAUUCUCUUCUU 68.2%

1 23

cel-miR-44 UGACUAGAGACAC-AUUCAGCU-

cel-miR-61 UGACUAGAACCGUUACUCAUCUC 63.6%

**miR-45: cel-miR-45, cel-miR-61 and cel-miR-247**

1 24

cel-miR-45 UGACUAGAGACACAUUCAGCU---

cel-miR-247 UGACUAGAG-CCUAUUCUCUUCUU 68.2%

1 23

cel-miR-45 UGACUAGAGACAC-AUUCAGCU-

cel-miR-61 UGACUAGAACCGUUACUCAUCUC 63.6%

**miR-46: cel-miR-46 and cel-miR-74**

1 22

cel-miR-46 UGUCAUGGAGUCGCUCUCUUCA

cel-miR-74 UGGCAAGAAAUGGCAGUCUACA 63.6%

**miR-48: cel-miR-48, cel-miR-241 and cel-miR-793**

1 23

cel-miR-48 UGAGGUAGGCUCAGUAGAUGCGA

cel-miR-241 UGAGGUAGGUGCGAGAAAUGA-- 60.9%

1 23

cel-miR-48 UGAGGUAGGCUCAGUAGAUGCGA

cel-miR-793 UGAGGUAUCUUAGUUAGACA-GA 60.9%

**miR-51: cel-miR-51, cel-miR-52, cel-miR-53, cel-miR-54 and**

**cel-miR-57**

1 25

cel-miR-51 UACCC-GUAGCUCCUAUCCAUGUU-

cel-miR-57 UACCCUGUAGAUCG-AGCUGUGUGU 64.0%

1 24

cel-miR-51 UACCCGUAGCUCC-UAUCCAUGUU

cel-miR-52 CACCCGUACAUAUGUUUCCGUGCU 62.5%

1 24

cel-miR-51 UACCCGUAGCUCC-UAUCCAUGUU

cel-miR-53 CACCCGUACAUUUGUUUCCGUGCU 62.5%

1 26

cel-miR-51 UACCCGUAGC--UCCUA-UCCAUGUU

cel-miR-54 UACCCGUAAUCUUCAUAAUCCGAG-- 61.5%

**miR-52: cel-miR-52, cel-miR-55 and cel-miR-273**

1 27

cel-miR-52 CACCCGUACAUAUGUUUCCGUGCU---

cel-miR-55 UACCCGUA--UAAGUUUC--UGCUGAG 66.7%

1 25

cel-miR-52 CACCCGUACAUAUGUUUCCG-UGCU

cel-miR-273 UGCCCGUAC---UGUGUCGGCUG-- 60.0%

**miR-53: cel-miR-53, cel-miR-55, cel-miR-56 and cel-miR-273**

1 25

cel-miR-53 CACCCGUACAUUUGUUUCCG-UGCU

cel-miR-56 UACCCGUAA---UGUUUCCGCUGAG 68.0%

1 27

cel-miR-53 CACCCGUACAUUUGUUUCCGUGCU---

cel-miR-55 UACCCGUAUAA--GUUUC--UGCUGAG 63.0%

1 25

cel-miR-53 CACCCGUACAUUUGUUUCCG-UGCU

cel-miR-273 UGCCCGUAC---UGUGUCGGCUG-- 60.0%

**miR-55: cel-miR-55, cel-miR-57, cel-miR-267 and cel-miR-273**

1 23

cel-miR-55 UACCCGUAUAAGUUUCUGCUGAG

cel-miR-267 --CCCGUG-AAGUGUCUGCUGCA 69.6%

1 23

cel-miR-55 UACCCGUAUAAGUUUCUGCUGAG

cel-miR-273 UGCCCGUACU-GUGUCGGCUG-- 65.2%

1 25

cel-miR-55 UACCC-GUAUAAGUUUCUGCUGAG-

cel-miR-57 UACCCUGUAGAUCGAGCUG-UGUGU 60.0%

**miR-56: cel-miR-56 and cel-miR-267**

1 22

cel-miR-56 UACCCGUAAUGUUUCCGCUGAG

cel-miR-267 --CCCGUGAAGUGUCUGCUGCA 63.6%

**miR-58: cel-miR-58 and cel-miR-82**

1 22

cel-miR-58 UGAGAUCGUUCAGUACGGCAAU

cel-miR-82 UGAGAUCAUCGUGAAAGCCAGU 63.6%

**miR-62: cel-miR-62 and cel-miR-792**

1 24

cel-miR-62 -UGAUAUGUAAUCUAGCUUACAG-

cel-miR-792 UUGAAAUCUCUUCAA-CUUUCAGA 62.5%

**miR-63: cel-miR-63 and cel-miR-66**

1 24

cel-miR-63 UAUGACACUGAAGCGAGUUGGAAA

cel-miR-66 CAUGACACUGAUUAGGGAUGUGA- 62.5%

**miR-67: cel-miR-67 and cel-miR-797**

1 26

cel-miR-67 --UCACAACCUCCUAGAAAGAGUAGA

cel-miR-797 UAUCACAGCAAUC-ACAAUGAGAAGA 61.5%

**miR-72: cel-miR-72, cel-miR-73, cel-miR-269 and cel-miR-270**

1 23

cel-miR-72 AGGCAAGAUGUUGGCAUAGC---

cel-miR-73 UGGCAAGAUGUAGGCAGUUCAGU 69.6%

1 23

cel-miR-72 AGGCAAGAUGUUGGCAUAGCUGA

cel-miR-269 -GGCAAGACUCUGGCAAAACU-- 65.2%

1 23

cel-miR-72 AGGCAAGAUGUUGGCAUAGCUGA

cel-miR-270 -GGCAUGAUGUAG-CAGUGGAG- 60.9%

**miR-73: cel-miR-73 and cel-miR-74**

1 23

cel-miR-73 UGGCAAGAUGUAGGCAGUUCAGU

cel-miR-74 UGGCAAGAAAU-GGCAGUCUACA 69.6%

**miR-74: cel-miR-74 and cel-miR-268**

1 25

cel-miR-74 UGGCAAGAAAUGG---CAGUCUACA

cel-miR-268 -GGCAAGAAUUAGAAGCAGUUUGGU 60.0%

**miR-80: cel-miR-80, cel-miR-81 and cel-miR-793**

1 24

cel-miR-80 UGAGAUCAUUAGUUGAAAGCCGA-

cel-miR-81 UGAGAUCAUCG--UGAAAGCUAGU 66.7%

1 25

cel-miR-80 UGAGAUCA-UUAGUU-GAAAGCCGA

cel-miR-793 UGAGGUAUCUUAGUUAGACAGA--- 60.0%

**miR-82: cel-miR-82 and cel-miR-793**

1 23

cel-miR-82 UGAGAUCAUCGU-GAAAGCCAGU

cel-miR-793 UGAGGU-AUCUUAGUUAGACAGA 65.2%

**miR-86: cel-miR-86 and cel-miR-785**

1 23

cel-miR-86 UAAGUGAAUGCUUUGCCACAGUC

cel-miR-785 UAAGUGAAUUGUUUUGUGUAGA- 60.9%

**miR-87: cel-miR-87 and cel-miR-233**

1 22

cel-miR-87 GUGAGCAAAGUUUCAGGUGUGC

cel-miR-233 UUGAGCAAUGCG-CAUGUGCGG 63.6%

**miR-239a: cel-miR-239a and cel-miR-355**

1 23

cel-miR-239a UUUGUACUACACAUAGGUACUGG

cel-miR-355 UUUGUUUUAGCCUGAGCUA-UG- 60.9%

**miR-250: cel-miR-250 and cel-miR-259**

1 24

cel-miR-250 -AAUCACAGUCAA--CUGUUGGCA

cel-miR-259 AAAUCUCAUCCUAAUCUGGUAGCA 62.5%

**miR-256: cel-miR-256 and cel-miR-796**

1 23

cel-miR-256 UGGAAUGCA-UAGAAGACUGUA-

cel-miR-796 UGGAAUGUAGUUGAGGUUAGUAA 65.2%

**miR-261: cel-miR-261 and cel-miR-787**

1 23

cel-miR-261 -UAGCU--UUUUAGUUU-UCACG

cel-miR-787 UAAGCUCGUUUUAGUAUCUUUCG 65.2%

**miR-267: cel-miR-267 and cel-miR-273**

1 22

cel-miR-267 --CCCGUGAAGUGUCUGCUGCA

cel-miR-273 UGCCCGUACUGUGUCGGCUG-- 63.6%

**miR-353: cel-miR-353 and cel-miR-357**

1 23

cel-miR-353 CAAUUGCCAUGUGUUG--GUAUU

cel-miR-357 UAAAUGCCAGUCGUUGCAGGAGU 60.9%

**miR-790: cel-miR-790 and cel-miR-791**

1 23

cel-miR-790 CUUGGCACUC-GCGAACACCGCG

cel-miR-791 UUUGGCACUCCGCAGAUAAGGCA 65.2%

**miR-793: cel-miR-793 and cel-miR-794**

1 23

cel-miR-793 UGAGGUA-UCUUAGUUAGACAGA

cel-miR-794 UGAGGUAAUCAUCGUU-GUCACU 69.6%

**miR-1021: cel-miR-81, cel-miR-82 and cel-miR-1021**

1 26

cel-miR-1021 AAGUGAGAUCAU-GUGAAAUCCUCGG

cel-miR-81 ---UGAGAUCAUCGUGAAAGC-UAGU 69.2%

1 26

cel-miR-1021 AAGUGAGAUCAU-GUGAAAUCCUCGG

cel-miR-82 ---UGAGAUCAUCGUGAAAGCCAGU- 65.4%

**miR-1022: cel-miR-82 and cel-miR-1022**

1 24

cel-miR-1022 -AAGAUCAUUGUUAGGACGCCAUC

cel-miR-82 UGAGAUCAUCGU--GAAAGCCAGU 62.5%
