# Supplementary material for: Sequence Relationships among C. elegans, D. melanogaster and Human microRNAs Highlight the Extensive Conservation of microRNAs in Biology
Source: PLoS One. 2008 Jul 30;3(7):e2818. doi: 10.1371/journal.pone.0002818 (PMC2486268; doi:10.1371/journal.pone.0002818)
Supplement: Dataset S4 — Homology table and sequence alignments of D. melanogaster miRNAs with similar 5′ ends. (0.12 MB DOC) [file pone.0002818.s008.doc]

**Supplementary Table S4: Searches for 7nt-long identities at the 5’ ends of *D. melanogaster* miRNAs detects 61 5’ homologs that can be grouped into 19 families.** The “less than” symbol (**<**) before miRNA names indicates that A-G base changes (G..U pairing) interrupt the 5’ 7nt homology block grouping specific miRNAs into families (see alignments below). Sequence similarities grouping miRNAs into families are summarized in the table and detailed in alignments. **(5’)** indicates 48 miRNAs that have high identity at the 5’ end but <70% overall similarity to some of their 5’ sequence-related miRNAs. Of these, 11 miRNAs (dme-bantam, dme-miR-7, dme-miR-14, dme-miR-219, dme-miR-280, dme-miR-289, dme-miR-306*, dme-miR-315, dme-miR-316, dme-miR-960 and dme-miR-967) are significantly related in sequence only at the 5’ end—they do not have ≥60% similarity over their full sequence with their 5’ sequence related miRNAs and thus are not included in Datasets S5 and S6.

| **miRNA**  **Group ID** | **Family Members** | **Sub-groups** |
| --- | --- | --- |
| **bantam**  *GA/GGA/GUCA* | **<**dme-bantam **(5’)** |  |
|  | **<**dme-miR-306* **(5’)** |  |
| **let-7**  *(U)G/AAGG/AUA(G/A)* | dme-let-7 **(5’)** | dme-let-7  dme-miR-984 |
|  | **<**dme-miR-963 **(5’)** |  |
|  | **<**dme-miR-977 **(5’)** |  |
|  | dme-miR-984 **(5’)** |  |
| **miR-2a**  *AUCACAG* | dme-miR-2a **(5’)** | dme-miR-2a  dme-miR-2b dme-miR-2c  dme-miR-13a  dme-miR-13b |
|  | dme-miR-2b **(5’)** |  |
|  | dme-miR-2c **(5’)** |  |
|  | dme-miR-6 **(5’)** |  |
|  | dme-miR-11 **(5’)** |  |
|  | dme-miR-13a **(5’)** |  |
|  | dme-miR-13b **(5’)** |  |
|  | dme-miR-308 **(5’)** |  |
| **miR-3**  *CACUGGG* | dme-miR-3 |  |
|  | dme-miR-309 **(5’)** |  |
|  | dme-miR-318 **(5’)** |  |
| **miR-9a**  *UCUUUGG* | dme-miR-9a |  |
|  | dme-miR-9b |  |
|  | dme-miR-9c |  |
| **miR-12**  *(UA/GA)A/GUAU(UUA)* | **<**dme-miR-12 **(5’)** | dme-miR-12  dme-miR-960 |
|  | **<**dme-miR-280 **(5’)** | **<**dme-miR-280  dme-miR-289 |
|  | **<**dme-miR-283 **(5’)** | dme-miR-283  dme-miR-289 |
|  | **<**dme-miR-289 **(5’)** |  |
|  | **<**dme-miR-960 **(5’)** |  |
| **miR-14**  *GUCUUUU* | dme-miR-14 **(5’)** |  |
|  | dme-miR-316 **(5’)** |  |
| **miR-31a**  *UGGCAAG* | dme-miR-31a |  |
|  | dme-miR-31b |  |
| **miR-219**  *UGAUUGU* | dme-miR-219 **(5’)** |  |
|  | dme-miR-315 **(5’)** |  |
| **miR-275**  *(C)AGG/AUAC(C)* | dme-miR-275 **(5’)** | dme-miR-275  dme-miR-306 |
|  | dme-miR-306 **(5’)** |  |
|  | **<**dme-miR-967 **(5’)** |  |
| **miR-276a**  *UAGGAAC* | dme-miR-276a |  |
|  | dme-miR-276b |  |
| **miR-279**  *UGACUAG* | dme-miR-279 **(5’)** |  |
|  | dme-miR-286 **(5’)** |  |
|  | dme-miR-996 **(5’)** |  |
| **miR-281-2***  *AG/AA/GG/ACUA/G* | **<**dme-miR-4 **(5’)** | dme-miR-4  dme-miR-79 |
|  | **<**dme-miR-7 **(5’)** |  |
|  | **<**dme-miR-79 **(5’)** |  |
|  | **<**dme-miR-281-1* **(5’)** | dme-miR-281-1*  dme-miR-281-2* |
|  | **<**dme-miR-281-2* **(5’)** |  |
| **miR-285**  *UAGCACC* | dme-miR-285 |  |
|  | dme-miR-995 |  |
|  | dme-miR-998 |  |
| **miR-312**  *AUUGCAC* | dme-miR-92a **(5’)** |  |
|  | dme-miR-92b **(5’)** |  |
|  | dme-miR-310 **(5’)** |  |
|  | dme-miR-311 **(5’)** |  |
|  | dme-miR-312 **(5’)** | dme-miR-312  dme-miR-313 |
|  | dme-miR-313 **(5’)** |  |
| **miR-1003**  *UCUCACA* | dme-miR-1003 **(5’)** |  |
|  | dme-miR-1004 **(5’)** |  |
| **miR-1006**  *AAAUUC(G/A)* | **<**dme-miR-1006 **(5’)** |  |
|  | **<**dme-miR-1014 **(5’)** |  |
| **miR-1010**  *UUCACCU* | dme-miR-1010 **(5’)** |  |
|  | dme-miR-1016 **(5’)** |  |
| **miR-iab4as-5p**  *ACGUAUA* | dme-miR-iab4as-5p |  |
|  | dme-miR-iab-4-5p |  |

**Supplementary Alignments S4:**

**5’ end sequence alignments of *D. melanogaster* miRNAs with significant homology at the 5’ 10nt.** Members of a group have ≥7 continuous nt of homology with at least one other group member. Nucleotides at the end of sequences indicate number of residues identical to the reference miRNA (top of group alignment), which has the closest sequence to the group consensus sequence. Sub-groups contain miRNAs with most closely similar 5’ end sequences. Grey shading denotes potential G..U pairing. The “less than” symbol (**<**) indicates allowed A-G base changes (G..U pairing) interrupting the 7nt homology block at the 5' end (10nt) which groups miRNAs into families. Superscript (5’) indicates miRNAs with 5’ end identity but <70% overall similarity to some of their miRNA homologs. Highlighted in blue are 11 miRNAs that show high homology at the 5’ end but low whole sequence similarity (<60%) with all their 5’ sequence-related miRNAs.

**bantam: <dme-bantam^(5’)^, <dme-miR-306*^(5’)^**

1 10

dme-bantam UGAGAUCAUU

dme-miR-306* GGGGGUCACU 6nt

**let-7: dme-let-7^(5’)^, <dme-miR-963^(5’)^ ,<dme-miR-977^(5’)^,**

**dme-miR-984^(5’)^**

1

dme-let-7 -UGAGGUAGUA

dme-miR-984 -UGAGGUAAAU 7nt

dme-miR-963 ACAAGGUAAA- 5nt

dme-miR-977 -UGAGAUAUUC 7nt

**Sub-group dme-let-7, dme-miR-984:**

1 10

dme-let-7 UGAGGUAGUA

dme-miR-984 UGAGGUAAAU 7nt

**miR-2a: dme-miR-2a^(5’)^, dme-miR-2b^(5’)^, dme-miR-2c^(5’)^,**

**dme-miR-6^(5’)^, dme-miR-11^(5’)^, dme-miR-13a^(5’)^,**

**dme-miR-13b^(5’)^, dme-miR-308^(5’)^**

1 10

dme-miR-2a UAUCACAGCC

dme-miR-2b UAUCACAGCC 10nt

dme-miR-2c UAUCACAGCC 10nt

dme-miR-11 CAUCACAGUC 8nt

dme-miR-6 UAUCACAGUG 8nt

dme-miR-13a UAUCACAGCC 10nt

dme-miR-13b UAUCACAGCC 10nt

dme-miR-308 AAUCACAGGA 7nt

**Sub-group dme-miR-2a, dme-miR-2b, dme-miR-2c,**

**dme-miR-13a, dme-miR-13b:**

1 10

dme-miR-2a UAUCACAGCC

dme-miR-2b UAUCACAGCC 10nt

dme-miR-2c UAUCACAGCC 10nt

dme-miR-13a UAUCACAGCC 10nt

dme-miR-13b UAUCACAGCC 10nt

**miR-3: dme-miR-3, dme-miR-309^(5’)^, dme-miR-318^(5’)^**

1 10

dme-miR-3 UCACUGGGCA

dme-miR-309 GCACUGGGUA 8nt

dme-miR-318 UCACUGGGCU 9nt

**miR-9a: dme-miR-9a, dme-miR-9b, dme-miR-9c**

1 10

dme-miR-9a UCUUUGGUUA

dme-miR-9b UCUUUGGUGA 9nt

dme-miR-9c UCUUUGGUAU 8nt

**miR-12: <dme-miR-12^(5’)^, <dme-miR-280^(5’)^, <dme-miR-283^(5’)^,**

**<dme-miR-289^(5’)^, <dme-miR-960^(5’)^**

1

dme-miR-12 UGAGUAUUAC--

dme-miR-960 UGAGUAUUCC-- 9nt

dme-miR-280 --UGUAUUUACG 4nt

dme-miR-283 UAAAUAUCAG-- 6nt

dme-miR-289 UAAAUAUUUA-- 6nt

**Sub-groups**

**i. dme-miR-12, dme-miR-960**

1 10

dme-miR-12 UGAGUAUUAC

dme-miR-960 UGAGUAUUCC 9nt

**i. <dme-miR-280, dme-miR-289**

1

dme-miR-289 UAAAUAUUUA--

dme-miR-280 --UGUAUUUACG 6nt

**ii. dme-miR-283, dme-miR-289**

1 10

dme-miR-289 UAAAUAUUUA

dme-miR-283 UAAAUAUCAG 7nt

**miR-14: dme-miR-14^(5’)^, dme-miR-316^(5’)^**

1

dme-miR-14 UCAGUCUUUU--

dme-miR-316 --UGUCUUUUUC 7nt

**miR-31a: dme-miR-31a, dme-miR-31b**

1 10

dme-miR-31a UGGCAAGAUG

dme-miR-31b UGGCAAGAUG 10nt

**miR-219: dme-miR-219^(5’)^, dme-miR-315^(5’)^**

1

dme-miR-219 ---UGAUUGUCCA

dme-miR-315 UUUUGAUUGU--- 7nt

**miR-275: dme-miR-275^(5’)^, dme-miR-306^(5’)^, <dme-miR-967^(5’)^**

1 10

dme-miR-275 UCAGGUACCU

dme-miR-306 UCAGGUACUU 9nt

dme-miR-967 AGAGAUACCU 7nt

**Sub-group dme-miR-275, dme-miR-306:**

1 10

dme-miR-275 UCAGGUACCU

dme-miR-306 UCAGGUACUU 9nt

**miR-276a: dme-miR-276a, dme-miR-276b**

1 10

dme-miR-276a UAGGAACUUC

dme-miR-276b UAGGAACUUA 9nt

**miR-279: dme-miR-279^(5’)^, dme-miR-286^(5’)^, dme-miR-996^(5’)^**

1 10

dme-miR-279 UGACUAGAUC

dme-miR-286 UGACUAGACC 9nt

dme-miR-996 UGACUAGAUU 9nt

**miR-281-2*: <dme-miR-4^(5’)^, <dme-miR-7^(5’)^, <dme-miR-79^(5’)^,**

**<dme-miR-281-1*^(5’)^, <dme-miR-281-2*^(5’)^**

1

dme-miR-281-2* AAGAGAGCUA--

dme-miR-281-1* AAGAGAGCUG-- 9nt

dme-miR-4 -AUAAAGCUAG- 7nt

dme-miR-7 UGGAAGACUA-- 5nt

dme-miR-79 –-UAAAGCUAGA 6nt

**Sub-groups**

1. **dme-miR-281-1*, dme-miR-281-2*:**

1 10

dme-miR-281-1* AAGAGAGCUG

dme-miR-281-2* AAGAGAGCUA 9nt

1. **dme-miR-4, dme-miR-79**

1

dme-miR-4 AUAAAGCUAG-

dme-miR-79 -UAAAGCUAGA 9nt

**miR-285: dme-miR-285, dme-miR-995, dme-miR-998**

1 10

dme-miR-285 UAGCACCAUU

dme-miR-995 UAGCACCACA 8nt

dme-miR-998 UAGCACCAUG 9nt

**miR-312: dme-miR-92a^(5’)^, dme-miR-92b^(5’)^, dme-miR-310^(5’)^,**

**dme-miR-311^(5’)^, dme-miR-312^(5’)^, dme-miR-313^(5’)^**

1 10

dme-miR-312 UAUUGCACUU

dme-miR-313 UAUUGCACUU 10nt

dme-miR-311 UAUUGCACAU 9nt

dme-miR-310 UAUUGCACAC 8nt

dme-miR-92a CAUUGCACUU 9nt

dme-miR-92b AAUUGCACUA 8nt

**Sub-group dme-miR-312, dme-miR-313:**

1 10

dme-miR-312 UAUUGCACUU

dme-miR-313 UAUUGCACUU 10nt

**miR-1003: dme-miR-1003^(5’)^, dme-miR-1004^(5’)^**

1 10

dme-miR-1003 UCUCACAUUU

dme-miR-1004 UCUCACAUCA 8nt

**miR-1006: <dme-miR-1006^(5’)^, <dme-miR-1014^(5’)^**

1 10

dme-miR-1006 UAAAUUCGAU

dme-miR-1014 AAAAUUCAUU 7nt

**miR-1010: dme-miR-1010^(5’)^, dme-miR-1016^(5’)^**

1

dme-miR-1010 UUUCACCUAU

dme-miR-1016 -UUCACCUCUC 8nt

**miR-iab4as-5p: dme-miR-iab4as-5p, dme-miR-iab-4-5p**

1

dme-miR-iab-4-5p --ACGUAUACUG

dme-miR-iab4as-5p UUACGUAUAC--
