# Supplementary material for: Sequence Relationships among C. elegans, D. melanogaster and Human microRNAs Highlight the Extensive Conservation of microRNAs in Biology
Source: PLoS One. 2008 Jul 30;3(7):e2818. doi: 10.1371/journal.pone.0002818 (PMC2486268; doi:10.1371/journal.pone.0002818)
Supplement: Dataset S5 — Homology table and alignments of D. melanogaster miRNAs showing ≥70% overall sequence identity. (0.08 MB DOC) [file pone.0002818.s009.doc]

**Supplementary Table S5: Searches of ≥70% sequence identity over the lengths of *D. melanogaster* miRNAs identify 14 families with 38 members*.***

| **miRNA Group ID** | **Family Members** | Sub-groups |
| --- | --- | --- |
| **miR-2a** | dme-miR-2a | dme-miR-2a  dme-miR-2b  dme-miR-2c |
|  | dme-miR-2b |  |
|  | dme-miR-2c |  |
|  | dme-miR-13a | dme-miR-13a  dme-miR-13b |
|  | dme-miR-13b |  |
| **miR-3** | dme-miR-3 |  |
|  | dme-miR-309 |  |
|  | dme-miR-318 |  |
| **miR-9a** | dme-miR-9a |  |
|  | dme-miR-9b |  |
|  | dme-miR-9c |  |
| **miR-10** | dme-miR-10 |  |
|  | dme-miR-100 |  |
| **miR-31a** | dme-miR-31a |  |
|  | dme-miR-31b |  |
| **miR-92a** | dme-miR-92a | dme-miR-92a  dme-miR-92b |
|  | dme-miR-92b |  |
|  | dme-miR-310 |  |
|  | dme-miR-311 |  |
|  | dme-miR-312 |  |
|  | dme-miR-313 |  |
| **miR-263a** | dme-miR-263a |  |
|  | dme-miR-263b |  |
| **miR-276a** | dme-miR-276a |  |
|  | dme-miR-276b |  |
| **miR-281-1*** | dme-miR-281-1* |  |
|  | dme-miR-281-2* |  |
| **miR-954** | dme-miR-954 |  |
|  | dme-miR-966 |  |
| **miR-998** | dme-miR-285 |  |
|  | dme-miR-995 |  |
|  | dme-miR-998 |  |
| **miR-1009** | dme-miR-1009 |  |
|  | dme-miR-1010 |  |
| **miR-miR-iab-4-3p** | dme-miR-iab-4-3p |  |
|  | dme-miR-iab4as-3p |  |
| **miR-miR-iab-4-5p** | dme-miR-iab-4-5p |  |
|  | dme-miR-iab4as-5p |  |

**Supplementary Alignments S5:**

**Sequence alignments of *D. melanogaster* miRNAs with ≥70% overall sequence identity.** Members of families are ≥70% identical to at least one other miRNA member. Percentages at the end of miRNA sequences indicate identity with the reference miRNA of their corresponding family (top of group alignment)—the reference miRNA has the closest sequence to the consensus sequence of a miRNA family. Sub-groups contain miRNA family members with ≥80% sequence identity. Grey shading indicates potential G..U pairing

**miR-2a: dme-miR-2a, dme-miR-2b, dme-miR-2c,**

**dme-miR-13a, dme-miR-13b**

1 23

dme-miR-2a UAUCACAGCCAGCUUUGAUGAGC

dme-miR-2b UAUCACAGCCAGCUUUGAGGAGC 95.7%

dme-miR-2c UAUCACAGCCAGCUUUGAUGGGC 95.7%

dme-miR-13a UAUCACAGCCAU-UUUGAUGAGU 87.0%

dme-miR-13b UAUCACAGCCAU-UUUGACGAGU 82.6%

**Sub-groups**

**i. dme-miR-2a, dme-miR-2b, dme-miR-2c:**

1 23

dme-miR-2a UAUCACAGCCAGCUUUGAUGAGC

dme-miR-2b UAUCACAGCCAGCUUUGAGGAGC 95.7%

dme-miR-2c UAUCACAGCCAGCUUUGAUGGGC 95.7%

**ii. dme-miR-13a, dme-miR-13b:**

1 22

dme-miR-13a UAUCACAGCCAUUUUGAUGAGU

dme-miR-13b UAUCACAGCCAUUUUGACGAGU 95.5%

**miR-3: dme-miR-3, dme-miR-309, dme-miR-318**

1 22

dme-miR-3 UCACUGGGCAAAGUGUGUCUCA

dme-miR-309 GCACUGGGUAAAGUUUGUCCUA 77.3%

dme-miR-318 UCACUGGGCUUUGUUUAUCUCA 77.3%

**miR-9a: dme-miR-9a, dme-miR-9b, dme-miR-9c**

1 24

dme-miR-9a UCUUUGGUUAUCU-AGCUGUAUGA

dme-miR-9b UCUUUGGUGAUUUUAGCUGUAUG- 83.3%

dme-miR-9c UCUUUGGUAUUCU-AGCUGUAGA- 87.0%

**miR-10: dme-miR-10 and dme-miR-100**

1 22

dme-miR-10 ACCCUGUAGAUCCGAAUUUGU-

dme-miR-100 AACCCGUAAAUCCGAACUUGUG 77.3%

**miR-31a: dme-miR-31a, dme-miR-31b**

1 23

dme-miR-31a UGGCAAGAUGUCGGCAUAGCUGA

dme-miR-31b UGGCAAGAUGUCGGAAUAGCUG- 91.3%

**miR-92a: dme-miR-92a, dme-miR-92b, dme-miR-310,**

**dme-miR-311, dme-miR-312, dme-miR-313**

1 22

dme-miR-92a CAUUGCACUUGUCCCGGCCUAU

dme-miR-92b AAUUGCACUAGUCCCGGCCUGC 81.8%

dme-miR-310 UAUUGCACACUUCCCGGCCUUU 77.3%

dme-miR-311 UAUUGCACAUUCACCGGCCUGA 68.2%

dme-miR-312 UAUUGCACUUGAGACGGCCUGA 72.7%

dme-miR-313 UAUUGCACUUUUCACAGCCCGA 68.2%

**Sub-group dme-miR-92a, dme-miR-92b:**

1 22

dme-miR-92a CAUUGCACUUGUCCCGGCCUAU

dme-miR-92b AAUUGCACUAGUCCCGGCCUGC 81.8%

**miR-263a: dme-miR-263a, dme-miR-263b**

1 24

dme-miR-263a GUUAAUGGCACUGGAAGAAUUCAC

dme-miR-263b ---CUUGGCACUGGGAGAAUUCAC 75.0%

**miR-276a: dme-miR-276a, dme-miR-276b**

1 22

dme-miR-276a UAGGAACUUCAUACCGUGCUCU

dme-miR-276b UAGGAACUUAAUACCGUGCUCU 95.5%

**miR-281-1*: dme-miR-281-1*, dme-miR-281-2***

1 22

dme-miR-281-1* AAGAGAGCUGUCCGUCGACAGU

dme-miR-281-2* AAGAGAGCUAUCCGUCGACAGU 95.5%

**miR-954: dme-miR-954, dme-miR-966**

1 21

dme-miR-954 UCUGGGUGUUGCGUUGUGUGU

dme-miR-966 UGUGGGUUGUGGGCUGUGUGG 71.4%

**miR-998: dme-miR-285, dme-miR-995, dme-miR-998**

1 22

dme-miR-998 UAGCACCAUGAGAU-UCAGCUC

dme-miR-995 UAGCACCACAUGAU-UCGGCUU 76.2%

dme-miR-285 UAGCACCAUUCGAAAUCAGUGC 72.7%

**miR-1009: dme-miR-1009, dme-miR-1010**

1 24

dme-miR-1009 UCUCAAAAAUUGUUACAUUU-CAG

dme-miR-1010 UUUCACCUAUCGUUCCAUUUGCAG 70.8%

**dme-miR-iab-4-3p: dme-miR-iab-4-3p, dme-miR-iab4as-3p**

1 24

dme-miR-iab-4-3p CGGUAUACCUUCAGUAUACGUAAC

dme-miR-iab4as-3p --GGAUACAUUCAGUAUACGUUUA 70.8%

**dme-miR-iab-4-5p: dme-miR-iab-4-5p, dme-miR-iab4as-5p**

1 24

dme-miR-iab-4-5p --ACGUAUACUGAAUGUAUCCUGA

dme-miR-iab4as-5p UUACGUAUACUGAAGGUAUACCG- 75.0%
