# Supplementary material for: Sequence Relationships among C. elegans, D. melanogaster and Human microRNAs Highlight the Extensive Conservation of microRNAs in Biology
Source: PLoS One. 2008 Jul 30;3(7):e2818. doi: 10.1371/journal.pone.0002818 (PMC2486268; doi:10.1371/journal.pone.0002818)
Supplement: Dataset S6 — Table and alignments of D. melanogaster miRNA sequences with 60–69.9% similarity. (0.15 MB DOC) [file pone.0002818.s010.doc]

**Supplementary Table S6: Searches for an overall similarity of 60-69.9% detects 61 sequence relationships between 77 *D. melanogaster* miRNAs.** 56 of these miRNAs are not related in sequence to other miRNAs above the ≥70% threshold (Dataset S5). Significantly, 25 of the 61 sequence relationships are homologous over 7 continuous nt at the 5’ end (Dataset S4, sequence alignments below).

| **miRNA**  **Group ID** | **Distantly Related *D. melanogaster* miRNAs** | | **Overall Identity**  **(60-69.9)** |
| --- | --- | --- | --- |
| **let-7** | dme-let-7 | dme-miR-984 | 69.6 |
|  |  | dme-miR-991 | 68.2 |
|  |  | dme-miR-977 | 68.2 |
|  |  | dme-miR-960 | 63.6 |
|  |  | dme-miR-966 | 63.6 |
| **miR-1** | dme-miR-1 | dme-miR-989 | 60.9 |
| **miR-4** | dme-miR-4 | dme-miR-79 | 65.2 |
| **miR-7** | dme-miR-7 | dme-miR-34 | 60.9 |
| **miR-10** | dme-miR-10 | dme-miR-125 | 69.6 |
| **miR-11** | dme-miR-11 | dme-miR-6 | 63.6 |
|  |  | dme-miR-276* | 60.9 |
| **miR-33** | dme-miR-33 | dme-miR-974 | 60.9 |
| **miR-100** | dme-miR-100 | dme-miR-125 | 65.2 |
| **miR-124** | dme-miR-124 | dme-miR-306 | 60.9 |
| **miR-275** | dme-miR-275 | dme-miR-306 | 62.5 |
| **miR-276*** | dme-miR-276* | dme-miR-309 | 60.0 |
| **miR-279** | dme-miR-279 | dme-miR-996 | 65.2 |
| **miR-280** | dme-miR-280 | dme-miR-303 | 60.7 |
| **miR-283** | dme-miR-283 | dme-miR-12 | 60.9 |
|  |  | dme-miR-277 | 60.9 |
| **miR-285** | dme-miR-285 | dme-miR-31b | 60.9 |
|  |  | dme-miR-983 | 60.9 |
| **miR-286** | dme-miR-286 | dme-miR-279 | 69.6 |
|  |  | dme-miR-996 | 69.6 |
|  |  | dme-miR-125 | 62.5 |
|  |  | dme-miR-970 | 60.0 |
| **miR-305** | dme-miR-305 | dme-miR-276a | 65.2 |
|  |  | dme-miR-12 | 60.9 |
|  |  | dme-miR-276b | 60.9 |
|  |  | dme-miR-311 | 60.0 |
| **miR-307** | dme-miR-307 | dme-miR-13a | 65.2 |
|  |  | dme-miR-2a | 60.9 |
|  |  | dme-miR-13b | 60.9 |
| **miR-308** | dme-miR-308 | dme-miR-13a | 60.9 |
|  |  | dme-miR-304 | 60.9 |
|  |  | dme-miR-1007 | 60.0 |
| **miR-309** | dme-miR-309 | dme-miR-318 | 63.6 |
| **miR-310** | dme-miR-310 | dme-miR-313 | 68.2 |
|  |  | dme-miR-312 | 63.6 |
| **miR-311** | dme-miR-311 | dme-miR-92a | 68.2 |
|  |  | dme-miR-92b | 68.2 |
| **miR-313** | dme-miR-313 | dme-miR-92a | 68.2 |
|  |  | dme-miR-92b | 68.2 |
| **miR-932** | dme-miR-932 | dme-miR-978 | 60.0 |
| **miR-963** | dme-miR-963 | dme-miR-984 | 63.0 |
| **miR-965** | dme-miR-965 | dme-miR-1012 | 62.5 |
| **miR-976** | dme-miR-976 | dme-miR-971 | 65.2 |
|  |  | dme-miR-315 | 60.9 |
| **miR-980** | dme-miR-980 | dme-miR-995 | 60.9 |
| **miR-987** | dme-miR-987 | dme-miR-991 | 60.0 |
| **miR-992** | dme-miR-992 | dme-miR-277 | 62.5 |
|  |  | dme-miR-983 | 62.5 |
| **miR-1003** | dme-miR-1003 | dme-miR-1004 | 63.6 |
| **miR-1006** | dme-miR-1006 | dme-miR-288 | 64.0 |
|  |  | dme-miR-304 | 60.9 |
|  |  | dme-miR-1014 | 60.9 |
| **miR-1009** | dme-miR-1009 | dme-miR-1014 | 61.5 |
| **miR-1011** | dme-miR-1011 | dme-miR-137 | 60.9 |
|  |  | dme-miR-1015 | 60.9 |
| **miR-1016** | dme-miR-1016 | dme-miR-1010 | 66.7 |
|  |  | dme-miR-975 | 60.9 |

**Supplementary Alignments S6:**

**Aligned sequences of *D. melanogaster* miRNAs with 60-69.9% overall sequence similarity.** Identity between *D. melanogaster* miRNAs is given in percentage at the end of each alignment. Grey shading denotes potential G..U pairing.

**let-7: dme-let-7, dme-miR-960, dme-miR-966,**

**dme-miR-977, dme-miR-984, dme-miR-991**

1 23

dme-let-7 UGAGGUAG-UA-GGUUGUAUAGU

dme-miR-984 UGAGGUAAAUACGGUUGGAAUUU 69.6%

1 22

dme-let-7 -UGAGGUAGUAGGUUGUAUAGU

dme-miR-991 UUAAAGUUGUAGUUUGGAAAGU 68.2%

1 22

dme-let-7 UGAGGUAGU-AGGUUGUAUAGU

dme-miR-977 UGAGAUAUUCACGUUGUCUAA- 68.2%

1 22

dme-let-7 UGAGG-UAGUAGGUUGUAUAGU

dme-miR-960 UGAGUAUUCCAGAUUGCAUAGC 63.6%

1 22

dme-let-7 UGAGG-UAGUAGGUUGUAUAGU

dme-miR-966 UGUGGGUUGUGGGCUGUGUGG- 63.6%

**miR-1: dme-miR-1, dme-miR-989**

1 23

dme-miR-1 UGGAAUGUAAAGAAGUAUGGAG-

dme-miR-989 UGUGAUGUGACGUAGU--GGAAC 60.9%

**miR-4: dme-miR-4, dme-miR-79**

1 23

dme-miR-4 AUAAAGCUAGACAACCAUUGA--

dme-miR-79 –UAAAGCUAGAUUACCAAAGCAU 65.2%

**miR-7: dme-miR-7, dme-miR-34**

1 23

dme-miR-7 UGGAAGACUAGUGAUUUUGUUGU

dme-miR-34 UGGCAGUGUGGUUAGCUGGUUG- 60.9%

**miR-10: dme-miR-10, dme-miR-125**

1 23

dme-miR-10 ACCCUGUAGAUCCGAAUUUGU--

dme-miR-125 UCCCUG-AGACCCUAACUUGUGA 69.6%

**miR-11: dme-miR-11, dme-miR-6, dme-miR-276***

1 22

dme-miR-11 CAUCACAGUCUGAGUUCUUGC-

dme-miR-6 UAUCACAGUGGCUGUUCUUUUU 63.6%

1 23

dme-miR-11 CAUCACAGUCU-GAGUUCUUGC-

dme-miR-276* CAGCGAGGUAUAGAGUUCCUACG 60.9%

**miR-33: dme-miR-33, dme-miR-974**

1 23

dme-miR-33 -AG-GUGCAUUGUAGUCGCAUUG

dme-miR-974 AAGCGAGCAAAGAAGUAGUAUU- 60.9%

**miR-100: dme-miR-100, dme-miR-125**

1 23

dme-miR-100 AACCCGUAAAUCCGAACUUGUG-

dme-miR-125 –UCCCUGAGACCCUAACUUGUGA 65.2%

**miR-124: dme-miR-124, dme-miR-306**

1 23

dme-miR-124 UAAGGCACGCGGUGAAUGCCAAG

dme-miR-306 UCAGGUACUUAGUGACUCUCAA- 60.9%

**miR-275: dme-miR-275, dme-miR-306**

1 24

dme-miR-275 UCAGGUACCUGAAGUAGCGCGCG-

dme-miR-306 UCAGGUACUU—-AGUGACUCUCAA 62.5%

**miR-276*: dme-miR-276*, dme-miR-309**

1 25

dme-miR-276* -CAGCGAGGUAUAGAGU-UCCUACG

dme-miR-309 GCACUG-GGUAAAGUUUGUCCUA-- 60.0%

**miR-279: dme-miR-279, dme-miR-996**

1 23

dme-miR-279 UGACUAGAUC-CACACUCAUUAA

dme-miR-996 UGACUAGAUUUCAUGCUCGUCU- 65.2%

**miR-280: dme-miR-280, dme-miR-303**

1 28

dme-miR-280 UGUAUUUACGUUGCAUAUGAAA-UGAUA

dme-miR-303 ----UUUAGGUUUCACAGGAAACUGGU- 60.7%

**miR-283: dme-miR-283, dme-miR-12, dme-miR-277**

1 23

dme-miR-283 UAAAUAU--CAGCUGGUAAUUCU

dme-miR-12 UGAGUAUUACAUCAGGUACUGGU 60.9%

1 23

dme-miR-283 UAAAU--AUCAGCUGGUAAUUCU

dme-miR-277 UAAAUGCACUAUCUGGUACGACA 60.9%

**miR-285: dme-miR-285, dme-miR-31b, dme-miR-983**

1 23

dme-miR-285 UAGCACCAU-UCGAAAUCAGUGC

dme-miR-31b UGGCAAGAUGUCGGAAUAGCUG- 60.9%

1 23

dme-miR-285 -UAGCACCAUUCGAAAUCAGUGC

dme-miR-983 AUAAUACGUUUCGAACU-AAUGA 60.9%

**miR-286: dme-miR-286, dme-miR-125, dme-miR-279,**

**dme-miR-970, dme-miR-996**

1 23

dme-miR-286 UGACUAGACCGAACACUCGUGCU

dme-miR-279 UGACUAGAUCCA-CACUCAUUAA 69.6%

1 23

dme-miR-286 UGACUAGACCGAACACUCGUGCU

dme-miR-996 UGACUAGAUUUCAUGCUCGU-CU 69.6%

1 24

dme-miR-286 UGACU-AGACCGAACACUCGUGCU

dme-miR-125 UCCCUGAGACCCUA-ACUUGUGA- 62.5%

1 25

dme-miR-286 UGACUAGACCGAACACUCGUGCU--

dme-miR-970 UCAUAAGAC---ACACGCG-GCUAU 60.0%

**miR-305: dme-miR-305, dme-miR-12, dme-miR-276a,**

**dme-miR-276b, dme-miR-311**

1 23

dme-miR-276a UAGGAACUUCAUACCGUGCUCU-

dme-miR-305 AUUGUACUUCAUCAGGUGCUCUG 65.2%

1 23

dme-miR-12 UGAGUAUUACAUCAGGUACUGGU

dme-miR-305 AUUGUACUUCAUCAGGUGCUCUG 60.9%

1 23

dme-miR-276b UAGGAACUUAAUACCGUGCUCU-

dme-miR-305 AUUGUACUUCAUCAGGUGCUCUG 60.9%

1 25

dme-miR-305 -AUUGUACUUCAUCAGGUGCUCUG-

dme-miR-311 UAUUGCACAU--UCACCGGC-CUGA 60.0%

**miR-307: dme-miR-307, dme-miR-2a, dme-miR-13a,**

**dme-miR-13b**

1 23

dme-miR-13a UAUCACAGCCAUUUUGA-UGAGU

dme-miR-307 --UCACAACCUCCUUGAGUGAG- 65.2%

1 23

dme-miR-2a UAUCACAGCCAGCUUUGAUGAGC

dme-miR-307 --UCACAACCUCCUUGAGUGAG- 60.9%

1 23

dme-miR-13b UAUCACAGCCAUUUUGAC-GAGU

dme-miR-307 --UCACAACCUCCUUGAGUGAG- 60.9%

**miR-308: dme-miR-308, dme-miR-13a, dme-miR-304,**

**dme-miR-1007**

1 23

dme-miR-13a UAUCACAGCCAUUUUGAUGAGU-

dme-miR-308 AAUCACAGG-AUUAUACUGUGAG 60.9%

1 23

dme-miR-304 UAAUCUCAAUUUGUAAAUGUGAG

dme-miR-308 –AAUCACAGGAUUAUACUGUGAG 60.9%

1 25

dme-miR-1007 UAAGCUCA--AUUA-ACUGUUUGCA

dme-miR-308 -AAUCACAGGAUUAUACUGUGAG-- 60.0%

**miR-309: dme-miR-309, dme-miR-318**

1 22

dme-miR-309 GCACUGGGUAAAGUUUGUCCUA

dme-miR-318 UCACUGGGCUUUGUUUAUCUCA 63.6%

**miR-310: dme-miR-310, dme-miR-312, dme-miR-313**

1 22

dme-miR-310 UAUUGCACACUUCCCGGCCUUU

dme-miR-313 UAUUGCACUUUUCACAGCCCGA 68.2%

1 22

dme-miR-310 UAUUGCACACUUCCCGGCCUUU

dme-miR-312 UAUUGCACUUGAGACGGCCUGA 63.6%

**miR-311: dme-miR-311, dme-miR-92a, dme-miR-92b**

1 22

dme-miR-311 UAUUGCACAUUCACCGGCCUGA

dme-miR-92a CAUUGCACUUGUCCCGGCCUAU 68.2%

1 22

dme-miR-311 UAUUGCACAUUCACCGGCCUGA

dme-miR-92b AAUUGCACUAGUCCCGGCCUGC 68.2%

**miR-313: dme-miR-313, dme-miR-92a, dme-miR-92b**

1 22

dme-miR-313 UAUUGCACUUUUCACAGCCCGA

dme-miR-92a CAUUGCACUUGUCCCGGCCUAU 68.2%

1 22

dme-miR-313 UAUUGCACUUUUCACAGCCCGA

dme-miR-92b AAUUGCACUAGUCCCGGCCUGC 68.2%

**miR-932: dme-miR-932, dme-miR-978**

1 25

dme-miR-932 ---UCAAUUCCGUAGUGCAUUGCAG

dme-miR-978 UGUCCAGUGCCGUAA---AUUGCAG 60.0%

**miR-963: dme-miR-963, dme-miR-984**

1 27

dme-miR-963 ACAAGGUAAAUAUCAGGUUG---UUUC

dme-miR-984 -UGAGGUAAAUAC--GGUUGGAAUUU- 63.0%

**miR-965: dme-miR-965, dme-miR-1012**

1 24

dme-miR-965 UAAGCGUAUAGCUUUUCCCCUU--

dme-miR-1012 UUAGUCAA-AGAUUUUCCCCAUAG 62.5%

**miR-976: dme-miR-976, dme-miR-315, dme-miR-971**

1 23

dme-miR-976 UUGGA-UUAGUUAUCAUCAAUGC

dme-miR-971 UUGGUGUUACUUCUUA-CAGUGA 65.2%

1 23

dme-miR-976 -UUGGAUUAGUUAUCAUCAAUGC

dme-miR-315 UUUUGAUUGUUGCUCAGAAA-GC 60.9%

**miR-980: dme-miR-980, dme-miR-995**

1 23

dme-miR-980 UAGCUGCCUUGUGAAG-GGCUUA

dme-miR-995 UAGCA-CCACAUGAUUCGGCUU- 60.9%

**miR-987: dme-miR-987, dme-miR-991**

1 25

dme-miR-987 -UAAAGUAAAUAGUCUGGAUUGAUG

dme-miR-991 UUAAAGUUG-UAGUUUGGAAAGU-- 60.0%

**miR-992: dme-miR-992, dme-miR-277, dme-miR-983**

1 24

dme-miR-992 --AGUACACGUUUCUGGUACUAAG

dme-miR-277 UAAAUGCAC-UAUCUGGUACGACA 62.5%

1 24

dme-miR-992 AGUACACGUUUCUGGUACUAAG--

dme-miR-983 AUAAUACGUUUC--GAACUAAUGA 62.5%

**miR-1003: dme-miR-1003, dme-miR-1004**

1 22

dme-miR-1003 UCUCACAUUUACAUAUUCACAG

dme-miR-1004 UCUCACAUCACUUCCCUCACAG 63.6%

**miR-1006: dme-miR-1006, dme-miR-288, dme-miR-304,**

**dme-miR-1014**

1 25

dme-miR-1006 --UAAAUUCGAUUUCUUAUUCAUAG

dme-miR-288 UUUCAUGUCGAUUUCAU-UUCAUG- 64.0%

1 23

dme-miR-1006 UAAAUUCGAUUUCUUAUUCAUAG

dme-miR-304 UAAUCUCAAUUUGUAAAUGUGAG 60.9%

1 23

dme-miR-1006 UAAAUUCGAUUUCUUAUUCAUAG

dme-miR-1014 AAAAUUCAUUUUCAUUUGCAG-- 60.9%

**miR-1009: dme-miR-1009, dme-miR-1014**

1 26

dme-miR-1009 UCUCAAAAAUUG--UUACAUUU-CAG

dme-miR-1014 -----AAAAUUCAUUUUCAUUUGCAG 61.5%

**miR-1011: dme-miR-1011, dme-miR-137, dme-miR-1015**

1 23

dme-miR-1011 UUAUUGGUUCAAAUCGCUCGCAG

dme-miR-137 -UAUUGCUUGAGAAUACACGUAG 60.9%

1 23

dme-miR-1011 UUAUUGGUUCAAAUCGCUCGCAG

dme-miR-1015 -UCCUGGGACAUCUCUCUUGCAG 60.9%

**miR-1016: dme-miR-1016, dme-miR-1010, dme-miR-975**

1 24

dme-miR-1016 -UUCACCUCUC--UCCAUACUUAG

dme-miR-1010 UUUCACCUAUCGUUCCAUUUGCAG 66.7%

1 23

dme-miR-1016 UUCAC-CUCUCUCCAUACU-UAG

dme-miR-975 UAAACACUUCCUACAUCCUGUAU 60.9%
