# Supplementary material for: Sequence Relationships among C. elegans, D. melanogaster and Human microRNAs Highlight the Extensive Conservation of microRNAs in Biology
Source: PLoS One. 2008 Jul 30;3(7):e2818. doi: 10.1371/journal.pone.0002818 (PMC2486268; doi:10.1371/journal.pone.0002818)
Supplement: Dataset S7 — Homology table and alignments of C. elegans miRNAs related at the 5′ end to Drosophila miRNAs. (0.32 MB DOC) [file pone.0002818.s011.doc]

**Supplementary Table S7: Analysis of 5’ end sequences identifies 176 relationships between 87 *C. elegans* miRNAs and 62 *D. melanogaster* miRNAs.** Superscript “less than” (**<**) symbol before miRNA names indicates allowed A-G base changes (G..U pairing) interrupting the 5’ 7nt homology block that groups specific miRNAs into families (see alignments below). Homologous nucleotides grouping miRNAs into families are summarized in the table and detailed in alignments. **(5’)** identifies 68 miRNAs with high identity at the 5’ end but <70% overall similarity to some of their sequence-related miRNAs. Of these, 35 miRNAs (cel-miR-48, cel-miR-52, cel-miR-53, cel-miR-54, cel-miR-55, cel-miR-56, cel-miR-62, cel-miR-63, cel-miR-64, cel-miR-65, cel-miR-66, cel-miR-86, cel-miR-90, cel-miR-229, cel-miR-233, cel-miR-238, cel-miR-249, cel-miR-251, cel-miR-252, cel-miR-259, cel-miR-260, cel-miR-267, cel-miR-268, cel-miR-273, cel-miR-356, cel-miR-357, cel-miR-358, cel-miR-785, cel-miR-787, cel-miR-790, cel-miR-791, cel-miR-795, cel-miR-796, cel-miR-1018 and cel-miR-1022) are significantly related in sequence only at the 5’ end—they do not have ≥60% extended similarity with their 5’ *Drosophila* relatives and thus are not included in Datasets S8 and S9.

|  | **5’ End Sequence Related miRNAs** | |  |
| --- | --- | --- | --- |
| **miRNA Group ID** | ***C. elegans*** | ***D. melanogaster*** | **# Identical nt at 5' End (10nt)** |
| let-7  *G/AAGG/AUAA/G* | cel-let-7 **(5’)** | dme-let-7 | 10 |
|  |  | dme-miR-984 | 7 |
|  |  | **<**dme-miR-963 | 5 |
|  |  | **<**dme-miR-977 | 7 |
| lin-4 UCCCUGA | cel-lin-4 | dme-miR-125 | 10 |
| miR-1 UGGAAUG | cel-miR-1 | dme-miR-1 | 10 |
| miR-2 AUCACAG | cel-miR-2 **(5’)** | dme-miR-2a | 10 |
|  |  | dme-miR-2c | 10 |
|  |  | dme-miR-2b | 10 |
|  |  | dme-miR-13a | 10 |
|  |  | dme-miR-13b | 10 |
|  |  | dme-miR-6 | 8 |
|  |  | dme-miR-11 | 8 |
|  |  | dme-miR-308 | 7 |
| miR-34 GGCAGUG | cel-miR-34 | dme-miR-34 | 9 |
| miR-43 AUCACAG | cel-miR-43 **(5’)** | dme-miR-6 | 9 |
|  |  | dme-miR-2b | 8 |
|  |  | dme-miR-2a | 8 |
|  |  | dme-miR-2c | 8 |
|  |  | dme-miR-11 | 8 |
|  |  | dme-miR-13a | 8 |
|  |  | dme-miR-13b | 8 |
|  |  | dme-miR-308 | 7 |
| miR-44 UGACUAG | cel-miR-44 **(5’)** | dme-miR-279 | 8 |
|  |  | dme-miR-286 | 8 |
|  |  | dme-miR-996 | 8 |
| miR-45 UGACUAG | cel-miR-45 **(5’)** | dme-miR-279 | 8 |
|  |  | dme-miR-286 | 8 |
|  |  | dme-miR-996 | 8 |
| miR-46 UGUCAUG | cel-miR-46 | dme-miR-281 | 9 |
| miR-47  *UGUCAUG* | cel-miR-47 | dme-miR-281 | 9 |
| miR-48 (U)G/AAGG/AUA(G/A) | cel-miR-48 **(5’)** | dme-let-7 | 8 |
|  |  | **<**dme-miR-977 | 7 |
|  |  | dme-miR-984 | 7 |
|  |  | **<**dme-miR-963 | 5 |
| miR-49 AGCACCA | cel-miR-49 **(5’)** | dme-miR-995 | 8 |
|  |  | dme-miR-998 | 8 |
|  |  | dme-miR-285 | 7 |
| miR-50  *GAUAUGU* | cel-miR-50 | dme-miR-190 | 8 |
| miR-51 ACCCGUA | cel-miR-51 **(5’)** | dme-miR-100 | 7 |
| miR-52  *ACCCGUA* | cel-miR-52 **(5’)** | dme-miR-100 | 8 |
| miR-53  *ACCCGUA* | cel-miR-53 **(5’)** | dme-miR-100 | 8 |
| miR-54  *ACCCGUA* | cel-miR-54 **(5’)** | dme-miR-100 | 8 |
| miR-55  *ACCCGUA* | cel-miR-55 **(5’)** | dme-miR-100 | 8 |
| miR-56  *ACCCGUA* | cel-miR-56 **(5’)** | dme-miR-100 | 8 |
| miR-57  *ACCCUGU* | cel-miR-57 **(5’)** | dme-miR-10 | 9 |
| miR-58 GA/GGA/GUCG/A | cel-miR-58 **(5’)** | dme-bantam | 9 |
|  |  | **<**dme-miR-306* | 5 |
| miR-61 UGACUAG | cel-miR-61 **(5’)** | dme-miR-279 | 9 |
|  |  | dme-miR-286 | 9 |
|  |  | dme-miR-996 | 8 |
| miR-62  *GAUAUGU* | cel-miR-62 **(5’)** | dme-miR-190 | 7 |
| miR-63 UGA/GCACU | cel-miR-63 **(5’)** | **<**dme-miR-263b | 7 |
| miR-64  *UGA/GCACU* | cel-miR-64 **(5’)** | **<**dme-miR-263b | 7 |
| miR-65  *UGA/GCACU* | cel-miR-65 **(5’)** | **<**dme-miR-263b | 7 |
| miR-66  *UGA/GCACU* | cel-miR-66 **(5’)** | **<**dme-miR-263b | 8 |
| miR-67 UCACAAC | cel-miR-67 | dme-miR-307 | 10 |
| miR-72 GGCAAGA | cel-miR-72 | dme-miR-31a | 9 |
|  |  | dme-miR-31b | 9 |
| miR-73 UGGCAAG | cel-miR-73 **(5’)** | dme-miR-31a | 10 |
|  |  | dme-miR-31b | 10 |
| miR-74 UGGCAAG | cel-miR-74 **(5’)** | dme-miR-31a | 8 |
|  |  | dme-miR-31b | 8 |
| miR-75 AA/GAGCUA/G | cel-miR-75 **(5’)** | dme-miR-4 | 8 |
|  |  | dme-miR-79 | 8 |
|  |  | **<**dme-miR-281-2* | 6 |
|  |  | **<**dme-miR-281-1* | 5 |
| miR-76  *UUCGUUG* | cel-miR-76 | dme-miR-981 | 9 |
| miR-79 AA/GA/GG/ACUA/G | cel-miR-79 **(5’)** | dme-miR-4 | 10 |
|  |  | dme-miR-79 | 9 |
|  |  | **<**dme-miR-281-2* | 7 |
|  |  | **<**dme-miR-281-1* | 6 |
|  |  | **<**dme-miR-7 | 5 |
| miR-80 GA/GGA/GUCA | cel-miR-80 | dme-bantam | 10 |
|  |  | **<**dme-miR-306* | 6 |
| miR-81 GA/GGA/GUCA | cel-miR-81 | dme-bantam | 9 |
|  |  | **<**dme-miR-306* | 6 |
| miR-82 GA/GGA/GUCA | cel-miR-82 | dme-bantam | 9 |
|  |  | **<**dme-miR-306* | 6 |
| miR-83 UAGCACC | cel-miR-83 **(5’)** | dme-miR-285 | 9 |
|  |  | dme-miR-995 | 9 |
|  |  | dme-miR-998 | 9 |
| miR-84 (U)G/AAGG/AUA(G/A) | cel-miR-84 **(5’)** | dme-let-7 | 10 |
|  |  | **<**dme-miR-977 | 7 |
|  |  | dme-miR-984 | 7 |
|  |  | **<**dme-miR-963 | 5 |
| miR-86  *AAGUG/AAA* | cel-miR-86 **(5’)** | **<**dme-miR-987 | 7 |
| miR-87 UGAGCAA | cel-miR-87 | dme-miR-87 | 8 |
| miR-90  *GAUAUGU* | cel-miR-90 **(5’)** | dme-miR-190 | 8 |
| miR-124 UAAGGCA | cel-miR-124 | dme-miR-124 | 10 |
| miR-228  *(AA)UGGCA(CU)* | cel-miR-228 **(5’)** | dme-miR-263b | 8 |
|  |  | dme-miR-263a | 7 |
| miR-229  *(AA)UGG/ACA(CU)* | cel-miR-229 **(5’)** | **<**dme-miR-263b | 7 |
|  |  | **<**dme-miR-263a | 6 |
| miR-231  *AAGCUCG* | cel-miR-231 **(5’)** | dme-miR-993 | 8 |
| miR-232 UAAAUGC | cel-miR-232 **(5’)** | dme-miR-277 | 8 |
| miR-233 UUGAGCA | cel-miR-233 **(5’)** | dme-miR-87 | 8 |
| miR-234  *UAUUGCU* | cel-miR-234 | dme-miR-137 | 8 |
| miR-235 AUUGCAC | cel-miR-235 | dme-miR-310 | 9 |
|  |  | dme-miR-312 | 9 |
|  |  | dme-miR-313 | 9 |
|  |  | dme-miR-311 | 8 |
|  |  | dme-miR-92b | 8 |
|  |  | dme-miR-92a | 8 |
| miR-236 UAAUACU | cel-miR-236 | dme-miR-8 | 10 |
| miR-237 UCCCUGA | cel-miR-237 **(5’)** | dme-miR-125 | 9 |
| miR-238 UUGUACU | cel-miR-238 **(5’)** | dme-miR-305 | 8 |
| miR-239a  *UUGUACU* | cel-miR-239a **(5’)** | dme-miR-305 | 8 |
| miR-239b  *UUGUACU* | cel-miR-239b **(5’)** | dme-miR-305 | 8 |
| miR-240  *UACUGGC* | cel-miR-240 **(5’)** | dme-miR-193 | 8 |
| miR-241 (U)G/AAGG/AUA(G/A) | cel-miR-241 **(5’)** | dme-let-7 | 8 |
|  |  | dme-miR-984 | 8 |
|  |  | **<**dme-miR-977 | 7 |
|  |  | **<**dme-miR-963 | 5 |
| miR-244  *UCUUUGG* | cel-miR-244 **(5’)** | dme-miR-9a | 9 |
|  |  | dme-miR-9b | 8 |
|  |  | dme-miR-9c | 8 |
| miR-245 UUGGUCC | cel-miR-245 | dme-miR-133 | 9 |
| miR-247 UGACUAG | cel-miR-247 **(5’)** | dme-miR-279 | 9 |
|  |  | dme-miR-286 | 9 |
|  |  | dme-miR-996 | 8 |
| miR-249 UCACAGG | cel-miR-249 **(5’)** | dme-miR-308 | 8 |
| miR-250 AUCACAG | cel-miR-250 **(5’)** | dme-miR-11 | 9 |
|  |  | dme-miR-2a | 8 |
|  |  | dme-miR-2b | 8 |
|  |  | dme-miR-2c | 8 |
|  |  | dme-miR-6 | 8 |
|  |  | dme-miR-13a | 8 |
|  |  | dme-miR-13b | 8 |
|  |  | dme-miR-308 | 8 |
| miR-251  *UUAAGUA* | cel-miR-251 **(5’)** | dme-miR-1002 | 10 |
| miR-252  *UAAGUAG* | cel-miR-252 **(5’)** | dme-miR-1002 | 8 |
| miR-256 (UG)G/AAAUG(CA) | cel-miR-256 | dme-miR-1 | 8 |
|  |  | **<**dme-miR-277 | 6 |
| miR-259 AAUCUCA | cel-miR-259 **(5’)** | dme-miR-304 | 7 |
| miR-260  *GUGAUGU* | cel-miR-260 **(5’)** | dme-miR-989 | 8 |
| miR-266 GGCAAGA | cel-miR-266 **(5’)** | dme-miR-31a | 7 |
|  |  | dme-miR-31b | 7 |
| miR-267 CCCGUG/AA | cel-miR-267 **(5’)** | **<**dme-miR-100 | 7 |
| miR-268 GGCAAGA | cel-miR-268 **(5’)** | dme-miR-31b | 7 |
|  |  | dme-miR-31a | 7 |
| miR-269 GGCAAGA | cel-miR-269 **(5’)** | dme-miR-31a | 7 |
|  |  | dme-miR-31b | 7 |
| miR-273 G/ACCCGUA | cel-miR-273 **(5’)** | **<**dme-miR-100 | 6 |
| miR-356 UUGAGCA | cel-miR-356 **(5’)** | dme-miR-87 | 8 |
| miR-357 UAAAUGC | cel-miR-357 **(5’)** | dme-miR-277 | 7 |
| miR-358 UUGGUAU | cel-miR-358 **(5’)** | dme-miR-9c | 7 |
| miR-359 UCACUGG | cel-miR-359 **(5’)** | dme-miR-318 | 9 |
|  |  | dme-miR-3 | 8 |
| miR-785  *AAGUG/AAA* | cel-miR-785 **(5’)** | **<**dme-miR-987 | 7 |
| miR-787  *AAGCUCG* | cel-miR-787 **(5’)** | dme-miR-993 | 8 |
| miR-790 CUUGGCA | cel-miR-790 **(5’)** | dme-miR-263b | 9 |
| miR-791 UUGGCAC | cel-miR-791 **(5’)** | dme-miR-263b | 8 |
| miR-793 UGAGG/AUA | cel-miR-793 **(5’)** | dme-let-7 | 7 |
|  |  | **<**dme-miR-977 | 7 |
|  |  | dme-miR-984 | 7 |
| miR-794 (U)G/AAGG/AUA(A/G) | cel-miR-794 **(5’)** | dme-let-7 | 8 |
|  |  | **<**dme-miR-977 | 8 |
|  |  | dme-miR-984 | 8 |
|  |  | **<**dme-miR-963 | 6 |
| miR-795 (U)G/AAGG/AUA(G/A) | cel-miR-795 **(5’)** | dme-miR-984 | 9 |
|  |  | dme-let-7 | 8 |
|  |  | **<**dme-miR-977 | 6 |
|  |  | **<**dme-miR-963 | 6 |
| miR-796 UGGAAUG | cel-miR-796 **(5’)** | dme-miR-1 | 9 |
| miR-797  *UAUCACA* | cel-miR-797 **(5’)** | dme-miR-2a | 9 |
|  |  | dme-miR-2b | 9 |
|  |  | dme-miR-2c | 9 |
|  |  | dme-miR-13a | 9 |
|  |  | dme-miR-13b | 9 |
|  |  | dme-miR-6 | 8 |
|  |  | dme-miR-308 | 8 |
|  |  | dme-miR-11 | 7 |
| miR-1018  *GAGAUCA* | cel-miR-1018 **(5’)** | dme-bantam | 7 |
| miR-1022  *A/GA/GGA/GUCA* | cel-miR-1022 **(5’)** | dme-bantam | 8 |
|  |  | **<**dme-miR-306* | 5 |

**Supplementary Alignments: S7**

**5’ sequence alignments of *C. elegans* and *D. melanogaster* miRNAs with significant identity at the 5’ end (10nt).** Members of a group have ≥7 continuous nt of homology with at least one other group member. Nucleotides at the end of sequences indicate the number of residues identical to the reference miRNA (top of group alignment), which has the closest sequence to the group consensus sequence. Grey shading denotes potential G..U pairing. Superscript “less than” (**<**) symbol before miRNA names indicates allowed A-G base changes (G..U pairing) interrupting the 7nt homology block that groups miRNAs into families. Superscript (5’) indicates worm miRNAs with 5’ homology but weak extended identity (<70%) to some of their fly related sequences. 35 of these worm miRNAs (highlighted in blue) have <60% overall similarity with all their 5’-related fly miRNAs.

**let-7: cel-let-7 ^(5’)^, dme-let-7, <dme-miR-963, <dme-miR-977,**

**dme-miR-984**

1

cel-let-7 -UGAGGUAGUA

dme-let-7 -UGAGGUAGUA 10nt

dme-miR-984 -UGAGGUAAAU 7nt

dme-miR-963 ACAAGGUAAA- 5nt

dme-miR-977 -UGAGAUAUUC 7nt

**lin-4: cel-lin-4, dme-miR-125**

1 10

cel-lin-4 UCCCUGAGAC

dme-miR-125 UCCCUGAGAC 10nt

**miR-1: cel-miR-1, dme-miR-1**

1 10

cel-miR-1 UGGAAUGUAA

dme-miR-1 UGGAAUGUAA 10nt

**miR-2: cel-miR-2 ^(5’)^, dme-miR-2a, dme-miR-2b, dme-miR-2c,**

**dme-miR-6, dme-miR-11, dme-miR-13a,**

**dme-miR-13b, dme-miR-308**

1 10

cel-miR-2 UAUCACAGCC

dme-miR-2a UAUCACAGCC 10nt

dme-miR-2b UAUCACAGCC 10nt

dme-miR-2c UAUCACAGCC 10nt

dme-miR-13a UAUCACAGCC 10nt

dme-miR-13b UAUCACAGCC 10nt

dme-miR-11 CAUCACAGUC 8nt

dme-miR-6 UAUCACAGUG 8nt

dme-miR-308 AAUCACAGGA 7nt

**miR-34: cel-miR-34, dme-miR-34**

1 10

cel-miR-34 AGGCAGUGUG

dme-miR-34 UGGCAGUGUG 9nt

**miR-43: cel-miR-43 ^(5’)^, dme-miR-2a, dme-miR-2b,**

**dme-miR-2c, dme-miR-6, dme-miR-11,**

**dme-miR-13a, dme-miR-13b, dme-miR-308**

1 10

cel-miR-43 UAUCACAGUU

dme-miR-6 UAUCACAGUG 9nt

dme-miR-11 CAUCACAGUC 8nt

dme-miR-13a UAUCACAGCC 8nt

dme-miR-13b UAUCACAGCC 8nt

dme-miR-2a UAUCACAGCC 8nt

dme-miR-2b UAUCACAGCC 8nt

dme-miR-2c UAUCACAGCC 8nt

dme-miR-308 AAUCACAGGA 7nt

**miR-44: cel-miR-44 ^(5’)^, dme-miR-279, dme-miR-286,**

**dme-miR-996**

1 10

cel-miR-44 UGACUAGAGA

dme-miR-286 UGACUAGACC 8nt

dme-miR-279 UGACUAGAUC 8nt

dme-miR-996 UGACUAGAUU 8nt

**miR-45: cel-miR-45 ^(5’)^, dme-miR-279, dme-miR-286,**

**dme-miR-996**

1 10

cel-miR-45 UGACUAGAGA

dme-miR-286 UGACUAGACC 8nt

dme-miR-279 UGACUAGAUC 8nt

dme-miR-996 UGACUAGAUU 8nt

**miR-46: cel-miR-46, dme-miR-281**

1 10

cel-miR-46 UGUCAUGGAG

dme-miR-281 UGUCAUGGAA 9nt

**miR-47: cel-miR-47, dme-miR-281**

1 10

cel-miR-47 UGUCAUGGAG

dme-miR-281 UGUCAUGGAA 9nt

**miR-48: cel-miR-48 ^(5’)^, dme-let-7, <dme-miR-963,**

**<dme-miR-977, dme-miR-984**

1

cel-miR-48 -UGAGGUAGGC

dme-let-7 -UGAGGUAGUA 8nt

dme-miR-963 ACAAGGUAAA- 5nt

dme-miR-977 -UGAGAUAUUC 7nt

dme-miR-984 -UGAGGUAAAU 7nt

**miR-49: cel-miR-49 ^(5’)^, dme-miR-285, dme-miR-995,**

**dme-miR-998**

1 10

cel-miR-49 AAGCACCACG

dme-miR-285 UAGCACCAUU 7nt

dme-miR-998 UAGCACCAUG 8nt

dme-miR-995 UAGCACCACA 8nt

**miR-50: cel-miR-50, dme-miR-190**

1 10

cel-miR-50 UGAUAUGUCU

dme-miR-190 AGAUAUGUUU 8nt

**miR-51: cel-miR-51 ^(5’)^, dme-miR-100**

1 10

cel-miR-51 UACCCGUAGC

dme-miR-100 AACCCGUAAA 7nt

**miR-52: cel-miR-52 ^(5’)^, dme-miR-100**

1 10

cel-miR-52 CACCCGUACA

dme-miR-100 AACCCGUAAA 8nt

**miR-53: cel-miR-53 ^(5’)^, dme-miR-100**

1 10

cel-miR-53 CACCCGUACA

dme-miR-100 AACCCGUAAA 8nt

**miR-54: cel-miR-54 ^(5’)^, dme-miR-100**

1 10

cel-miR-54 UACCCGUAAU

dme-miR-100 AACCCGUAAA 8nt

**miR-55: cel-miR-55 ^(5’)^, dme-miR-100**

1 10

cel-miR-55 UACCCGUAUA

dme-miR-100 AACCCGUAAA 8nt

**miR-56: cel-miR-56 ^(5’)^, dme-miR-100**

1 10

cel-miR-56 UACCCGUAAU

dme-miR-100 AACCCGUAAA 8nt

**miR-57: cel-miR-57 ^(5’)^, dme-miR-10**

1

cel-miR-57 UACCCUGUAG-

dme-miR-10 -ACCCUGUAGA 9nt

**miR-58: cel-miR-58 ^(5’)^, dme-bantam, <dme-miR-306***

1 10

cel-miR-58 UGAGAUCGUU

dme-bantam UGAGAUCAUU 9nt

dme-miR-306* GGGGGUCACU 5nt

**miR-61: cel-miR-61 ^(5’)^, dme-miR-279, dme-miR-286,**

**dme-miR-996**

1 10

cel-miR-61 UGACUAGAAC

dme-miR-286 UGACUAGACC 9nt

dme-miR-279 UGACUAGAUC 9nt

dme-miR-996 UGACUAGAUU 8nt

**miR-62: cel-miR-62 ^(5’)^, dme-miR-190**

1 10

cel-miR-62 UGAUAUGUAA

dme-miR-190 AGAUAUGUUU 7nt

**miR-63: cel-miR-63 ^(5’)^, <dme-miR-263b**

1 10

cel-miR-63 UAUGACACUG

dme-miR-263b CUUGGCACUG 7nt

**miR-64: cel-miR-64 ^(5’)^, <dme-miR-263b**

1 10

cel-miR-64 UAUGACACUG

dme-miR-263b CUUGGCACUG 7nt

**miR-65: cel-miR-65 ^(5’)^, <dme-miR-263b**

1 10

cel-miR-65 UAUGACACUG

dme-miR-263b CUUGGCACUG 7nt

**miR-66: cel-miR-66 ^(5’)^, <dme-miR-263b**

1 10

cel-miR-66 CAUGACACUG

dme-miR-263b CUUGGCACUG 8nt

**miR-67: cel-miR-67, dme-miR-307**

1 10

cel-miR-67 UCACAACCUC

dme-miR-307 UCACAACCUC 10nt

**miR-72: cel-miR-72, dme-miR-31a, dme-miR-31b**

1 10

cel-miR-72 AGGCAAGAUG

dme-miR-31a UGGCAAGAUG 9nt

dme-miR-31b UGGCAAGAUG 9nt

**miR-73: cel-miR-73 ^(5’)^, dme-miR-31a, dme-miR-31b**

1 10

cel-miR-73 UGGCAAGAUG

dme-miR-31a UGGCAAGAUG 10nt

dme-miR-31b UGGCAAGAUG 10nt

**miR-74: cel-miR-74 ^(5’)^, dme-miR-31a, dme-miR-31b**

1 10

cel-miR-74 UGGCAAGAAA

dme-miR-31a UGGCAAGAUG 8nt

dme-miR-31b UGGCAAGAUG 8nt

**miR-75: cel-miR-75 ^(5’)^, dme-miR-4, dme-miR-79,**

**<dme-miR-281-1*, <dme-miR-281-2***

1

cel-miR-75 -UUAAAGCUAC-

dme-miR-4 -AUAAAGCUAG- 8nt

dme-miR-79 --UAAAGCUAGA 8nt

dme-miR-281-1* AAGAGAGCUG-- 5nt

dme-miR-281-2* AAGAGAGCUA-- 6nt

**miR-76: cel-miR-76, dme-miR-981**

1 10

cel-miR-76 UUCGUUGUUG

dme-miR-981 UUCGUUGUCG 9nt

**miR-79: cel-miR-79 ^(5’)^, dme-miR-4, <dme-miR-7,**

**dme-miR-79,<dme-miR-281-1*, <dme-miR-281-2***

1

cel-miR-79 -AUAAAGCUAG-

dme-miR-4 -AUAAAGCUAG- 10nt

dme-miR-79 --UAAAGCUAGA 9nt

dme-miR-7 UGGAAGACUA-- 5nt

dme-miR-281-1* AAGAGAGCUG-- 7nt

dme-miR-281-2* AAGAGAGCUA-- 7nt

**miR-80: cel-miR-80, dme-bantam, <dme-miR-306***

1 10

cel-miR-80 UGAGAUCAUU

dme-bantam UGAGAUCAUU 10nt

dme-miR-306* GGGGGUCACU 6nt

**miR-81: cel-miR-81, dme-bantam, <dme-miR-306***

1 10

cel-miR-81 UGAGAUCAUC

dme-bantam UGAGAUCAUU 9nt

dme-miR-306* GGGGGUCACU 6nt

**miR-82: cel-miR-82, dme-bantam, <dme-miR-306***

1 10

cel-miR-82 UGAGAUCAUC

dme-bantam UGAGAUCAUU 9nt

dme-miR-306* GGGGGUCACU 6nt

**miR-83: cel-miR-83 ^(5’)^, dme-miR-285, dme-miR-995,**

**dme-miR-998**

1 10

cel-miR-83 UAGCACCAUA

dme-miR-995 UAGCACCACA 9nt

dme-miR-285 UAGCACCAUU 9nt

dme-miR-998 UAGCACCAUG 9nt

**miR-84: cel-miR-84 ^(5’)^, dme-let-7, <dme-miR-963,**

**<dme-miR-977, dme-miR-984**

1

cel-miR-84 -UGAGGUAGUA

dme-let-7 -UGAGGUAGUA 10nt

dme-miR-963 ACAAGGUAAA- 5nt

dme-miR-984 -UGAGGUAAAU 7nt

dme-miR-977 -UGAGAUAUUC 7nt

**miR-86: cel-miR-86 ^(5’)^, <dme-miR-987**

1

cel-miR-86 -UAAGUGAAUG

dme-miR-987 UAAAGUAAAU- 7nt

**miR-87: cel-miR-87, dme-miR-87**

1 10

cel-miR-87 GUGAGCAAAG

dme-miR-87 UUGAGCAAAA 8nt

**miR-90: cel-miR-90 ^(5’)^, dme-miR-190**

1 10

cel-miR-90 UGAUAUGUUG

dme-miR-190 AGAUAUGUUU 8nt

**miR-124: cel-miR-124, dme-miR-124**

1 10

cel-miR-124 UAAGGCACGC

dme-miR-124 UAAGGCACGC 10nt

**miR-228: cel-miR-228 ^(5’)^, dme-miR-263a, dme-miR-263b**

1

cel-miR-228 ---AAUGGCACUG

dme-miR-263a GUUAAUGGCA--- 7nt

dme-miR-263b ---CUUGGCACUG 8nt

**miR-229: cel-miR-229 ^(5’)^, <dme-miR-263a, <dme-miR-263b**

1

cel-miR-229 ---AAUGACACUG

dme-miR-263a GUUAAUGGCA--- 6nt

dme-miR-263b ---CUUGGCACUG 7nt

**miR-231: cel-miR-231 ^(5’)^, dme-miR-993**

1 10

cel-miR-231 UAAGCUCGUG

dme-miR-993 GAAGCUCGUC 8nt

**miR-232: cel-miR-232 ^(5’)^, dme-miR-277**

1 10

cel-miR-232 UAAAUGCAUC

dme-miR-277 UAAAUGCACU 8nt

**miR-233: cel-miR-233 ^(5’)^, dme-miR-87**

1 10

cel-miR-233 UUGAGCAAUG

dme-miR-87 UUGAGCAAAA 8nt

**miR-234: cel-miR-234, dme-miR-137**

1 10

cel-miR-234 UUAUUGCUCG-

dme-miR-137 -UAUUGCUUGA 8nt

**miR-235: cel-miR-235, dme-miR-92a, dme-miR-92b,**

**dme-miR-310, dme-miR-311, dme-miR-312,**

**dme-miR-313**

1 10

cel-miR-235 UAUUGCACUC

dme-miR-310 UAUUGCACAC 9nt

dme-miR-311 UAUUGCACAU 8nt

dme-miR-312 UAUUGCACUU 9nt

dme-miR-313 UAUUGCACUU 9nt

dme-miR-92a CAUUGCACUU 8nt

dme-miR-92b AAUUGCACUA 8nt

**miR-236: cel-miR-236, dme-miR-8**

1 10

cel-miR-236 UAAUACUGUC

dme-miR-8 UAAUACUGUC 10nt

**miR-237: cel-miR-237 ^(5’)^, dme-miR-125**

1 10

cel-miR-237 UCCCUGAGAA

dme-miR-125 UCCCUGAGAC 9nt

**miR-238: cel-miR-238 ^(5’)^, dme-miR-305**

1 10

cel-miR-238 UUUGUACUCC

dme-miR-305 AUUGUACUUC 8nt

**miR-239a: cel-miR-239a ^(5’)^, dme-miR-305**

1 10

cel-miR-239a UUUGUACUAC

dme-miR-305 AUUGUACUUC 8nt

**miR-239b: cel-miR-239b ^(5’)^, dme-miR-305**

1 10

cel-miR-239b UUUGUACUAC

dme-miR-305 AUUGUACUUC 8nt

**miR-240: cel-miR-240 ^(5’)^, dme-miR-193**

1 10

cel-miR-240 UACUGGCCCC

dme-miR-193 UACUGGCCUA 8nt

**miR-241: cel-miR-241 ^(5’)^, dme-let-7, <dme-miR-963, <dme-miR-977, dme-miR-984**

1

cel-miR-241 -UGAGGUAGGU

dme-let-7 -UGAGGUAGUA 8nt

dme-miR-963 ACAAGGUAAA- 5nt

dme-miR-977 -UGAGAUAUUC 7nt

dme-miR-984 -UGAGGUAAAU 8nt

**miR-244: cel-miR-244 ^(5’)^, dme-miR-9a, dme-miR-9b,**

**dme-miR-9c**

1 10

cel-miR-244 UCUUUGGUUG

dme-miR-9a UCUUUGGUUA 9nt

dme-miR-9b UCUUUGGUGA 8nt

dme-miR-9c UCUUUGGUAU 8nt

**miR-245: cel-miR-245, dme-miR-133**

1

cel-miR-245 AUUGGUCCCC-

dme-miR-133 -UUGGUCCCCU 9nt

**miR-247: cel-miR-247 ^(5’)^, dme-miR-279, dme-miR-286,**

**dme-miR-996**

1 10

cel-miR-247 UGACUAGAGC

dme-miR-286 UGACUAGACC 9nt

dme-miR-279 UGACUAGAUC 9nt

dme-miR-996 UGACUAGAUU 8nt

**miR-249: cel-miR-249 ^(5’)^, dme-miR-308**

1

cel-miR-249 --UCACAGGACU

dme-miR-308 AAUCACAGGA-- 8nt

**miR-250: cel-miR-250 ^(5’)^, dme-miR-2a, dme-miR-2b,**

**dme-miR-2c, dme-miR-6, dme-miR-11,**

**dme-miR-13a, dme-miR-13b, dme-miR-308**

1 10

cel-miR-250 AAUCACAGUC

dme-miR-11 CAUCACAGUC 9nt

dme-miR-308 AAUCACAGGA 8nt

dme-miR-6 UAUCACAGUG 8nt

dme-miR-13a UAUCACAGCC 8nt

dme-miR-13b UAUCACAGCC 8nt

dme-miR-2a UAUCACAGCC 8nt

dme-miR-2b UAUCACAGCC 8nt

dme-miR-2c UAUCACAGCC 8nt

**miR-251: cel-miR-251 ^(5’)^, dme-miR-1002**

1 10

cel-miR-251 UUAAGUAGUG

dme-miR-1002 UUAAGUAGUG 10nt

**miR-252: cel-miR-252 ^(5’)^, dme-miR-1002**

1 10

cel-miR-252 AUAAGUAGUA

dme-miR-1002 UUAAGUAGUG 8nt

**miR-256: cel-miR-256, dme-miR-1, <dme-miR-277**

1

cel-miR-256 UGGAAUGCAU-

dme-miR-1 UGGAAUGUAA- 8nt

dme-miR-277 -UAAAUGCACU 6nt

**miR-259: cel-miR-259 ^(5’)^, dme-miR-304**

1 10

cel-miR-259 AAAUCUCAUC

dme-miR-304 UAAUCUCAAU 7nt

**miR-260: cel-miR-260 ^(5’)^, dme-miR-989**

1

cel-miR-260 -GUGAUGUCGA

dme-miR-989 UGUGAUGU-GA 8nt

**miR-266: cel-miR-266 ^(5’)^, dme-miR-31a, dme-miR-31b**

1 10

cel-miR-266 AGGCAAGACU

dme-miR-31a UGGCAAGAUG 7nt

dme-miR-31b UGGCAAGAUG 7nt

**miR-267: cel-miR-267 ^(5’)^, <dme-miR-100**

1

cel-miR-267 --CCCGUGAAGU

dme-miR-100 AACCCGUAAA-- 7nt

**miR-268: cel-miR-268 ^(5’)^, dme-miR-31a, dme-miR-31b**

1

cel-miR-268 -GGCAAGAAUU

dme-miR-31a UGGCAAGAUG- 7nt

dme-miR-31b UGGCAAGAUG- 7nt

**miR-269: cel-miR-269 ^(5’)^, dme-miR-31a, dme-miR-31b**

1

cel-miR-269 -GGCAAGACUC

dme-miR-31a UGGCAAGAUG- 7nt

dme-miR-31b UGGCAAGAUG- 7nt

**miR-273: cel-miR-273 ^(5’)^, <dme-miR-100**

1 10

cel-miR-273 UGCCCGUACU

dme-miR-100 AACCCGUAAA 6nt

**miR-356: cel-miR-356 ^(5’)^, dme-miR-87**

1 10

cel-miR-356 UUGAGCAACG

dme-miR-87 UUGAGCAAAA 8nt

**miR-357: cel-miR-357 ^(5’)^, dme-miR-277**

1 10

cel-miR-357 UAAAUGCCAG

dme-miR-277 UAAAUGCACU 7nt

**miR-358: cel-miR-358 ^(5’)^, dme-miR-9c**

1

cel-miR-358 --AUUGGUAUCC

dme-miR-9c UCUUUGGUAU-- 7nt

**miR-359: cel-miR-359 ^(5’)^, dme-miR-3, dme-miR-318**

1 10

cel-miR-359 UCACUGGUCU

dme-miR-318 UCACUGGGCU 9nt

dme-miR-3 UCACUGGGCA 8nt

**miR-785: cel-miR-785 ^(5’)^, <dme-miR-987**

1

cel-miR-785 -UAAGUGAAUU

dme-miR-987 UAAAGUAAAU- 7nt

**miR-787: cel-miR-787 ^(5’)^, dme-miR-993**

1 10

cel-miR-787 UAAGCUCGUU

dme-miR-993 GAAGCUCGUC 8nt

**miR-790: cel-miR-790 ^(5’)^, dme-miR-263b**

1 10

cel-miR-790 CUUGGCACUC

dme-miR-263b CUUGGCACUG 9nt

**miR-791: cel-miR-791 ^(5’)^, dme-miR-263b**

1 10

cel-miR-791 UUUGGCACUC

dme-miR-263b CUUGGCACUG 8nt

**miR-793: cel-miR-793 ^(5’)^, dme-let-7, <dme-miR-977,**

**dme-miR-984**

1 10

cel-miR-793 UGAGGUAUCU

dme-let-7 UGAGGUAGUA 7nt

dme-miR-977 UGAGAUAUUC 7nt

dme-miR-984 UGAGGUAAAU 7nt

**miR-794: cel-miR-794 ^(5’)^, dme-let-7, <dme-miR-963, <dme-miR-977, dme-miR-984**

1

cel-miR-794 -UGAGGUAAUC

dme-let-7 -UGAGGUAGUA 8nt

dme-miR-963 ACAAGGUAAA- 6nt

dme-miR-977 -UGAGAUAUUC 8nt

dme-miR-984 -UGAGGUAAAU 8nt

**miR-795: cel-miR-795 ^(5’)^, dme-let-7, <dme-miR-963, <dme-miR-977, dme-miR-984**

1

cel-miR-795 -UGAGGUAGAU

dme-let-7 -UGAGGUAGUA 8nt

dme-miR-963 ACAAGGUAAA- 6nt

dme-miR-977 -UGAGAUAUUC 6nt

dme-miR-984 -UGAGGUAAAU 9nt

**miR-796: cel-miR-796 ^(5’)^, dme-miR-1**

1 10

cel-miR-796 UGGAAUGUAG

dme-miR-1 UGGAAUGUAA 9nt

**miR-797: cel-miR-797 ^(5’)^, dme-miR-2a, dme-miR-2b,**

**dme-miR-2c, dme-miR-6, dme-miR-11,**

**dme-miR-13a, dme-miR-13b, dme-miR-308**

1 10

cel-miR-797 UAUCACAGCA

dme-miR-13a UAUCACAGCC 9nt

dme-miR-13b UAUCACAGCC 9nt

dme-miR-2a UAUCACAGCC 9nt

dme-miR-2b UAUCACAGCC 9nt

dme-miR-2c UAUCACAGCC 9nt

dme-miR-6 UAUCACAGUG 8nt

dme-miR-308 AAUCACAGGA 8nt

dme-miR-11 CAUCACAGUC 7nt

**miR-1018: cel-miR-1018 ^(5’)^, dme-bantam**

1

cel-miR-1018 AGAGAGAUCA--

dme-bantam --UGAGAUCAUU 7nt

**miR-1022: cel-miR-1022 ^(5’)^, dme-bantam, dme-miR-306***

1

cel-miR-1022 -AAGAUCAUUG

dme-bantam UGAGAUCAUU- 8nt

dme-miR-306* GGGGGUCACU- 5nt
