# Supplementary material for: Sequence Relationships among C. elegans, D. melanogaster and Human microRNAs Highlight the Extensive Conservation of microRNAs in Biology
Source: PLoS One. 2008 Jul 30;3(7):e2818. doi: 10.1371/journal.pone.0002818 (PMC2486268; doi:10.1371/journal.pone.0002818)
Supplement: Dataset S8 — Identity table and alignments of C. elegans miRNAs with ≥70% full sequence homology to Drosophila miRNAs. (0.09 MB DOC) [file pone.0002818.s012.doc]

**Supplementary Table S8: ≥70% homology searches of worm and fly mature miRNA sequences reveals 43 sequence relationships between 31 *C. elegans* miRNAs and 37 *D. melanogaster* miRNAs.**

|  | **Sequence Related miRNAs** | |  |
| --- | --- | --- | --- |
| **miRNA Group ID** | ***C. elegans*** | ***D. melanogaster*** | **Overall Identity (≥70%)** |
| let-7 | cel-let-7 | dme-let-7 | 95.5 |
|  |  | dme-miR-984 | 75.0 |
| lin-4 | cel-lin-4 | dme-miR-125 | 81.8 |
| miR-1 | cel-miR-1 | dme-miR-1 | 90.9 |
| miR-2 | cel-miR-2 | dme-miR-2a | 95.7 |
|  |  | dme-miR-2c | 95.7 |
|  |  | dme-miR-2b | 91.3 |
|  |  | dme-miR-13a | 82.6 |
|  |  | dme-miR-13b | 78.3 |
| miR-34 | cel-miR-34 | dme-miR-34 | 87.5 |
| miR-46 | cel-miR-46 | dme-miR-281 | 78.3 |
| miR-47 | cel-miR-47 | dme-miR-281 | 73.9 |
| miR-50 | cel-miR-50 | dme-miR-190 | 75.0 |
| miR-67 | cel-miR-67 | dme-miR-307 | 70.8 |
| miR-72 | cel-miR-72 | dme-miR-31a | 91.3 |
|  |  | dme-miR-31b | 82.6 |
| miR-73 | cel-miR-73 | dme-miR-31a | 73.9 |
| miR-76 | cel-miR-76 | dme-miR-981 | 77.3 |
| miR-79 | cel-miR-79 | dme-miR-79 | 82.6 |
| miR-80 | cel-miR-80 | dme-bantam | 80.0 |
| miR-81 | cel-miR-81 | dme-bantam | 78.3 |
| miR-82 | cel-miR-82 | dme-bantam | 73.9 |
| miR-83 | cel-miR-83 | dme-miR-285 | 72.7 |
|  |  | dme-miR-998 | 72.7 |
| miR-84 | cel-miR-84 | dme-let-7 | 77.3 |
| miR-87 | cel-miR-87 | dme-miR-87 | 86.4 |
| miR-124 | cel-miR-124 | dme-miR-124 | 91.3 |
| miR-228 | cel-miR-228 | dme-miR-263a | 73.1 |
| miR-234 | cel-miR-234 | dme-miR-137 | 73.9 |
| miR-235 | cel-miR-235 | dme-miR-311 | 86.4 |
|  |  | dme-miR-310 | 81.8 |
|  |  | dme-miR-92b | 77.3 |
|  |  | dme-miR-312 | 77.3 |
|  |  | dme-miR-313 | 77.3 |
|  |  | dme-miR-92a | 72.7 |
| miR-236 | cel-miR-236 | dme-miR-8 | 82.6 |
| miR-239a | cel-miR-239a | dme-miR-12 | 70.8 |
| miR-245 | cel-miR-245 | dme-miR-133 | 73.9 |
| miR-247 | cel-miR-247 | dme-miR-996 | 72.7 |
| miR-250 | cel-miR-250 | dme-miR-1007 | 72.7 |
| miR-252 | cel-miR-252 | dme-miR-252 | 78.3 |
| miR-256 | cel-miR-256 | dme-miR-1 | 72.7 |
| miR-794 | cel-miR-794 | dme-miR-977 | 72.7 |

**Supplementary Alignments S8:**

**Sequence alignments of *C. elegans-D. melanogaster* miRNAs with ≥70% overall sequence identity.** Members of a family are ≥70% identical to at least one other miRNA member. Identity to *C. elegans* miRNAs is given in percentage at the end of each *D. melanogaster* homolog sequence. Shaded in grey are nucleotides that can potentially form G..U pairing.

**let-7: cel-let-7, dme-let-7, dme-miR-984**

1 24

cel-let-7 UGAGGUAG-UA-GGUUGUAUAGUU

dme-let-7 UGAGGUAG-UA-GGUUGUAUAGU- 95.5%

dme-miR-984 UGAGGUAAAUACGGUUGGAAUUU- 75.0%

**lin-4: cel-lin-4, dme-miR-125**

1 22

cel-lin-4 UCCCUGAGACCUCAAGU-GUGA

dme-miR-125 UCCCUGAGACCCUAACUUGUGA 81.8%

**miR-1: cel-miR-1, dme-miR-1**

1 22

cel-miR-1 UGGAAUGUAAAGAAGUAUGUA-

dme-miR-1 UGGAAUGUAAAGAAGUAUGGAG 90.9%

**miR-2: cel-miR-2, dme-miR-2a, dme-miR-2b, dme-miR-2c,**

**dme-miR-13a, dme-miR-13b**

1 23

cel-miR-2 UAUCACAGCCAGCUUUGAUGUGC

dme-miR-2c UAUCACAGCCAGCUUUGAUGGGC 95.7%

dme-miR-2a UAUCACAGCCAGCUUUGAUGAGC 95.7%

dme-miR-2b UAUCACAGCCAGCUUUGAGGAGC 91.3%

dme-miR-13b UAUCACAGCCAU-UUUGACGAGU 78.3%

dme-miR-13a UAUCACAGCCAU-UUUGAUGAGU 82.6%

**miR-34: cel-miR-34, dme-miR-34**

1 24

cel-miR-34 AGGCAGUGUGGUUAGCUGGUUG--

dme-miR-34 UGGCAGUGUGGUUAGCUGGUUGUG 87.5%

**miR-46: cel-miR-46, dme-miR-281**

1 23

cel-miR-46 UGUCAUGGAGUCGCUCUCUUCA-

dme-miR-281 UGUCAUGGAAUUGCUCUCUUUGU 78.3%

**miR-47: cel-miR-47, dme-miR-281**

1 23

cel-miR-47 UGUCAUGGAGGCGCUCUCUUCA-

dme-miR-281 UGUCAUGGAAUUGCUCUCUUUGU 73.9%

**miR-50: cel-miR-50, dme-miR-190**

1 24

cel-miR-50 UGAUAUGUCUGGUAUUCUUGGG--

dme-miR-190 AGAUAUGUUUGAUAUUCUUGGUUG 75.0%

**miR-67: cel-miR-67, dme-miR-307**

1 24

cel-miR-67 UCACAACCUCCUAGAAAGAGUAGA

dme-miR-307 UCACAACCUCCUUGAGUGAG---- 70.8%

**miR-72: cel-miR-72, dme-miR-31a, dme-miR-31b**

1 23

cel-miR-72 AGGCAAGAUGUUGGCAUAGCUGA

dme-miR-31a UGGCAAGAUGUCGGCAUAGCUGA 91.3%

dme-miR-31b UGGCAAGAUGUCGGAAUAGCUG- 82.6%

**miR-73: cel-miR-73, dme-miR-31a**

1 23

cel-miR-73 UGGCAAGAUGUAGGCAGUUCAGU

dme-miR-31a UGGCAAGAUGUCGGCAUAGCUGA 73.9%

**miR-76: cel-miR-76, dme-miR-981**

1 22

cel-miR-76 UUCGUUGUUGAUGAAGCCUUGA

dme-miR-981 UUCGUUGUCGACGAAACCUGCA 77.3%

**miR-79: cel-miR-79, dme-miR-79**

1 23

cel-miR-79 AUAAAGCUAGGUUACCAAAGCU-

dme-miR-79 -UAAAGCUAGAUUACCAAAGCAU 82.6%

**miR-80: cel-miR-80, dme-bantam**

1 25

cel-miR-80 UGAGAUCAUUAGUUGAAAGCCGA--

dme-bantam UGAGAUCAUU--UUGAAAGCUGAUU 80.0%

**miR-81: cel-miR-81, dme-bantam**

1 23

cel-miR-81 UGAGAUCAUCGUGAAAGCUAGU-

dme-bantam UGAGAUCAUUUUGAAAGCUGAUU 78.3%

**miR-82: cel-miR-82, dme-bantam**

1 23

cel-miR-82 UGAGAUCAUCGUGAAAGCCAGU-

dme-bantam UGAGAUCAUUUUGAAAGCUGAUU 73.9%

**miR-83: cel-miR-83, dme-miR-285, dme-miR-998**

1 22

cel-miR-83 UAGCACCAUAUAAAUUCAGUAA

dme-miR-285 UAGCACCAUUCGAAAUCAGUGC 72.7%

dme-miR-998 UAGCACCAUG-AGAUUCAGCUC 72.7%

**miR-84: cel-miR-84, dme-let-7**

1 22

cel-miR-84 UGAGGUAGUAUGUAAUAUUGUA

dme-let-7 UGAGGUAGUAGGUUGUAUAGU- 77.3%

**miR-87: cel-miR-87, dme-miR-87**

1 22

cel-miR-87 GUGAGCAAAGUUUCAGGUGUGC

dme-miR-87 UUGAGCAAAAUUUCAGGUGUG- 86.4%

**miR-124: cel-miR-124, dme-miR-124**

1 23

cel-miR-124 UAAGGCACGCGGUGAAUGCCA--

dme-miR-124 UAAGGCACGCGGUGAAUGCCAAG 91.3%

**miR-228: cel-miR-228, dme-miR-263a**

1 26

cel-miR-228 ---AAUGGCACUGCAUGAAUUCACGG

dme-miR-263a GUUAAUGGCACUGGAAGAAUUCAC-- 73.1%

**miR-234: cel-miR-234, dme-miR-137**

1 23

cel-miR-234 UUAUUGCUCGAGAAUACCCUU--

dme-miR-137 -UAUUGCUUGAGAAUACACGUAG 73.9%

**miR-235: cel-miR-235, dme-miR-92a, dme-miR-92b,**

**dme-miR-310, dme-miR-311, dme-miR-312,**

**dme-miR-313**

1 22

cel-miR-235 UAUUGCACUCUCCCCGGCCUGA

dme-miR-311 UAUUGCACAUUCACCGGCCUGA 86.4%

dme-miR-310 UAUUGCACACUUCCCGGCCUUU 81.8%

dme-miR-92a CAUUGCACUUGUCCCGGCCUAU 72.7%

dme-miR-92b AAUUGCACUAGUCCCGGCCUGC 77.3%

dme-miR-312 UAUUGCACUUGAGACGGCCUGA 77.3%

dme-miR-313 UAUUGCACUUUUCACAGCCCGA 77.3%

**miR-236: cel-miR-236, dme-miR-8**

1 23

cel-miR-236 UAAUACUGUCAGGUAAUGACGCU

dme-miR-8 UAAUACUGUCAGGUAAAGAUGUC 82.6%

**miR-239a: cel-miR-239a, dme-miR-12**

1 24

cel-miR-239a UUUGUACUACACAUAGGUACUGG-

dme-miR-12 UGAGUAUUACAUC-AGGUACUGGU 70.8%

**miR-245: cel-miR-245, dme-miR-133**

1 23

cel-miR-245 AUUGGUCCCCUCCAAGUAGCUC-

dme-miR-133 -UUGGUCCCCUUCAACCAGCUGU 73.9%

**miR-247: cel-miR-247, dme-miR-996**

1 22

cel-miR-247 UGACUAGAGCCUAUUCUCUUCU

dme-miR-996 UGACUAGAUUUCAUGCUCGUCU 72.7%

**miR-250: cel-miR-250, dme-miR-1007**

1 22

cel-miR-250 -AAUCACAGUCAACUGUUGGCA

dme-miR-1007 UAAGCUCAAUUAACUGUUUGCA 72.7%

**miR-252: cel-miR-252, dme-miR-252**

1 23

cel-miR-252 AUAAGUAGUAGUGCCGCAGGUAA

dme-miR-252 CUAAGUACUAGUGCCGCAGGAG- 78.3%

**miR-256: cel-miR-256, dme-miR-1**

1 22

cel-miR-256 UGGAAUGCAUAGAAGACUGUA-

dme-miR-1 UGGAAUGUAAAGAAGUAUGGAG 72.7%

**miR-794: cel-miR-794, dme-miR-977**

1 22

cel-miR-794 UGAGGUAAUCAUCGUUGUCACU

dme-miR-977 UGAGAUAUUCA-CGUUGUCUAA 72.7%
