# Supplementary material for: Sequence Relationships among C. elegans, D. melanogaster and Human microRNAs Highlight the Extensive Conservation of microRNAs in Biology
Source: PLoS One. 2008 Jul 30;3(7):e2818. doi: 10.1371/journal.pone.0002818 (PMC2486268; doi:10.1371/journal.pone.0002818)
Supplement: Dataset S9 — Table and alignments of C. elegans and Drosophila miRNAs with 60–69.9% similarity over whole sequence. (0.21 MB DOC) [file pone.0002818.s013.doc]

**Supplementary Table S9: 60-69.9% overall sequence similarity search detects 91 sequence relationships between 60 *C. elegans* miRNAs and 62 *D. melanogaster* miRNAs.** 53 of these *C. elegans* miRNAs are not related in sequence to other fly miRNAs over the ≥70% threshold (Dataset S8). Significantly, 51 of the 91 sequence relationships share 7-10 continuous nt of similarity at the 5’ end (Dataset S7, sequence alignments below).

|  | Distantly Related miRNAs | |  |
| --- | --- | --- | --- |
| **miRNA**  **Group ID** | ***C. elegans*** | ***D. melanogaster*** | Overall Identity **(60-69.9%)** |
| **let-7** | cel-let-7 | dme-miR-977 | 65.2 |
|  |  | dme-miR-991 | 65.2 |
| **lin-4** | cel-lin-4 | dme-miR-10 | 60.9 |
| **miR-2** | cel-miR-2 | dme-miR-6 | 60.9 |
| **miR-43** | cel-miR-43 | dme-miR-2b | 60.9 |
| **miR-44** | cel-miR-44 | dme-miR-286 | 69.6 |
|  |  | dme-miR-279 | 68.2 |
|  |  | dme-miR-996 | 63.6 |
| **miR-45** | cel-miR-45 | dme-miR-286 | 69.6 |
|  |  | dme-miR-279 | 68.2 |
|  |  | dme-miR-996 | 63.6 |
| **miR-49** | cel-miR-49 | dme-miR-998 | 63.6 |
| **miR-50** | cel-miR-50 | dme-miR-958 | 60.9 |
| **miR-51** | cel-miR-51 | dme-miR-10 | 62.5 |
|  |  | dme-miR-100 | 65.2 |
| **miR-52** | cel-miR-52 | dme-miR-955 | 62.5 |
| **miR-57** | cel-miR-57 | dme-miR-100 | 68.0 |
|  |  | dme-miR-10 | 66.7 |
| **miR-58** | cel-miR-58 | dme-bantam | 60.9 |
|  |  | dme-miR-1000 | 60.9 |
| **miR-59** | cel-miR-59 | dme-miR-1 | 60.9 |
|  |  | dme-miR-34 | 60.0 |
| **miR-61** | cel-miR-61 | dme-miR-279 | 69.6 |
|  |  | dme-miR-996 | 63.6 |
|  |  | dme-miR-286 | 60.9 |
| **miR-70** | cel-miR-70 | dme-miR-1008 | 60.9 |
| **miR-73** | cel-miR-73 | dme-miR-31b | 69.6 |
| **miR-74** | cel-miR-74 | dme-miR-31a | 60.9 |
| **miR-75** | cel-miR-75 | dme-miR-4 | 69.6 |
| **miR-79** | cel-miR-79 | dme-miR-4 | 68.2 |
| **miR-81** | cel-miR-81 | dme-miR-960 | 60.9 |
| **miR-83** | cel-miR-83 | dme-miR-995 | 68.2 |
| **miR-85** | cel-miR-85 | dme-miR-985 | 60.0 |
| **miR-86** | cel-miR-86 | dme-miR-965 | 62.5 |
|  |  | dme-miR-1012 | 60.9 |
| **miR-228** | cel-miR-228 | dme-miR-263b | 69.6 |
| **miR-231** | cel-miR-231 | dme-miR-993 | 66.7 |
| **miR-232** | cel-miR-232 | dme-miR-277 | 60.0 |
| **miR-233** | cel-miR-233 | dme-miR-87 | 63.6 |
| **miR-237** | cel-miR-237 | dme-miR-125 | 60.0 |
| **miR-239a** | cel-miR-239a | dme-miR-305 | 65.2 |
| **miR-239b** | cel-miR-239b | dme-miR-305 | 65.2 |
|  |  | dme-miR-12 | 62.5 |
| **miR-240** | cel-miR-240 | dme-miR-193 | 60.9 |
| **miR-241** | cel-miR-241 | dme-miR-984 | 60.9 |
| **miR-244** | cel-miR-244 | dme-miR-9a | 68.0 |
|  |  | dme-miR-9b | 66.7 |
| **miR-246** | cel-miR-246 | dme-miR-992 | 63.6 |
| **miR-247** | cel-miR-247 | dme-miR-286 | 69.6 |
|  |  | dme-miR-279 | 65.2 |
| **miR-249** | cel-miR-249 | dme-miR-1008 | 66.7 |
| **miR-250** | cel-miR-250 | dme-miR-2c | 65.2 |
|  |  | dme-miR-11 | 63.6 |
|  |  | dme-miR-308 | 62.5 |
|  |  | dme-miR-990 | 61.5 |
|  |  | dme-miR-2b | 60.9 |
| **miR-252** | cel-miR-252 | dme-miR-1013 | 60.9 |
| **miR-253*** | cel-miR-253* | dme-miR-1004 | 60.9 |
| **miR-254** | cel-miR-254 | dme-miR-1016 | 60.9 |
| **miR-259** | cel-miR-259 | dme-miR-975 | 60.0 |
| **miR-261** | cel-miR-261 | dme-miR-1008 | 63.6 |
| **miR-262** | cel-miR-262 | dme-miR-iab-4-5p | 60.9 |
| **miR-264** | cel-miR-264 | dme-miR-34 | 69.6 |
| **miR-265** | cel-miR-265 | dme-let-7 | 65.2 |
|  |  | dme-miR-974 | 60.9 |
|  |  | dme-miR-984 | 60.9 |
| **miR-266** | cel-miR-266 | dme-miR-31a | 65.2 |
|  |  | dme-miR-31b | 63.6 |
| **miR-269** | cel-miR-269 | dme-miR-31a | 60.9 |
| **miR-270** | cel-miR-270 | dme-miR-31a | 60.9 |
| **miR-272** | cel-miR-272 | dme-miR-966 | 66.7 |
| **miR-359** | cel-miR-359 | dme-miR-318 | 68.2 |
|  |  | dme-miR-14 | 63.6 |
|  |  | dme-miR-9c | 60.9 |
| **miR-784** | cel-miR-784 | dme-miR-277 | 64.0 |
| **miR-787** | cel-miR-787 | dme-miR-1006 | 60.9 |
| **miR-792** | cel-miR-792 | dme-miR-1016 | 60.9 |
| **miR-793** | cel-miR-793 | dme-let-7 | 63.6 |
| **miR-794** | cel-miR-794 | dme-let-7 | 65.2 |
|  |  | dme-miR-984 | 65.2 |
|  |  | dme-miR-287 | 63.6 |
|  |  | dme-miR-963 | 60.0 |
| **miR-796** | cel-miR-796 | dme-miR-1 | 65.2 |
| **miR-797** | cel-miR-797 | dme-miR-2b | 68.0 |
|  |  | dme-miR-13a | 64.0 |
|  |  | dme-miR-2a | 61.5 |
|  |  | dme-miR-13b | 60.0 |
| **miR-798** | cel-miR-798 | dme-miR-1003 | 60.9 |
| **miR-1019** | cel-miR-1019 | dme-miR-932 | 62.5 |
| **miR-1020** | cel-miR-1020 | dme-miR-1014 | 61.9 |
|  |  | dme-miR-1009 | 60.0 |

**Supplementary Alignments S9:**

**Alignments of sequence-related *C. elegans* and *D. melanogaster* miRNAs with overall 60-69.9% similarity.** Identity to *C. elegans* miRNAs is given in percentage at the end of each *D. melanogaster* homolog sequence. Grey shading denotes potential G..U pairing.

**let-7: cel-let-7, dme-miR-977, dme-miR-991**

1 23

cel-let-7 UGAGGUAGU-AGGUUGUAUAGUU

dme-miR-977 UGAGAUAUUCACGUUGUCUAA-- 65.2%

1 23

cel-let-7 -UGAGGUAGUAGGUUGUAUAGUU

dme-miR-991 UUAAAGUUGUAGUUUGGAAAGU- 65.2%

**lin-4: cel-lin-4, dme-miR-10**

1 23

cel-lin-4 UCCCUG-AGA-CCUCAAGUGUGA

dme-miR-10 ACCCUGUAGAUCCGAAUUUGU-- 60.9%

**miR-2: cel-miR-2, dme-miR-6**

1 23

cel-miR-2 UAUCACAGCCAGCUUUGAUGUGC

dme-miR-6 UAUCACAGUG-GCUGUUCUUUUU 60.9%

**miR-43: cel-miR-43, dme-miR-2b**

1 23

cel-miR-43 UAUCACAGUUUACUUGCUGUCGC

dme-miR-2b UAUCACAGCCAGCUUUGAGGAGC 60.9%

**miR-44: cel-miR-44, dme-miR-279, dme-miR-286,**

**dme-miR-996**

1 23

cel-miR-44 UGACUAGAGACA-CAUUCA-GCU

dme-miR-286 UGACUAGACCGAACACUCGUGCU 69.6%

1 22

cel-miR-44 UGACUAGAGACACAUUCAGCU-

dme-miR-279 UGACUAGAUCCACACUCAUUAA 68.2%

1 22

cel-miR-44 UGACUAGAGA-CACAUUCAGCU

dme-miR-996 UGACUAGAUUUCAUGCUCGUCU 63.6%

**miR-45: cel-miR-45, dme-miR-279, dme-miR-286,**

**dme-miR-996**

1 23

cel-miR-45 UGACUAGAGACA-CAUUCA-GCU

dme-miR-286 UGACUAGACCGAACACUCGUGCU 69.6%

1 22

cel-miR-45 UGACUAGAGACACAUUCAGCU-

dme-miR-279 UGACUAGAUCCACACUCAUUAA 68.2%

1 22

cel-miR-45 UGACUAGAGA-CACAUUCAGCU

dme-miR-996 UGACUAGAUUUCAUGCUCGUCU 63.6%

**miR-49: cel-miR-49, dme-miR-998**

1 22

cel-miR-49 AAGCACCACGAGAAGCUGCAGA

dme-miR-998 UAGCACCAUGAGAUUCAGCUC- 63.6%

**miR-50: cel-miR-50, dme-miR-958**

1 23

cel-miR-50 UGAUAUGUCUGGUAUUCUUGGG-

dme-miR-958 UGAGAU-UCUUCUAUUCUACUUU 60.9%

**miR-51: cel-miR-51, dme-miR-10, dme-miR-100**

1 23

cel-miR-51 UACCCGUAGCUCCUAUCCAUGUU

dme-miR-100 AACCCGUAAAUCCGAACU-UGUG 65.2%

1 24

cel-miR-51 UACCC-GUAGCUCCUAUCCAUGUU

dme-miR-10 -ACCCUGUAGAUCCGAAUU-UGU- 62.5%

**miR-52: cel-miR-52, dme-miR-955**

1 24

cel-miR-52 CACCCGUACAUAUGUUUCCGUGCU

dme-miR-955 CAUC-GUGCAGAGGUUUGAGUGUC 62.5%

**miR-57: cel-miR-57, dme-miR-10, dme-miR-100**

1 25

cel-miR-57 UACCCUGUAGAUC-GAGCUGUGUGU

dme-miR-100 -AACCCGUAAAUCCGAACU-UGUG- 68.0%

1 24

cel-miR-57 UACCCUGUAGAUCGAGCUGUGUGU

dme-miR-10 -ACCCUGUAGAUCCGAAUUUGU-- 66.7%

**miR-58: cel-miR-58, dme-bantam, dme-miR-1000**

1 23

cel-miR-58 UGAGAUCGUUCAGUACGGCAAU-

dme-bantam UGAGAUCAUUUUGAAAGCUGAUU 60.9%

1 23

cel-miR-58 UGAGAUCGUUCAGU-ACGGCAAU

dme-miR-1000 --AUAUUGUCCUGUCACAGCAGU 60.9%

**miR-59: cel-miR-59, dme-miR-1, dme-miR-34**

1 23

cel-miR-59 UCGAAUCGUUUAUCAGGAUGAUG

dme-miR-1 UGGAAU-GUAAAGAAGUAUGGAG 60.9%

1 25

cel-miR-59 UCGAAUCGU--UUAUCAGGAUGAUG

dme-miR-34 UGGCAGUGUGGUUAGCUGGUUG-UG 60.0%

**miR-61: cel-miR-61, dme-miR-279, dme-miR-286,**

**dme-miR-996**

1 23

cel-miR-61 UGACUAGAACCGUUACUCAUC--

dme-miR-279 UGACUAGAUCCAC-ACUCAUUAA 69.6%

1 22

cel-miR-61 UGACUAGAACCGUUACUCAUC-

dme-miR-996 UGACUAGAUUUCAUGCUCGUCU 63.6%

1 23

cel-miR-61 UGACUAGAACCGUUACUCAUC--

dme-miR-286 UGACUAGACCGAACACUCGUGCU 60.9%

**miR-70: cel-miR-70, dme-miR-1008**

1 23

cel-miR-70 UAAUACGUCGUUGGUGUUUCCAU

dme-miR-1008 UCACAGCUU-UUUGUGUUUACA- 60.9%

**miR-73: cel-miR-73, dme-miR-31b**

1 23

cel-miR-73 UGGCAAGAUGUAGGCAGUUCAGU

dme-miR-31b UGGCAAGAUGUCGGAAUAGCUG- 69.6%

**miR-74: cel-miR-74, dme-miR-31a**

1 23

cel-miR-74 UGGCAAGAAAU-GGCAGUCUACA

dme-miR-31a UGGCAAGAUGUCGGCAUAGCUGA 60.9%

**miR-75: cel-miR-75, dme-miR-4**

1 23

cel-miR-75 UUAAAGCUAC-CAACCGGCUUCA

dme-miR-4 AUAAAGCUAGACAACCA--UUGA 69.6%

**miR-79: cel-miR-79, dme-miR-4**

1 22

cel-miR-79 AUAAAGCUAGGUUACCAAAGCU

dme-miR-4 AUAAAGCUAGACAACCAUUGA- 68.2%

**miR-81: cel-miR-81, dme-miR-960**

1 23

cel-miR-81 UGAGAUCAUCGUGAAAGC-UAGU

dme-miR-960 UGAG-UAUUCCAGAUUGCAUAGC 60.9%

**miR-83: cel-miR-83, dme-miR-995**

1 22

cel-miR-83 UAGCACCAUAUAAAUUCAGUAA

dme-miR-995 UAGCACCACAUGA-UUCGGCUU 68.2%

**miR-85: cel-miR-85, dme-miR-985**

1 25

cel-miR-85 UACAAAGUAUUUGAAAAGUCGUGC-

dme-miR-985 --CAAA-UGUUCCAAUGGUCGGGCA 60.0%

**miR-86: cel-miR-86, dme-miR-965, dme-miR-1012**

1 23

cel-miR-86 UAAGUGAAUGCUUUGCCACAGUC

dme-miR-1012 UUAGUCAAAGAUUUUCCCCAUAG 60.9%

1 24

cel-miR-86 UAAGUGAAU-GCUUUGCCACAGUC

dme-miR-965 UAAGCGUAUAGCUUUUCCCCUU-- 62.5%

**miR-228: cel-miR-228, dme-miR-263b**

1 23

cel-miR-228 AAUGGCACUGCAUGAAUUCACGG

dme-miR-263b CUUGGCACUGGGAGAAUUCAC-- 69.6%

**miR-231: cel-miR-231, dme-miR-993**

1 24

cel-miR-231 UAAGCUCGUGAUCAACAGGCAGAA

dme-miR-993 GAAGCUCGUC-UCUACAGGUAUCU 66.7%

**miR-232: cel-miR-232, dme-miR-277**

1 25

cel-miR-232 UAAAUGCAUCUUAACUGCGGUGA--

dme-miR-277 UAAAUGCAC--UAUCUGGUACGACA 60.0%

**miR-233: cel-miR-233, dme-miR-87**

1 22

cel-miR-233 UUGAGCAAUGCG-CAUGUGCGG

dme-miR-87 UUGAGCAAAAUUUCAGGUGUG- 63.6%

**miR-237: cel-miR-237, dme-miR-125**

1 25

cel-miR-237 UCCCUGAGAAUUCUCGAACAGCU--

dme-miR-125 UCCCUGAGA---CCCUAACUUGUGA 60.0%

**miR-239a: cel-miR-239a, dme-miR-305**

1 24

cel-miR-239a UUUGUACUACA-CAUAGGUACUGG

dme-miR-305 AUUGUACUUCAUCAGGUGCUCUG- 65.2%

**miR-239b: cel-miR-239b, dme-miR-12, dme-miR-305**

1 23

cel-miR-239b UUUGUACUACA-CAAAAGUACUG

dme-miR-305 AUUGUACUUCAUCAGGUGCUCUG 65.2%

1 24

cel-miR-239b UUUGUACUACACAAAAGUACUG--

dme-miR-12 UGAGUAUUACAUCAG-GUACUGGU 62.5%

**miR-240: cel-miR-240, dme-miR-193**

1 23

cel-miR-240 UACUGGCCCCCAAA-UCUUCGCU

dme-miR-193 UACUGGCCUACUAAGUCCCAAC- 60.9%

**miR-241: cel-miR-241, dme-miR-984**

1 23

cel-miR-241 UGAGGUAGGUGCGA--GAAAUGA

dme-miR-984 UGAGGUAAAUACGGUUGGAAUUU 60.9%

**miR-244: cel-miR-244, dme-miR-9a, dme-miR-9b**

1 25

cel-miR-244 UCUUUGGUUGUACAAAGUGGUAUG-

dme-miR-9a UCUUUGGUUAUCU--AGCUGUAUGA 68.0%

1 24

cel-miR-244 UCUUUGGUUGUACAAAGUGGUAUG

dme-miR-9b UCUUUGGUGAUUUUAGCUG-UAUG 66.7%

**miR-246: cel-miR-246, dme-miR-992**

1 22

cel-miR-246 -UUACAUGUUUCGGGUAGGAGC

dme-miR-992 AGUACACGUUUCUGGUACUAAG 63.6%

**miR-247: cel-miR-247, dme-miR-279, dme-miR-286**

1 23

cel-miR-247 UGACUAGAGCCUAUUCUCUU-CU

dme-miR-286 UGACUAGACCGAACACUCGUGCU 69.6%

1 23

cel-miR-247 UGACUAGAGCCUAUUCUCUUCU-

dme-miR-279 UGACUAGAUCC-ACACUCAUUAA 65.2%

**miR-249: cel-miR-249, dme-miR-1008**

1 24

cel-miR-249 UCACAGGACUUUUGAGCGUUGCC-

dme-miR-1008 UCACAG--CUUUUU-GUGUUUACA 66.7%

**miR-250: cel-miR-250, dme-miR-2b, dme-miR-2c, dme-miR-11,**

**dme-miR-308, dme-miR-990**

1 23

cel-miR-250 AAUCACAGUCAACUGU--UGGCA

dme-miR-2c UAUCACAGCCAGCUUUGAUGGGC 65.2%

1 22

cel-miR-250 AAUCACAGUCA-ACUGUUGGCA

dme-miR-11 CAUCACAGUCUGAGUUCUUGC- 63.6%

1 24

cel-miR-250 AAUCACAG---UCAACUGUUGGCA

dme-miR-308 AAUCACAGGAUUAUACUGUGAG-- 62.5%

1 21

cel-miR-250 AAUCACAGUCAACUGUUGGCA

dme-miR-990 AUUCACCGUUCUGAGUUGGCC 61.9%

1 23

cel-miR-250 AAUCACAGUCAACUGUUGGCA--

dme-miR-2b UAUCACAGCCAGCUUUGAGGAGC 60.9%

**miR-252: cel-miR-252, dme-miR-1013**

1 23

cel-miR-252 AUAAGUAGUAGUGCCGCAGGUAA

dme-miR-1013 AUAAA-AGUA-UGCCGAACUCG- 60.9%

**miR-253*: cel-miR-253*, dme-miR-1004**

1 23

cel-miR-253* -CACACCUCACUAACACUGACC-

dme-miR-1004 UCUCACAUCACUU-CCCUCACAG 60.9%

**miR-254: cel-miR-254, dme-miR-1016**

1 23

cel-miR-254 UGCAAAUCUUUCGCGACUGUAGG

dme-miR-1016 UUCACCUCUCUCCAUACU-UAG- 60.9%

**miR-259: cel-miR-259, dme-miR-975**

1 25

cel-miR-259 AAAUCUCAUCCUA-AUCUGGUAGCA

dme-miR-975 UAAACACUUCCUACAUCCUGUAU-- 60.0%

**miR-261: cel-miR-261, dme-miR-1008**

1 22

cel-miR-261 ---UAGCUUUUUAGUUUUCACG

dme-miR-1008 UCACAGCUUUUU-GUGUUUACA 63.6%

**miR-262: cel-miR-262, dme-miR-iab-4-5p**

1 23

cel-miR-262 --GUUU-CUCGAUGUUUUCUGAU

dme-miR-iab-4-5p ACGUAUACUGAAUGUAUCCUGA- 60.9%

**miR-264: cel-miR-264, dme-miR-34**

1 23

cel-miR-264 -GGC-GGGUGGUU-GUUGUUAUG

dme-miR-34 UGGCAGUGUGGUUAGCUGGU-UG 69.6%

**miR-265: cel-miR-265, dme-let-7, dme-miR-974, dme-miR-984**

1 23

cel-miR-265 UGAGGGAGGAAGGGUGGUAU---

dme-let-7 UGAGGUAGUA--GGUUGUAUAGU 65.2%

1 23

cel-miR-265 UGAGGGAGGAAGG--GUGGUAU-

dme-miR-974 -AAGCGAGCAAAGAAGUAGUAUU 60.9%

1 23

cel-miR-265 UGAGGGAGGAAGGGU-GGUAU--

dme-miR-984 UGAGGUAAAUACGGUUGGAAUUU 60.9%

**miR-266: cel-miR-266, dme-miR-31a, dme-miR-31b**

1 23

cel-miR-266 AGGCAAGACUUUGGCAAAGC---

dme-miR-31a UGGCAAGAUGUCGGCAUAGCUGA 65.2%

1 22

cel-miR-266 AGGCAAGACUUUGGCAAAGC--

dme-miR-31b UGGCAAGAUGUCGGAAUAGCUG 63.6%

**miR-269: cel-miR-269, dme-miR-31a**

1 23

cel-miR-269 -GGCAAGACUCUGGCAAAACU--

dme-miR-31a UGGCAAGAUGUCGGCAUAGCUGA 60.9%

**miR-270: cel-miR-270, dme-miR-31a**

1 23

cel-miR-270 -GGCAUGAUGUAG-CAGUGGAG-

dme-miR-31a UGGCAAGAUGUCGGCAUAGCUGA 60.9%

**miR-272: cel-miR-272, dme-miR-966**

1 21

cel-miR-272 UGUAGGCA-UGGG-UGUUUG-

dme-miR-966 UGUGGGUUGUGGGCUGUGUGG 66.7%

**miR-359: cel-miR-359, dme-miR-9c, dme-miR-14,**

**dme-miR-318**

1 22

cel-miR-359 UCACUGGUCUUUCUCUGACGAA

dme-miR-318 UCACUGGGCUUUGUUUAUCUCA 68.2%

1 22

cel-miR-359 UCACUGGUCUUUCUCUGACGAA

dme-miR-14 UCAGUCUU-UUUCUCUCUCCUA 63.6%

1 23

cel-miR-359 UCACUGGUCUU-UCUCUGACGAA

dme-miR-9c UCUUUGGUAUUCUAGCUGUAGA- 60.9%

**miR-784: cel-miR-784, dme-miR-277**

1 25

cel-miR-784 ---UGGCACAAUCUGCGUACGUAGA

dme-miR-277 UAAAUGCACUAUCUG-GUACG-ACA 64.0%

**miR-787: cel-miR-787, dme-miR-1006**

1 23

cel-miR-787 UAAGCUCGUUUUAGUAUCUUUCG

dme-miR-1006 UAAAUUCGAUUUCUUAUUCAUAG 60.9%

**miR-792: cel-miR-792, dme-miR-1016**

1 23

cel-miR-792 UUGAAAUCUCUUCAACUUUCAGA

dme-miR-1016 UUCACCUCUCUCCAUACUU-AG- 60.9%

**miR-793: cel-miR-793, dme-let-7**

1 22

cel-miR-793 UGAGGUAUCUUAGUUAGACAGA

dme-let-7 UGAGGUAG-UAGGUUGUAUAGU 63.6%

**miR-794: cel-miR-794, dme-let-7, dme-miR-287, dme-miR-963,**

**dme-miR-984**

1 23

cel-miR-794 UGAGGUAAUCAUCGUUGUC-ACU

dme-let-7 UGAGGUAGUAG--GUUGUAUAGU 65.2%

1 23

cel-miR-794 UGAGGUAAUCAUCGUUGUCACU-

dme-miR-984 UGAGGUAAAUACGGUUGGAAUUU 65.2%

1 22

cel-miR-794 UGAGGUAAUCAUCGUUGUCACU

dme-miR-287 UGUGUUGAAAAUCGUUUGCAC- 63.6%

1 25

cel-miR-794 -UGAGGUAAUCAUC--GUUGUCACU

dme-miR-963 ACAAGGUAAAUAUCAGGUUGUUUC- 60.0%

**miR-796: cel-miR-796, dme-miR-1**

1 23

cel-miR-796 UGGAAUGUAGUUGAGGUUAGUAA

dme-miR-1 UGGAAUGUAAA-GAAGUAUGGAG 65.2%

**miR-797: cel-miR-797, dme-miR-2a, dme-miR-2b,**

**dme-miR-13a, dme-miR-13b**

1 25

cel-miR-797 UAUCACAGCAAUCACAAUGAGAAGA

dme-miR-2b UAUCACAGCCAGCUU--UGAGGAGC 68.0%

1 25

cel-miR-797 UAUCACAGCAAUCACAAUGAGAAGA

dme-miR-13a UAUCACAGCCAUUUUGAUGAGU--- 64.0%

1 25

cel-miR-797 UAUCACAGCAAUCACAAUGAGAAGA

dme-miR-13b UAUCACAGCCAUUUUGACGAGU--- 60.0%

1 26

cel-miR-797 UAUCACAGCAAUCAC-AAUGAGAAGA

dme-miR-2a UAUCACAGCCAGCUUUGAUGAGC--- 61.5%

**miR-798: cel-miR-798 and dme-miR-1003**

1 23

cel-miR-798 --UAAGCCUUACAUAUUGACUGA

dme-miR-1003 UCUCACAUUUACAUAUUCACAG- 60.9%

**miR-1019: cel-miR-1019, dme-miR-932**

1 24

cel-miR-1019 CUGUAAUUCCACAUUGCUUUCCAG

dme-miR-932 --UCAAUUCCGUAGUGCAUUGCAG 62.5%

**miR-1020: cel-miR-1020, dme-miR-1009, dme-miR-1014**

1 25

cel-miR-1020 ----AUUAUUCUGUGACACUUUCAG

dme-miR-1009 UCUCAAAAAU-UGUUACA-UUUCAG 60.0%

1 21

cel-miR-1020 AUUAUUCUGUGACACUUUCAG

dme-miR-1014 AAAAUUCAUUUUCAUUUGCAG 61.9%
