# Supplementary material for: Sequence Relationships among C. elegans, D. melanogaster and Human microRNAs Highlight the Extensive Conservation of microRNAs in Biology
Source: PLoS One. 2008 Jul 30;3(7):e2818. doi: 10.1371/journal.pone.0002818 (PMC2486268; doi:10.1371/journal.pone.0002818)
Supplement: Dataset S10 — Tables and alignments of C. elegans and H. sapiens miRNAs with homologous 5′ ends. (0.47 MB DOC) [file pone.0002818.s014.doc]

**Supplementary Table S10: 76 *C. elegans* miRNAs are related at the 5’ end with 98 human miRNAs in 262 sequence relationships.** Superscript “less than” (**<**) symbol before miRNA names indicates allowed A-G base changes (G..U pairing) interrupting the 5’ 7nt homology block that groups specific miRNAs into families (see alignments below). Homologous nucleotides grouping miRNAs into families are summarized in the table and detailed in alignments. **(5’)** marks 69 miRNAs with high identity at the 5’ end but <70% overall similarity with at least one of their sequence-related miRNAs. 41 of the 69 miRNAs (cel-miR-2, cel-miR-43, cel-miR-44, cel-miR-45, cel-miR-49, cel-miR-54, cel-miR-55, cel-miR-56, cel-miR-58, cel-miR-61, cel-miR-62, cel-miR-63, cel-miR-64, cel-miR-65, cel-miR-66, cel-miR-75, cel-miR-81, cel-miR-86, cel-miR-90, cel-miR-229, cel-miR-231, cel-miR-232, cel-miR-241, cel-miR-244, cel-miR-247, cel-miR-250, cel-miR-251, cel-miR-252, cel-miR-254, cel-miR-259, cel-miR-267, cel-miR-268, cel-miR-273, cel-miR-357, cel-miR-785, cel-miR-787, cel-miR-790, cel-miR-797, cel-miR-1018, cel-miR-1020 and cel-miR-1022) have poor overall similarity (<60%) to all their 5’ related human sequences and thus their sequence relationships are not listed in Datasets S11 and S12.

|  | | **5’ End Sequence Related miRNAs** | |  |
| --- | --- | --- | --- | --- |
| **miRNA Group ID** | ***C. elegans*** | | ***H. sapiens*** | **# Identical nt at 5' End (10nt)** |
| let-7  *(G)AGGUAG(U)* | cel-let-7 **(5’)** | | hsa-let-7a | 10 |
|  |  |  | hsa-let-7b | 10 |
|  |  |  | hsa-let-7c | 10 |
|  |  |  | hsa-let-7f | 10 |
|  |  |  | hsa-let-7g | 10 |
|  |  |  | hsa-let-7i | 10 |
|  |  |  | hsa-miR-98 | 10 |
|  |  |  | hsa-let-7d | 9 |
|  |  |  | hsa-let-7e | 9 |
|  |  |  | hsa-miR-196a | 7 |
|  |  |  | hsa-miR-196b | 7 |
| lin-4 CCCUGA/GG | cel-lin-4 **(5’)** | | hsa-miR-125a-5p | 10 |
|  |  |  | hsa-miR-125b | 10 |
|  |  |  | **<**hsa-miR-331-3p | 6 |
| miR-1 UGGAA/GUG | cel-miR-1 **(5’)** | | hsa-miR-1 | 10 |
|  |  |  | hsa-miR-206 | 10 |
|  |  |  | **<**hsa-miR-122 | 8 |
| miR-2  *AUCACAG* | cel-miR-2 **(5’)** | | hsa-miR-499-3p | 7 |
| miR-34 GGCAGUG | cel-miR-34 | | hsa-miR-34b* | 9 |
|  |  |  | hsa-miR-34c-5p | 9 |
|  |  |  | hsa-miR-449b | 9 |
|  |  |  | hsa-miR-34 | 8 |
|  |  |  | hsa-miR-449a | 8 |
| miR-43  *(A)UCACAG/A(U)* | cel-miR-43 **(5’)** | | hsa-miR-27a | 7 |
|  |  |  | hsa-miR-27b | 7 |
|  |  |  | hsa-miR-128 | 7 |
|  |  |  | hsa-miR-499-3p | 7 |
|  |  |  | **<**hsa-miR-768-3p | 6 |
| miR-44 (U)G/AACUA/GG(A) | cel-miR-44 **(5’)** | | **<**hsa-miR-134 | 6 |
|  |  |  | **<**hsa-miR-708* | 6 |
| miR-45  *(U)G/AACUA/GG(A)* | cel-miR-45 **(5’)** | | **<**hsa-miR-134 | 6 |
|  |  |  | **<**hsa-miR-708* | 6 |
| miR-48 GAGGUAG | cel-miR-48 **(5’)** | | hsa-let-7e | 9 |
|  |  |  | hsa-let-7a | 8 |
|  |  |  | hsa-let-7b | 8 |
|  |  |  | hsa-let-7c | 8 |
|  |  |  | hsa-let-7f | 8 |
|  |  |  | hsa-let-7g | 8 |
|  |  |  | hsa-let-7i | 8 |
|  |  |  | hsa-miR-98 | 8 |
|  |  |  | hsa-let-7d | 7 |
| miR-49 A/GG/ACACCA | cel-miR-49 **(5’)** | | hsa-miR-29a | 7 |
|  |  |  | hsa-miR-29b | 7 |
|  |  |  | hsa-miR-29c | 7 |
|  |  |  | **<**hsa-miR-593* | 7 |
|  |  |  | **<**hsa-miR-21* | 6 |
| miR-50 GAUAUGU | cel-miR-50 | | hsa-miR-190 | 9 |
|  |  |  | hsa-miR-190b | 9 |
| miR-51 ACCCGUA | cel-miR-51 **(5’)** | | hsa-miR-99a | 8 |
|  |  |  | hsa-miR-99b | 8 |
|  |  |  | hsa-miR-100 | 8 |
| miR-52 ACCCGUA | cel-miR-52 **(5’)** | | hsa-miR-99b | 9 |
|  |  |  | hsa-miR-99a | 8 |
|  |  |  | hsa-miR-100 | 8 |
| miR-53 ACCCGUA | cel-miR-53 **(5’)** | | hsa-miR-99b | 9 |
|  |  |  | hsa-miR-99a | 8 |
|  |  |  | hsa-miR-100 | 8 |
| miR-54  *ACCCGUA* | cel-miR-54 **(5’)** | | hsa-miR-99a | 7 |
|  |  |  | hsa-miR-99b | 7 |
|  |  |  | hsa-miR-100 | 7 |
| miR-55  *ACCCGUA* | cel-miR-55 **(5’)** | | hsa-miR-99a | 8 |
|  |  |  | hsa-miR-99b | 8 |
|  |  |  | hsa-miR-100 | 8 |
| miR-56  *ACCCGUA* | cel-miR-56 **(5’)** | | hsa-miR-99a | 7 |
|  |  |  | hsa-miR-99b | 7 |
|  |  |  | hsa-miR-100 | 7 |
| miR-57  *A/GCCCUGU* | cel-miR-57 **(5’)** | | hsa-miR-10a | 10 |
|  |  |  | hsa-miR-10b | 10 |
|  |  |  | **<**hsa-miR-146b-3p | 8 |
| miR-58  *UGA/GGAUC* | cel-miR-58 **(5’)** | | **<**hsa-miR-450b-3p | 7 |
| miR-61  *(U)G/AACUA/G(A)* | cel-miR-61 **(5’)** | | **<**hsa-miR-708* | 6 |
|  |  |  | **<**hsa-miR-134 | 6 |
| miR-62 GAUAUGU | cel-miR-62 **(5’)** | | hsa-miR-190 | 8 |
|  |  |  | hsa-miR-190b | 8 |
| miR-63 UG/AA/GCACU | cel-miR-63 **(5’)** | | **<**hsa-miR-183 | 9 |
|  |  |  | **<**hsa-miR-96 | 7 |
|  |  |  | **<**hsa-miR-200a | 7 |
|  |  |  | hsa-miR-514 | 7 |
| miR-64 UG/AA/GCACU | cel-miR-64 **(5’)** | | **<**hsa-miR-183 | 9 |
|  |  |  | **<**hsa-miR-96 | 7 |
|  |  |  | **<**hsa-miR-200a | 7 |
|  |  |  | hsa-miR-514 | 7 |
| miR-65  *UG/AA/GCACU* | cel-miR-65 **(5’)** | | **<**hsa-miR-183 | 9 |
|  |  |  | **<**hsa-miR-96 | 7 |
|  |  |  | **<**hsa-miR-200a | 7 |
|  |  |  | hsa-miR-514 | 7 |
| miR-66 UG/AA/GCACU | cel-miR-66 **(5’)** | | **<**hsa-miR-183 | 8 |
|  |  |  | hsa-miR-514 | 7 |
|  |  |  | **<**hsa-miR-96 | 6 |
|  |  |  | **<**hsa-miR-200a | 6 |
| miR-72 GGCAAGA | cel-miR-72 | | hsa-miR-31 | 10 |
| miR-73 GGCAAGA | cel-miR-73 **(5’)** | | hsa-miR-31 | 9 |
| miR-74  *(G)G/ACAAGA/G(A)* | cel-miR-74 **(5’)** | | hsa-miR-31 | 7 |
|  |  |  | **<**hsa-miR-513b | 6 |
|  |  |  | **<**hsa-miR-873 | 6 |
| miR-75  *AAAG/ACUA/G* | cel-miR-75 **(5’)** | | hsa-miR-9* | 8 |
|  |  |  | **<**hsa-miR-320 | 6 |
|  |  |  | **<**hsa-miR-548a-3p | 5 |
| miR-79 (AU)AAA/GG/AC(UA/G) | cel-miR-79 **(5’)** | | hsa-miR-9* | 10 |
|  |  |  | hsa-miR-340 | 7 |
|  |  |  | **<**hsa-miR-320 | 7 |
|  |  |  | **<**hsa-miR-548a-3p | 6 |
|  |  |  | **<**hsa-miR-7 | 5 |
| miR-80  *UGA/GGAUC* | cel-miR-80 **(5’)** | | **<**hsa-miR-450b-3p | 8 |
| miR-81  *UGA/GGAUC* | cel-miR-81 **(5’)** | | **<**hsa-miR-450b-3p | 8 |
| miR-82  *UGA/GGAUC* | cel-miR-82 **(5’)** | | **<**hsa-miR-450b-3p | 8 |
| miR-83 A/GG/ACACCA | cel-miR-83 **(5’)** | | hsa-miR-29a | 9 |
|  |  |  | hsa-miR-29b | 9 |
|  |  |  | hsa-miR-29c | 9 |
|  |  |  | **<**hsa-miR-21* | 6 |
|  |  |  | **<**hsa-miR-593* | 6 |
| miR-84 (G)AGGUAG(U) | cel-miR-84 **(5’)** | | hsa-let-7a | 10 |
|  |  |  | hsa-let-7b | 10 |
|  |  |  | hsa-let-7c | 10 |
|  |  |  | hsa-let-7f | 10 |
|  |  |  | hsa-let-7g | 10 |
|  |  |  | hsa-let-7i | 10 |
|  |  |  | hsa-miR-98 | 10 |
|  |  |  | hsa-let-7d | 9 |
|  |  |  | hsa-let-7e | 9 |
|  |  |  | hsa-miR-196a | 7 |
|  |  |  | hsa-miR-196b | 7 |
| miR-86  *AGUG/AAAU* | cel-miR-86 **(5’)** | | **<**hsa-miR-545* | 8 |
|  |  |  | **<**hsa-miR-559 | 7 |
| miR-90  *GAUAUGU* | cel-miR-90 **(5’)** | | hsa-miR-190 | 9 |
|  |  |  | hsa-miR-190b | 9 |
| miR-124 AAGGCAC | cel-miR-124 **(5’)** | | hsa-miR-124 | 10 |
|  |  |  | hsa-miR-506 | 9 |
| miR-228  *UG/AG/ACACU* | cel-miR-228 **(5’)** | | hsa-miR-183 | 9 |
|  |  |  | **<**hsa-miR-96 | 8 |
|  |  |  | **<**hsa-miR-514 | 7 |
|  |  |  | **<**hsa-miR-200a | 6 |
| miR-229  *UG/AA/GCACU* | cel-miR-229 **(5’)** | | hsa-miR-514 | 8 |
|  |  |  | **<**hsa-miR-183 | 8 |
|  |  |  | **<**hsa-miR-96 | 6 |
|  |  |  | **<**hsa-miR-200a | 6 |
| miR-231  *A/GAGCUCG/A* | cel-miR-231 **(5’)** | | hsa-miR-99b* | 9 |
|  |  |  | hsa-miR-99a* | 7 |
|  |  |  | **<**hsa-miR-556-5p | 6 |
| miR-232 (U)AAA/GUGC(A) | cel-miR-232 **(5’)** | | **<**hsa-miR-519a | 8 |
|  |  |  | **<**hsa-miR-519b-3p | 8 |
|  |  |  | **<**hsa-miR-519c-3p | 8 |
|  |  |  | **<**hsa-miR-302a | 8 |
|  |  |  | **<**hsa-miR-302b | 8 |
|  |  |  | **<**hsa-miR-302c | 8 |
|  |  |  | **<**hsa-miR-302d | 8 |
| miR-234  *UUAUUG/AC* | cel-miR-234 **(5’)** | | hsa-miR-137 | 8 |
|  |  |  | **<**hsa-miR-126* | 7 |
| miR-235 AUUG/ACAC | cel-miR-235 **(5’)** | | hsa-miR-92b | 10 |
|  |  |  | hsa-miR-92a | 9 |
|  |  |  | hsa-miR-25 | 8 |
|  |  |  | hsa-miR-32 | 8 |
|  |  |  | hsa-miR-367 | 8 |
|  |  |  | hsa-miR-363 | 7 |
|  |  |  | **<**hsa-miR-885-5p | 6 |
| miR-236 UAAUACU | cel-miR-236 | | hsa-miR-429 | 10 |
|  |  |  | hsa-miR-200b | 9 |
|  |  |  | hsa-miR-200c | 9 |
| miR-237 CCCUGA/GG | cel-miR-237 **(5’)** | | hsa-miR-125a-5p | 9 |
|  |  |  | hsa-miR-125b | 9 |
|  |  |  | **<**hsa-miR-331-3p | 6 |
| miR-240  *ACUGGCC* | cel-miR-240 **(5’)** | | hsa-miR-193b | 8 |
|  |  |  | hsa-miR-193a-3p | 7 |
| miR-241 GAGGUAG | cel-miR-241 **(5’)** | | hsa-let-7e | 9 |
|  |  |  | hsa-let-7a | 8 |
|  |  |  | hsa-let-7b | 8 |
|  |  |  | hsa-let-7c | 8 |
|  |  |  | hsa-let-7f | 8 |
|  |  |  | hsa-let-7g | 8 |
|  |  |  | hsa-let-7i | 8 |
|  |  |  | hsa-miR-98 | 8 |
|  |  |  | hsa-let-7d | 7 |
| miR-244  *UCUUUGG* | cel-miR-244 **(5’)** | | hsa-miR-9 | 9 |
| miR-245 UUGGUCC | cel-miR-245 | | hsa-miR-133a | 9 |
|  |  |  | hsa-miR-133b | 9 |
| miR-247  *(U)G/AACUA/GG(A)* | cel-miR-247 **(5’)** | | **<**hsa-miR-708* | 6 |
|  |  |  | **<**hsa-miR-134 | 6 |
| miR-250  *(A)UCACAG/A(U)* | cel-miR-250 **(5’)** | | hsa-miR-27a | 7 |
|  |  |  | hsa-miR-27b | 7 |
|  |  |  | hsa-miR-128 | 7 |
|  |  |  | hsa-miR-499-3p | 7 |
|  |  |  | **<**hsa-miR-768-3p | 6 |
| miR-251  *AAGUAG/AU* | cel-miR-251 **(5’)** | | **<**hsa-miR-26a | 7 |
|  |  |  | **<**hsa-miR-26b | 7 |
| miR-252  *AAGUAG/AU* | cel-miR-252 **(5’)** | | **<**hsa-miR-26a | 6 |
|  |  |  | **<**hsa-miR-26b | 6 |
| miR-254 UGCAAAU | cel-miR-254 **(5’)** | | hsa-miR-19a | 8 |
|  |  |  | hsa-miR-19b | 8 |
| miR-256  *(UGG)AA/GUG(CAU)* | cel-miR-256 **(5’)** | | hsa-miR-1 | 8 |
|  |  |  | hsa-miR-206 | 8 |
|  |  |  | **<**hsa-miR-122 | 6 |
|  |  |  | **<**hsa-miR-519a | 6 |
|  |  |  | **<**hsa-miR-519b-3p | 6 |
|  |  |  | **<**hsa-miR-519c-3p | 6 |
| miR-259  *AAUCUCA* | cel-miR-259 **(5’)** | | hsa-miR-216a | 8 |
|  |  |  | hsa-miR-216b | 8 |
| miR-266  *AGGCAAG* | cel-miR-266 | | hsa-miR-31 | 8 |
| miR-267  *CCCGUG/AA/G* | cel-miR-267 **(5’)** | | **<**hsa-miR-99a | 6 |
|  |  |  | **<**hsa-miR-99b | 6 |
|  |  |  | **<**hsa-miR-100 | 6 |
| miR-268  *(U)GCAG/AGA(A)* | cel-miR-268 **(5’)** | | hsa-miR-31 | 7 |
|  |  |  | **<**hsa-miR-873 | 7 |
| miR-269  *GGCAAGA* | cel-miR-269 | | hsa-miR-31 | 7 |
| miR-273 G/ACCCGUA | cel-miR-273 **(5’)** | | **<**hsa-miR-99a | 6 |
|  |  |  | **<**hsa-miR-99b | 6 |
|  |  |  | **<**hsa-miR-100 | 6 |
| miR-357  *UAAA/GUGC* | cel-miR-357 **(5’)** | | **<**hsa-miR-302a | 6 |
|  |  |  | **<**hsa-miR-302b | 6 |
|  |  |  | **<**hsa-miR-302c | 6 |
|  |  |  | **<**hsa-miR-302d | 6 |
| miR-785 AGUG/AAAU | cel-miR-785 **(5’)** | | **<**hsa-miR-545* | 7 |
|  |  |  | **<**hsa-miR-559 | 7 |
| miR-786 (AA)UGCCC(UG/A) | cel-miR-786 **(5’)** | | hsa-miR-365 | 8 |
|  |  |  | hsa-miR-18a* | 7 |
|  |  |  | hsa-miR-18b* | 6 |
| miR-787  *A/GAGCUCG/A* | cel-miR-787 **(5’)** | | hsa-miR-99a* | 8 |
|  |  |  | hsa-miR-99b* | 8 |
|  |  |  | **<**hsa-miR-556-5p | 6 |
| miR-790 UG/AG/ACACU | cel-miR-790 **(5’)** | | hsa-miR-96 | 8 |
|  |  |  | hsa-miR-183 | 7 |
|  |  |  | **<**hsa-miR-514 | 7 |
|  |  |  | **<**hsa-miR-200a | 5 |
| miR-791  *(UU)UG/AG/ACA(CU)* | cel-miR-791 **(5’)** | | hsa-miR-96 | 9 |
|  |  |  | hsa-miR-182 | 8 |
|  |  |  | hsa-miR-183 | 8 |
|  |  |  | **<**hsa-miR-514 | 7 |
|  |  |  | **<**hsa-miR-200a | 5 |
| miR-793  *(U)GAGGUA(U)* | cel-miR-793 **(5’)** | | hsa-let-7a | 7 |
|  |  |  | hsa-let-7b | 7 |
|  |  |  | hsa-let-7c | 7 |
|  |  |  | hsa-let-7e | 7 |
|  |  |  | hsa-let-7f | 7 |
|  |  |  | hsa-let-7g | 7 |
|  |  |  | hsa-let-7i | 7 |
|  |  |  | hsa-miR-98 | 7 |
|  |  |  | hsa-miR-202 | 7 |
| miR-794 (G)AGGUAA/G(U) | cel-miR-794 **(5’)** | | hsa-let-7a | 8 |
|  |  |  | hsa-let-7b | 8 |
|  |  |  | hsa-let-7c | 8 |
|  |  |  | hsa-let-7f | 8 |
|  |  |  | hsa-let-7g | 8 |
|  |  |  | hsa-let-7i | 8 |
|  |  |  | hsa-miR-98 | 8 |
|  |  |  | hsa-let-7d | 7 |
|  |  |  | hsa-let-7e | 7 |
|  |  |  | **<**hsa-miR-196a | 6 |
| miR-795 GAGGUAG | cel-miR-795 **(5’)** | | hsa-let-7a | 8 |
|  |  |  | hsa-let-7b | 8 |
|  |  |  | hsa-let-7c | 8 |
|  |  |  | hsa-let-7e | 8 |
|  |  |  | hsa-let-7f | 8 |
|  |  |  | hsa-let-7g | 8 |
|  |  |  | hsa-let-7i | 8 |
|  |  |  | hsa-miR-98 | 8 |
|  |  |  | hsa-let-7d | 7 |
| miR-796 UGGAA/GUG | cel-miR-796 **(5’)** | | hsa-miR-1 | 9 |
|  |  |  | hsa-miR-206 | 9 |
|  |  |  | **<**hsa-miR-122 | 7 |
| miR-797  *AUCACAG* | cel-miR-797 **(5’)** | | hsa-miR-499-3p | 7 |
| miR-1018 GA/GGAUCA | cel-miR-1018 **(5’)** | | **<**hsa-miR-450b-3p | 6 |
| miR-1020  *A/GUUCUGU* | cel-miR-1020 **(5’)** | | hsa-miR-148b* | 6 |
| miR-1022  *A/GGAUCAU* | cel-miR-1022 **(5’)** | | **<**hsa-miR-450b-3p | 6 |

**Supplementary Alignments S10:**

**5’ sequence alignments of *C. elegans* and *H. sapiens* miRNAs with significant identity at the 5’ end (10nt).** Members of a group have ≥7 continuous nt of homology with at least one other group member. Nucleotides at the end of sequences indicate the number of residues identical to the related *C. elegans* miRNA. Grey shading denotes potential G..U pairing. Superscript “less than” (**<**) symbol before miRNA name indicates allowed A-G base changes (G..U pairing) interrupting the 7nt homology block at the 5' 10nt which groups miRNAs into families. Superscript (5’) labels 69 worm miRNAs with 5’ homology but weak extended identity (<70%) to some of their 5’-related human sequences. 41 of these worm miRNAs (highlighted in blue) have <60% overall similarity with all their 5’-related human miRNAs.

**let-7: cel-let-7 ^(5’)^, hsa-let-7a, hsa-let-7b, hsa-let-7c, hsa-let-7d,**

**hsa-let-7e, hsa-let-7f, hsa-let-7g, hsa-let-7i,**

**hsa-miR-98, hsa-miR-196a, hsa-miR-196b**

1

cel-let-7 UGAGGUAGUA

hsa-let-7a UGAGGUAGUA 10nt

hsa-let-7b UGAGGUAGUA 10nt

hsa-let-7c UGAGGUAGUA 10nt

hsa-let-7d AGAGGUAGUA 9nt

hsa-let-7e UGAGGUAGGA 9nt

hsa-let-7f UGAGGUAGUA 10nt

hsa-let-7g UGAGGUAGUA 10nt

hsa-let-7i UGAGGUAGUA 10nt

hsa-miR-98 UGAGGUAGUA 10nt

hsa-miR-196a -UAGGUAGUUU 7nt

hsa-miR-196b -UAGGUAGUUU 7nt

**lin-4: cel-lin-4 ^(5’)^, hsa-miR-125a-5p, hsa-miR-125b,**

**<hsa-miR-331-3p**

1

cel-lin-4 -UCCCUGAGAC

hsa-miR-125a-5p -UCCCUGAGAC 10nt

hsa-miR-125b -UCCCUGAGAC 10nt

hsa-miR-331-3p GCCCCUGGGC- 6nt

**miR-1: cel-miR-1 ^(5’)^, hsa-miR-1, hsa-miR-206, <hsa-miR-122**

1 10

cel-miR-1 UGGAAUGUAA

hsa-miR-1 UGGAAUGUAA 10nt

hsa-miR-206 UGGAAUGUAA 10nt

hsa-miR-122 UGGAGUGUGA 8nt

**miR-2: cel-miR-2 ^(5’)^, hsa-miR-499-3p**

1

cel-miR-2 --UAUCACAGCC

hsa-miR-499-3p AACAUCACAG-- 7nt

**miR-34: cel-miR-34, hsa-miR-34, hsa-miR-34b*,**

**hsa-miR-34c-5p, hsa-miR-449a, hsa-miR-449b**

1 10

cel-miR-34 -AGGCAGUGUG

hsa-miR-34 -UGGCAGUGUC 8nt

hsa-miR-449a -UGGCAGUGUA 8nt

hsa-miR-449b -AGGCAGUGUA 9nt

hsa-miR-34b* UAGGCAGUGU- 9nt

hsa-miR-34c-5p -AGGCAGUGUA 9nt

**miR-43: cel-miR-43 ^(5’)^, hsa-miR-27a, hsa-miR-27b,**

**hsa-miR-128, hsa-miR-499-3p, <hsa-miR-768-3p**

1

cel-miR-43 --UAUCACAGUU--

hsa-miR-27a ---UUCACAGUGG- 7nt

hsa-miR-27b ---UUCACAGUGG- 7nt

hsa-miR-128 ----UCACAGUGAA 7nt

hsa-miR-499-3p AACAUCACAG---- 7nt

hsa-miR-768-3p ----UCACAAUGCU 6nt

**miR-44: cel-miR-44 ^(5’)^, <hsa-miR-134, <hsa-miR-708***

1

cel-miR-44 --UGACUAGAGA

hsa-miR-134 UGUGACUGGU-- 6nt

hsa-miR-708* --CAACUAGACU 6nt

**miR-45: cel-miR-45 ^(5’)^, <hsa-miR-134, <hsa-miR-708***

1

cel-miR-45 --UGACUAGAGA

hsa-miR-134 UGUGACUGGU-- 6nt

hsa-miR-708* --CAACUAGACU 6nt

**miR-48: cel-miR-48 ^(5’)^, hsa-let-7a, hsa-let-7b, hsa-let-7c,**

**hsa-let-7d, hsa-let-7e, hsa-let-7f, hsa-let-7g,**

**hsa-let-7i, hsa-miR-98**

1 10

cel-miR-48 UGAGGUAGGC

hsa-let-7a UGAGGUAGUA 8nt

hsa-let-7b UGAGGUAGUA 8nt

hsa-let-7c UGAGGUAGUA 8nt

hsa-let-7d AGAGGUAGUA 7nt

hsa-let-7e UGAGGUAGGA 9nt

hsa-let-7f UGAGGUAGUA 8nt

hsa-let-7g UGAGGUAGUA 8nt

hsa-let-7i UGAGGUAGUA 8nt

hsa-miR-98 UGAGGUAGUA 8nt

**miR-49: cel-miR-49 ^(5’)^, <hsa-miR-21* , hsa-miR-29a,**

**hsa-miR-29b, hsa-miR-29c, <hsa-miR-593***

1 10

cel-miR-49 AAGCACCACG

hsa-miR-29a UAGCACCAUC 7nt

hsa-miR-29b UAGCACCAUU 7nt

hsa-miR-29c UAGCACCAUU 7nt

hsa-miR-593* AGGCACCAGC 7nt

hsa-miR-21* CAACACCAGU 6nt

**miR-50: cel-miR-50, hsa-miR-190, hsa-miR-190b**

1 10

cel-miR-50 UGAUAUGUCU

hsa-miR-190 UGAUAUGUUU 9nt

hsa-miR-190b UGAUAUGUUU 9nt

**miR-51: cel-miR-51 ^(5’)^, hsa-miR-99a, hsa-miR-99b,**

**hsa-miR-100**

1 10

cel-miR-51 UACCCGUAGC

hsa-miR-99a AACCCGUAGA 8nt

hsa-miR-99b CACCCGUAGA 8nt

hsa-miR-100 AACCCGUAGA 8nt

**miR-52: cel-miR-52 ^(5’)^, hsa-miR-99a, hsa-miR-99b,**

**hsa-miR-100**

1 10

cel-miR-52 CACCCGUACA

hsa-miR-99a AACCCGUAGA 8nt

hsa-miR-99b CACCCGUAGA 9nt

hsa-miR-100 AACCCGUAGA 8nt

**miR-53: cel-miR-53 ^(5’)^, hsa-miR-99a, hsa-miR-99b,**

**hsa-miR-100**

1 10

cel-miR-53 CACCCGUACA

hsa-miR-99a AACCCGUAGA 8nt

hsa-miR-99b CACCCGUAGA 9nt

hsa-miR-100 AACCCGUAGA 8nt

**miR-54: cel-miR-54 ^(5’)^, hsa-miR-99a, hsa-miR-99b,**

**hsa-miR-100**

1 10

cel-miR-54 UACCCGUAAU

hsa-miR-100 AACCCGUAGA 7nt

hsa-miR-99a AACCCGUAGA 7nt

hsa-miR-99b CACCCGUAGA 7nt

**miR-55: cel-miR-55 ^(5’)^, hsa-miR-99a, hsa-miR-99b,**

**hsa-miR-100**

1 10

cel-miR-55 UACCCGUAUA

hsa-miR-99a AACCCGUAGA 8nt

hsa-miR-99b CACCCGUAGA 8nt

hsa-miR-100 AACCCGUAGA 8nt

**miR-56: cel-miR-56 ^(5’)^, hsa-miR-99a, hsa-miR-99b,**

**hsa-miR-100**

1 10

cel-miR-56 UACCCGUAAU

hsa-miR-99a AACCCGUAGA 7nt

hsa-miR-99b CACCCGUAGA 7nt

hsa-miR-100 AACCCGUAGA 7nt

**miR-57: cel-miR-57 ^(5’)^, hsa-miR-10a, hsa-miR-10b,**

**<hsa-miR-146b-3p**

1 10

cel-miR-57 UACCCUGUAG

hsa-miR-10a UACCCUGUAG 10nt

hsa-miR-10b UACCCUGUAG 10nt

hsa-miR-146b-3p UGCCCUGUGG 8nt

**miR-58: cel-miR-58 ^(5’)^, <hsa-miR-450b-3p**

1

cel-miR-58 -UGAGAUCGUU

hsa-miR-450b-3p UUGGGAUCAU- 7nt

**miR-61: cel-miR-61 ^(5’)^, <hsa-miR-708*, <hsa-miR-134**

1

cel-miR-61 --UGACUAGAAC

hsa-miR-134 UGUGACUGGU-- 6nt

hsa-miR-708* --CAACUAGACU 6nt

**miR-62: cel-miR-62 ^(5’)^, hsa-miR-190, hsa-miR-190b**

1 10

cel-miR-62 UGAUAUGUAA

hsa-miR-190 UGAUAUGUUU 8nt

hsa-miR-190b UGAUAUGUUU 8nt

**miR-63: cel-miR-63 ^(5’)^, <hsa-miR-96, <hsa-miR-183,**

**<hsa-miR-200a, hsa-miR-514**

1

cel-miR-63 UAUGACACUG--

hsa-miR-183 UAUGGCACUG-- 9nt

hsa-miR-96 UUUGGCACUA-- 7nt

hsa-miR-200a --UAACACUGUC 7nt

hsa-miR-514 AUUGACACUU-- 7nt

**miR-64: cel-miR-64 ^(5’)^, <hsa-miR-96, <hsa-miR-183,**

**<hsa-miR-200a, hsa-miR-514**

1

cel-miR-64 UAUGACACUG--

hsa-miR-183 UAUGGCACUG-- 9nt

hsa-miR-96 UUUGGCACUA-- 7nt

hsa-miR-200a --UAACACUGUC 7nt

hsa-miR-514 AUUGACACUU-- 7nt

**miR-65: cel-miR-65 ^(5’)^, <hsa-miR-96, <hsa-miR-183,**

**<hsa-miR-200a, hsa-miR-514**

1

cel-miR-65 UAUGACACUG--

hsa-miR-183 UAUGGCACUG-- 9nt

hsa-miR-96 UUUGGCACUA-- 7nt

hsa-miR-200a --UAACACUGUC 7nt

hsa-miR-514 AUUGACACUU-- 7nt

**miR-66: cel-miR-66 ^(5’)^, <hsa-miR-96, <hsa-miR-183,**

**<hsa-miR-200a, hsa-miR-514**

1

cel-miR-66 CAUGACACUG--

hsa-miR-200a --UAACACUGUC 6nt

hsa-miR-183 UAUGGCACUG-- 8nt

hsa-miR-96 UUUGGCACUA-- 6nt

hsa-miR-514 AUUGACACUU-- 7nt

**miR-72: cel-miR-72, hsa-miR-31**

1 10

cel-miR-72 AGGCAAGAUG

hsa-miR-31 AGGCAAGAUG 10nt

**miR-73: cel-miR-73 ^(5’)^, hsa-miR-31**

1 10

cel-miR-73 UGGCAAGAUG

hsa-miR-31 AGGCAAGAUG 9nt

**miR-74: cel-miR-74 ^(5’)^, hsa-miR-31, <hsa-miR-513b,**

**<hsa-miR-873**

1

cel-miR-74 -UGGCAAGAAA--

hsa-miR-513b UUCACAAGGA--- 6nt

hsa-miR-31 -AGGCAAGAUG-- 7nt

hsa-miR-873 ---GCAGGAACUU 6nt

**miR-75: cel-miR-75 ^(5’)^, hsa-miR-9*, <hsa-miR-320,**

**<hsa-miR-548a-3p**

1

cel-miR-75 UUAAAGCUAC-

hsa-miR-9* AUAAAGCUAG- 8nt

hsa-miR-320 -AAAAGCUGGG 6nt

hsa-miR-548a-3p -CAAAACUGGC 5nt

**miR-79: cel-miR-79 ^(5’)^, <hsa-miR-7, hsa-miR-9*, hsa-miR-340,**

**<hsa-miR-320, <hsa-miR-548a-3p**

1

cel-miR-79 --AUAAAGCUAG-

hsa-miR-320 ---AAAAGCUGGG 7nt

hsa-miR-548a-3p ---CAAAACUGGC 6nt

hsa-miR-7 -UGGAAGACUA-- 5nt

hsa-miR-340 UUAUAAAGCA--- 7nt

hsa-miR-9* --AUAAAGCUAG- 10nt

**miR-80: cel-miR-80 ^(5’)^, <hsa-miR-450b-3p**

1

cel-miR-80 -UGAGAUCAUU

hsa-miR-450b-3p UUGGGAUCAU- 8nt

**miR-81: cel-miR-81 ^(5’)^, <hsa-miR-450b-3p**

1

cel-miR-81 -UGAGAUCAUC

hsa-miR-450b-3p UUGGGAUCAU- 8nt

**miR-82: cel-miR-82 ^(5’)^, <hsa-miR-450b-3p**

1

cel-miR-82 -UGAGAUCAUC

hsa-miR-450b-3p UUGGGAUCAU- 8nt

**miR-83: cel-miR-83 ^(5’)^, <hsa-miR-21*, hsa-miR-29a,**

**hsa-miR-29b, hsa-miR-29c, <hsa-miR-593***

1 10

cel-miR-83 UAGCACCAUA

hsa-miR-29a UAGCACCAUC 9nt

hsa-miR-29c UAGCACCAUU 9nt

hsa-miR-29b UAGCACCAUU 9nt

hsa-miR-21* CAACACCAGU 6nt

hsa-miR-593* AGGCACCAGC 6nt

**miR-84: cel-miR-84 ^(5’)^, hsa-let-7a, hsa-let-7b, hsa-let-7c,**

**hsa-let-7d, hsa-let-7e, hsa-let-7f, hsa-let-7g,**

**hsa-let-7i, hsa-miR-98, hsa-miR-196a, hsa-miR-196b**

1 10

cel-miR-84 UGAGGUAGUA

hsa-let-7a UGAGGUAGUA 10nt

hsa-let-7b UGAGGUAGUA 10nt

hsa-let-7c UGAGGUAGUA 10nt

hsa-let-7d AGAGGUAGUA 9nt

hsa-let-7e UGAGGUAGGA 9nt

hsa-let-7f UGAGGUAGUA 10nt

hsa-let-7g UGAGGUAGUA 10nt

hsa-let-7i UGAGGUAGUA 10nt

hsa-miR-98 UGAGGUAGUA 10nt

hsa-miR-196a -UAGGUAGUUU 7nt

hsa-miR-196b -UAGGUAGUUU 7nt

**miR-86: cel-miR-86 ^(5’)^, <hsa-miR-545*, <hsa-miR-559**

1

cel-miR-86 -UAAGUGAAUG

hsa-miR-545* -UCAGUAAAUG 8nt

hsa-miR-559 UAAAGUAAAU- 7nt

**miR-90: cel-miR-90 ^(5’)^, hsa-miR-190, hsa-miR-190b**

1 10

cel-miR-90 UGAUAUGUUG

hsa-miR-190 UGAUAUGUUU 9nt

hsa-miR-190b UGAUAUGUUU 9nt

**miR-124: cel-miR-124 ^(5’)^, hsa-miR-124, hsa-miR-506**

1 10

cel-miR-124 UAAGGCACGC

hsa-miR-124 UAAGGCACGC 10nt

hsa-miR-506 UAAGGCACCC 9nt

**miR-228: cel-miR-228 ^(5’)^, <hsa-miR-96, hsa-miR-183,**

**<hsa-miR-200a, <hsa-miR-514**

1

cel-miR-228 AAUGGCACUG--

hsa-miR-183 UAUGGCACUG-- 9nt

hsa-miR-200a --UAACACUGUC 6nt

hsa-miR-514 AUUGACACUU-- 7nt

hsa-miR-96 UUUGGCACUA-- 8nt

**miR-229: cel-miR-229 ^(5’)^,** **<hsa-miR-96, <hsa-miR-183,**

**<hsa-miR-200a, hsa-miR-514**

1

cel-miR-229 AAUGACACUG--

hsa-miR-200a --UAACACUGUC 6nt

hsa-miR-183 UAUGGCACUG-- 8nt

hsa-miR-96 UUUGGCACUA-- 6nt

hsa-miR-514 AUUGACACUU-- 8nt

**miR-231: cel-miR-231 ^(5’)^, hsa-miR-99a*, hsa-miR-99b*,**

**<hsa-miR-556-5p**

1

cel-miR-231 --UAAGCUCGUG

hsa-miR-99b* --CAAGCUCGUG 9nt

hsa-miR-556-5p GAUGAGCUCA-- 6nt

hsa-miR-99a* --CAAGCUCGCU 7nt

**miR-232: cel-miR-232 ^(5’)^, <hsa-miR-519a, <hsa-miR-519b-3p,**

**<hsa-miR-519c-3p, <hsa-miR-302a, <hsa-miR-302b,**

**<hsa-miR-302c, <hsa-miR-302d**

1 10

cel-miR-232 UAAAUGCAUC

hsa-miR-519a AAAGUGCAUC 8nt

hsa-miR-519b-3p AAAGUGCAUC 8nt

hsa-miR-519c-3p AAAGUGCAUC 8nt

hsa-miR-302a UAAGUGCUUC 8nt

hsa-miR-302b UAAGUGCUUC 8nt

hsa-miR-302c UAAGUGCUUC 8nt

hsa-miR-302d UAAGUGCUUC 8nt

**miR-234: cel-miR-234 ^(5’)^, <hsa-miR-126*, hsa-miR-137**

1

cel-miR-234 --UUAUUGCUCG

hsa-miR-126* CAUUAUUACU-- 7nt

hsa-miR-137 --UUAUUGCUUA 8nt

**miR-235: cel-miR-235 ^(5’)^, hsa-miR-25, hsa-miR-32,**

**hsa-miR-92a, hsa-miR-92b, hsa-miR-363,**

**hsa-miR-367, <hsa-miR-885-5p**

1

cel-miR-235 --UAUUGCACUC

hsa-miR-25 --CAUUGCACUU 8nt

hsa-miR-32 --UAUUGCACAU 8nt

hsa-miR-92a --UAUUGCACUU 9nt

hsa-miR-92b --UAUUGCACUC 10nt

hsa-miR-363 --AAUUGCACGG 7nt

hsa-miR-367 --AAUUGCACUU 8nt

hsa-miR-885-5p UCCAUUACAC-- 6nt

**miR-236: cel-miR-236, hsa-miR-200b, hsa-miR-200c,**

**hsa-miR-429**

1 10

cel-miR-236 UAAUACUGUC

hsa-miR-200b UAAUACUGCC 9nt

hsa-miR-200c UAAUACUGCC 9nt

hsa-miR-429 UAAUACUGUC 10nt

**miR-237: cel-miR-237 ^(5’)^, hsa-miR-125a-5p, hsa-miR-125b,**

**<hsa-miR-331-3p**

1

cel-miR-237 -UCCCUGAGAA

hsa-miR-125a-5p -UCCCUGAGAC 9nt

hsa-miR-125b -UCCCUGAGAC 9nt

hsa-miR-331-3p GCCCCUGGGC- 6nt

**miR-240: cel-miR-240 ^(5’)^, hsa-miR-193a-3p, hsa-miR-193b**

1 10

cel-miR-240 UACUGGCCCC

hsa-miR-193a-3p AACUGGCCUA 7nt

hsa-miR-193b AACUGGCCCU 8nt

**miR-241: cel-miR-241 ^(5’)^, hsa-let-7a, hsa-let-7b, hsa-let-7c,**

**hsa-let-7d, hsa-let-7e, hsa-let-7f, hsa-let-7g,**

**hsa-let-7i, hsa-miR-98**

1 10

cel-miR-241 UGAGGUAGGU

hsa-let-7a UGAGGUAGUA 8nt

hsa-let-7b UGAGGUAGUA 8nt

hsa-let-7c UGAGGUAGUA 8nt

hsa-let-7d AGAGGUAGUA 7nt

hsa-let-7e UGAGGUAGGA 9nt

hsa-let-7f UGAGGUAGUA 8nt

hsa-let-7g UGAGGUAGUA 8nt

hsa-let-7i UGAGGUAGUA 8nt

hsa-miR-98 UGAGGUAGUA 8nt

**miR-244: cel-miR-244 ^(5’)^, hsa-miR-9**

1 10

cel-miR-244 UCUUUGGUUG

hsa-miR-9 UCUUUGGUUA 9nt

**miR-245: cel-miR-245, hsa-miR-133a, hsa-miR-133b**

1

cel-miR-245 AUUGGUCCCC-

hsa-miR-133a -UUGGUCCCCU 9nt

hsa-miR-133b -UUGGUCCCCU 9nt

**miR-247: cel-miR-247 ^(5’)^, <hsa-miR-708*, <hsa-miR-134**

1

cel-miR-247 --UGACUAGAGC

hsa-miR-134 UGUGACUGGU-- 6nt

hsa-miR-708* --CAACUAGACU 6nt

**miR-250: cel-miR-250 ^(5’)^, hsa-miR-27a, hsa-miR-27b,**

**hsa-miR-128, hsa-miR-499-3p, <hsa-miR-768-3p**

1

cel-miR-250 --AAUCACAGUC--

hsa-miR-128 ----UCACAGUGAA 7nt

hsa-miR-499-3p AACAUCACAG---- 7nt

hsa-miR-27a ---UUCACAGUGG- 7nt

hsa-miR-27b ---UUCACAGUGG- 7nt

hsa-miR-768-3p ----UCACAAUGCU 6nt

**miR-251: cel-miR-251 ^(5’)^, <hsa-miR-26a, <hsa-miR-26b**

1

cel-miR-251 -UUAAGUAGUG

hsa-miR-26a UUCAAGUAAU- 7nt

hsa-miR-26b UUCAAGUAAU- 7nt

**miR-252: cel-miR-252 ^(5’)^, <hsa-miR-26a, <hsa-miR-26b**

1

cel-miR-252 -AUAAGUAGUA

hsa-miR-26a UUCAAGUAAU- 6nt

hsa-miR-26b UUCAAGUAAU- 6nt

**miR-254: cel-miR-254 ^(5’)^, hsa-miR-19a, hsa-miR-19b**

1

cel-miR-254 --UGCAAAUCUU

hsa-miR-19a UGUGCAAAUC-- 8nt

hsa-miR-19b UGUGCAAAUC-- 8nt

**miR-256: cel-miR-256 ^(5’)^, hsa-miR-1, <hsa-miR-122,**

**hsa-miR-206, <hsa-miR-519a, <hsa-miR-519b-3p,**

**<hsa-miR-519c-3p**

1

cel-miR-256 UGGAAUGCAU-

hsa-miR-1 UGGAAUGUAA- 8nt

hsa-miR-122 UGGAGUGUGA- 6nt

hsa-miR-206 UGGAAUGUAA- 8nt

hsa-miR-519a -AAAGUGCAUC 6nt

hsa-miR-519b-3p -AAAGUGCAUC 6nt

hsa-miR-519c-3p -AAAGUGCAUC 6nt

**miR-259: cel-miR-259 ^(5’)^, hsa-miR-216a, hsa-miR-216b**

1 10

cel-miR-259 AAAUCUCAUC

hsa-miR-216a UAAUCUCAGC 8nt

hsa-miR-216b AAAUCUCUGC 8nt

**miR-266: cel-miR-266, hsa-miR-31**

1 10

cel-miR-266 AGGCAAGACU

hsa-miR-31 AGGCAAGAUG 8nt

**miR-267: cel-miR-267 ^(5’)^, <hsa-miR-99a, <hsa-miR-99b,**

**<hsa-miR-100**

1

cel-miR-267 --CCCGUGAAGU

hsa-miR-99a AACCCGUAGA-- 6nt

hsa-miR-99b CACCCGUAGA-- 6nt

hsa-miR-100 AACCCGUAGA-- 6nt

**miR-268: cel-miR-268 ^(5’)^, hsa-miR-31, <hsa-miR-873**

1

cel-miR-268 -GGCAAGAAUU-

hsa-miR-31 AGGCAAGAUG-- 7nt

hsa-miR-873 --GCAGGAACUU 7nt

**miR-269: cel-miR-269, hsa-miR-31**

1

cel-miR-269 -GGCAAGACU

hsa-miR-31 AGGCAAGAUG 7nt

**miR-273: cel-miR-273 ^(5’)^, <hsa-miR-99a, <hsa-miR-99b,**

**<hsa-miR-100**

1 10

cel-miR-273 UGCCCGUACU

hsa-miR-99a AACCCGUAGA 6nt

hsa-miR-99b CACCCGUAGA 6nt

hsa-miR-100 AACCCGUAGA 6nt

**miR-357: cel-miR-357 ^(5’)^, <hsa-miR-302a, <hsa-miR-302b,**

**<hsa-miR-302c, <hsa-miR-302d**

1 10

cel-miR-357 UAAAUGCCAG

hsa-miR-302a UAAGUGCUUC 6nt

hsa-miR-302b UAAGUGCUUC 6nt

hsa-miR-302c UAAGUGCUUC 6nt

hsa-miR-302d UAAGUGCUUC 6nt

**miR-785: cel-miR-785 ^(5’)^, <hsa-miR-545*, <hsa-miR-559**

1

cel-miR-785 -UAAGUGAAUU

hsa-miR-545* -UCAGUAAAUG 7nt

hsa-miR-559 UAAAGUAAAU- 7nt

**miR-786: cel-miR-786 ^(5’)^, <hsa-miR-18a*, <hsa-miR-18b*,**

**hsa-miR-365**

1

cel-miR-786 UAAUGCCCUG---

hsa-miR-18a* -ACUGCCCUAA-- 7nt

hsa-miR-18b* ---UGCCCUAAAU 6nt

hsa-miR-365 UAAUGCCCCU--- 8nt

**miR-787: cel-miR-787 ^(5’)^, hsa-miR-99a*, hsa-miR-99b*,**

**<hsa-miR-556-5p**

1 12

cel-miR-787 --UAAGCUCGUU

hsa-miR-99b* --CAAGCUCGUG 8nt

hsa-miR-99a* --CAAGCUCGCU 8nt

hsa-miR-556-5p GAUGAGCUCA-- 6nt

**miR-790: cel-miR-790 ^(5’)^, hsa-miR-96, hsa-miR-183,**

**<hsa-miR-200a, <hsa-miR-514**

1

cel-miR-790 CUUGGCACUC--

hsa-miR-183 UAUGGCACUG-- 7nt

hsa-miR-200a --UAACACUGUC 5nt

hsa-miR-514 AUUGACACUU-- 7nt

hsa-miR-96 UUUGGCACUA-- 8nt

**miR-791: cel-miR-791 ^(5’)^, hsa-miR-96, hsa-miR-182,**

**hsa-miR-183, <hsa-miR-200a, <hsa-miR-514**

1

cel-miR-791 UUUGGCACUC--

hsa-miR-96 UUUGGCACUA-- 9nt

hsa-miR-182 UUUGGCAAUG-- 8nt

hsa-miR-183 UAUGGCACUG-- 8nt

hsa-miR-200a --UAACACUGUC 5nt

hsa-miR-514 AUUGACACUU-- 7nt

**miR-793: cel-miR-793 ^(5’)^, hsa-let-7a, hsa-let-7b, hsa-let-7c,**

**hsa-let-7e, hsa-let-7f, hsa-let-7g, hsa-let-7i,**

**hsa-miR-98, hsa-miR-202**

1 10

cel-miR-793 UGAGGUAUCU

hsa-let-7a UGAGGUAGUA 7nt

hsa-let-7b UGAGGUAGUA 7nt

hsa-let-7c UGAGGUAGUA 7nt

hsa-let-7e UGAGGUAGGA 7nt

hsa-let-7f UGAGGUAGUA 7nt

hsa-let-7g UGAGGUAGUA 7nt

hsa-let-7i UGAGGUAGUA 7nt

hsa-miR-98 UGAGGUAGUA 7nt

hsa-miR-202 AGAGGUAUAG 7nt

**miR-794: cel-miR-794 ^(5’)^, hsa-let-7a, hsa-let-7b, hsa-let-7c,**

**hsa-let-7d, hsa-let-7e, hsa-let-7f, hsa-let-7g,**

**hsa-let-7i, hsa-miR-98, <hsa-miR-196a**

1

cel-miR-794 UGAGGUAAUC-

hsa-let-7d AGAGGUAGUA- 7nt

hsa-miR-196a -UAGGUAGUUU 6nt

hsa-let-7a UGAGGUAGUA- 8nt

hsa-let-7b UGAGGUAGUA- 8nt

hsa-let-7c UGAGGUAGUA- 8nt

hsa-let-7f UGAGGUAGUA- 8nt

hsa-let-7g UGAGGUAGUA- 8nt

hsa-let-7i UGAGGUAGUA- 8nt

hsa-let-7e UGAGGUAGGA- 7nt

hsa-miR-98 UGAGGUAGUA- 8nt

**miR-795: cel-miR-795 ^(5’)^, hsa-let-7a, hsa-let-7b, hsa-let-7c,**

**hsa-let-7d, hsa-let-7e, hsa-let-7f, hsa-let-7g,**

**hsa-let-7i, hsa-miR-98**

1 10

cel-miR-795 UGAGGUAGAU

hsa-let-7a UGAGGUAGUA 8nt

hsa-let-7b UGAGGUAGUA 8nt

hsa-let-7c UGAGGUAGUA 8nt

hsa-let-7d AGAGGUAGUA 7nt

hsa-let-7e UGAGGUAGGA 8nt

hsa-let-7f UGAGGUAGUA 8nt

hsa-let-7g UGAGGUAGUA 8nt

hsa-let-7i UGAGGUAGUA 8nt

hsa-miR-98 UGAGGUAGUA 8nt

**miR-796: cel-miR-796 ^(5’)^, hsa-miR-1, <hsa-miR-122,**

**hsa-miR-206**

1 10

cel-miR-796 UGGAAUGUAG

hsa-miR-122 UGGAGUGUGA 7nt

hsa-miR-1 UGGAAUGUAA 9nt

hsa-miR-206 UGGAAUGUAA 9nt

**miR-797: cel-miR-797 ^(5’)^, hsa-miR-499-3p**

1

cel-miR-797 --UAUCACAGCA

hsa-miR-499-3p AACAUCACAG-- 7nt

**miR-1018: cel-miR-1018 ^(5’)^, <hsa-miR-450b-3p**

1

cel-miR-1018 AGAGAGAUCA-

hsa-miR-450b-3p -UUGGGAUCAU 6nt

**miR-1020: cel-miR-1020 ^(5’)^, hsa-miR-148b***

1

cel-miR-1020 AUUAUUCUGU-

hsa-miR-148b* -AAGUUCUGUU 6nt

**miR-1022: cel-miR-1022 ^(5’)^, <hsa-miR-450b-3p**

1

cel-miR-1022 --AAGAUCAUUG

hsa-miR-450b-3p UUGGGAUCAU-- 6nt
