# Supplementary material for: Sequence Relationships among C. elegans, D. melanogaster and Human microRNAs Highlight the Extensive Conservation of microRNAs in Biology
Source: PLoS One. 2008 Jul 30;3(7):e2818. doi: 10.1371/journal.pone.0002818 (PMC2486268; doi:10.1371/journal.pone.0002818)
Supplement: Dataset S11 — Sequence identity table and alignments of C. elegans-H. sapiens miRNAs with ≥70% overall homology. (0.12 MB DOC) [file pone.0002818.s015.doc]

**Supplementary Table S11: 22 *C. elegans* miRNAs (grouped in 22 families) are ≥70% identical to 46 human miRNAs in 57 sequence relationships (alignments below).**

|  | **Sequence Related miRNAs** | |  |
| --- | --- | --- | --- |
| **miRNA Group ID** | ***C. elegan*s** | ***H. sapiens*** | **Overall Identity (≥70%)** |
| **let-7** | cel-let-7 | hsa-let-7a | 100.0 |
|  |  | hsa-let-7c | 95.5 |
|  |  | hsa-let-7e | 95.5 |
|  |  | hsa-let-7f | 95.5 |
|  |  | hsa-let-7b | 90.9 |
|  |  | hsa-let-7d | 90.9 |
|  |  | hsa-let-7g | 90.9 |
|  |  | hsa-miR-98 | 90.9 |
|  |  | hsa-let-7i | 81.8 |
| **lin-4** | cel-lin-4 | hsa-miR-125b | 81.8 |
|  |  | hsa-miR-125a-5p | 75.0 |
| **miR-1** | cel-miR-1 | hsa-miR-1 | 95.5 |
|  |  | hsa-miR-206 | 81.8 |
| **miR-34** | cel-miR-34 | hsa-miR-34c-5p | 87.0 |
|  |  | hsa-miR-34a | 82.6 |
|  |  | hsa-miR-34b* | 82.6 |
|  |  | hsa-miR-449a | 75.0 |
|  |  | hsa-miR-449b | 75.0 |
| **miR-50** | cel-miR-50 | hsa-miR-190 | 72.7 |
|  |  | hsa-miR-190b | 70.8 |
| **miR-51** | cel-miR-51 | hsa-miR-99a | 73.9 |
| **miR-57** | cel-miR-57 | hsa-miR-10a | 75.0 |
|  |  | hsa-miR-99a | 72.0 |
|  |  | hsa-miR-100 | 72.0 |
|  |  | hsa-miR-10b | 70.8 |
| **miR-72** | cel-miR-72 | hsa-miR-31 | 87.0 |
| **miR-79** | cel-miR-79 | hsa-miR-9* | 77.3 |
| **miR-83** | cel-miR-83 | hsa-miR-29a | 77.3 |
|  |  | hsa-miR-29c | 77.3 |
|  |  | hsa-miR-29b | 73.9 |
| **miR-84** | cel-miR-84 | hsa-miR-98 | 81.8 |
|  |  | hsa-let-7a | 77.3 |
|  |  | hsa-let-7c | 77.3 |
|  |  | hsa-let-7b | 72.7 |
|  |  | hsa-let-7e | 72.7 |
|  |  | hsa-let-7f | 72.7 |
| **miR-124** | cel-miR-124 | hsa-miR-124 | 95.2 |
| **miR-228** | cel-miR-228 | hsa-miR-183 | 73.9 |
| **miR-234** | cel-miR-234 | hsa-miR-137 | 73.9 |
| **miR-235** | cel-miR-235 | hsa-miR-92a | 81.8 |
|  |  | hsa-miR-92b | 81.8 |
|  |  | hsa-miR-25 | 72.7 |
| **miR-236** | cel-miR-236 | hsa-miR-200b | 78.3 |
|  |  | hsa-miR-200c | 78.3 |
|  |  | hsa-miR-141 | 73.9 |
|  |  | hsa-miR-200a | 73.9 |
|  |  | hsa-miR-429 | 73.9 |
| **miR-240** | cel-miR-240 | hsa-miR-193b | 72.7 |
| **miR-245** | cel-miR-245 | hsa-miR-133a | 77.3 |
|  |  | hsa-miR-133b | 77.3 |
| **miR-256** | cel-miR-256 | hsa-miR-1 | 77.3 |
| **miR-266** | cel-miR-266 | hsa-miR-31 | 76.2 |
|  |  | hsa-miR-301a | 73.9 |
|  |  | hsa-miR-301b | 73.9 |
|  |  | hsa-miR-25* | 71.4 |
| **miR-269** | cel-miR-269 | hsa-miR-31 | 76.2 |
| **miR-793** | cel-miR-793 | hsa-let-7g | 70.8 |

**Supplementary Alignments S11:**

**Sequence alignments of *C. elegans_H. sapiens* miRNAs with ≥70% overall sequence identity.** Members of a family are ≥70% identical to at least one other miRNA member. Identity to *C. elegans* miRNAs is given in percentage at the end of each human homolog sequence. Shaded in grey indicate potential G..U pairing.

**let-7: cel-let-7, hsa-let-7a, hsa-let-7b, hsa-let-7c, hsa-let-7d,**

**hsa-let-7e, hsa-let-7f, hsa-let-7g, hsa-let-7i, hsa-miR-98**

1 22

cel-let-7 UGAGGUAGUAGGUUGUAUAGUU

hsa-let-7a UGAGGUAGUAGGUUGUAUAGUU 100.0%

hsa-let-7b UGAGGUAGUAGGUUGUGUGGUU 90.9%

hsa-let-7c UGAGGUAGUAGGUUGUAUGGUU 95.5%

hsa-miR-98 UGAGGUAGUAAGUUGUAUUGUU 90.9%

hsa-let-7f UGAGGUAGUAGAUUGUAUAGUU 95.5%

hsa-let-7g UGAGGUAGUAGUUUGUACAGUU 90.9%

hsa-let-7i UGAGGUAGUAGUUUGUGCUGUU 81.8%

hsa-let-7d AGAGGUAGUAGGUUGCAUAGUU 90.9%

hsa-let-7e UGAGGUAGGAGGUUGUAUAGUU 95.5%

**lin-4: cel-lin-4, hsa-miR-125a-5p, hsa-miR-125b**

1 24

cel-lin-4 UCCCUGAGACC---UCAAGUGUGA

hsa-miR-125a-5p UCCCUGAGACCCUUUAACCUGUGA 75.0%

hsa-miR-125b UCCCUGAGACCC--UAACUUGUGA 81.8%

**miR-1: cel-miR-1, hsa-miR-1, hsa-miR-206**

1 22

cel-miR-1 UGGAAUGUAAAGAAGUAUGUA-

hsa-miR-1 UGGAAUGUAAAGAAGUAUGUAU 95.5%

hsa-miR-206 UGGAAUGUAAGGAAGUGUGUGG 81.8%

**miR-34: cel-miR-34, hsa-miR-34a, hsa-miR-34b*,**

**hsa-miR-34c-5p, hsa-miR-449a, hsa-miR-449b**

1 26

cel-miR-34 -AGGCAGUGUGG--UUAGCUGGUUG-

hsa-miR-34a -UGGCAGUGUC---UUAGCUGGUUGU 82.6%

hsa-miR-449a -UGGCAGUGUAUUGUUAGCUGGU--- 75.0%

hsa-miR-449b -AGGCAGUGUAUUGUUAGCUGGC--- 75.0%

hsa-miR-34b* UAGGCAGUGUCA--UUAGCUGAUUG- 82.6%

hsa-miR-34c-5p -AGGCAGUGUAG--UUAGCUGAUUGC 87.0%

**miR-50: cel-miR-50, hsa-miR-190, hsa-miR-190b**

1 23

cel-miR-50 UGAUAUGUCUGGUAUUCUUGGG-

hsa-miR-190 UGAUAUGUUUGAUAUAUUAGGU- 72.7%

hsa-miR-190b UGAUAUGUUUGAUAU--UGGGUU 70.8%

**miR-51: cel-miR-51, hsa-miR-99a**

1 23

cel-miR-51 UACCCGUAGCUCCUAUCCAUGUU

hsa-miR-99a AACCCGUAGAUCCGAUCU-UGUG 73.9%

**miR-57: cel-miR-57, hsa-miR-10a, hsa-miR-10b, hsa-miR-99a, hsa-miR-100**

1 24

cel-miR-57 UACCCUGUAGAUCGAGCUGUGUGU

hsa-miR-10a UACCCUGUAGAUCCGAAUUUGUG- 75.0%

hsa-miR-99a -AACCCGUAGAUCCGAUCUUGUG- 72.0%

hsa-miR-100 -AACCCGUAGAUCCGAACUUGUG- 72.0%

hsa-miR-10b UACCCUGUAGAACCGAAUUUGUG- 70.8%

**miR-72: cel-miR-72, hsa-miR-31**

1 23

cel-miR-72 AGGCAAGAUGUUGGCAUAGCUGA

hsa-miR-31 AGGCAAGAUGCUGGCAUAGCU-- 87.0%

**miR-79: cel-miR-79, hsa-miR-9***

1 22

cel-miR-79 AUAAAGCUAGGUUACCAAAGCU

hsa-miR-9* AUAAAGCUAGAUAACCGAAAGU 77.3%

**miR-83: cel-miR-83, hsa-miR-29a, hsa-miR-29b, hsa-miR-29c**

1 23

cel-miR-83 UAGCACCAUAUAAAUUCAGUAA-

hsa-miR-29b UAGCACCAUUUGAAAUCAGUGUU 73.9%

hsa-miR-29a UAGCACCAUCUGAAAUCGGUUA- 77.3%

hsa-miR-29c UAGCACCAUUUGAAAUCGGUUA- 77.3%

**cel-miR-84: cel-miR-84, hsa-let-7a, hsa-let-7b, hsa-let-7c,**

**hsa-let-7e, hsa-let-7f, hsa-miR-98**

1 22

cel-miR-84 UGAGGUAGUAUGUAAUAUUGUA

hsa-miR-98 UGAGGUAGUAAGUUGUAUUGUU 81.8%

hsa-let-7a UGAGGUAGUAGGUUGUAUAGUU 77.3%

hsa-let-7c UGAGGUAGUAGGUUGUAUGGUU 77.3%

hsa-let-7b UGAGGUAGUAGGUUGUGUGGUU 72.7%

hsa-let-7e UGAGGUAGGAGGUUGUAUAGUU 72.7%

hsa-let-7f UGAGGUAGUAGAUUGUAUAGUU 72.7%

**cel-miR-124: cel-miR-124, hsa-miR-124**

1 21

cel-miR-124 UAAGGCACGCGGUGAAUGCCA

hsa-miR-124 UAAGGCACGCGGUGAAUGCC- 95.2%

**cel-miR-228: cel-miR-228, hsa-miR-183**

1 23

cel-miR-228 AAUGGCACUGCAUGAAUUCACGG

hsa-miR-183 UAUGGCACUGGUAGAAUUCACU- 73.9%

**cel-miR-234: cel-miR-234, hsa-miR-137**

1 23

cel-miR-234 UUAUUGCUCGAGAAUACCCUU--

hsa-miR-137 UUAUUGCUUAAGAAUACGCGUAG 73.9%

**cel-miR-235: cel-miR-235, hsa-miR-25, hsa-miR-92a,**

**hsa-miR-92b**

1 22

cel-miR-235 UAUUGCACUCUCCCCGGCCUGA

hsa-miR-25 CAUUGCACUUGUCUCGGUCUGA 72.7%

hsa-miR-92a UAUUGCACUUGUCCCGGCCUGU 81.8%

hsa-miR-92b UAUUGCACUCGUCCCGGCCUCC 81.8%

**cel-miR-236: cel-miR-236, hsa-miR-141, hsa-miR-220a,**

**hsa-miR-200b, hsa-miR-200c, hsa-miR-429**

1 23

cel-miR-236 UAAUACUGUCAGGUAAUGACGCU

hsa-miR-141 UAACACUGUCUGGUAAAGAUGG- 73.9%

hsa-miR-200a UAACACUGUCUGGUAACGAUGU- 73.9%

hsa-miR-200b UAAUACUGCCUGGUAAUGAUGA- 78.3%

hsa-miR-200c UAAUACUGCCGGGUAAUGAUGGA 78.3%

hsa-miR-429 UAAUACUGUCUGGUAAAACCGU- 73.9%

**cel-miR-240: cel-miR-240, hsa-miR-193b**

1 22

cel-miR-240 UACUGGCCCCCAAAUCUUCGCU

hsa-miR-193b AACUGGCCCUCAAAGUCCCGCU 72.7%

**cel-miR-245: cel-miR-245, hsa-miR-133a, hsa-miR-133b**

1 22

cel-miR-245 AUUGGUCCCCUCCAAGUAGCUC

hsa-miR-133a UUUGGUCCCCUUCAACCAGCUG 77.3%

hsa-miR-133b UUUGGUCCCCUUCAACCAGCUA 77.3%

**cel-miR-256: cel-miR-256, hsa-miR-1**

1 22

cel-miR-256 UGGAAUGCAUAGAAGACUGUA-

hsa-miR-1 UGGAAUGUAAAGAAGUAUGUAU 77.3%

**cel-miR-266: cel-miR-266, hsa-miR-25*, hsa-miR-31,**

**hsa-miR-301a, hsa-miR-301b**

1 22

cel-miR-266 AGGCA-AGACUUUGGCAAAGC-

hsa-miR-25* AGGCGGAGACUUGGGCAAUUG- 71.4%

hsa-miR-31 AGGCA-AGAUGCUGGCAUAGCU 76.2%

1 23

cel-miR-266 -AG-GCAAGACU-UUGGCAAAGC

hsa-miR-301a CAGUGCAAUAGUAUUGUCAAAGC 73.9%

hsa-miR-301b CAGUGCAAUGAUAUUGUCAAAGC 73.9%

**cel-miR-269: cel-miR-269, hsa-miR-31**

1 21

cel-miR-269 -GGCAAGACUCUGGCAAAACU

hsa-miR-31 AGGCAAGAUGCUGGCAUAGCU 76.2%

**cel-miR-793: cel-miR-793, hsa-let-7g**

1 24

cel-miR-793 UGAGGUAUCUUAGUUAG-ACAGA-

hsa-let-7g UGAGGUAG--UAGUUUGUACAGUU 70.8%
