# Supplementary material for: Sequence Relationships among C. elegans, D. melanogaster and Human microRNAs Highlight the Extensive Conservation of microRNAs in Biology
Source: PLoS One. 2008 Jul 30;3(7):e2818. doi: 10.1371/journal.pone.0002818 (PMC2486268; doi:10.1371/journal.pone.0002818)
Supplement: Dataset S12 — Similarity table and sequence alignments of C. elegans-H. sapiens miRNAs with 60–69.9% overall identity. (0.24 MB DOC) [file pone.0002818.s016.doc]

**Supplementary Table S12: 67 *C. elegans* miRNAs show 60-69.9% overall similarity to 87 human miRNAs in 108 sequence relationships.** 55 of these *C. elegans* miRNAs are not related in sequence to other human miRNAs above the ≥70% threshold (Dataset S11). 26 of the 108 sequence relationships are ≥7nt homologous at the 5’ 10nt (Dataset S10, sequence alignments below).

|  | | Distantly Related miRNAs | |  |  |
| --- | --- | --- | --- | --- | --- |
| **miRNA**  **Group ID** | ***C. elegans*** | | ***H. sapiens*** | **Overall Identity**  **(60-69.9%)** | |
| **let-7** | cel-let-7 | | hsa-miR-625 | 60.9 | |
| **lin-4** | cel-lin-4 | | hsa-miR-362-5p | 64.0 | |
|  |  |  | hsa-miR-10a | 62.5 | |
| **lsy-6** | cel-lsy-6 | | hsa-miR-628-3p | 63.6 | |
| **miR-1** | cel-miR-1 | | hsa-miR-452* | 63.6 | |
| **miR-2** | cel-miR-2 | | hsa-miR-27b | 60.9 | |
| **miR-34** | cel-miR-34 | | hsa-miR-122 | 60.9 | |
| **miR-35** | cel-miR-35 | | hsa-miR-412 | 64.0 | |
| **miR-37** | cel-miR-37 | | hsa-miR-412 | 64.0 | |
| **miR-43** | cel-miR-43 | | hsa-miR-130a* | 66.7 | |
|  |  |  | hsa-miR-214* | 60.9 | |
| **miR-44** | cel-miR-44 | | hsa-miR-30b | 63.6 | |
|  |  |  | hsa-miR-380* | 60.0 | |
| **miR-45** | cel-miR-45 | | hsa-miR-30b | 63.6 | |
|  |  |  | hsa-miR-380* | 60.0 | |
| **miR-46** | cel-miR-46 | | hsa-miR-647 | 60.9 | |
|  |  |  | hsa-miR-933 | 60.0 | |
| **miR-47** | cel-miR-47 | | hsa-miR-485-3p | 62.5 | |
|  |  |  | hsa-miR-493* | 62.5 | |
| **miR-48** | cel-miR-48 | | hsa-let-7c | 62.5 | |
|  |  |  | hsa-miR-196a | 62.5 | |
| **miR-49** | cel-miR-49 | | hsa-miR-671-5p | 60.0 | |
| **miR-51** | cel-miR-51 | | hsa-miR-100 | 69.6 | |
|  |  |  | hsa-miR-10a | 66.7 | |
|  |  |  | hsa-miR-99b | 65.2 | |
|  |  |  | hsa-miR-10b | 62.5 | |
| **miR-52** | cel-miR-52 | | hsa-miR-99b | 62.5 | |
| **miR-53** | cel-miR-53 | | hsa-miR-99b | 62.5 | |
| **miR-56*** | cel-miR-56* | | hsa-miR-450b-3p | 66.7 | |
| **miR-60** | cel-miR-60 | | hsa-miR-544 | 66.7 | |
|  |  |  | hsa-miR-19b-2* | 65.2 | |
|  |  |  | hsa-miR-507 | 65.2 | |
|  |  |  | hsa-miR-19a* | 60.9 | |
| **miR-61** | cel-miR-61 | | hsa-miR-625* | 60.9 | |
| **miR-72** | cel-miR-72 | | hsa-miR-301b | 65.4 | |
|  |  |  | hsa-miR-301a | 61.5 | |
|  |  |  | hsa-miR-593* | 60.0 | |
|  |  |  | hsa-miR-608 | 60.0 | |
| **miR-73** | cel-miR-73 | | hsa-miR-185 | 62.5 | |
|  |  |  | hsa-miR-31 | 60.9 | |
|  |  |  | hsa-miR-593* | 60.0 | |
|  |  |  | hsa-miR-608 | 60.0 | |
| **miR-74** | cel-miR-74 | | hsa-miR-185 | 66.7 | |
|  |  |  | hsa-miR-873 | 60.9 | |
|  |  |  | hsa-miR-7-2* | 60.0 | |
| **miR-77** | cel-miR-77 | | hsa-miR-412 | 61.5 | |
| **miR-78** | cel-miR-78 | | hsa-miR-30b* | 62.5 | |
| **miR-79** | cel-miR-79 | | hsa-miR-142-5p | 60.9 | |
| **miR-80** | cel-miR-80 | | hsa-miR-450b-3p | 62.5 | |
| **miR-81** | cel-miR-81 | | hsa-miR-155 | 62.5 | |
| **miR-82** | cel-miR-82 | | hsa-miR-155 | 62.5 | |
|  |  |  | hsa-miR-450b-3p | 60.9 | |
| **miR-83** | cel-miR-83 | | hsa-miR-15a | 60.9 | |
| **miR-84** | cel-miR-84 | | hsa-let-7d | 68.2 | |
|  |  |  | hsa-let-7g | 68.2 | |
|  |  |  | hsa-let-7i | 68.2 | |
| **miR-86** | cel-miR-86 | | hsa-miR-301b | 64.0 | |
| **miR-87** | cel-miR-87 | | hsa-miR-545 | 69.6 | |
|  |  |  | hsa-miR-361-5p | 60.9 | |
| **miR-230** | cel-miR-230 | | hsa-miR-662 | 62.5 | |
|  |  |  | hsa-miR-575 | 60.9 | |
| **miR-231** | cel-miR-231 | | hsa-miR-100* | 62.5 | |
| **miR-232** | cel-miR-232 | | hsa-miR-367 | 65.2 | |
|  |  |  | hsa-miR-29c | 60.9 | |
|  |  |  | hsa-miR-302a | 60.9 | |
|  |  |  | hsa-miR-155 | 60.0 | |
| **miR-233** | cel-miR-233 | | hsa-miR-143* | 60.9 | |
| **miR-234** | cel-miR-234 | | hsa-miR-145* | 60.9 | |
| **miR-237** | cel-miR-237 | | hsa-miR-125a-5p | 60.0 | |
|  |  |  | hsa-miR-125b | 60.0 | |
| **miR-239b** | cel-miR-239b | | hsa-miR-153 | 60.9 | |
| **miR-247** | cel-miR-247 | | hsa-miR-625* | 60.9 | |
| **miR-250** | cel-miR-250 | | hsa-miR-125b-2* | 66.7 | |
| **miR-256** | cel-miR-256 | | hsa-miR-206 | 68.2 | |
| **miR-257** | cel-miR-257 | | hsa-miR-889 | 60.9 | |
| **miR-262** | cel-miR-262 | | hsa-miR-450a | 65.2 | |
| **miR-265** | cel-miR-265 | | hsa-miR-483-5p | 63.6 | |
| **miR-273** | cel-miR-273 | | hsa-miR-585 | 65.0 | |
| **miR-353** | cel-miR-353 | | hsa-miR-32* | 69.6 | |
|  |  |  | hsa-miR-222* | 60.9 | |
|  |  |  | hsa-miR-450a | 60.9 | |
| **miR-354** | cel-miR-354 | | hsa-miR-1238 | 60.9 | |
| **miR-357** | cel-miR-357 | | hsa-miR-155 | 69.6 | |
|  |  |  | hsa-miR-21* | 60.9 | |
| **miR-358** | cel-miR-358 | | hsa-miR-598 | 60.9 | |
| **miR-359** | cel-miR-359 | | hsa-miR-220c | 60.9 | |
|  |  |  | hsa-miR-330-5p | 60.9 | |
| **miR-360** | cel-miR-360 | | hsa-miR-943 | 60.9 | |
| **miR-392** | cel-miR-392 | | hsa-miR-101* | 60.0 | |
| **miR-784** | cel-miR-784 | | hsa-miR-379 | 68.2 | |
| **miR-785** | cel-miR-785 | | hsa-miR-29a* | 62.5 | |
| **miR-786** | cel-miR-786 | | hsa-miR-365 | 60.9 | |
| **miR-787** | cel-miR-787 | | hsa-miR-374b* | 62.5 | |
| **miR-788** | cel-miR-788 | | hsa-miR-877* | 60.0 | |
| **miR-789** | cel-miR-789 | | hsa-miR-615-3p | 65.2 | |
| **miR-790** | cel-miR-790 | | hsa-miR-218-2* | 68.2 | |
|  |  |  | hsa-miR-182 | 62.5 | |
| **miR-791** | cel-miR-791 | | hsa-miR-96 | 66.7 | |
| **miR-793** | cel-miR-793 | | hsa-let-7i | 62.5 | |
|  |  |  | hsa-let-7a | 60.9 | |
|  |  |  | hsa-miR-98 | 60.9 | |
|  |  |  | hsa-miR-545* | 60.9 | |
| **miR-794** | cel-miR-794 | | hsa-let-7g | 66.7 | |
| **miR-795** | cel-miR-795 | | hsa-let-7b | 62.5 | |
|  |  |  | hsa-let-7f | 62.5 | |
| **miR-796** | cel-miR-796 | | hsa-miR-1 | 69.6 | |
|  |  |  | hsa-miR-206 | 65.2 | |
|  |  |  | hsa-miR-26b | 60.9 | |

**Supplementary Alignments S12:**

**Aligned sequences of *C. elegans_H. sapiens* miRNAs with 60-69.9% full sequence similarity.** Identity to *C. elegans* miRNAs is given in percentage at the end of each *H. sapiens* homolog sequence. Grey shading indicates potential G..U pairing.

**let-7: cel-let-7, hsa-miR-625**

1 23

cel-let-7 UGAGGUAGUAGGUUGUAUAGUU-

hsa-miR-625 --AGGGGGAAAGUUCUAUAGUCC 60.9%

**lin-4: cel-lin-4, hsa-miR-10a, hsa-miR-362-5p**

1 25

cel-lin-4 --UCCCUGAGACCUCAAGUGUGA--

hsa-miR-362-5p AAUCCUUGGAACCU-AGGUGUGAGU 64.0%

1 24

cel-lin-4 -UCCCUG-AGACCUCAAGU-GUGA

hsa-miR-10a UACCCUGUAGAUCCGAAUUUGUG- 62.5%

**lys-6: cel-lys-6, hsa-miR-628-3p**

1 22

cel-lsy-6 UUUUGUAUGAGACGCAUUUCG-

hsa-miR-628-3p UCUAGUAAGAGUGGCAGU-CGA 63.6%

**miR-1: cel-miR-1, hsa-miR-452***

1 22

cel-miR-1 -UGGAAUGUAAAGAAGUAUGUA

hsa-miR-452* CUCAUCUGCAAAGAAGUAAGUG 63.6%

**miR-2: cel-miR-2, hsa-miR-27b**

1 23

cel-miR-2 UAUCACAGCCAGCUUUGAUGUGC

hsa-miR-27b -UUCACAGUG-GCUAAGUUCUGC 60.9%

**miR-34: cel-miR-34, hsa-miR-122**

1 23

cel-miR-34 AGGCAGUGUGGUUA-GCUGGUUG

hsa-miR-122 -UGGAGUGUGACAAUGGUGUUUG 60.9%

**miR-35: cel-miR-35, hsa-miR-412**

1 25

cel-miR-35 ---UCACCGGGUGGAAACUAGCAGU

hsa-miR-412 ACUUCACCUGGUCC--ACUAGCCGU 64.0%

**miR-37: cel-miR-37, hsa-miR-412**

1 25

cel-miR-37 ---UCACCGGGUGAACACUUGCAGU

hsa-miR-412 ACUUCACCUGGU--CCACUAGCCGU 64.0%

**miR-43: cel-miR-43, hsa-miR-130a*, hsa-miR-214***

1 24

cel-miR-43 UAUCACAGUUUACUUGCUGUC-GC

hsa-miR-130a* -UUCACAUUGUGCUA-CUGUCUGC 66.7%

1 23

cel-miR-43 UAUCACAGUUUACUUGCUGUCGC

hsa-miR-214* UGCCUGUCUACACUUGCUGU-GC 60.9%

**miR-44: cel-miR-44, hsa-miR-30b, hsa-miR-380***

1 22

cel-miR-44 UGACUAGAGAC-ACAUUCAGCU

hsa-miR-30b UGUAAACAUCCUACACUCAGCU 63.6%

1 25

cel-miR-44 ----UGACUAGAGACACAUUCAGCU

hsa-miR-380* UGGUUGACCAUAGA-ACAUGC-GC- 60.0%

**miR-45: cel-miR-45, hsa-miR-30b, hsa-miR-380***

1 22

cel-miR-45 UGACUAGAGAC-ACAUUCAGCU

hsa-miR-30b UGUAAACAUCCUACACUCAGCU 63.6%

1 25

cel-miR-45 ----UGACUAGAGACACAUUCAGCU

hsa-miR-380* UGGUUGACCAUAGA-ACAUGC-GC- 60.0%

**miR-46: cel-miR-46, hsa-miR-647, hsa-miR-933**

1 23

cel-miR-46 -UGUCAUGGAGUCGCUCUCUUCA

hsa-miR-647 GUGGC-UGCACUCACUUCCUUC- 60.9%

1 25

cel-miR-46 UGU---CAUGGAGUCGCUCUCUUCA

hsa-miR-933 UGUGCGCAGGGAGAC-CUCUCCC-- 60.0%

**miR-47: cel-miR-47, hsa-miR-485-3p, hsa-miR-493***

1 24

cel-miR-47 UGUCAUGGAGGCGCUCUCUUCA--

hsa-miR-485-3p -GUCAUACACG-GCUCUCCUCUCU 62.5%

1 24

cel-miR-47 -UGU-CAUGGAGGCGCUCUCUUCA

hsa-miR-493* UUGUACAUGGUAG-GCUUUCAUU- 62.5%

**miR-48: cel-miR-48, hsa-let-7c, hsa-miR-196a**

1 24

cel-miR-48 UGAGGUAGGCUCAGUAG-AUGCGA

hsa-let-7c UGAGGUAG--UAGGUUGUAUGGUU 62.5%

1 24

cel-miR-48 UGAGGUAGGCUCA-GUAGAUGCGA

hsa-miR-196a -UAGGUAGUUUCAUGUUGUUGGG- 62.5%

**miR-49: cel-miR-49, hsa-miR-671-5p**

1 25

cel-miR-49 ---AAGCACCACGAGAAGCUGCAGA

hsa-miR-671-5p AGGAAGC-CCUGGAGGGGCUGGAG- 60.0%

**miR-51: cel-miR-51, hsa-miR-10a, hsa-miR-10b, hsa-miR-99b,**

**hsa-miR-100**

1 23

cel-miR-51 UACCCGUAGCUCCUAUCCAUGUU

hsa-miR-100 AACCCGUAGAUCCGAACU-UGUG 69.6%

1 24

cel-miR-51 UACCC-GUAGCUCCUAUCCAUGUU

hsa-miR-10a UACCCUGUAGAUCCGAAUU-UGUG 66.7%

1 23

cel-miR-51 UACCCGUAGCUCCUAUCCAUGUU

hsa-miR-99b CACCCGUAGAACCGA-CCUUGCG 65.2%

1 24

cel-miR-51 UACCC-GUAGCUCCUAUCCAUGUU

hsa-miR-10b UACCCUGUAGAACCGAAUU-UGUG 62.5%

**miR-52: cel-miR-52, hsa-miR-99b**

1 24

cel-miR-52 CACCCGUACAUAUGUUUCCGUGCU

hsa-miR-99b CACCCGUAGAACCGA-—CCUUGCG 62.5%

**miR-53: cel-miR-53, hsa-miR-99b**

1 24

cel-miR-53 CACCCGUACAUUUGUUUCCGUGCU

hsa-miR-99b CACCCGUAGAACCGA--CCUUGCG 62.5%

**miR-56*: cel-miR-56*, hsa-miR-450b-3p**

1 24

cel-miR-56* UGGCGGAUCCAUUUUGGGUUG-UA

hsa-miR-450b-3p UUG-GGAUC-AUUUUGCAUCCAUA 66.7%

**miR-60: cel-miR-60, hsa-miR-19a*, hsa-miR-19b-2*,**

**hsa-miR-507, hsa-miR-544**

1 24

cel-miR-60 UAUUAUGCACAUUUU-CUAGUUCA

hsa-miR-544 -AUUCUGCAUUUUUAGCAAGUUC- 66.7%

1 23

cel-miR-60 UAUUAUGCACAUUUUCUAGUUCA

hsa-miR-19b-2* AGUUUUGCAGGUUUGC-AUUUCA 65.2%

1 23

cel-miR-60 UAUUAUGCACAUUUUCUAGUUCA

hsa-miR-507 --UUUUGCACCUUUUGGAGUGAA 65.2%

1 23

cel-miR-60 UAUUAUGCACAUUUUCUAGUUCA

hsa-miR-19a* AGUUUUGCAUAGUUGC-ACUACA 60.9%

**miR-61: cel-miR-61, hsa-miR-625***

1 23

cel-miR-61 UGACUAGA-ACCGUUACUCAUC-

hsa-miR-625* -GACUAUAGAACUUUCCCCCUCA 60.9%

**miR-72: cel-miR-72, hsa-miR-301a, hsa-miR-301b,**

**hsa-miR-593*, hsa-miR-608**

1 26

cel-miR-72 -AG-GCAA-GAUGUUGGCAUAGCUGA

hsa-miR-301b CAGUGCAAUGAUAUUGUCAAAGC--- 65.4%

1 26

cel-miR-72 -AG-GCAAGA-UGUUGGCAUAGCUGA

hsa-miR-301a CAGUGCAAUAGUAUUGUCAAAGC--- 61.5%

1 25

cel-miR-72 AGGCAAGAUGUUGGCAUAGCUGA--

hsa-miR-593* AGGCACCAGCCAGGCAUUGCUCAGC 60.0%

1 25

cel-miR-72 AGGCAAGAUGUUGGCAUAGCUGA--

hsa-miR-608 AGGGGUGGUGUUGGGACAGCUCCGU 60.0%

**miR-73: cel-miR-73, hsa-miR-31, hsa-miR-185,**

**hsa-miR-593*, hsa-miR-608**

1 24

cel-miR-73 UGGCAAGAUGUAGGCAGUUCAGU-

hsa-miR-185 UGG--AGAGAAAGGCAGUUCCUGA 62.5%

1 23

cel-miR-73 UGGCAAGAUGUAGGCAGUUCAGU

hsa-miR-31 AGGCAAGAUGCUGGCAUAGCU-- 60.9%

1 25

cel-miR-73 UGGCAAGAUGUAGGCAGU--UCAGU

hsa-miR-593* AGGCACCAGCCAGGCAUUGCUCAGC 60.0%

1 25

cel-miR-73 UGGCAAGAUGUAGG--CAGUUCAGU

hsa-miR-608 AGGGGUGGUGUUGGGACAGCUCCGU 60.0%

**miR-74: cel-miR-74, hsa-miR-7-2*, hsa-miR-185, hsa-miR-873**

1 24

cel-miR-74 UGGCAAGAAAUGGCAGUCUACA--

hsa-miR-185 UGGAGAGAAA-GGCAGU-UCCUGA 66.7%

1 23

cel-miR-74 UGGCAAGAAAUGGC-AGUCUACA

hsa-miR-873 --GCAGGAACUUGUGAGUCUCCU 60.9%

1 25

cel-miR-74 UGGCAAGAAAUGGCAGUCUACA---

hsa-miR-7-2* ---CAACAAAUCCCAGUCUACCUAA 60.0%

**miR-77: cel-miR-77, hsa-miR-412**

1 26

cel-miR-77 --UUCAUCAGG-CCA-UAGCUGUCCA

hsa-miR-412 ACUUCACCUGGUCCACUAGCCGU--- 61.5%

**miR-78: cel-miR-78, hsa-miR-30b***

1 24

cel-miR-78 --UGGAGGCCUGGUUGUUUG-UGC

hsa-miR-30b* CUGGGAGG--UGGAUGUUUACUUC 62.5%

**miR-79: cel-miR-79, hsa-miR-142-5p**

1 23

cel-miR-79 -AUAAAGCUAGGUUACCAAAGCU

hsa-miR-142-5p CAUAAAG-UAGAA-AGCACUACU 60.9%

**miR-80: cel-miR-80, hsa-miR-450b-3p**

1 24

cel-miR-80 -UGAGAUCAUUAGUUGAAAGCCGA

hsa-miR-450b-3p UUGGGAUCAUU--UUGCAUCCAUA 62.5%

**miR-81: cel-miR-81, hsa-miR-155**

1 24

cel-miR-81 -UGA-GAUCAUCGUGAAAGCUAGU

hsa-miR-155 UUAAUGCUAAUCGUGAUAGGG-GU 62.5%

**miR-82: cel-miR-82, hsa-miR-155, hsa-miR-450b-3p**

1 24

cel-miR-82 -UGA-GAUCAUCGUGAAAGCCAGU

hsa-miR-155 UUAAUGCUAAUCGUGAUAGGG-GU 62.5%

1 23

cel-miR-82 -UGAGAUCAUCGUGAAAGCCAGU

hsa-miR-450b-3p UUGGGAUCAUUUUGCAU-CCAUA 60.9%

**miR-83: cel-miR-83, hsa-miR-15a**

1 23

cel-miR-83 UAGCACCAUAUAAAU-UCAGUAA

hsa-miR-15a UAGCAGCACAUAAUGGUUUGUG- 60.9%

**miR-84: cel-miR-84, hsa-let-7d, hsa-let-7g, hsa-let-7i**

1 22

cel-miR-84 UGAGGUAGUAUGUAAUAUUGUA

hsa-let-7d AGAGGUAGUAGGUUGCAUAGU- 68.2%

1 22

cel-miR-84 UGAGGUAGUAUGUAAUAUUGUA

hsa-let-7g UGAGGUAGUAGUUUGUACAGUU 68.2%

1 22

cel-miR-84 UGAGGUAGUAUGUAAUAUUGUA

hsa-let-7i UGAGGUAGUAGUUUGUGCUGUU 68.2%

**miR-86: cel-miR-86, hsa-miR-301b**

1 25

cel-miR-86 UAAGUG-AAUGCU-UUGCCACAGUC

hsa-miR-301b -CAGUGCAAUGAUAUUGUCAAAGC- 64.0%

**miR-87: cel-miR-87, hsa-miR-361-5p, hsa-miR-545**

1 23

cel-miR-87 GUGAGCAAAGUUUCAG-GUGUGC

hsa-miR-545 -UCAGCAAACAUUUAUUGUGUGC 69.6%

1 23

cel-miR-87 GUGAGCAAAGUUUC-AGGUGUGC

hsa-miR-361-5p -UUAUCAGAAUCUCCAGGGGUAC 60.9%

**miR-230: cel-miR-230, hsa-miR-575, hsa-miR-662**

1 24

cel-miR-230 GUAUUA-GUUGUGCGACCAGGAGA

hsa-miR-662 -UCCCACGUUGUG-GCCCAGCAG- 62.5%

1 23

cel-miR-230 GUAUUAGUUGUGCGACCAGGAGA

hsa-miR-575 GAGCCAGUUG---GAC-AGGAGC 60.9%

**miR-231: cel-miR-231, hsa-miR-100***

1 24

cel-miR-231 UAAGCUCGUGAUCAACAGGCAGAA

hsa-miR-100* CAAGCUUGU-AUCUAUAGGUAUG- 62.5%

**miR-232: cel-miR-232, hsa-miR-29c, hsa-miR-155,**

**hsa-miR-302a, hsa-miR-367**

1 23

cel-miR-232 UAAAUGCAUCUUAACUGCGGUGA

hsa-miR-367 -AAUUGCACUUUAGCAAUGGUGA 65.2%

1 23

cel-miR-232 UAAAUGCAUCUUAACUGCGGUGA

hsa-miR-29c UAGCACCAUUUGAAAU-CGGUUA 60.9%

1 23

cel-miR-232 UAAAUGCAUCUUAACUGCGGUGA

hsa-miR-302a UAAGUGCUUCCAUGUUUUGGUGA 60.9%

1 25

cel-miR-232 UAAAUGC--AUCUUAACUGCGGUGA

hsa-miR-155 UUAAUGCUAAUCGUGAUAGGGGU-- 60.0%

**miR-233: cel-miR-233, hsa-miR-143***

1 23

cel-miR-233 UUGAGCAAUGC-GCAUGUGCGG-

hsa-miR-143* -GGUGCAGUGCUGCAUCUCUGGU 60.9%

**miR-234: cel-miR-234, hsa-miR-145***

1 23

cel-miR-234 UUAUUGCUCGAGAAUACCCUU--

hsa-miR-145* GGAUUCCUGGA-AAUACUGUUCU 60.9%

**miR-237: cel-miR-237, hsa-miR-125a-5p, hsa-miR-125b**

1 25

cel-miR-237 UCCCUGAGAAUUCUCGAACAGCU--

hsa-miR-125a-5p UCCCUGAGACC-CUUUAACCUGUGA 60.0%

1 25

cel-miR-237 UCCCUGAGAAUUCUCGAACAGCU--

hsa-miR-125b UCCCUGAGA---CCCUAACUUGUGA 60.0%

**miR-239b: cel-miR-239b, hsa-miR-153**

1 23

cel-miR-239b UUUGUACUA-CACAAAAGUACUG

hsa-miR-153 -UUGCAUAGUCACAAAAGUGAUC 60.9%

**miR-247: cel-miR-247, hsa-miR-625***

1 23

cel-miR-247 UGACUAGAGC-CUAUUCUCUUCU

hsa-miR-625* -GACUAUAGAACUUUCCCCCUCA 60.9%

**miR-250: cel-miR-250, hsa-miR-125b-2***

1 24

cel-miR-250 AAUCACA-GUCAA-CUGUUGGCA-

hsa-miR-125b-2* --UCACAAGUCAGGCUCUUGGGAC 66.7%

**miR-256: cel-miR-256, hsa-miR-206**

1 22

cel-miR-256 UGGAAUGCAUAGAAGACUGUA-

hsa-miR-206 UGGAAUGUAAGGAAGUGUGUGG 68.2%

**miR-257: cel-miR-257, hsa-miR-889**

1 23

cel-miR-257 -GAGUAUCAGGAGUACCCAGUGA

hsa-miR-889 UUAAUAUC-GGACAACC-AUUGU 60.9%

**miR-262: cel-miR-262, hsa-miR-450a**

1 23

cel-miR-262 GUUUCUCGAUGUUUUC-UGAU--

hsa-miR-450a -UUUUGCGAUGUGUUCCUAAUAU 65.2%

**miR-265: cel-miR-265, hsa-miR-483-5p**

1 22

cel-miR-265 -UGA-GGGAGGAAGGGUGGUAU

hsa-miR-483-5p AAGACGGGAGGAAAGAAGGGAG 63.6%

**miR-273: cel-miR-273, hsa-miR-585**

1 20

cel-miR-273 UGCCCGUACUGUGUCGGCUG

hsa-miR-585 UGGGCGUA-UCUGUAUGCUA 65.0%

**miR-353: cel-miR-353, hsa-miR-32*, hsa-miR-222*,**

**hsa-miR-450a**

1 23

cel-miR-353 CAAUUGCCAUGUGU-UGGUAUU-

hsa-miR-32* CAAUUUAG-UGUGUGUGAUAUUU 69.6%

1 23

cel-miR-353 --CAAUUGCCAUGUGUUGGUAUU

hsa-miR-222* CUCAGUAGCCA-GUGUAGAUCCU 60.9%

1 23

cel-miR-353 CAAUUGCCAUGUGUUGGUAUU--

hsa-miR-450a -UUUUGCGAUGUGUUCCUAAUAU 60.9%

**miR-354: cel-miR-354, hsa-miR-1238**

1 23

cel-miR-354 --ACCUUGUUUGUUGCUGCUCCU

hsa-miR-1238 CUUCCUCGUCUGU--CUGCCCC- 60.9%

**miR-357: cel-miR-357, hsa-miR-21*, hsa-miR-155**

1 23

cel-miR-357 UAAAUGCCAGUCGUUGCAGGAGU

hsa-miR-155 UUAAUGCUAAUCGUGAUAGGGGU 69.6%

1 23

cel-miR-357 UAAAUGCCAGUCGUUGCAGGAGU

hsa-miR-21* -CAACACCAGUCGAUGG-GCUGU 60.9%

**miR-358: cel-miR-358, hsa-miR-598**

1 23

cel-miR-358 AUUGGU-AUCCCUGUCAAGGUCU

hsa-miR-598 -UACGUCAUCGUUGUCAUCGUCA 60.9%

**miR-359: cel-miR-359, hsa-miR-220c, hsa-miR-330-5p**

1 23

cel-miR-359 UCACUGGUCUUUCUCUGACGAA-

hsa-miR-220c ACACAGGGCUGU-UGUGAAGACU 60.9%

1 23

cel-miR-359 UCACUGGUCUU-UCUCUGACGAA

hsa-miR-330-5p UCUCUGGGCCUGUGUCUUAGGC- 60.9%

**miR-360: cel-miR-360, hsa-miR-943**

1 23

cel-miR-360 -UGACCGUAAUCCCGUUCACAA-

hsa-miR-943 CUGACUGU--UGCCGUCCUCCAG 60.9%

**miR-392: cel-miR-392, hsa-miR-101***

1 25

cel-miR-392 UAUCAUCGAUCAC-GUG-UGAUGA-

hsa-miR-101* ---CAGUUAUCACAGUGCUGAUGCU 60.0%

**miR-784: cel-miR-784, hsa-miR-379**

1 22

cel-miR-784 UGGCACAAUCUGCGUACGUAGA

hsa-miR-379 UGGUAGACUAUG-GAACGUAGG 68.2%

**miR-785: cel-miR-785, hsa-miR-29a***

1 24

cel-miR-785 UAAGUGAAUUGUUUUG-UGUAGA-

hsa-miR-29a* --ACUGAUUUCUUUUGGUGUUCAG 62.5%

**miR-786: cel-miR-786, hsa-miR-365**

1 23

cel-miR-786 UAAUGCCCUGAAUGAUGUUCAAU

hsa-miR-365 UAAUGCCCCUAAAAAUCCUUAU- 60.9%

**miR-787: cel-miR-787, hsa-miR-374b***

1 24

cel-miR-787 -UAAGCUCGUUUUAGUAUCUUUCG

hsa-miR-374b* CUUAGCAGGUUGUAUUAUCAUU-- 62.5%

**miR-788: cel-miR-788, hsa-miR-877***

1 25

cel-miR-788 UCCGCUUCUAACUUCCAUUUGCAG-

hsa-miR-877* UCCUCUUCUCCCUCC---UCCCAGG 60.0%

**miR-789: cel-miR-789, hsa-miR-615-3p**

1 23

cel-miR-789 UCCCUGCCUGGGUCACCAAUUGU

hsa-miR-615-3p UCCGAGCCUGGGUCUCCCUCUU- 65.2%

**miR-790: cel-miR-790, hsa-miR-218-2*, hsa-miR-182**

1 22

cel-miR-790 CUUGGCACUCGCGAACACCGCG

hsa-miR-218-2* CAUGGUUCUGUCAAGCACCGCG 68.2%

1 24

cel-miR-790 CUUGGCACUCGC-GAACAC-CGCG

hsa-miR-182 UUUGGCAAUGGUAGAACUCACACU 62.5%

**miR-791: cel-miR-791, hsa-miR-96**

1 24

cel-miR-791 UUUGGCACUCCGCAGAUAAG-GCA

hsa-miR-96 UUUGGCACUA-GCACAUUUUUGCU 66.7%

**miR-793: cel-miR-793, hsa-let-7a, hsa-let-7i, hsa-miR-98,**

**hsa-miR-545***

1 24

cel-miR-793 UGAGGUAUCUUAGUUAGA-CAGA-

hsa-let-7i UGAGGUAG--UAGUUUGUGCUGUU 62.5%

1 23

cel-miR-793 UGAGGUAUCUUAGUUAGACAGA-

hsa-let-7a UGAGGUAG-UAGGUUGUAUAGUU 60.9%

1 23

cel-miR-793 UGAGGUAUCUUAGUUAGACAGA-

hsa-miR-98 UGAGGUAG-UAAGUUGUAUUGUU 60.9%

1 23

cel-miR-793 UGAGGUA-UCUUAGUUAGACAGA

hsa-miR-545* UCAGUAAAUGUUUAUUAGAU-GA 60.9%

**miR-794: cel-miR-794, hsa-let-7g**

1 24

cel-miR-794 UGAGGUAAUCAUCGUUGU-CACU-

hsa-let-7g UGAGGUAGUAGU--UUGUACAGUU 66.7%

**miR-795: cel-miR-795, hsa-let-7b, hsa-let-7f**

1 24

cel-miR-795 UGAGGUAGAUUGAUCAGCGAGCUU

hsa-let-7b UGAGGUAGUAGGUU--GUGUGGUU 62.5%

1 24

cel-miR-795 UGAGGUAGAUUGAUCAGCGAGCUU

hsa-let-7f UGAGGUAG-UAGAUUGUAUAGUU- 62.5%

**miR-796: cel-miR-796, hsa-miR-1, hsa-miR-26b, hsa-miR-206**

1 23

cel-miR-796 UGGAAUGUAGUUGAGGUUAGUAA

hsa-miR-1 UGGAAUGUAAA-GAAGUAUGUAU 69.6%

1 23

cel-miR-796 UGGAAUGUAGUUGAGGUUAGUAA

hsa-miR-206 UGGAAUGUAAG-GAAGUGUGUGG 65.2%

1 23

cel-miR-796 UGGAAUGUAGUUGAGGUUAGUAA

hsa-miR-26b UUCAA-GUAAUUCAGGAUAGGU- 60.9%
