# Supplementary material for: Sequence Relationships among C. elegans, D. melanogaster and Human microRNAs Highlight the Extensive Conservation of microRNAs in Biology
Source: PLoS One. 2008 Jul 30;3(7):e2818. doi: 10.1371/journal.pone.0002818 (PMC2486268; doi:10.1371/journal.pone.0002818)
Supplement: Dataset S13 — Table and alignments of D. melanogaster and human miRNAs with 5′ homology. (0.44 MB DOC) [file pone.0002818.s017.doc]

**Supplementary Table S13: 82 *D. melanogaster* miRNAs are related at the 5’ end with 117 human miRNAs in 231 sequence relationships.** Superscript “less than” symbol (**<**) before miRNA names indicates allowed A-G base changes (G..U pairing) interrupting the 5’ 7nt homology block that groups miRNAs into families (see alignments below). Sequence similarities grouping miRNAs into families are summarized in the table and detailed in alignments. **(5’)** identifies 67 *Drosophila* miRNAs with <70% overall homology to some of their 5’ related human miRNAs—32 (dme-miR-2a, dme-miR-2b, dme-miR-2c, dme-miR-3, dme-miR-13a, dme-miR-13b, dme-miR-263a, dme-miR-274, dme-miR-276a, dme-miR-276b, dme-miR-277, dme-miR-279, dme-miR-281-1*, dme-miR-281-2*, dme-miR-283, dme-miR-286, dme-miR-306*, dme-miR-308, dme-miR-314, dme-miR-316, dme-miR-318, dme-miR-960, dme-miR-963, dme-miR-967, dme-miR-983, dme-miR-986, dme-miR-990, dme-miR-996, dme-miR-1001, dme-miR-1002, dme-miR-1003 and dme-miR-1016) are mostly related at the 5’ region with all their 5’ cognate miRNAs in humans (<60% overall similarity, not present in Datasets S12 and S14).

|  | **5’ End Sequence Related miRNAs** | |  | |
| --- | --- | --- | --- | --- |
| **miRNA Group ID** | ***D. melanogaster*** | ***H. sapiens*** | | **# Identical nt at 5' End (10nt)** |
| bantam  *UGA/GGAUCAU* | dme-bantam **(5’)** | hsa-miR-450b-3p | | 8 |
| let-7  *(G)AGGUAG(U)* | dme-let-7 **(5’)** | hsa-let-7a | | 10 |
|  |  | hsa-let-7b | | 10 |
|  |  | hsa-let-7c | | 10 |
|  |  | hsa-let-7f | | 10 |
|  |  | hsa-let-7g | | 10 |
|  |  | hsa-let-7i | | 10 |
|  |  | hsa-miR-98 | | 10 |
|  |  | hsa-let-7d | | 9 |
|  |  | hsa-let-7e | | 9 |
|  |  | hsa-miR-196a | | 7 |
|  |  | hsa-miR-196b | | 7 |
| miR-1 UGGAA/GUG | dme-miR-1 **(5’)** | hsa-miR-1 | | 10 |
|  |  | hsa-miR-206 | | 10 |
|  |  | **<**hsa-miR-122 | | 8 |
| miR-2a  *AUCACAG* | dme-miR-2a **(5’)** | hsa-miR-499-3p | | 7 |
| miR-2b  *AUCACAG* | dme-miR-2b **(5’)** | hsa-miR-499-3p | | 7 |
| miR-2c  *AUCACAG* | dme-miR-2c **(5’)** | hsa-miR-499-3p | | 7 |
| miR-3  *CUGGGCA* | dme-miR-3 **(5’)** | hsa-miR-612 | | 7 |
| miR-4  *(AU)AAAG/AC(UA/G)* | dme-miR-4 **(5’)** | hsa-miR-9* | | 10 |
|  |  | **<**hsa-miR-320 | | 7 |
|  |  | hsa-miR-340 | | 7 |
|  |  | **<**hsa-miR-548a-3p | | 6 |
|  |  | **<**hsa-miR-7 | | 5 |
| miR-6  *(A)UCACAG/A(U)* | dme-miR-6 **(5’)** | hsa-miR-27a | | 8 |
|  |  | hsa-miR-27b | | 8 |
|  |  | hsa-miR-128 | | 8 |
|  |  | hsa-miR-499-3p | | 7 |
|  |  | **<**hsa-miR-768-3p | | 7 |
| miR-7  *(G)AA/GG/AA/GCU(A/G)* | dme-miR-7 **(5’)** | hsa-miR-7 | | 10 |
|  |  | **<**hsa-miR-9* | | 5 |
|  |  | **<**hsa-miR-146a | | 5 |
|  |  | **<**hsa-miR-146b-5p | | 5 |
|  |  | **<**hsa-miR-548a-3p | | 5 |
| miR-8  *UAAUACU* | dme-miR-8 | hsa-miR-429 | | 10 |
|  |  | hsa-miR-200b | | 9 |
|  |  | hsa-miR-200c | | 9 |
| miR-9a  *UCUUUGG* | dme-miR-9a | hsa-miR-9 | | 10 |
| miR-9b  *UCUUUGG* | dme-miR-9b | hsa-miR-9 | | 9 |
| miR-9c  *UCUUUGG* | dme-miR-9c | hsa-miR-9 | | 8 |
| miR-10  *A/GCCCUGU* | dme-miR-10 **(5’)** | hsa-miR-10a | | 9 |
|  |  | hsa-miR-10b | | 9 |
|  |  | **<**hsa-miR-146-3p | | 7 |
| miR-11  *(A)UCACAG/A(U)* | dme-miR-11 **(5’)** | hsa-miR-499-3p | | 8 |
|  |  | hsa-miR-27a | | 7 |
|  |  | hsa-miR-27b | | 7 |
|  |  | hsa-miR-128 | | 7 |
|  |  | **<**hsa-miR-768-3p | | 6 |
| miR-12  *UGAGUAU* | dme-miR-12 **(5’)** | hsa-miR-496 | | 10 |
| miR-13a  *AUCACAG* | dme-miR-13a **(5’)** | hsa-miR-499-3p | | 7 |
| miR-13b  *AUCACAG* | dme-miR-13b **(5’)** | hsa-miR-499-3p | | 7 |
| miR-14  *GUCUUUU* | dme-miR-14 **(5’)** | hsa-miR-511 | | 7 |
| miR-31a  *GGCAAGA* | dme-miR-31a | hsa-miR-31 | | 9 |
| miR-31b  *GGCAAGA* | dme-miR-31b | hsa-miR-31 | | 9 |
| miR-33  *(GG)UG/ACAU(UG)* | dme-miR-33 **(5’)** | hsa-miR-18a | | 8 |
|  |  | hsa-miR-18b | | 8 |
|  |  | hsa-miR-33a | | 8 |
|  |  | hsa-miR-33b | | 8 |
|  |  | **<**hsa-miR-221 | | 8 |
| miR-34  *GGCAGUG* | dme-miR-34 **(5’)** | hsa-miR-34a | | 9 |
|  |  | hsa-miR-449a | | 9 |
|  |  | hsa-miR-34b* | | 8 |
|  |  | hsa-miR-34c-5p | | 8 |
|  |  | hsa-miR-449b | | 8 |
| miR-79  *AA/GA/GG/ACUA/G* | dme-miR-79 **(5’)** | hsa-miR-9* | | 9 |
|  |  | **<**hsa-miR-320 | | 7 |
|  |  | **<**hsa-miR-548a-3p | | 6 |
|  |  | **<**hsa-miR-7 | | 5 |
| miR-92a  *AUUG/ACAC* | dme-miR-92a **(5’)** | hsa-miR-25 | | 10 |
|  |  | hsa-miR-92a | | 9 |
|  |  | hsa-miR-367 | | 9 |
|  |  | hsa-miR-92b | | 8 |
|  |  | hsa-miR-32 | | 8 |
|  |  | hsa-miR-363 | | 7 |
|  |  | hsa-miR-885-5p | | 7 |
| miR-92b  *AUUG/ACAC* | dme-miR-92b **(5’)** | hsa-miR-367 | | 9 |
|  |  | hsa-miR-25 | | 8 |
|  |  | hsa-miR-92a | | 8 |
|  |  | hsa-miR-92b | | 8 |
|  |  | hsa-miR-363 | | 8 |
|  |  | hsa-miR-32 | | 7 |
|  |  | hsa-miR-885-5p | | 6 |
| miR-100  *ACCCGUA* | dme-miR-100 | hsa-miR-99a | | 9 |
|  |  | hsa-miR-100 | | 9 |
|  |  | hsa-miR-99b | | 8 |
| miR-124  *UAAGGCA* | dme-miR-124 **(5’)** | hsa-miR-124 | | 10 |
|  |  | hsa-miR-506 | | 9 |
| miR-125  *CCCUGA/GG* | dme-miR-125 **(5’)** | hsa-miR-125a-5p | | 10 |
|  |  | hsa-miR-125b | | 10 |
|  |  | **<**hsa-miR-331-3p | | 6 |
| miR-133  *UUGGUCC* | dme-miR-133 | hsa-miR-133a | | 9 |
|  |  | hsa-miR-133b | | 9 |
| miR-137  *UAUUGCU* | dme-miR-137 | hsa-miR-137 | | 8 |
| miR-184  *UGGACGG* | dme-miR-184 | hsa-miR-184 | | 10 |
| miR-190  *GAUAUGU* | dme-miR-190 | hsa-miR-190 | | 9 |
|  |  | hsa-miR-190b | | 9 |
| miR-193  *ACUGGCC* | dme-miR-193 **(5’)** | hsa-miR-193a-3p | | 9 |
|  |  | hsa-miR-193b | | 7 |
| miR-210  *UGUGCGU* | dme-miR-210 | hsa-miR-210 | | 9 |
| miR-219  *UGAUUGU* | dme-miR-219 | hsa-miR-219-5p | | 10 |
| miR-263a  *GUUAAUG* | dme-miR-263a **(5’)** | **<**hsa-miR-569 | | 7 |
| miR-263b  *UG/AG/ACACU* | dme-miR-263b **(5’)** | **<**hsa-miR-96 | | 8 |
|  |  | hsa-miR-183 | | 8 |
|  |  | **<**hsa-miR-514 | | 7 |
|  |  | **<**hsa-miR-200a | | 6 |
| miR-274  *UUUGUGA* | dme-miR-274 **(5’)** | hsa-miR-758 | | 9 |
| miR-276a  *AGGAA/GCU* | dme-miR-276a **(5’)** | **<**hsa-miR-28-5p | | 6 |
| miR-276b  *AGGAA/GCU* | dme-miR-276b **(5’)** | **<**hsa-miR-28-5p | | 7 |
| miR-277  *(U)AAA/GUGC(A)* | dme-miR-277 **(5’)** | **<**hsa-miR-148a | | 8 |
|  |  | **<**hsa-miR-302a | | 6 |
|  |  | **<**hsa-miR-302b | | 6 |
|  |  | **<**hsa-miR-302c | | 6 |
|  |  | **<**hsa-miR-302d | | 6 |
|  |  | **<**hsa-miR-519a | | 6 |
|  |  | **<**hsa-miR-519b-3p | | 6 |
|  |  | **<**hsa-miR-519c-3p | | 6 |
| miR-279  *(UG)ACUA/GG(AU)* | dme-miR-279 **(5’)** | hsa-miR-28-3p | | 7 |
|  |  | **<**hsa-miR-134 | | 6 |
| miR-281-1*  *AG/AAG/ACUG/A* | dme-miR-281-1* **(5’)** | **<**hsa-miR-146a | | 7 |
|  |  | **<**hsa-miR-146b-5p | | 7 |
|  |  | **<**hsa-miR-9* | | 6 |
|  |  | **<**hsa-miR-320 | | 6 |
|  |  | **<**hsa-miR-548a-3p | | 5 |
| miR-281-2*  *AG/AAG/ACUA/G* | dme-miR-281-2* **(5’)** | **<**hsa-miR-9* | | 7 |
|  |  | **<**hsa-miR-146a | | 6 |
|  |  | **<**hsa-miR-146b-5p | | 6 |
|  |  | **<**hsa-miR-320 | | 5 |
| miR-283  *UA/GAA/GUAU* | dme-miR-283 **(5’)** | **<**hsa-miR-496 | | 6 |
| miR-285  *A/GG/ACACCA* | dme-miR-285 **(5’)** | hsa-miR-29b | | 10 |
|  |  | hsa-miR-29c | | 10 |
|  |  | hsa-miR-29a | | 9 |
|  |  | **<**hsa-miR-21* | | 7 |
|  |  | **<**hsa-miR-593* | | 6 |
| miR-286  *(U)G/AACUA/GG(A)* | dme-miR-286 **(5’)** | hsa-miR-708* | | 7 |
|  |  | **<**hsa-miR-134 | | 6 |
| miR-304  *UAAUCUC* | dme-miR-304 | hsa-miR-216a | | 8 |
| miR-306*  *GGGG/AUCA* | dme-miR-306* **(5’)** | **<**hsa-miR-450b-3p | | 6 |
| miR-308  *AUCACAG* | dme-miR-308 **(5’)** | hsa-miR-499-3p | | 7 |
| miR-310  *AUUG/ACAC* | dme-miR-310 **(5’)** | hsa-miR-32 | | 9 |
|  |  | hsa-miR-92b | | 9 |
|  |  | hsa-miR-92a | | 8 |
|  |  | hsa-miR-25 | | 7 |
|  |  | hsa-miR-367 | | 7 |
|  |  | hsa-miR-363 | | 7 |
|  |  | **<**hsa-miR-885-5p | | 6 |
| miR-311  *AUUG/ACAC* | dme-miR-311 **(5’)** | hsa-miR-32 | | 10 |
|  |  | hsa-miR-92a | | 9 |
|  |  | hsa-miR-25 | | 8 |
|  |  | hsa-miR-92b | | 8 |
|  |  | hsa-miR-367 | | 8 |
|  |  | hsa-miR-363 | | 7 |
|  |  | **<**hsa-miR-885-5p | | 6 |
| miR-312  *AUUG/ACAC* | dme-miR-312 **(5’)** | hsa-miR-92a | | 10 |
|  |  | hsa-miR-25 | | 9 |
|  |  | hsa-miR-32 | | 9 |
|  |  | hsa-miR-92b | | 9 |
|  |  | hsa-miR-367 | | 9 |
|  |  | hsa-miR-363 | | 7 |
|  |  | **<**hsa-miR-885-5p | | 6 |
| miR-313  *AUUG/ACAC* | dme-miR-313 **(5’)** | hsa-miR-92a | | 10 |
|  |  | hsa-miR-25 | | 9 |
|  |  | hsa-miR-32 | | 9 |
|  |  | hsa-miR-92b | | 9 |
|  |  | hsa-miR-367 | | 9 |
|  |  | hsa-miR-363 | | 7 |
|  |  | **<**hsa-miR-885-5p | | 6 |
| miR-314  *UUUG/AAGC* | dme-miR-314 **(5’)** | **<**hsa-miR-498 | | 8 |
| miR-316  *UGUCUUU* | dme-miR-316 **(5’)** | hsa-miR-511 | | 8 |
| miR-318  *A/GCUGGGC* | dme-miR-318 **(5’)** | **<**hsa-miR-612 | | 6 |
| miR-375  *UUUGUUC* | dme-miR-375 | hsa-miR-375 | | 10 |
| miR-957  *AAACCGU* | dme-miR-957 **(5’)** | hsa-miR-451 | | 7 |
| miR-960  *UGAGUAU* | dme-miR-960 **(5’)** | hsa-miR-496 | | 9 |
| miR-961  *UUUGA/GUC* | dme-miR-961 **(5’)** | **<**hsa-miR-133a | | 8 |
|  |  | **<**hsa-miR-133b | | 8 |
| miR-963  *A/GAGGUAA/G* | dme-miR-963 **(5’)** | **<**hsa-let-7a | | 5 |
|  |  | **<**hsa-let-7b | | 5 |
|  |  | **<**hsa-let-7c | | 5 |
|  |  | **<**hsa-let-7d | | 5 |
|  |  | **<**hsa-let-7e | | 5 |
|  |  | **<**hsa-let-7f | | 5 |
|  |  | **<**hsa-let-7g | | 5 |
|  |  | **<**hsa-let-7i | | 5 |
|  |  | **<**hsa-miR-98 | | 5 |
| miR-964  *UUAGA/GAUA* | dme-miR-964 **(5’)** | **<**hsa-miR-651 | | 7 |
| miR-967  *A/GGAGAUA* | dme-miR-967 **(5’)** | **<**hsa-miR-620 | | 6 |
| miR-977  *(U)GAGA/GUA(U)* | dme-miR-977 **(5’)** | **<**hsa-let-7a | | 7 |
|  |  | **<**hsa-let-7b | | 7 |
|  |  | **<**hsa-let-7c | | 7 |
|  |  | **<**hsa-let-7f | | 7 |
|  |  | **<**hsa-let-7g | | 7 |
|  |  | **<**hsa-let-7i | | 7 |
|  |  | **<**hsa-miR-98 | | 7 |
|  |  | **<**hsa-let-7e | | 6 |
|  |  | **<**hsa-miR-202 | | 6 |
| miR-980  *AGCUGCC* | dme-miR-980 **(5’)** | hsa-miR-22 | | 7 |
| miR-983  *AUAAUAC* | dme-miR-983 **(5’)** | hsa-miR-655 | | 8 |
| miR-984  *GAGGUAA/G* | dme-miR-984 **(5’)** | hsa-let-7a | | 7 |
|  |  | hsa-let-7b | | 7 |
|  |  | hsa-let-7c | | 7 |
|  |  | hsa-let-7e | | 7 |
|  |  | hsa-let-7f | | 7 |
|  |  | hsa-let-7g | | 7 |
|  |  | hsa-let-7i | | 7 |
|  |  | hsa-miR-98 | | 7 |
|  |  | **<**hsa-let-7d | | 6 |
| miR-986  *UCUCG/AAA/G* | dme-miR-986 **(5’)** | **<**hsa-miR-513c | | 6 |
| miR-987  *AGUAAAU* | dme-miR-987 **(5’)** | hsa-miR-559 | | 10 |
|  |  | hsa-miR-545* | | 7 |
| miR-990  *UUCACCG/A* | dme-miR-990 **(5’)** | **<**hsa-miR-197 | | 6 |
| miR-993  *A/GAGCUCG/A* | dme-miR-993 **(5’)** | hsa-miR-99b* | | 8 |
|  |  | hsa-miR-99a* | | 7 |
|  |  | **<**hsa-miR-556-5p | | 5 |
| miR-995  *A/GG/ACACCA* | dme-miR-995 **(5’)** | hsa-miR-29a | | 8 |
|  |  | hsa-miR-29b | | 8 |
|  |  | hsa-miR-29c | | 8 |
|  |  | **<**hsa-miR-21* | | 6 |
|  |  | **<**hsa-miR-593* | | 6 |
| miR-996  *(UG/A)ACUA/GG(AU)* | dme-miR-996 **(5’)** | hsa-miR-28-3p | | 8 |
|  |  | **<**hsa-miR-708* | | 7 |
|  |  | **<**hsa-miR-134 | | 6 |
| miR-998  *A/GG/ACACCA* | dme-miR-998 **(5’)** | hsa-miR-29a | | 9 |
|  |  | hsa-miR-29b | | 9 |
|  |  | hsa-miR-29c | | 9 |
|  |  | **<**hsa-miR-21* | | 6 |
|  |  | <hsa-miR-593* | | 6 |
| miR-1001  *GGGUAAA/G* | dme-miR-1001 **(5’)** | **<**hsa-miR-555 | | 8 |
| miR-1002  *AAGUAG/AU* | dme-miR-1002 **(5’)** | hsa-miR-26a | | 7 |
|  |  | hsa-miR-26b | | 7 |
| miR-1003  *UCUCACA* | dme-miR-1003 **(5’)** | hsa-miR-342-3p | | 7 |
| miR-1010  *UUCACCU* | dme-miR-1010 **(5’)** | hsa-miR-412 | | 7 |
| miR-1016  *UUCACCU* | dme-miR-1016 **(5’)** | hsa-miR-412 | | 7 |

**Supplementary Alignments S13:**

**5’ sequence alignments of *D. melanogaster* and *H. sapiens* miRNAs with significant identity at the 5’ end (10nt).** Members of a group have ≥7 continuous nt of homology with at least one other group member. Nucleotides at the end of sequences indicate the number of residues identical to the related *D. melanogaster* miRNA. Grey shading denotes potential G..U pairing. Superscript “less than” (**<**) symbol before miRNA names indicates allowed A-G base change (G..U pairing) interrupting the 7nt homology block at the 5' end (10nt) which groups miRNAs into families. Superscript (5’) identifies fly miRNAs with 5’ homology but weak extended identity (<70%) to some of their 5’-related human sequences. 32 of these fly miRNAs (highlighted in blue) have <60% overall similarity with all their 5’-related human miRNAs.

**bantam: dme-bantam ^(5’)^, hsa-miR-450b-3p**

1

dme-bantam -UGAGAUCAUU

hsa-miR-450b-3p UUGGGAUCAU- 8nt

**let-7: dme-let-7 ^(5’)^, hsa-let-7a, hsa-let-7b, hsa-let-7c,**

**hsa-let-7d, hsa-let-7e, hsa-let-7f, hsa-let-7g, hsa-let-7i,**

**hsa-miR-98, hsa-miR-196a, hsa-miR-196b**

1

dme-let-7 UGAGGUAGUA-

hsa-let-7a UGAGGUAGUA- 10nt

hsa-let-7b UGAGGUAGUA- 10nt

hsa-let-7c UGAGGUAGUA- 10nt

hsa-let-7f UGAGGUAGUA- 10nt

hsa-let-7g UGAGGUAGUA- 10nt

hsa-let-7i UGAGGUAGUA- 10nt

hsa-miR-98 UGAGGUAGUA- 10nt

hsa-let-7d AGAGGUAGUA- 9nt

hsa-let-7e UGAGGUAGGA- 9nt

hsa-miR-196a -UAGGUAGUUU 7nt

hsa-miR-196b -UAGGUAGUUU 7nt

**miR-1: dme-miR-1 ^(5’)^, hsa-miR-1, hsa-miR-206, <hsa-miR-122**

1 10

dme-miR-1 UGGAAUGUAA

hsa-miR-1 UGGAAUGUAA 10nt

hsa-miR-206 UGGAAUGUAA 10nt

hsa-miR-122 UGGAGUGUGA 8nt

**miR-2a: dme-miR-2a ^(5’)^, hsa-miR-499-3p**

1

dme-miR-2a --UAUCACAGCC

hsa-miR-499-3p AACAUCACAG-- 7nt

**miR-2b: dme-miR-2b ^(5’)^, hsa-miR-499-3p**

1

dme-miR-2b --UAUCACAGCC

hsa-miR-499-3p AACAUCACAG-- 7nt

**miR-2c: dme-miR-2c ^(5’)^, hsa-miR-499-3p**

1

dme-miR-2c --UAUCACAGCC

hsa-miR-499-3p AACAUCACAG-- 7nt

**miR-3: dme-miR-3 ^(5’)^, hsa-miR-612**

1

dme-miR-3 UCACUGGGCA--

hsa-miR-612 --GCUGGGCAGG 7nt

**miR-4: dme-miR-4 ^(5’)^, <hsa-miR-7, hsa-miR-9*, <hsa-miR-320,**

**<hsa-miR-340, <hsa-miR-548a-3p**

1

dme-miR-4 --AUAAAGCUAG-

hsa-miR-320 ---AAAAGCUGGG 7nt

hsa-miR-548a-3p ---CAAAACUGGC 6nt

hsa-miR-7 -UGGAAGACUA-- 5nt

hsa-miR-340 UUAUAAAGCA--- 7nt

hsa-miR-9* --AUAAAGCUAG- 10nt

**miR-6: dme-miR-6 ^(5’)^, hsa-miR-27a, hsa-miR-27b, hsa-miR-128,**

**hsa-miR-499-3p, <hsa-miR-768-3p**

1

dme-miR-6 --UAUCACAGUG--

hsa-miR-27a ---UUCACAGUGG- 8nt

hsa-miR-27b ---UUCACAGUGG- 8nt

hsa-miR-128 ----UCACAGUGAA 8nt

hsa-miR-499-3p AACAUCACAG---- 7nt

hsa-miR-768-3p ----UCACAAUGCU 7nt

**miR-7: dme-miR-7 ^(5’)^, hsa-miR-7, <hsa-miR-9*, <hsa-miR-146a,**

**<hsa-miR-146b-5p, <hsa-miR-548a-3p**

1 12

dme-miR-7 UGGAAGACUA--

hsa-miR-7 UGGAAGACUA-- 10nt

hsa-miR-146a -UGAGAACUGA- 5nt

hsa-miR-146b-5p -UGAGAACUGA- 5nt

hsa-miR-548a-3p --CAAAACUGGC 5nt

hsa-miR-9* -AUAAAGCUAG- 5nt

**miR-8: dme-miR-8, hsa-miR-429, hsa-miR-200b, hsa-miR-200c**

1 10

dme-miR-8 UAAUACUGUC

hsa-miR-429 UAAUACUGUC 10nt

hsa-miR-200b UAAUACUGCC 9nt

hsa-miR-200c UAAUACUGCC 9nt

**miR-9a: dme-miR-9a, hsa-miR-9**

1 10

dme-miR-9a UCUUUGGUUA

hsa-miR-9 UCUUUGGUUA 10nt

**miR-9b: dme-miR-9b, hsa-miR-9**

1 10

dme-miR-9b UCUUUGGUGA

hsa-miR-9 UCUUUGGUUA 9nt

**miR-9c: dme-miR-9c, hsa-miR-9**

1 10

dme-miR-9c UCUUUGGUAU

hsa-miR-9 UCUUUGGUUA 8nt

**miR-10: dme-miR-10 ^(5’)^, dme-miR-10a, dme-miR-10b,**

**<hsa-miR-146b-3p**

1

dme-miR-10 -ACCCUGUAGA

hsa-miR-10a UACCCUGUAG 9nt

hsa-miR-10b UACCCUGUAG 9nt

hsa-miR-146b-3p UGCCCUGUGG 7nt

**miR-11: dme-miR-11 ^(5’)^, hsa-miR-27a, hsa-miR-27b,**

**hsa-miR-128, hsa-miR-499-3p, <hsa-miR-768-3p**

1

dme-miR-11 --CAUCACAGUC--

hsa-miR-27a ---UUCACAGUGG- 7nt

hsa-miR-27b ---UUCACAGUGG- 7nt

hsa-miR-128 ----UCACAGUGAA 7nt

hsa-miR-499-3p AACAUCACAG---- 8nt

hsa-miR-768-3p ----UCACAAUGCU 6nt

**miR-12: dme-miR-12 ^(5’)^, hsa-miR-496**

1 10

dme-miR-12 UGAGUAUUAC

hsa-miR-496 UGAGUAUUAC 10nt

**miR-13a: dme-miR-13a ^(5’)^, hsa-miR-499-3p**

1

dme-miR-13a --UAUCACAGCC

hsa-miR-499-3p AACAUCACAG-- 7nt

**miR-13b: dme-miR-13b ^(5’)^, hsa-miR-499-3p**

1

dme-miR-13b --UAUCACAGCC

hsa-miR-499-3p AACAUCACAG-- 7nt

**miR-14: dme-miR-14 ^(5’)^, hsa-miR-511**

1

dme-miR-14 UCAGUCUUUU-

hsa-miR-511 -GUGUCUUUUG 7nt

**miR-31a: dme-miR-31a, hsa-miR-31**

1 10

dme-miR-31a UGGCAAGAUG

hsa-miR-31 AGGCAAGAUG 9nt

**miR-31b: dme-miR-31b, hsa-miR-31**

1 10

dme-miR-31b UGGCAAGAUG

hsa-miR-31 AGGCAAGAUG 9nt

**miR-33: dme-miR-33 ^(5’)^, hsa-miR-18a, hsa-miR-18b,**

**hsa-miR-33a, hsa-miR-33b, < hsa-miR-221**

1

dme-miR-33 --AGGUGCAUUG--

hsa-miR-33a ----GUGCAUUGUA 8nt

hsa-miR-18a UAAGGUGCAU---- 8nt

hsa-miR-18b UAAGGUGCAU---- 8nt

hsa-miR-221 --AGCUACAUUG-- 8nt

hsa-miR-33b ----GUGCAUUGCU 8nt

**miR-34: dme-miR-34 ^(5’)^, hsa-miR-34a, hsa-miR-34b*,**

**hsa-miR-34c-5p, hsa-miR-449a, hsa-miR-449b**

1

dme-miR-34 -UGGCAGUGUG

hsa-miR-34a -UGGCAGUGUC 9nt

hsa-miR-34b* UAGGCAGUGU- 8nt

hsa-miR-34c-5p -AGGCAGUGUA 8nt

hsa-miR-449a -UGGCAGUGUA 9nt

hsa-miR-449b -AGGCAGUGUA 8nt

**miR-79: dme-miR-79 ^(5’)^, <hsa-miR-7, hsa-miR-9*,**

**<hsa-miR-320, <hsa-miR-548a-3p**

1

dme-miR-79 --UAAAGCUAGA

hsa-miR-9* -AUAAAGCUAG- 9nt

hsa-miR-320 --AAAAGCUGGG 7nt

hsa-miR-548a-3p --CAAAACUGGC 6nt

hsa-miR-7 UGGAAGACUA-- 5nt

**miR-92a: dme-miR-92a ^(5’)^, hsa-miR-25, hsa-miR-32,**

**hsa-miR-92a, hsa-miR-92b, hsa-miR-363,**

**hsa-miR-367, hsa-miR-885-5p**

1

dme-miR-92a --CAUUGCACUU

hsa-miR-885-5p UCCAUUACAC-- 7nt

hsa-miR-25 --CAUUGCACUU 10nt

hsa-miR-32 --UAUUGCACAU 8nt

hsa-miR-363 --AAUUGCACGG 7nt

hsa-miR-367 --AAUUGCACUU 9nt

hsa-miR-92a --UAUUGCACUU 9nt

hsa-miR-92b --UAUUGCACUC 8nt

**miR-92b: dme-miR-92b ^(5’)^, hsa-miR-25, hsa-miR-32,**

**hsa-miR-92a, hsa-miR-92b, hsa-miR-363,**

**hsa-miR-367, hsa-miR-885-5p**

1

dme-miR-92b --AAUUGCACUA

hsa-miR-25 --CAUUGCACUU 8nt

hsa-miR-32 --UAUUGCACAU 7nt

hsa-miR-363 --AAUUGCACGG 8nt

hsa-miR-367 --AAUUGCACUU 9nt

hsa-miR-885-5p UCCAUUACAC-- 6nt

hsa-miR-92a --UAUUGCACUU 8nt

hsa-miR-92b --UAUUGCACUC 8nt

**miR-100: dme-miR-100, hsa-miR-99a, hsa-miR-99b,**

**hsa-miR-100**

1 10

dme-miR-100 AACCCGUAAA

hsa-miR-99a AACCCGUAGA 9nt

hsa-miR-100 AACCCGUAGA 9nt

hsa-miR-99b CACCCGUAGA 8nt

**miR-124: dme-miR-124 ^(5’)^, hsa-miR-124, hsa-miR-506**

1 10

dme-miR-124 UAAGGCACGC

hsa-miR-124 UAAGGCACGC 10nt

hsa-miR-506 UAAGGCACCC 9nt

**miR-125: dme-miR-125 ^(5’)^, hsa-miR-125a-5p, hsa-miR-125b,**

**<hsa-miR-331-3p**

1

dme-miR-125 -UCCCUGAGAC

hsa-miR-125b -UCCCUGAGAC 10nt

hsa-miR-125a-5p -UCCCUGAGAC 10nt

hsa-miR-331-3p GCCCCUGGGC- 6nt

**miR-133: dme-miR-133, hsa-miR-133a, hsa-miR-133b**

1

dme-miR-133 -UUGGUCCCCU

hsa-miR-133a UUUGGUCCCC- 9nt

hsa-miR-133b UUUGGUCCCC- 9nt

**miR-137: dme-miR-137, hsa-miR-137**

1

dme-miR-137 -UAUUGCUUGA

hsa-miR-137 UUAUUGCUUA- 8nt

**miR-184: dme-miR-184, hsa-miR-184**

1 10

dme-miR-184 UGGACGGAGA

hsa-miR-184 UGGACGGAGA 10nt

**miR-190: dme-miR-190, hsa-miR-190, hsa-miR-190b**

1 10

dme-miR-190 AGAUAUGUUU

hsa-miR-190 UGAUAUGUUU 9nt

hsa-miR-190b UGAUAUGUUU 9nt

**miR-193: dme-miR-193 ^(5’)^, hsa-miR-193a-3p, hsa-miR-193b**

1

dme-miR-193 UACUGGCCUA

hsa-miR-193a-3p AACUGGCCUA 9nt

hsa-miR-193b AACUGGCCCU 7nt

**miR-210: dme-miR-210, hsa-miR-210**

1 10

dme-miR-210 UUGUGCGUGU

hsa-miR-210 CUGUGCGUGU 9nt

**miR-219: dme-miR-219, hsa-miR-219-5p**

1 10

dme-miR-219 UGAUUGUCCA

hsa-miR-219-5p UGAUUGUCCA 10nt

**miR-263a: dme-miR-263a ^(5’)^, <hsa-miR-569**

1

dme-miR-263a -GUUAAUGGCA

hsa-miR-569 AGUUAAUGAA- 7nt

**miR-263b:** **dme-miR-263b** **^(5’)^, <hsa-miR-96, hsa-miR-183,**

**<hsa-miR-200a, <hsa-miR-514**

1 12

dme-miR-263b CUUGGCACUG--

hsa-miR-96 UUUGGCACUA-- 8nt

hsa-miR-183 UAUGGCACUG-- 8nt

hsa-miR-200a --UAACACUGUC 6nt

hsa-miR-514 AUUGACACUU-- 7nt

**miR-274: dme-miR-274 ^(5’)^, hsa-miR-758**

1

dme-miR-274 UUUUGUGACC-

hsa-miR-758 -UUUGUGACCU 9nt

**miR-276a: dme-miR-276a ^(5’)^, <hsa-miR-28-5p**

1 10

dme-miR-276a UAGGAACUUC

hsa-miR-28-5p AAGGAGCUCA 6nt

**miR-276b: dme-miR-276b ^(5’)^, <hsa-miR-28-5p**

1 10

dme-miR-276b UAGGAACUUA

hsa-miR-28-5p AAGGAGCUCA 7nt

**miR-277: dme-miR-277 ^(5’)^, < hsa-miR-148a, <hsa-miR-302a,**

**<hsa-miR-302b, <hsa-miR-302c, <hsa-miR-302d,**

**<hsa-miR-519a, <hsa-miR-519b-3p, <hsa-miR-519c-3p,**

1 10

dme-miR-277 UAAAUGCACU

hsa-miR-148a UCAGUGCACU 8nt

hsa-miR-302a UAAGUGCUUC 6nt

hsa-miR-302b UAAGUGCUUC 6nt

hsa-miR-302c UAAGUGCUUC 6nt

hsa-miR-302d UAAGUGCUUC 6nt

hsa-miR-519a AAAGUGCAUC 6nt

hsa-miR-519b-3p AAAGUGCAUC 6nt

hsa-miR-519c-3p AAAGUGCAUC 6nt

**miR-279: dme-miR-279 ^(5’)^, hsa-miR-28-3p, <hsa-miR-134**

1

dme-miR-279 --UGACUAGAUC-

hsa-miR-134 UGUGACUGGU--- 6nt

hsa-miR-28-3p ---CACUAGAUUG 7nt

**miR-281-1*: dme-miR-281-1* ^(5’)^, <hsa-miR-9*, <hsa-miR-146a,**

**<hsa-miR-146b-5p, <hsa-miR-320,<hsa-miR-548a-3p**

1

dme-miR-281-1* AAGAGAGCUG--

hsa-miR-9* -AUAAAGCUAG- 6nt

hsa-miR-146a -UGAGAACUGA- 7nt

hsa-miR-146b-5p -UGAGAACUGA- 7nt

hsa-miR-320 --AAAAGCUGGG 6nt

hsa-miR-548a-3p --CAAAACUGGC 5nt

**miR-281-2*: dme-miR-281-2* ^(5’)^, <hsa-miR-9*, <hsa-miR-146a,**

**<hsa-miR-146b-5p, <hsa-miR-320**

1

dme-miR-281-2* AAGAGAGCUA--

hsa-miR-146a -UGAGAACUGA- 6nt

hsa-miR-146b-5p -UGAGAACUGA- 6nt

hsa-miR-320 --AAAAGCUGGG 5nt

hsa-miR-9* -AUAAAGCUAG- 7nt

**miR-283: dme-miR-283 ^(5’)^, <hsa-miR-496**

1 10

dme-miR-283 UAAAUAUCAG

hsa-miR-496 UGAGUAUUAC 6nt

**miR-285: dme-miR-285 ^(5’)^, <hsa-miR-21*, hsa-miR-29a,**

**hsa-miR-29b, hsa-miR-29c, <hsa-miR-593***

1 10

dme-miR-285 UAGCACCAUU

hsa-miR-21* CAACACCAGU 7nt

hsa-miR-29a UAGCACCAUC 9nt

hsa-miR-29b UAGCACCAUU 10nt

hsa-miR-29c UAGCACCAUU 10nt

hsa-miR-593* AGGCACCAGC 6nt

**miR-286: dme-miR-286 ^(5’)^, <hsa-miR-134, hsa-miR-708***

1

dme-miR-286 --UGACUAGACC

hsa-miR-134 UGUGACUGGU-- 6nt

hsa-miR-708* --CAACUAGACU 7nt

**miR-304: dme-miR-304, hsa-miR-216a**

1 10

dme-miR-304 UAAUCUCAAU

hsa-miR-216a UAAUCUCAGC 8nt

**miR-306*: dme-miR-306* ^(5’)^, <hsa-miR-450b-3p**

1

dme-miR-306* -GGGGGUCACU

hsa-miR-450b-3p UUGGGAUCAU- 6nt

**miR-308: dme-miR-308 ^(5’)^, hsa-miR-499-3p**

1 12

dme-miR-308 --AAUCACAGGA

hsa-miR-499-3p AACAUCACAG-- 7nt

**miR-310: dme-miR-310 ^(5’)^, hsa-miR-25, hsa-miR-32,**

**hsa-miR-92a, hsa-miR-92b, hsa-miR-363,**

**hsa-miR-367, <hsa-miR-885-5p**

1

dme-miR-310 --UAUUGCACAC

hsa-miR-32 --UAUUGCACAU 9nt

hsa-miR-25 --CAUUGCACUU 7nt

hsa-miR-885-5p UCCAUUACAC-- 6nt

hsa-miR-363 --AAUUGCACGG 7nt

hsa-miR-367 --AAUUGCACUU 7nt

hsa-miR-92a --UAUUGCACUU 8nt

hsa-miR-92b --UAUUGCACUC 9nt

**miR-311: dme-miR-311 ^(5’)^, hsa-miR-25, hsa-miR-32,**

**hsa-miR-92a, hsa-miR-92b, hsa-miR-363,**

**hsa-miR-367, <hsa-miR-885-5p**

1

dme-miR-311 --UAUUGCACAU

hsa-miR-32 --UAUUGCACAU 10nt

hsa-miR-25 --CAUUGCACUU 8nt

hsa-miR-885-5p UCCAUUACAC-- 6nt

hsa-miR-363 --AAUUGCACGG 7nt

hsa-miR-367 --AAUUGCACUU 8nt

hsa-miR-92a --UAUUGCACUU 9nt

hsa-miR-92b --UAUUGCACUC 8nt

**miR-312: dme-miR-312 ^(5’)^, hsa-miR-25, hsa-miR-32,**

**hsa-miR-92a, hsa-miR-92b, hsa-miR-363,**

**hsa-miR-367, <hsa-miR-885-5p**

1

dme-miR-312 --UAUUGCACUU

hsa-miR-25 --CAUUGCACUU 9nt

hsa-miR-32 --UAUUGCACAU 9nt

hsa-miR-92a --UAUUGCACUU 10nt

hsa-miR-92b --UAUUGCACUC 9nt

hsa-miR-363 --AAUUGCACGG 7nt

hsa-miR-367 --AAUUGCACUU 9nt

hsa-miR-885-5p UCCAUUACAC-- 6nt

**miR-313: dme-miR-313 ^(5’)^, hsa-miR-25, hsa-miR-32,**

**hsa-miR-92a, hsa-miR-92b, hsa-miR-363,**

**hsa-miR-367, <hsa-miR-885-5p**

1 12

dme-miR-313 --UAUUGCACUU

hsa-miR-25 --CAUUGCACUU 9nt

hsa-miR-32 --UAUUGCACAU 9nt

hsa-miR-92a --UAUUGCACUU 10nt

hsa-miR-92b --UAUUGCACUC 9nt

hsa-miR-363 --AAUUGCACGG 7nt

hsa-miR-367 --AAUUGCACUU 9nt

hsa-miR-885-5p UCCAUUACAC-- 6nt

**miR-314: dme-miR-314 ^(5’)^, <hsa-miR-498**

1

dme-miR-314 UAUUCGAGCC-

hsa-miR-498 -UUUCAAGCCA 8nt

**miR-316: dme-miR-316 ^(5’)^, hsa-miR-511**

1

dme-miR-316 -UGUCUUUUUC

hsa-miR-511 GUGUCUUUUG- 8nt

**miR-318: dme-miR-318 ^(5’)^, <hsa-miR-612**

1 12

dme-miR-318 UCACUGGGCU--

hsa-miR-612 --GCUGGGCAGG 6nt

**miR-375: dme-miR-375, hsa-miR-375**

1 10

dme-miR-375 UUUGUUCGUU

hsa-miR-375 UUUGUUCGUU 10nt

**miR-957: dme-miR-957 ^(5’)^, hsa-miR-451**

1

dme-miR-957 UGAAACCGUC--

hsa-miR-451 --AAACCGUUAC 7nt

**miR-960: dme-miR-960 ^(5’)^, hsa-miR-496**

1 10

dme-miR-960 UGAGUAUUCC

hsa-miR-496 UGAGUAUUAC 9nt

**miR-961: dme-miR-961 ^(5’)^, <hsa-miR-133a, <hsa-miR-133b**

1 10

dme-miR-961 UUUGAUCACC

hsa-miR-133a UUUGGUCCCC 8nt

hsa-miR-133b UUUGGUCCCC 8nt

**miR-963: dme-miR-963 ^(5’)^, <hsa-let-7a, <hsa-let-7b,**

**<hsa-let-7c, <hsa-let-7d, <hsa-let-7e, <hsa-let-7f,**

**<hsa-let-7g, <hsa-let-7i, <hsa-miR-98**

1

dme-miR-963 ACAAGGUAAA-

hsa-let-7a -UGAGGUAGUA 5nt

hsa-let-7b -UGAGGUAGUA 5nt

hsa-let-7c -UGAGGUAGUA 5nt

hsa-let-7d -AGAGGUAGUA 5nt

hsa-let-7e -UGAGGUAGGA 5nt

hsa-let-7f -UGAGGUAGUA 5nt

hsa-let-7g -UGAGGUAGUA 5nt

hsa-let-7i -UGAGGUAGUA 5nt

hsa-miR-98 -UGAGGUAGUA 5nt

**miR-964: dme-miR-964 ^(5’)^, <hsa-miR-651**

1

dme-miR-964 -UUAGAAUAGG

hsa-miR-651 UUUAGGAUAA- 7nt

**miR-967: dme-miR-967 ^(5’)^, <hsa-miR-620**

1

dme-miR-967 --AGAGAUACCU

hsa-miR-620 AUGGAGAUAG-- 6nt

**miR-977: dme-miR-977 ^(5’)^, <hsa-let-7a, <hsa-let-7b, <hsa-let-7c,**

**<hsa-let-7e, <hsa-let-7f, <hsa-let-7g, <hsa-let-7i,**

**<hsa-miR-98, <hsa-miR-202**

1 10

dme-miR-977 UGAGAUAUUC

hsa-let-7a UGAGGUAGUA 7nt

hsa-let-7b UGAGGUAGUA 7nt

hsa-let-7c UGAGGUAGUA 7nt

hsa-let-7e UGAGGUAGGA 6nt

hsa-let-7f UGAGGUAGUA 7nt

hsa-let-7g UGAGGUAGUA 7nt

hsa-let-7i UGAGGUAGUA 7nt

hsa-miR-98 UGAGGUAGUA 7nt

hsa-miR-202 AGAGGUAUAG 6nt

**miR-980: dme-miR-980 ^(5’)^, hsa-miR-22**

1 10

dme-miR-980 UAGCUGCCUU

hsa-miR-22 AAGCUGCCAG 7nt

**miR-983: dme-miR-983 ^(5’)^, hsa-miR-655**

1 10

dme-miR-983 AUAAUACGUU

hsa-miR-655 AUAAUACAUG 8nt

**miR-984: dme-miR-984 ^(5’)^, hsa-let-7a, hsa-let-7b, hsa-let-7c,**

**<hsa-let-7d, hsa-let-7e, hsa-let-7f, hsa-let-7g,**

**hsa-let-7i, hsa-miR-98**

1 10

dme-miR-984 UGAGGUAAAU

hsa-let-7e UGAGGUAGGA 7nt

hsa-let-7a UGAGGUAGUA 7nt

hsa-let-7b UGAGGUAGUA 7nt

hsa-let-7c UGAGGUAGUA 7nt

hsa-let-7d AGAGGUAGUA 6nt

hsa-let-7f UGAGGUAGUA 7nt

hsa-let-7g UGAGGUAGUA 7nt

hsa-let-7i UGAGGUAGUA 7nt

hsa-miR-98 UGAGGUAGUA 7nt

**miR-986: dme-miR-986 ^(5’)^, <hsa-miR-513c**

1

dme-miR-986 -UCUCGAAUAG

hsa-miR-513c UUCUCAAGGA- 6nt

**miR-987: dme-miR-987 ^(5’)^, hsa-miR-545*, hsa-miR559**

1

dme-miR-987 UAAAGUAAAU-

hsa-miR-545* -UCAGUAAAUG 7nt

hsa-miR-559 UAAAGUAAAU- 10nt

**miR-990: dme-miR-990 ^(5’)^, <hsa-miR-197**

1

dme-miR-990 AUUCACCGUU-

hsa-miR-197 -UUCACCACCU 6nt

**miR-993: dme-miR-993 ^(5’)^, hsa-miR-99a*, hsa-miR-99b*,**

**<hsa-miR-556-5p**

1 12

dme-miR-993 --GAAGCUCGUC

hsa-miR-99a* --CAAGCUCGCU 7nt

hsa-miR-99b* --CAAGCUCGUG 8nt

hsa-miR-556-5p GAUGAGCUCA-- 5nt

**miR-995: dme-miR-995 ^(5’)^, <hsa-miR-21*, hsa-miR-29a,**

**hsa-miR-29b, hsa-miR-29c, <hsa-miR-593***

1 10

dme-miR-995 UAGCACCACA

hsa-miR-21* CAACACCAGU 6nt

hsa-miR-29a UAGCACCAUC 8nt

hsa-miR-29b UAGCACCAUU 8nt

hsa-miR-29c UAGCACCAUU 8nt

hsa-miR-593* AGGCACCAGC 6nt

**miR-996: dme-miR-996 ^(5’)^, hsa-miR-28-3p, <hsa-miR-134,**

**<hsa-miR-708***

1

dme-miR-996 --UGACUAGAUU-

hsa-miR-28-3p ---CACUAGAUUG 8nt

hsa-miR-134 UGUGACUGGU--- 6nt

hsa-miR-708* --CAACUAGACU- 7nt

**miR-998: dme-miR-998 ^(5’)^, <hsa-miR-21*, hsa-miR-29a,**

**hsa-miR-29b, hsa-miR-29c, <hsa-miR-593***

1 10

dme-miR-998 UAGCACCAUG

hsa-miR-21* CAACACCAGU 6nt

hsa-miR-29a UAGCACCAUC 9nt

hsa-miR-29b UAGCACCAUU 9nt

hsa-miR-29c UAGCACCAUU 9nt

hsa-miR-593* AGGCACCAGC 6nt

**miR-1001: dme-miR-1001 ^(5’)^, <hsa-miR-555**

1 10

dme-miR-1001 UGGGUAAACU

hsa-miR-555 AGGGUAAGCU 8nt

**miR-1002: dme-miR-1002 ^(5’)^, hsa-miR-26a, hsa-miR-26b**

1

dme-miR-1002 -UUAAGUAGUG

hsa-miR-26a UUCAAGUAAU- 7nt

hsa-miR-26b UUCAAGUAAU- 7nt

**miR-1003: dme-miR-1003 ^(5’)^, hsa-miR-342-3p**

1 10

dme-miR-1003 UCUCACAUUU

hsa-miR-342-3p UCUCACACAG 7nt

**miR-1010: dme-miR-1010 ^(5’)^, hsa-miR-412**

1

dme-miR-1010 -UUUCACCUAU

hsa-miR-412 ACUUCACCUG- 7nt

**miR-1016: dme-miR-1016 ^(5’)^, hsa-miR-412**

1

dme-miR-1016 --UUCACCUCUC

hsa-miR-412 ACUUCACCUG-- 7nt
