# Supplementary material for: Sequence Relationships among C. elegans, D. melanogaster and Human microRNAs Highlight the Extensive Conservation of microRNAs in Biology
Source: PLoS One. 2008 Jul 30;3(7):e2818. doi: 10.1371/journal.pone.0002818 (PMC2486268; doi:10.1371/journal.pone.0002818)
Supplement: Dataset S14 — Table and alignments of D. melanogaster and human miRNAs with ≥70% overall sequence homology. (0.15 MB DOC) [file pone.0002818.s018.doc]

**Supplementary Table S14: ≥70% homology search of fly and human miRNA full sequences reveals 84 sequence relationships between 40 *D.melanogaster* miRNAs and 56 *H.sapiens* miRNAs.**

|  | **Sequence Related miRNAs** | |  |
| --- | --- | --- | --- |
| **miRNA Group ID** | ***D. melanogaster*** | ***H. sapiens*** | **Overall Identity (≥70%)** |
| let-7 | dme-let-7 | hsa-let-7a | 95.5 |
|  |  | hsa-let-7c | 90.9 |
|  |  | hsa-let-7e | 90.9 |
|  |  | hsa-let-7f | 90.9 |
|  |  | hsa-let-7b | 86.4 |
|  |  | hsa-let-7d | 86.4 |
|  |  | hsa-let-7g | 86.4 |
|  |  | hsa-miR-98 | 86.4 |
|  |  | hsa-let-7i | 77.3 |
| miR-1 | dme-miR-1 | hsa-miR-1 | 90.9 |
|  |  | hsa-miR-206 | 81.8 |
| miR-4 | dme-miR-4 | hsa-miR-9* | 77.3 |
| miR-7 | dme-miR-7 | hsa-miR-7 | 100.0 |
| miR-8 | dme-miR-8 | hsa-miR-141 | 82.6 |
|  |  | hsa-miR-200a | 82.6 |
|  |  | hsa-miR-200b | 78.3 |
|  |  | hsa-miR-200c | 78.3 |
|  |  | hsa-miR-429 | 78.3 |
| miR-9a | dme-miR-9a | hsa-miR-9 | 100.0 |
| miR-9b | dme-miR-9b | hsa-miR-9 | 83.3 |
| miR-9c | dme-miR-9c | hsa-miR-9 | 87.0 |
| miR-10 | dme-miR-10 | hsa-miR-10a | 91.3 |
|  |  | hsa-miR-10b | 87.0 |
|  |  | hsa-miR-100 | 81.8 |
|  |  | hsa-miR-99a | 77.3 |
| miR-11 | dme-miR-11 | hsa-miR-27b | 73.9 |
| miR-31a | dme-miR-31a | hsa-miR-31 | 78.3 |
| miR-31b | dme-miR-31b | hsa-miR-31 | 77.3 |
| miR-33 | dme-miR-33 | hsa-miR-33a | 78.3 |
|  |  | hsa-miR-33b | 72.7 |
| miR-34 | dme-miR-34 | hsa-miR-34a | 87.5 |
|  |  | hsa-miR-34c-5p | 79.2 |
|  |  | hsa-miR-449a | 73.1 |
|  |  | hsa-miR-34b* | 72.0 |
| miR-79 | dme-miR-79 | hsa-miR-9* | 75.0 |
| miR-92a | dme-miR-92a | hsa-miR-92a | 90.9 |
|  |  | hsa-miR-25 | 81.8 |
|  |  | hsa-miR-92b | 81.8 |
| miR-92b | dme-miR-92b | hsa-miR-92a | 86.4 |
|  |  | hsa-miR-92b | 86.4 |
|  |  | hsa-miR-25 | 77.3 |
| miR-100 | dme-miR-100 | hsa-miR-100 | 95.5 |
|  |  | hsa-miR-99a | 90.9 |
|  |  | hsa-miR-10a | 78.3 |
|  |  | hsa-miR-99b | 77.3 |
|  |  | hsa-miR-10b | 73.9 |
| miR-124 | dme-miR-124 | hsa-miR-124 | 87.0 |
| miR-125 | dme-miR-125 | hsa-miR-125b | 100.0 |
|  |  | hsa-miR-125a-5p | 87.5 |
|  |  | hsa-miR-10a | 70.8 |
|  |  | hsa-miR-10b | 70.8 |
| miR-133 | dme-miR-133 | hsa-miR-133a | 100.0 |
|  |  | hsa-miR-133b | 90.9 |
| miR-137 | dme-miR-137 | hsa-miR-137 | 87.0 |
| miR-184 | dme-miR-184 | hsa-miR-184 | 95.5 |
| miR-190 | dme-miR-190 | hsa-miR-190b | 79.2 |
|  |  | hsa-miR-190 | 75.0 |
| miR-193 | dme-miR-193 | hsa-miR-193a-3p | 81.8 |
| miR-210 | dme-miR-210 | hsa-miR-210 | 86.4 |
| miR-219 | dme-miR-219 | hsa-miR-219-5p | 91.3 |
| miR-263a | dme-miR-263a | hsa-miR-183 | 76.0 |
| miR-263b | dme-miR-263b | hsa-miR-183 | 81.8 |
|  |  | hsa-miR-182 | 70.8 |
| miR-285 | dme-miR-285 | hsa-miR-29b | 87.0 |
|  |  | hsa-miR-29c | 81.8 |
|  |  | hsa-miR-29a | 77.3 |
| miR-304 | dme-miR-304 | hsa-miR-216a | 73.9 |
| miR-306 | dme-miR-306 | hsa-miR-873 | 72.7 |
| miR-310 | dme-miR-310 | hsa-miR-92a | 81.8 |
|  |  | hsa-miR-92b | 81.8 |
| miR-311 | dme-miR-311 | hsa-miR-92a | 77.3 |
| miR-312 | dme-miR-312 | hsa-miR-92a | 81.8 |
|  |  | hsa-miR-25 | 77.3 |
|  |  | hsa-miR-92b | 72.7 |
| miR-313 | dme-miR-313 | hsa-miR-92a | 77.3 |
|  |  | hsa-miR-25 | 72.7 |
| miR-375 | dme-miR-375 | hsa-miR-375 | 77.3 |
| miR-984 | dme-miR-984 | hsa-let-7a | 75.0 |
|  |  | hsa-let-7d | 70.8 |
|  |  | hsa-let-7f | 70.8 |
|  |  | hsa-let-7g | 70.8 |
| miR-993 | dme-miR-993 | hsa-miR-100* | 73.9 |
| miR-995 | dme-miR-995 | hsa-miR-29a | 72.7 |
|  |  | hsa-miR-29c | 72.7 |

**Supplementary alignments S14:**

**Sequence alignments of *D.melanogaster-H.sapiens* miRNAs with ≥70% overall identity.** Members of a family are ≥70% identical to at least one other miRNA member. Identity to *D.melanogaster* miRNAs is given in percentage at the end of each *H.sapiens* homolog sequence. Shaded in grey indicate potential G..U pairing.

**let-7: dme-let-7, hsa-let-7a, hsa-let-7b, hsa-let-7c,**

**hsa-let-7d, hsa-let-7e, hsa-let-7f, hsa-let-7g,**

**hsa-miR-98, hsa-let-7i**

1 22

dme-let-7 UGAGGUAGUAGGUUGUAUAGU-

hsa-let-7a UGAGGUAGUAGGUUGUAUAGUU 95.5%

hsa-let-7b UGAGGUAGUAGGUUGUGUGGUU 86.4%

hsa-let-7c UGAGGUAGUAGGUUGUAUGGUU 90.9%

hsa-miR-98 UGAGGUAGUAAGUUGUAUUGUU 86.4%

hsa-let-7f UGAGGUAGUAGAUUGUAUAGUU 90.9%

hsa-let-7g UGAGGUAGUAGUUUGUACAGUU 86.4%

hsa-let-7i UGAGGUAGUAGUUUGUGCUGUU 77.3%

hsa-let-7d AGAGGUAGUAGGUUGCAUAGUU 86.4%

hsa-let-7e UGAGGUAGGAGGUUGUAUAGUU 90.9%

**miR-1: dme-miR-1, hsa-miR-1, hsa-miR-206**

1 22

dme-miR-1 UGGAAUGUAAAGAAGUAUGGAG

hsa-miR-1 UGGAAUGUAAAGAAGUAUGUAU 90.9%

hsa-miR-206 UGGAAUGUAAGGAAGUGUGUGG 81.8%

**miR-4: dme-miR-4, hsa-miR-9***

1 22

dme-miR-4 AUAAAGCUAGACAACC-AUUGA

hsa-miR-9* AUAAAGCUAGAUAACCGAAAGU 77.3%

**miR-7: dme-miR-7, hsa-miR-7**

1 23

dme-miR-7 UGGAAGACUAGUGAUUUUGUUGU

hsa-miR-7 UGGAAGACUAGUGAUUUUGUUGU 100%

**miR-8: dme-miR-8, hsa-miR-141, hsa-miR-200a, hsa-miR-200b,**

**hsa-miR-200c, hsa-miR-429**

1 23

dme-miR-8 UAAUACUGUCAGGUAAAGAUGUC

hsa-miR-429 UAAUACUGUCUGGUAAAACCGU- 78.3%

hsa-miR-141 UAACACUGUCUGGUAAAGAUGG- 82.6%

hsa-miR-200a UAACACUGUCUGGUAACGAUGU- 82.6%

hsa-miR-200b UAAUACUGCCUGGUAAUGAUGA- 78.3%

hsa-miR-200c UAAUACUGCCGGGUAAUGAUGGA 78.3%

**miR-9a: dme-miR-9a, hsa-miR-9**

1 23

dme-miR-9a UCUUUGGUUAUCUAGCUGUAUGA

hsa-miR-9 UCUUUGGUUAUCUAGCUGUAUGA 100.0%

**miR-9b: dme-miR-9b, hsa-miR-9**

1 24

dme-miR-9b UCUUUGGUGAUUUUAGCUGUAUG-

hsa-miR-9 UCUUUGGUUAUCU-AGCUGUAUGA 83.3%

**miR-9c: dme-miR-9c, hsa-miR-9**

1 23

dme-miR-9c UCUUUGGUAUUCUAGCUGUA-GA

hsa-miR-9 UCUUUGGUUAUCUAGCUGUAUGA 87.0%

**miR-10: dme-miR-10, hsa-miR-10a, hsa-miR-10b, hsa-miR-99a,**

**hsa-miR-100**

1 23

dme-miR-10 -ACCCUGUAGAUCCGAAUUUGU-

hsa-miR-10a UACCCUGUAGAUCCGAAUUUGUG 91.3%

hsa-miR-100 -AACCCGUAGAUCCGAACUUGUG 81.8%

hsa-miR-99a -AACCCGUAGAUCCGAUCUUGUG 77.3%

hsa-miR-10b UACCCUGUAGAACCGAAUUUGUG 87.0%

**miR-11: dme-miR-11, hsa-miR-27b**

1 23

dme-miR-11 CAUCACAGU--CUGAGUUCUUGC

hsa-miR-27b -UUCACAGUGGCUAAGUUCU-GC 73.9%

**miR-31a: dme-miR-31a, hsa-miR-31**

1 23

dme-miR-31a UGGCAAGAUGUCGGCAUAGCUGA

hsa-miR-31 AGGCAAGAUGCUGGCAUAGCU-- 78.3%

**miR-31b: dme-miR-31b, hsa-miR-31**

1 22

dme-miR-31b UGGCAAGAUGUCGGAAUAGCUG

hsa-miR-31 AGGCAAGAUGCUGGCAUAGCU- 77.3%

**miR-33: dme-miR-33, hsa-miR-33a, hsa-miR-33b**

1 23

dme-miR-33 AGGUGCAUUGUAGUCGCAUUG--

hsa-miR-33a --GUGCAUUGUAGUUGCAUUGCA 78.3%

hsa-miR-33b --GUGCAUUGCUGUUGCAUUGC- 72.7%

**miR-34: dme-miR-34, hsa-miR-34a, hsa-miR-34b*,**

**hsa-miR-34c-5p, hsa-miR-449a**

1 27

dme-miR-34 -UGGCAGUGUGG--UUAGCUGGUUGUG

hsa-miR-34a -UGGCAGUGUC---UUAGCUGGUUGU- 87.5%

hsa-miR-34b* UAGGCAGUGUCA--UUAGCUGAUUG-- 72.0%

hsa-miR-34c-5p -AGGCAGUGUAG--UUAGCUGAUUGC- 79.2%

hsa-miR-449a -UGGCAGUGUAUUGUUAGCUGGU---- 73.1%

**miR-79: dme-miR-79, hsa-miR-9***

1 24

dme-miR-79 -UAAAGCUAGAUUACC-AAAGCAU

hsa-miR-9* AUAAAGCUAGAUAACCGAAAGU-- 75.0%

**miR-92a: dme-miR-92a, hsa-miR-92a, hsa-miR-25,**

**hsa-miR-92b**

1 22

dme-miR-92a CAUUGCACUUGUCCCGGCCUAU

hsa-miR-25 CAUUGCACUUGUCUCGGUCUGA 81.8%

hsa-miR-92a UAUUGCACUUGUCCCGGCCUGU 90.9%

hsa-miR-92b UAUUGCACUCGUCCCGGCCUCC 81.8%

**miR-92b: dme-miR-92b, hsa-miR-25, hsa-miR-92a,**

**hsa-miR-92b**

1 22

dme-miR-92b AAUUGCACUAGUCCCGGCCUGC

hsa-miR-25 CAUUGCACUUGUCUCGGUCUGA 77.3%

hsa-miR-92a UAUUGCACUUGUCCCGGCCUGU 86.4%

hsa-miR-92b UAUUGCACUCGUCCCGGCCUCC 86.4%

**miR-100: dme-miR-100, hsa-miR-10a, hsa-miR-10b,**

**hsa-miR-99a, hsa-miR-99b, hsa-miR-100**

1 23

dme-miR-100 -AACCCGUAAAUCCGAACUUGUG

hsa-miR-100 -AACCCGUAGAUCCGAACUUGUG 95.5%

hsa-miR-10a UACCCUGUAGAUCCGAAUUUGUG 78.3%

hsa-miR-10b UACCCUGUAGAACCGAAUUUGUG 73.9%

hsa-miR-99a -AACCCGUAGAUCCGAUCUUGUG 90.9%

hsa-miR-99b -CACCCGUAGAACCGACCUUGCG 77.3%

**miR-124: dme-miR-124, hsa-miR-124**

1 23

dme-miR-124 UAAGGCACGCGGUGAAUGCCAAG

hsa-miR-124 UAAGGCACGCGGUGAAUGCC--- 87.0%

**miR-125: dme-miR-125, hsa-miR-10a, hsa-miR-10b,**

**hsa-miR-125a-5p, hsa-miR-125b**

1 26

dme-miR-125 -UCCCUG-AGACCCU--AACUUGUGA

hsa-miR-10a UACCCUGUAGAUCCG--AAUUUGUG- 70.8%

hsa-miR-10b UACCCUGUAGAACCG--AAUUUGUG- 70.8%

hsa-miR-125a-5p -UCCCUG-AGACCCUUUAACCUGUGA 87.5%

hsa-miR-125b -UCCCUG-AGACCCU--AACUUGUGA 100.0%

**miR-133: dme-miR-133, hsa-miR-133a, hsa-miR-133b**

1 22

dme-miR-133 UUGGUCCCCUUCAACCAGCUGU

hsa-miR-133a UUGGUCCCCUUCAACCAGCUGU 100.0%

hsa-miR-133b UUGGUCCCCUUCAACCAGCUA- 90.9%

**miR-137: dme-miR-137, hsa-miR-137**

1 23

dme-miR-137 -UAUUGCUUGAGAAUACACGUAG

hsa-miR-137 UUAUUGCUUAAGAAUACGCGUAG 87.0%

**miR-184: dme-miR-184, hsa-miR-184**

1 22

dme-miR-184 UGGACGGAGAACUGAUAAGGGC

hsa-miR-184 UGGACGGAGAACUGAUAAGGGU 95.5%

**miR-190: dme-miR-190, hsa-miR-190, hsa-miR-190b**

1 24

dme-miR-190 AGAUAUGUUUGAUAUUCUUGGUUG

hsa-miR-190 UGAUAUGUUUGAUAUAUUAGGU-- 75.0%

hsa-miR-190b UGAUAUGUUUGAUAU--UGGGUU- 79.2%

**miR-193: dme-miR-193, hsa-miR-193a-3p**

1 22

dme-miR-193 UACUGGCCUACUAAGUCCCAAC

hsa-miR-193a-3p AACUGGCCUACAAAGUCCCAGU 81.8%

**miR-210: dme-miR-210, hsa-miR-210**

1 22

dme-miR-210 UUGUGCGUGUGACAGCGGCUA-

hsa-miR-210 CUGUGCGUGUGACAGCGGCUGA 86.4%

**miR-219: dme-miR-219, hsa-miR-219-5p**

1 23

dme-miR-219 UGAUUGUCCAAACGCAAUUCUUG

hsa-miR-219-5p UGAUUGUCCAAACGCAAUUCU-- 91.3%

**miR-263a: dme-miR-263a, hsa-miR-183**

1 25

dme-miR-263a GUUAAUGGCACUGGAAGAAUUCAC-

hsa-miR-183 ---UAUGGCACUGGUAGAAUUCACU 76.0%

**miR-263b: dme-miR-263b, hsa-miR-182, hsa-miR-183**

1 24

dme-miR-263b CUUGGCACUGGGAGAAUUCAC---

hsa-miR-182 UUUGGCAAUGGUAGAACUCACACU 70.8%

hsa-miR-183 UAUGGCACUGGUAGAAUUCACU-- 81.8%

**miR-285: dme-miR-285, hsa-miR-29a, hsa-miR-29b,**

**hsa-miR-29c**

1 23

dme-miR-285 UAGCACCAUUCGAAAUCAGUGC-

hsa-miR-29a UAGCACCAUCUGAAAUCGGUU-- 77.3%

hsa-miR-29b UAGCACCAUUUGAAAUCAGUGUU 87.0%

hsa-miR-29c UAGCACCAUUUGAAAUCGGU--- 81.8%

**miR-304: dme-miR-304, hsa-miR-216a**

1 23

dme-miR-304 UAAUCUCAAUUUGUAAAUGUGAG

hsa-miR-216a UAAUCUCAGCUGGCAACUGUGA- 73.9%

**miR-306: dme-miR-306, hsa-miR-873**

1 22

dme-miR-306 UCAGGUACUUAGUGACUCUCAA

hsa-miR-873 GCAGGAACUU-GUGAGUCUCCU 72.7%

**miR-310: dme-miR-310, hsa-miR-92a, hsa-miR-92b**

1 22

dme-miR-310 UAUUGCACACUUCCCGGCCUUU

hsa-miR-92a UAUUGCACUUGUCCCGGCCUGU 81.8%

hsa-miR-92b UAUUGCACUCGUCCCGGCCUCC 81.8%

**miR-311: dme-miR-311, hsa-miR-92a**

1 22

dme-miR-311 UAUUGCACAUUCACCGGCCUGA

hsa-miR-92a UAUUGCACUUGUCCCGGCCUGU 77.3%

**miR-312: dme-miR-312, hsa-miR-25, hsa-miR-92a,**

**hsa-miR-92b**

1 22

dme-miR-312 UAUUGCACUUGAGACGGCCUGA

hsa-miR-25 CAUUGCACUUGUCUCGGUCUGA 77.3%

hsa-miR-92a UAUUGCACUUGUCCCGGCCUGU 81.8%

hsa-miR-92b UAUUGCACUCGUCCCGGCCUCC 72.7%

**miR-313: dme-miR-313, hsa-miR-92a, hsa-miR-25**

1 22

dme-miR-313 UAUUGCACUUUUCACAGCCCGA

hsa-miR-25 CAUUGCACUUGUCUCGGUCUGA 72.7%

hsa-miR-92a UAUUGCACUUGUCCCGGCCUGU 77.3%

**miR-375: dme-miR-375, hsa-miR-375**

1 22

dme-miR-375 UUUGUUCGUUUGGCUUAAGUUA

hsa-miR-375 UUUGUUCGUUCGGCUCGCGUGA 77.3%

**miR-984: dme-miR-984, hsa-let-7a, hsa-let-7d, hsa-let-7f,**

**hsa-let-7g**

1 24

dme-miR-984 UGAGGUAAAUACGGUUGGA-AUUU

hsa-let-7d AGAGGUAG-UA-GGUUGCAUAGUU 70.8%

hsa-let-7a UGAGGUAG-UA-GGUUGUAUAGUU 75.0%

hsa-let-7f UGAGGUAG-UA-GAUUGUAUAGUU 70.8%

hsa-let-7g UGAGGUAG-UA-GUUUGUACAGUU 70.8%

**miR-993: dme-miR-993, hsa-miR-100***

1 23

dme-miR-993 GAAGCUCGUCUCUACAGGUAUCU

hsa-miR-100* CAAGCUUGUAUCUAUAGGUAUG- 73.9%

**miR-995: dme-miR-995, hsa-miR-29a, hsa-miR-29c**

1 22

dme-miR-995 UAGCACCACAUGAU-UCGGCUU

hsa-miR-29a UAGCACCAUCUGAAAUCGGUUA 72.7%

hsa-miR-29c UAGCACCAUUUGAAAUCGGUUA 72.7%
