# Supplementary material for: Sequence Relationships among C. elegans, D. melanogaster and Human microRNAs Highlight the Extensive Conservation of microRNAs in Biology
Source: PLoS One. 2008 Jul 30;3(7):e2818. doi: 10.1371/journal.pone.0002818 (PMC2486268; doi:10.1371/journal.pone.0002818)
Supplement: Dataset S15 — Similarity table and sequence alignments of D. melanogaster-H. sapiens miRNAs with 60–69.9% overall identity. (0.31 MB DOC) [file pone.0002818.s019.doc]

**Supplementary Table S15: 60-69.9% overall sequence similarity search detects 151 sequence relationships between 76 *D.melanogaster* miRNAs and 112 human miRNAs.** 51 of these *Drosophila* miRNAs are not related in sequence to human miRNAs over the ≥70% threshold (Dataset S14). Of the 151 sequence relationships, 46 have ≥7nt of continuous similarity at the 5’ end (Dataset S13, sequence alignments below).

|  | Distantly Related miRNAs | |  |
| --- | --- | --- | --- |
| **miRNA**  **Group ID** | ***D.melanogaster*** | ***H. sapiens*** | Overall Identity **(60-69.9%)** |
| **dme-bantam** | dme-bantam | hsa-miR-450b-3p | 62.5 |
| **miR-iab-4-3p** | dme-miR-iab-4-3p | hsa-miR-144* | 68.0 |
| **miR-iab-4-5p** | dme-miR-iab-4-5p | hsa-miR-568 | 65.2 |
| **miR-iab4as-3p** | dme-miR-iab4as-3p | hsa-miR-144* | 65.2 |
| **let-7** | dme-let-7 | hsa-miR-625 | 60.9 |
| **miR-2b** | dme-miR-2b | hsa-miR-125b-2* | 60.0 |
| **miR-3** | dme-miR-3 | hsa-miR-330-5p | 64.0 |
| **miR-4** | dme-miR-4 | hsa-miR-142-5p | 65.2 |
| **miR-6** | dme-miR-6 | hsa-miR-128 | 69.6 |
|  |  | hsa-miR-27b | 66.7 |
|  |  | hsa-miR-27a | 62.5 |
|  |  | hsa-miR-513b | 60.9 |
|  |  | hsa-miR-513c | 60.9 |
|  |  | hsa-miR-553 | 60.9 |
| **miR-7** | dme-miR-7 | hsa-miR-338-3p | 69.6 |
|  |  | hsa-miR-553 | 62.5 |
|  |  | hsa-miR-224 | 60.0 |
| **miR-9a** | dme-miR-9a | hsa-miR-581 | 60.9 |
|  |  | hsa-miR-362-5p | 60.0 |
|  |  | hsa-miR-502-5p | 60.0 |
| **miR-9c** | dme-miR-9c | hsa-miR-581 | 63.6 |
|  |  | hsa-miR-502-5p | 62.5 |
|  |  | hsa-miR-362-5p | 61.5 |
|  |  | hsa-miR-500 | 60.0 |
| **miR-10** | dme-miR-10 | hsa-miR-99b | 69.6 |
|  |  | hsa-miR-125b | 69.6 |
|  |  | hsa-miR-125a-5p | 60.0 |
| **miR-11** | dme-miR-11 | hsa-miR-27a | 69.6 |
|  |  | hsa-miR-125b-2* | 60.0 |
| **miR-12** | dme-miR-12 | hsa-miR-496 | 65.2 |
| **miR-13a** | dme-miR-13a | hsa-miR-150 | 60.9 |
| **miR-13b** | dme-miR-13b | hsa-miR-150 | 65.2 |
| **miR-31a** | dme-miR-31a | hsa-miR-185 | 62.5 |
| **miR-33** | dme-miR-33 | hsa-miR-18a | 66.7 |
|  |  | hsa-miR-18b | 64.0 |
| **miR-34** | dme-miR-34 | hsa-miR-449b | 65.4 |
|  |  | hsa-miR-122 | 64.0 |
| **miR-87** | dme-miR-87 | hsa-miR-545 | 69.6 |
|  |  | hsa-miR-361-5p | 60.9 |
| **miR-92a** | dme-miR-92a | hsa-miR-885-5p | 66.7 |
| **miR-92b** | dme-miR-92b | hsa-miR-885-5p | 62.5 |
|  |  | hsa-miR-363 | 60.9 |
| **miR-100** | dme-miR-100 | hsa-miR-125b | 65.2 |
| **miR-124** | dme-miR-124 | hsa-miR-885-3p | 60.0 |
| **miR-125** | dme-miR-125 | hsa-miR-100 | 69.6 |
|  |  | hsa-miR-99a | 65.2 |
|  |  | hsa-miR-362-5p | 61.5 |
|  |  | hsa-miR-99b | 60.9 |
| **miR-193** | dme-miR-193 | hsa-miR-193b | 68.2 |
| **miR-263b** | dme-miR-263b | hsa-miR-635 | 65.2 |
| **miR-276*** | dme-miR-276* | hsa-miR-106a* | 60.9 |
| **miR-277** | dme-miR-277 | hsa-miR-577 | 60.9 |
| **miR-278** | dme-miR-278 | hsa-miR-658 | 60.0 |
| **miR-279** | dme-miR-279 | hsa-miR-30c | 62.5 |
|  |  | hsa-miR-425 | 62.5 |
|  |  | hsa-miR-30b | 60.9 |
| **miR-281** | dme-miR-281 | hsa-miR-493* | 64.0 |
|  |  | hsa-miR-485-3p | 62.5 |
| **miR-281-2*** | dme-miR-281-2* | hsa-miR-371-3p | 60.9 |
| **miR-283** | dme-miR-283 | hsa-miR-216a | 65.2 |
|  |  | hsa-miR-216b | 60.9 |
|  |  | hsa-miR-374a | 60.9 |
|  |  | hsa-miR-374b | 60.9 |
| **miR-286** | dme-miR-286 | hsa-miR-30b | 60.9 |
| **miR-287** | dme-miR-287 | hsa-miR-147 | 63.6 |
| **miR-303** | dme-miR-303 | hsa-miR-452 | 60.9 |
|  |  | hsa-miR-584 | 60.9 |
|  |  | hsa-miR-648 | 60.9 |
| **miR-304** | dme-miR-304 | hsa-miR-216b | 65.2 |
|  |  | hsa-miR-618 | 62.5 |
| **miR-306*** | dme-miR-306* | hsa-miR-493 | 62.5 |
| **miR-307** | dme-miR-307 | hsa-miR-338-5p | 60.9 |
| **miR-308** | dme-miR-308 | hsa-miR-216a | 60.9 |
| **miR-310** | dme-miR-310 | hsa-miR-25 | 63.6 |
|  |  | hsa-miR-885-5p | 63.6 |
| **miR-311** | dme-miR-311 | hsa-miR-25 | 68.2 |
|  |  | hsa-miR-92b | 68.2 |
|  |  | hsa-miR-32 | 65.2 |
| **miR-312** | dme-miR-312 | hsa-miR-367 | 63.6 |
|  |  | hsa-miR-183 | 62.5 |
| **miR-313** | dme-miR-313 | hsa-miR-92b | 68.2 |
|  |  | hsa-miR-32 | 63.6 |
| **mIR-315** | dme-miR-315 | hsa-miR-335* | 60.9 |
| **miR-316** | dme-miR-316 | hsa-miR-214* | 65.2 |
|  |  | hsa-miR-142-3p | 62.5 |
| **miR-318** | dme-miR-318 | hsa-miR-330-5p | 60.9 |
| **miR-932** | dme-miR-932 | hsa-miR-324-5p | 60.9 |
| **miR-954** | dme-miR-954 | hsa-miR-574-5p | 69.6 |
|  |  | hsa-miR-432 | 65.2 |
| **miR-957** | dme-miR-957 | hsa-miR-451 | 66.7 |
|  |  | hsa-miR-30b | 63.6 |
|  |  | hsa-miR-30a | 60.9 |
|  |  | hsa-miR-30c | 60.9 |
|  |  | hsa-miR-30d | 60.9 |
|  |  | hsa-miR-30e | 60.9 |
| **miR-961** | dme-miR-961 | hsa-miR-133a | 60.9 |
|  |  | hsa-miR-133b | 60.9 |
| **miR-963** | dme-miR-963 | hsa-miR-196a | 60.0 |
| **miR-964** | dme-miR-964 | hsa-miR-651 | 61.5 |
| **miR-966** | dme-miR-966 | hsa-miR-637 | 62.5 |
|  |  | hsa-miR-574-5p | 61.5 |
|  |  | hsa-miR-760 | 60.9 |
| **miR-968** | dme-miR-968 | hsa-miR-325 | 61.5 |
| **miR-974** | dme-miR-974 | hsa-miR-516a-5p | 60.9 |
| **miR-976** | dme-miR-976 | hsa-miR-450b-3p | 60.9 |
| **miR-977** | dme-miR-977 | hsa-let-7a | 65.2 |
|  |  | hsa-let-7b | 60.9 |
|  |  | hsa-let-7c | 60.9 |
|  |  | hsa-let-7e | 60.9 |
|  |  | hsa-let-7f | 60.9 |
|  |  | hsa-let-7i | 60.9 |
|  |  | hsa-miR-98 | 60.9 |
| **miR-978** | dme-miR-978 | hsa-miR-192* | 60.9 |
| **miR-980** | dme-miR-980 | hsa-miR-22 | 63.6 |
|  |  | hsa-miR-103 | 62.5 |
|  |  | hsa-miR-107 | 62.5 |
|  |  | hsa-miR-345 | 60.0 |
| **miR-982** | dme-miR-982 | hsa-miR-620 | 60.9 |
| **miR-984** | dme-miR-984 | hsa-let-7c | 66.7 |
|  |  | hsa-let-7e | 66.7 |
|  |  | hsa-let-7i | 62.5 |
|  |  | hsa-miR-98 | 62.5 |
|  |  | hsa-let-7b | 60.9 |
| **miR-987** | dme-miR-987 | hsa-miR-26b | 60.0 |
|  |  | hsa-miR-545* | 60.0 |
| **miR-990** | dme-miR-990 | hsa-miR-32 | 60.9 |
|  |  | hsa-miR-506 | 60.9 |
|  |  | hsa-miR-513a-3p | 60.0 |
| **miR-991** | dme-miR-991 | hsa-let-7g | 69.6 |
|  |  | hsa-let-7a | 65.2 |
|  |  | hsa-let-7f | 65.2 |
|  |  | hsa-let-7c | 60.9 |
|  |  | hsa-let-7d | 60.9 |
|  |  | hsa-let-7e | 60.9 |
|  |  | hsa-let-7i | 60.9 |
| **miR-993** | dme-miR-993 | hsa-miR-99a* | 65.2 |
|  |  | hsa-miR-10a* | 60.9 |
|  |  | hsa-miR-99b* | 60.9 |
| **miR-995** | dme-miR-995 | hsa-miR-15a | 66.7 |
|  |  | hsa-miR-15b | 66.7 |
|  |  | hsa-miR-29b | 65.2 |
| **miR-998** | dme-miR-998 | hsa-miR-29a | 68.2 |
|  |  | hsa-miR-29c | 68.2 |
|  |  | hsa-miR-29b | 65.2 |
| **miR-1000** | dme-miR-1000 | hsa-miR-581 | 63.6 |
| **miR-1001** | dme-miR-1001 | hsa-miR-331-5p | 68.2 |
| **miR-1003** | dme-miR-1003 | hsa-miR-192* | 69.6 |
| **miR-1005** | dme-miR-1005 | hsa-miR-542-5p | 60.9 |
| **miR-1010** | dme-miR-1010 | hsa-miR-412 | 61.5 |
| **miR-1011** | dme-miR-1011 | hsa-miR-137 | 65.2 |

**Supplementary Alignments S15:**

**Alignments of sequence-related *D.melanogaster* and *H. sapiens* miRNAs with overall 60-69.9% similarity.** Identity to *D.melanogaster* miRNAs is given in percentage at the end of each *H. sapiens* homolog sequence. Grey shading indicates potential G..U pairing.

**Bantam: dme-bantam, hsa-miR-450b-3p**

1 24

dme-bantam -UGAGAUCAUUUUGAAAGCUGAUU

hsa-miR-450b-3p UUGGGAUCAUUUUGCAUCCAUA-- 62.5%

**miR-iab-4-3p: dme-miR-iab-4-3p, hsa-miR-144***

1 25

dme-miR-iab-4-3p CGGUAUACCUUCAGUAUAC-GUAAC

hsa-miR-144* --GGAUAUCAUCA-UAUACUGUAAG 68.0%

**miR-iab-4-5p: dme-miR-iab-4-5p, hsa-miR-568**

1 23

dme-miR-iab-4-5p ACGUAUACUGAAUGUAUCCUGA-

hsa-miR-568 AUGUAUA---AAUGUAUACACAC 65.2%

**miR-iab4as-3p: dme-miR-iab4as-3p, hsa-miR-144***

1 23

dme-miR-iab4as-3p GGAUACAUUCAGUAUACGUUUA-

hsa-miR-144* GGAUAUCAUCA-UAUACUGUAAG 65.2%

**let-7: dme-let-7, hsa-miR-625**

1 23

dme-let-7 UGAGGUAGUAGGUUGUAUAGU--

hsa-miR-625 --AGGGGGAAAGUUCUAUAGUCC 60.9%

**miR-2b: dme-miR-2b, hsa-miR-125b-2***

1 25

dme-miR-2b UAUCACA-GCCAG-CUUUGAGGAGC

hsa-miR-125b-2* --UCACAAGUCAGGCUCUUGGGAC- 60.0%

**miR-3: dme-miR-3, hsa-miR-330-5p**

1 25

dme-miR-3 UCACUGGGCAAAGUGUGUCUCA---

hsa-miR-330-5p UCUCUGGGCC---UGUGUCUUAGGC 64.0%

**miR-4: dme-miR-4, hsa-miR-142-5p**

1 23

dme-miR-4 -AUAAAGCUAGACAACCAUUGA-

hsa-miR-142-5p CAUAAAG-UAGA-AAGCACUACU 65.2%

**miR-6: dme-miR-6, hsa-miR-27a, hsa-miR-27b, hsa-miR-128,**

**hsa-miR-513b, hsa-miR-513c, hsa-miR-553**

1 23

dme-miR-6 UAUCACAGUGG-CUGUUCUUUUU

hsa-miR-128 --UCACAGUGAACCGGUCUCUUU 69.6%

1 24

dme-miR-6 UAUCACAGUGGCU--GUUCUUUUU

hsa-miR-27b -UUCACAGUGGCUAAGUUCUGC-- 66.7%

1 24

dme-miR-6 UAUCACAGUGGCU--GUUCUUUUU

hsa-miR-27a -UUCACAGUGGCUAAGUUCCGC-- 62.5%

1 23

dme-miR-6 UAUCACA-GUGGCUGUUCUUUUU

hsa-miR-513b -UUCACAAGGAGGUGUCAUUUAU 60.9%

1 23

dme-miR-6 -UAUCACAGUGGCUGUUCUUUUU

hsa-miR-513c UUCUCAAGGAGG-UGUCGUUUAU 60.9%

1 23

dme-miR-6 UAUCACAGUG-GCUGUUCUUUUU

hsa-miR-553 -AAAACGGUGAGAUUUUGUUUU- 60.9%

**miR-7: dme-miR-7, hsa-miR-224, hsa-miR-338-3p, hsa-miR-553**

1 23

dme-miR-7 UGGAAGACUAGUGAUUUUGUUGU

hsa-miR-338-3p UCCAGCAUCAGUGAUUUUGUUG- 69.6%

1 24

dme-miR-7 UGGAAGACUA-GUGAUUUUGUUGU

hsa-miR-553 ---AAAACGGUGAGAUUUUGUUUU 62.5%

1 25

dme-miR-7 UGGAAG--ACUAGUGAUUUUGUUGU

hsa-miR-224 --CAAGUCACUAGUGGUUCCGUU-- 60.0%

**miR-9a: dme-miR-9a, hsa-miR-362-5p, hsa-miR-502-5p,**

**hsa-miR-581**

1 23

dme-miR-9a UCUUUGGUUAUCUAGCUGUAUGA

hsa-miR-581 UCUUGUGUUCUCUAGAUCAGU-- 60.9%

1 25

dme-miR-9a --UCUUUGGUUAUCUAGCUGUAUGA

hsa-miR-362-5p AAUCCUUGGA-ACCUAGGUGUGAGU 60.0%

1 25

dme-miR-9a -UCUUUGGUUAUCUAGCUG-UAUGA

hsa-miR-502-5p AUCCUUGCU-AUCUGGGUGCUA--- 60.0%

**miR-9c: dme-miR-9c, hsa-miR-362-5p, hsa-miR-500,**

**hsa-miR-502-5p, hsa-miR-581**

1 22

dme-miR-9c UCUUUGGUAUUCUAGCUGUAGA

hsa-miR-581 UCUUGUGUUCUCUAGAUC-AGU 63.6%

1 24

dme-miR-9c -UCUUUGGUAUUCUAGCUG-UAGA

hsa-miR-502-5p AUCCUUGCUAU-CUGGGUGCUA-- 62.5%

1 26

dme-miR-9c --UCUUUGGUAUUCUAGCUGUAGA--

hsa-miR-362-5p AAUCCUUGG-AACCUAGGUGU-GAGU 61.5%

1 25

dme-miR-9c ---UCUUUGGUAUUCUAGCUGUAGA

hsa-miR-500 UAAUCCUUGCUAC-CUGGGUG-AGA 60.0%

**miR-10: dme-miR-10, hsa-miR-99b, hsa-miR-125a-5p,**

**hsa-miR-125b**

1 23

dme-miR-10 -ACCCUGUAGAUCCGAAUUUGU-

hsa-miR-99b CACCC-GUAGAACCGACCUUGCG 69.6%

1 23

dme-miR-10 ACCCUGUAGAUCCGAAUUUGU--

hsa-miR-125b UCCCUG-AGACCCUAACUUGUGA 69.6%

1 25

dme-miR-10 ACCCUGUAGAUCCG--AAUUUGU--

hsa-miR-125a-5p UCCCUG-AGACCCUUUAACCUGUGA 60.0%

**miR-11: dme-miR-11, hsa-miR-27a, hsa-miR-125b-2***

1 23

dme-miR-11 CAUCACAGU--CUGAGUUCUUGC

hsa-miR-27a -UUCACAGUGGCUAAGUUCC-GC 69.6%

1 25

dme-miR-11 CAUCACA-GUCUGAGUUCUUGC---

hsa-miR-125b-2* --UCACAAGUCAG-GCUCUUGGGAC 60.0%

**miR-12: dme-miR-12, hsa-miR-496**

1 23

dme-miR-12 UGAGUAUUACAUCAGGUACUGGU

hsa-miR-496 UGAGUAUUACAUG-GCCAAUCUC 65.2%

**miR-13a: dme-miR-13a, hsa-miR-150**

1 23

dme-miR-13a UAUCACAGCCAUUUUGAUGAGU-

hsa-miR-150 UCUCCCAACCCUUGU-ACCAGUG 60.9%

**miR-13b: dme-miR-13b, hsa-miR-150**

1 23

dme-miR-13b UAUCACAGCCAUUUUGACGAGU-

hsa-miR-150 UCUCCCAACCCUUGU-ACCAGUG 65.2%

**miR-31a: dme-miR-31, hsa-miR-185**

1 24

dme-miR-31a UGGCAAGAUGUCGGCA-UAGCUGA

hsa-miR-185 UGGAGAGAAA--GGCAGUUCCUGA 62.5%

**miR-33: dme-miR-33, hsa-miR-18a, hsa-miR-18b**

1 24

dme-miR-33 --AGGUGCAUUGUAGU-CGCAUUG

hsa-miR-18a UAAGGUGCAUC-UAGUGCAGAUAG 66.7%

1 24

dme-miR-33 --AGGUGCAUUGUAGU-CGCAUUG

hsa-miR-18b UAAGGUGCAUC-UAGUGCAGUUAG 64.0%

**miR-34: dme-miR-34, hsa-miR-122, hsa-miR-449b**

1 26

dme-miR-34 UGGCAGUGUG--GUUAGCUGGUUGUG

hsa-miR-449b AGGCAGUGUAUUGUUAGCUGGC---- 65.4%

1 25

dme-miR-34 UGGCAGUGUGGUUA-GCUGGUUGUG

hsa-miR-122 UGG-AGUGUGACAAUGGUGUUUG-- 64.0%

**miR-87: dme-miR-87, hsa-miR-361-5p, hsa-miR-545**

1 23

dme-miR-87 UUGAGCAAA-AUUUCAGGUGUG-

hsa-miR-545 -UCAGCAAACAUUUAUUGUGUGC 69.6%

1 23

dme-miR-87 UUGAGCAAAAUUUC-AGGUGUG-

hsa-miR-361-5p -UUAUCAGAAUCUCCAGGGGUAC 60.9%

**miR-92a: dme-miR-92a, hsa-miR-885-5p**

1 24

dme-miR-92a --CAUUGCACUUGUCCCGGCCUAU

hsa-miR-885-5p UCCAUUACACU--ACCCUGCCUCU 66.7%

**miR-92b: dme-miR-92b, hsa-miR-363, hsa-miR-885-5p**

1 24

dme-miR-92b --AAUUGCACUAGUCCCGGCCUGC

hsa-miR-885-5p UCCAUUACACUA--CCCUGCCUCU 62.5%

1 23

dme-miR-92b AAUUGCACUAGUCCCGGCCUGC-

hsa-miR-363 AAUUGCACG-GUAUCCAUCUGUA 60.9%

**miR-100: dme-miR-100, hsa-miR-125b**

1 23

dme-miR-100 AACCCGUAAAUCCGAACUUGUG-

hsa-miR-125b -UCCCUGAGACCCUAACUUGUGA 65.2%

**miR-124: dme-miR-124, hsa-miR-885-3p**

1 25

dme-miR-124 UAAGGCA-CGCGGUGAA-UGCCAAG

hsa-miR-885-3p --AGGCAGCGGGGUGUAGUGGAUA- 60.0%

**miR-125: dme-miR-125, hsa-miR-99a, hsa-miR-99b,**

**hsa-miR-100, hsa-miR-362-5p**

1 23

dme-miR-125 -UCCCUGAGACCCUAACUUGUGA

hsa-miR-100 AACCCGUAGAUCCGAACUUGUG- 69.6%

1 23

dme-miR-125 -UCCCUGAGACCCUAACUUGUGA

hsa-miR-99a AACCCGUAGAUCCGAUCUUGUG- 65.2%

1 26

dme-miR-125 --UCCCUGAGACCCUAACUUGUGA--

hsa-miR-362-5p AAUCCUUG-GAACCUAGGU-GUGAGU 61.5%

1 23

dme-miR-125 -UCCCUGAGACCCUAACUUGUGA

hsa-miR-99b CACCCGUAGAACCGACCUUGCG- 60.9%

**miR-193: dme-miR-193, hsa-miR-193b**

1 22

dme-miR-193 UACUGGCCUACUAAGUCCCAAC

hsa-miR-193b AACUGGCCCUCAAAGUCCCGCU 68.2%

**miR-263b: dme-miR-263b, hsa-miR-635**

1 23

dme-miR-263b -CUUGG-CACUGGGAGAAUUCAC

hsa-miR-635 ACUUGGGCACUGAAACAAUGUCC 65.2%

**miR-276*: dme-miR-276*, hsa-miR-106a***

1 23

dme-miR-276* CAGCGAGGUAUAGAGUUCCUACG

hsa-miR-106a* CUGCAAUGUAAGCACUUCUUAC- 60.9%

**miR-277: dme-miR-277, hsa-miR-577**

1 23

dme-miR-277 UAAAUGCACUAUCUGGUACGACA

hsa-miR-577 UAGAUAAAAUAU-UGGUACCUG- 60.9%

**miR-278: dme-miR-278, hsa-miR-658**

1 25

dme-miR-278 -UCGGUGGGACUUUCGUCCGUUU--

hsa-miR-658 GGCGGAGGGAAGUAGGUCCGUUGGU 60.0%

**miR-279: dme-miR-279, hsa-miR-30b, hsa-miR-30c,**

**hsa-miR-425**

1 24

dme-miR-279 UGACUAGAUCC-ACACUCAUUAA-

hsa-miR-30c UGUAAACAUCCUACACUC-UCAGC 62.5%

1 24

dme-miR-279 --UGACUAGAUCCACACUCAUUAA

hsa-miR-425 AAUGACACGAUC-ACUCCCGUUGA 62.5%

1 23

dme-miR-279 UGACUAGAUCC-ACACUCAUUAA

hsa-miR-30b UGUAAACAUCCUACACUCAGCU- 60.9%

**miR-281: dme-miR-281, hsa-miR-493*, hsa-miR-485-3p**

1 25

dme-miR-281 -UGU-CAUGGAAUUGCUCUCUUUGU

hsa-miR-493* UUGUACAUGGUAG-GCUUUCAUU-- 64.0%

1 24

dme-miR-281 UGUCAUGGAAUUGCUCUCUU-UGU

hsa-miR-485-3p -GUCAUACACG-GCUCUCCUCUCU 62.5%

**miR-281-2*: dme-miR-281-2*, hsa-miR-371-3p**

1 23

dme-miR-281-2* AAGAGA-GCUAUCCGUCGACAGU

hsa-miR-371-3p AAGUGCCGCCAUCUUUUGAGUGU 60.9%

**miR-283: dme-miR-283, hsa-miR-216a, hsa-miR-216b,**

**hsa-miR-374a, hsa-miR-374b**

1 23

dme-miR-283 UAAAUAUCAGCUGGUAAUUCU--

hsa-miR-216a -UAAUCUCAGCUGGCAACUGUGA 65.2%

1 23

dme-miR-283 UAAAUAUCAGCUGGUAAUUCU--

hsa-miR-216b -AAAUCUCUGCAGGCAAAUGUGA 60.9%

1 23

dme-miR-283 -UA-AAUAUCAGCUGGUAAUUCU

hsa-miR-374a UUAUAAUACAACCUGAUAAGUG- 60.9%

1 23

dme-miR-283 -UA-AAUAUCAGCUGGUAAUUCU

hsa-miR-374b AUAUAAUACAACCUGCUAAGUG- 60.9%

**miR-286: dme-miR-286, hsa-miR-30b**

1 23

dme-miR-286 UGACUAGACCGAACACUCGUGCU

hsa-miR-30b UGUAAACAUCCUACACUCA-GCU 60.9%

**miR-287: dme-miR-287, hsa-miR-147**

1 22

dme-miR-287 -UGUGUUGAAAAUCGUUUGCAC

hsa-miR-147 GUGUGUGGAAAUGCUUCUGC-- 63.6%

**miR-303: dme-miR-303, hsa-miR-452, hsa-miR-584,**

**hsa-miR-648**

1 23

dme-miR-303 UUUAGGUUUCACAGGAAACUGGU

hsa-miR-452 AACUGUUUGCAGAGGAAACUGA- 60.9%

1 23

dme-miR-303 UUUAGGUUUCACAGGAAACUGGU

hsa-miR-584 UUAUGGUUUGCCUGGGA-CUGAG 60.9%

1 23

dme-miR-303 UUUAGGUUUCACAGGAAACUGGU

hsa-miR-648 ---AAGUGUG-CAGGGCACUGGU 60.9%

**miR-304: dme-miR-304, hsa-miR-216b, hsa-miR-618**

1 23

dme-miR-304 UAAUCUCAAUUUGUAAAUGUGAG

hsa-miR-216b AAAUCUCUGCAGGCAAAUGUGA- 65.2%

1 24

dme-miR-304 UAAUCUCAAUUUGUAAAUGUGAG-

hsa-miR-618 -AAACUCUACUUGUCCUUCUGAGU 62.5%

**miR-306*: dme-miR-306*, hsa-miR-493**

1 24

dme-miR-306* -GGGGGUC-ACUCUGUGCCUGUGC

hsa-miR-493 UGAAGGUCUACUGUGUGCCAGG-- 62.5%

**miR-307: dme-miR-307, hsa-miR-338-5p**

1 23

dme-miR-307 UCACAACCUCCUUGAG-UGAG--

hsa-miR-338-5p -AACAAUAUCCUGGUGCUGAGUG 60.9%

**miR-308: dme-miR-308, hsa-miR-216a**

1 23

dme-miR-308 -AAUCACAGGAUUAUACUGUGAG

hsa-miR-216a UAAUCUCAGCUGGCAACUGUGA- 60.9%

**miR-310: dme-miR-310, hsa-miR-25, hsa-miR-885-5p**

1 22

dme-miR-310 UAUUGCACACUUCCCGGCCUUU

hsa-miR-25 CAUUGCACUUGUCUCGGUCUGA 63.6%

1 22

dme-miR-310 UAUUGCACACUUCCCGGCCUUU

hsa-miR-885-5p UCCAUUACACUACCCUGCCUCU 63.6%

**miR-311: dme-miR-311, hsa-miR-25, hsa-miR-32, hsa-miR-92b**

1 22

dme-miR-311 UAUUGCACAUUCACCGGCCUGA

hsa-miR-25 CAUUGCACUUGUCUCGGUCUGA 68.2%

1 22

dme-miR-311 UAUUGCACAUUCACCGGCCUGA

hsa-miR-92b UAUUGCACUCGUCCCGGCCUCC 68.2%

1 23

dme-miR-311 UAUUGCACAUUCACCGGCCUGA-

hsa-miR-32 UAUUGCACAUU-ACUAAGUUGCA 65.2%

**miR-312: dme-miR-312, hsa-miR-183, hsa-miR-367**

1 22

dme-miR-312 UAUUGCACUUGAGACGGCCUGA

hsa-miR-367 AAUUGCACUUUAGCAAUGGUGA 63.6%

1 24

dme-miR-312 UAUUGCACUUG-AGACGGC-CUGA

hsa-miR-183 UAUGGCACUGGUAGAAUUCACU-- 62.5%

**miR-313: dme-miR-313, hsa-miR-32, hsa-miR-92b**

1 22

dme-miR-313 UAUUGCACUUUUCACAGCCCGA

hsa-miR-92b UAUUGCACUCGUCCCGGCCUCC 68.2%

1 22

dme-miR-313 UAUUGCACUUUUCACAGCCCGA

hsa-miR-32 UAUUGCACAUUACUAAGUUGCA 63.6%

**miR-315: dme-miR-315, hsa-miR-335***

1 23

dme-miR-315 -UUUUGAUUGUUGCUCAGAAAGC

hsa-miR-335* UUUUUCAUUAUUGCUCCUGACC- 60.9%

**miR-316: dme-miR-316, hsa-miR-142-3p, hsa-miR-214***

1 23

dme-miR-316 UGUCUUUUUCCGCUUACUG-GCG

hsa-miR-214* UGCCUGUCUACACUUGCUGUGC- 65.2%

1 24

dme-miR-316 UGUCUU-UUUCCG-CUUACUGGCG

hsa-miR-142-3p UGUAGUGUUUCCUACUUUAUGGA- 62.5%

**miR-318: dme-miR-318, hsa-miR-330-5p**

1 23

dme-miR-318 UCACUGGGCUU-UGUUUAUCUCA

hsa-miR-330-5p UCUCUGGGCCUGUGUCUUAGGC- 60.9%

**miR-932: dme-miR-932, hsa-miR-324-5p**

1 23

dme-miR-932 UCAAUUCCGUAGUGCAUUGCAG-

hsa-miR-324-5p CGCAUCCCCUAGGGCAUUGGUGU 60.9%

**miR-954: dme-miR-954, hsa-miR-432, hsa-miR-574-5p**

1 23

dme-miR-954 UCUGGGUGU-UGCGUU-GUGUGU

hsa-miR-574-5p UGAGUGUGUGUGUGUGAGUGUGU 69.6%

1 23

dme-miR-954 UCUGGGUGUUG--CGUUGUGUGU

hsa-miR-432 UCUUGGAGUAGGUCAUUGGGUGG 65.2%

**miR-957: dme-miR-957, hsa-miR-30a, hsa-miR-30b,**

**hsa-miR-30c, hsa-miR-30d, hsa-miR-30e,**

**hsa-miR-451**

1 24

dme-miR-957 UGAAACCGU--CCAAAACUGAGGC

hsa-miR-451 --AAACCGUUACCAUUACUGAGUU 66.7%

1 22

dme-miR-957 UGAAACCGUCCAAAACUGAGGC

hsa-miR-30b UGUAAACAUCCUACACUCAGCU 63.6%

1 23

dme-miR-957 UGAAACCGUCCAAAACUG-AGGC

hsa-miR-30a UGUAAACAUCCUCGACUGGAAG- 60.9%

1 23

dme-miR-957 UGAAACCGUCCAAAACUGAG-GC

hsa-miR-30c UGUAAACAUCCUACACUCUCAGC 60.9%

1 23

dme-miR-957 UGAAACCGUCCAAAACUG-AGGC

hsa-miR-30d UGUAAACAUCCCCGACUGGAAG- 60.9%

1 23

dme-miR-957 UGAAACCGUCCAAAACUG-AGGC

hsa-miR-30e UGUAAACAUCCUUGACUGGAAG- 60.9%

**miR-961: dme-miR-961, hsa-miR-133a, hsa-miR-133b**

1 23

dme-miR-961 UUUGAUCACCAGUAACUGAGAU-

hsa-miR-133a UUUGGUCCCCUUCAACC-AGCUG 60.9%

1 23

dme-miR-961 UUUGAUCACCAGUAACUGAGAU-

hsa-miR-133b UUUGGUCCCCUUCAACC-AGCUA 60.9%

**miR-963 : dme-miR-963, hsa-miR-196a**

1 25

dme-miR-963 ACAAGGUAAAUAUCAGGUUGUUUC-

hsa-miR-196a --UAGGUAG-UUUCAUGUUGUUGGG 60.0%

**miR-964: dme-miR-964, hsa-miR-651**

1 26

dme-miR-964 -UUAGAAUAGGGGAGCUUAACUU---

hsa-miR-651 UUUAGGAUA----AGCUUGACUUUUG 61.5%

**miR-966: dme-miR-966, hsa-miR-574-5p, hsa-miR-637,**

**hsa-miR-760**

1 24

dme-miR-966 --UGUGGGUUGU-GGGCUGUGUGG

hsa-miR-637 ACUGGGGGCUUUCGGGCUCUGCGU 62.5%

1 26

dme-miR-966 ----UGUGGGU-UGUGGGCUGUGUGG

hsa-miR-574-5p UGAGUGUGUGUGUGUGAG-UGUGU-- 61.5%

1 23

dme-miR-966 UGUGGGUUGUGGG-CUGUGUGG-

hsa-miR-760 ---CGGCUCUGGGUCUGUGGGGA 60.9%

**miR-968: dme-miR-968, hsa-miR-325**

1 26

dme-miR-968 -UAAGUAG-UAUCCAUUAAAGGGUUG

hsa-miR-325 CCUAGUAGGUGUCCAGUAA-GUGU-- 61.5%

**miR-974: dme-miR-974, hsa-miR-516a-5p**

1 23

dme-miR-974 -AAGCGAGCAAAGAAGUAGUAUU

hsa-miR-516a-5p UUCUCGAGGAAAGAAGCACUUUC 60.9%

**miR-976: dme-miR-976, hsa-miR-450b-3p**

1 23

dme-miR-976 -UUGGAUUAGUUAUCAUCAAUGC

hsa-miR-450b-3p UUGGGAUCAUUUUGCAUCCAUA- 60.9%

**miR-977: dme-miR-977, hsa-let-7a, hsa-let-7b, hsa-let-7c,**

**hsa-let-7e, hsa-let-7f, hsa-let-7i, hsa-miR-98**

1 23

dme-miR-977 UGAGAUAUUCACGUUGUCUAA--

hsa-let-7a UGAGGUAGU-AGGUUGUAUAGUU 65.2%

1 23

dme-miR-977 UGAGAUAUUCACGUUGUCUAA--

hsa-let-7b UGAGGUAGU-AGGUUGUGUGGUU 60.9%

1 23

dme-miR-977 UGAGAUAUUCACGUUGUCUAA--

hsa-let-7c UGAGGUAGU-AGGUUGUAUGGUU 60.9%

1 23

dme-miR-977 UGAGAUAUUCACGUUGUCUAA--

hsa-let-7e UGAGGUAGG-AGGUUGUAUAGUU 60.9%

1 23

dme-miR-977 UGAGAUAUUCACGUUGUCUAA--

hsa-let-7f UGAGGUAGU-AGAUUGUAUAGUU 60.9%

1 23

dme-miR-977 UGAGAUAUUCACGUUGU-CUAA-

hsa-let-7i UGAGGUAGU-AGUUUGUGCUGUU 60.9%

1 23

dme-miR-977 UGAGAUAUUCACGUUGUCUAA--

hsa-miR-98 UGAGGUAGU-AAGUUGUAUUGUU 60.9%

**miR-978 : dme-miR-978, hsa-miR-192***

1 23

dme-miR-978 -UGUCCAGUGCCGUAAAUUGCAG

hsa-miR-192* CUG-CCAAUUCCAUAGGUCACAG 60.9%

**miR-980: dme-miR-980, hsa-miR-22, hsa-miR-103,**

**hsa-miR-107, hsa-miR-345**

1 22

dme-miR-980 UAGCUGCCUUGUGAAGGGCUUA

hsa-miR-22 AAGCUGCCAGUUGAAGAACUGU 63.6%

1 24

dme-miR-980 UAGCUGCCUUGUGAAGGGCUUA--

hsa-miR-103 -AGCAGCAUUGUACAGGGCUAUGA 62.5%

1 24

dme-miR-980 UAGCUGCCUUGUGAAGGGCUUA--

hsa-miR-107 -AGCAGCAUUGUACAGGGCUAUCA 62.5%

1 25

dme-miR-980 UAGCUG---CCUUGUGAAGGGCUUA

hsa-miR-345 --GCUGACUCCUAGUCCAGGGCUC- 60.0%

**miR-982: dme-miR-982, hsa-miR-620**

1 23

dme-miR-982 UCCUGGACAAAUAUGAAGUAAAU

hsa-miR-620 --AUGGAGAUAGAUAUAG-AAAU 60.9%

**miR-984: dme-miR-984, hsa-let-7b, hsa-let-7c, hsa-let-7e,**

**hsa-let-7i, hsa-miR-98**

1 24

dme-miR-984 UGAGGUAAAUACGGUUGGAAUUU-

hsa-let-7c UGAGGUAG-UA-GGUUGUAUGGUU 66.7%

1 24

dme-miR-984 UGAGGUAAAUACGGUUGGA-AUUU

hsa-let-7e UGAGGUAGGA--GGUUGUAUAGUU 66.7%

1 24

dme-miR-984 UGAGGUAAAUACGGUUGGAAUUU-

hsa-let-7i UGAGGUAG-UA-GUUUGUGCUGUU 62.5%

1 24

dme-miR-984 UGAGGUAAAUACGGUUGGAAUUU-

hsa-miR-98 UGAGGUAGUAA--GUUGUAUUGUU 62.5%

1 23

dme-miR-984 UGAGGUAAAUACGGUUGGAAUUU

hsa-let-7b UGAGGUAG-UAGGUUGUGUGGUU 60.9%

**miR-987: dme-miR-987, hsa-miR-26b, hsa-miR-545***

1 25

dme-miR-987 UAAAGUAAAUAGUCUGGAUUGAUG-

hsa-miR-545* -UCAGUAAAU-GUUUAU-UAGAUGA 60.0%

1 25

dme-miR-987 -UAAAGUAAAUAGUCUGGAUUGAUG

hsa-miR-26b UUCAAGUAAUU---CAGGAUAGGU- 60.0%

**miR-990: dme-miR-990, hsa-miR-32, hsa-miR-506,**

**hsa-miR-513a-3p**

1 23

dme-miR-990 -AUU-CACCGUUCUGAGUUGGCC

hsa-miR-32 UAUUGCACAUUACUAAGUUGCA- 60.9%

1 23

dme-miR-990 --AUUCACCGUUCUGAGUUGGCC

hsa-miR-506 UAAGGCACCCUUCUGAGUAGA-- 60.9%

1 25

dme-miR-990 ----AUUCACCGUUCUGAGUUGGCC

hsa-miR-513a-3p UAAAUUUCACCUUUCUGAGAAGG-- 60.0%

**miR-991: dme-miR-991, hsa-let-7a, hsa-let-7c, hsa-let-7d,**

**hsa-let-7e, hsa-let-7f, hsa-let-7g, hsa-let-7i**

1 23

dme-miR-991 UUAAAGUUGUAGUUUGGAAAGU-

hsa-let-7g -UGAGGUAGUAGUUUGUACAGUU 69.6%

1 23

dme-miR-991 UUAAAGUUGUAGUUUGGAAAGU-

hsa-let-7a -UGAGGUAGUAGGUUGUAUAGUU 65.2%

1 23

dme-miR-991 UUAAAGUUGUAGUUUGGAAAGU-

hsa-let-7f -UGAGGUAGUAGAUUGUAUAGUU 65.2%

1 23

dme-miR-991 UUAAAGUUGUAGUUUGGAAAGU-

hsa-let-7c -UGAGGUAGUAGGUUGUAUGGUU 60.9%

1 23

dme-miR-991 UUAAAGUUGUAGUUUGGAAAGU-

hsa-let-7d -AGAGGUAGUAGGUUGCAUAGUU 60.9%

1 23

dme-miR-991 UUAAAGUUGUAGUUUGGAAAGU-

hsa-let-7e -UGAGGUAGGAGGUUGUAUAGUU 60.9%

1 23

dme-miR-991 UUAAAGUUGUAGUUUGGAAAGU-

hsa-let-7i -UGAGGUAGUAGUUUGUGCUGUU 60.9%

**miR-993: dme-miR-993, hsa-miR-10a*, hsa-miR-99a*,**

**hsa-miR-99b***

1 23

dme-miR-993 GAAGCUCGUCUCUACAGGUAUCU

hsa-miR-99a* CAAGCUCGCUUCUAUGGGUCUG- 65.2%

1 23

dme-miR-993 GAAGCUCGUCUCUACAGGUAUCU

hsa-miR-10a* CAAAUUCGUAUCUAGGGGAAUA- 60.9%

1 23

dme-miR-993 GAAGCUCGUCUCUACAGGUAUCU

hsa-miR-99b* CAAGCUCGUGUCUGUGGGUCCG- 60.9%

**miR-995: dme-miR-995, hsa-miR-15a, hsa-miR-15b,**

**hsa-miR-29b**

1 24

dme-miR-995 UAGCACCACAUGAUUCGGCUU---

hsa-miR-15a UAGCAGCACAUAAU--GGUUUGUG 66.7%

1 24

dme-miR-995 UAGCACCACAUGAUUCGGCUU---

hsa-miR-15b UAGCAGCACAUCAU--GGUUUACA 66.7%

1 23

dme-miR-995 UAGCACCACAUGAU-UCGGCUU-

hsa-miR-29b UAGCACCAUUUGAAAUCAGUGUU 65.2%

**miR-998: dme-miR-988, hsa-miR-29a, hsa-miR-29b,**

**hsa-miR-29c**

1 22

dme-miR-998 UAGCACCAUGAGAU-UCAGCUC

hsa-miR-29a UAGCACCAUCUGAAAUCGGUUA 68.2%

1 22

dme-miR-998 UAGCACCAUGAGAU-UCAGCUC

hsa-miR-29c UAGCACCAUUUGAAAUCGGUUA 68.2%

1 23

dme-miR-998 UAGCACCAUGAGAU-UCAGCUC-

hsa-miR-29b UAGCACCAUUUGAAAUCAGUGUU 65.2%

**miR-1000: dme-miR-1000, hsa-miR-581**

1 22

dme-miR-1000 AUAUUGUCCUGUC-ACAGCAGU

hsa-miR-581 -UCUUGUGUUCUCUAGAUCAGU 63.6%

**miR-1001: dme-miR-1001, hsa-miR-331-5p**

1 22

dme-miR-1001 -UGGGUAAACUCCCAAGGAUCA

hsa-miR-331-5p CUAGGUAUGGUCCCAGGGAUCC 68.2%

**miR-1003: dme-miR-1003, hsa-miR-192***

1 23

dme-miR-1003 UCUCACAUUUACAUAU-UCACAG

hsa-miR-192* -CUGCCAAUUCCAUAGGUCACAG 69.6%

**miR-1005: dme-miR-1005, hsa-miR-542-5p**

1 23

dme-miR-1005 UCUGGAAUCUUUAAUUCGC-AG-

hsa-miR-542-5p UCGGGGAUCAUCAUGUCACGAGA 60.9%

**miR-1010: dme-miR-1010, hsa-miR-412**

1 26

dme-miR-1010 -UUUCACCUAUCGUUCCAUUUGCAG-

hsa-miR-412 ACUUCACCU---GGUCCACUAGCCGU 61.5%

**miR-1011: dme-miR-1011, hsa-miR-137**

1 23

dme-miR-1011 UUAUUGGUUCAAAUCGCUCGCAG

hsa-miR-137 UUAUUGCUUAAGAAUACGCGUAG 65.2%
